# Palladium‐Catalyzed Annulations of an Anti‐Bredt Olefin

**DOI:** 10.1002/anie.3724263

**Published:** 2026-07-26

**Authors:** Zach G. Walters, Marie Deliaval, Aimee Long, Neil K. Garg

**Affiliations:** ^1^ Department of Chemistry and Biochemistry University of California Los Angeles California USA

**Keywords:** annulation, heterocycle synthesis, homogeneous catalysis, palladium, strained molecules

## Abstract

A famous class of alkene‐containing compounds that have intrigued chemists for more than a century are anti‐Bredt olefins (ABOs). ABOs are a subclass of bridgehead alkenes that reside in small ring systems, thus rendering them unstable and historically challenging to leverage in chemical synthesis. The present study describes metal catalyzed reactions of a [2.2.1]‐bridged bicyclic ABO, wherein two in situ‐generated species, both present in low concentration and generated transiently, undergo a productive reaction. The transformation uses palladium catalysis to unite the aforementioned ABO with indole‐based trapping partners, thus delivering unusual heterocyclic products. The scope of the reaction is reported, as well as computations concerning the putative Pd‐bound ABO species. The latter suggests that the short‐lived ABO is stabilized by Pd via the energetically favorable formation of an intermediate palladacycle. This methodology is expected to prompt the further development of unconventional reactions, including metal‐catalyzed processes of ABOs and other transient olefins.

## Graphical abstract

We report metal‐catalyzed trappings of a [2.2.1]‐bridged bicyclic anti‐Bredt olefin (ABO). The methodology yields unusual heterocyclic products and is expected to prompt the further development of unconventional reactions, including metal‐catalyzed processes of ABOs and other strained olefins.

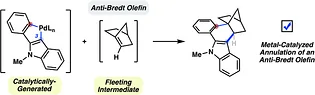

## 1 Introduction

**FIGURE 1:**
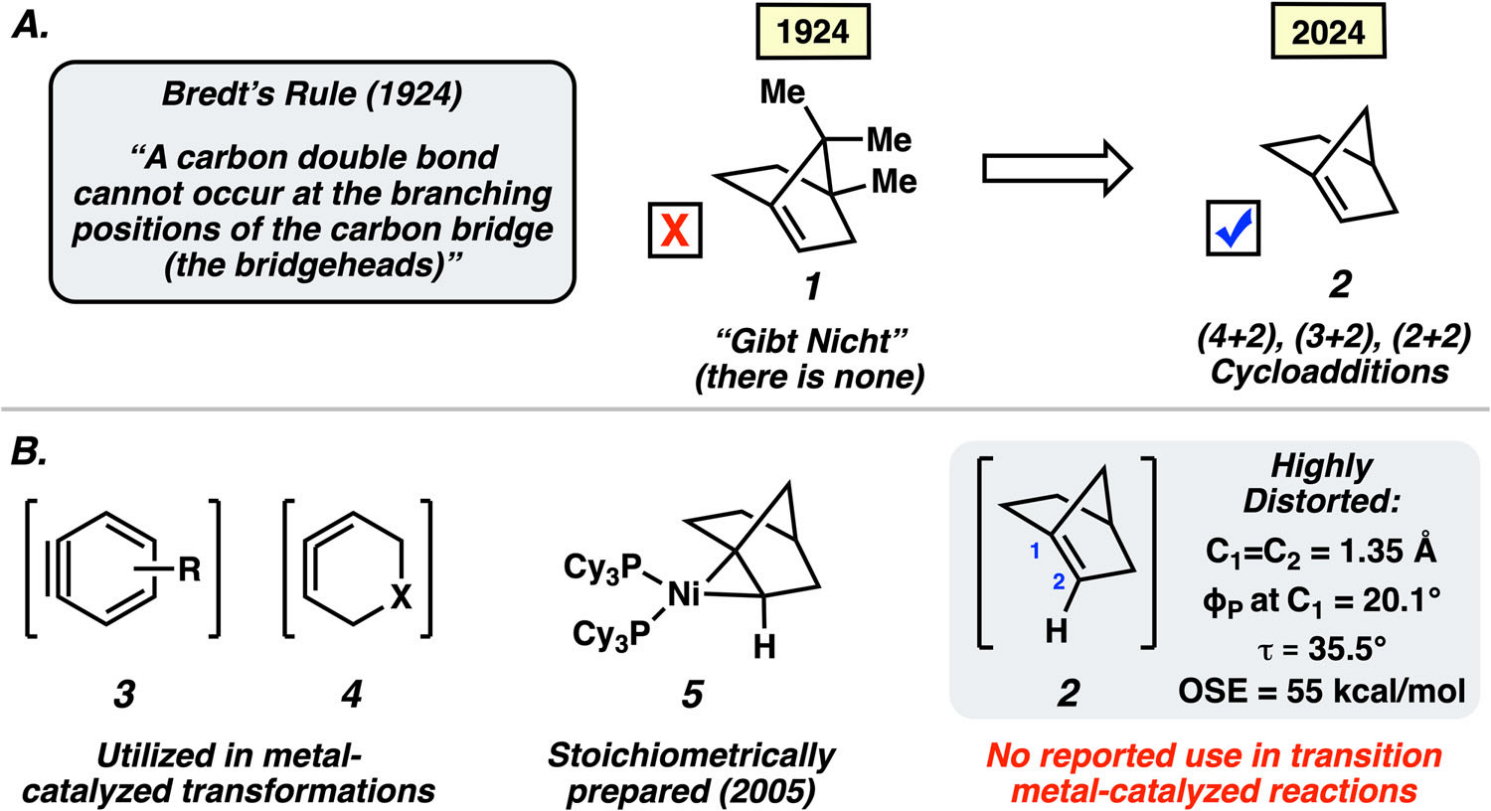
(A) Historical context for ABOs, including the 1924 codification of Bredt's rule and our 2024 study of ABO cycloadditions. (B) Compounds **3**–**5** have been utilized in relevant studies of transition metals and strained intermediates; structural properties of free ABO **2**. Cy = cyclohexyl, ɸ_p_ = pyramidalization angle, 𝜏 = twist angle, and OSE = olefin strain energy.

Bridgehead alkenes are an important and well‐studied class of compounds that possess one alkene carbon at the bridgehead position of a bridged bicyclic scaffold [1, 2, 3, 4, 5]. A notorious subtype of these are known as anti‐Bredt olefins (ABOs), which, in turn, have long inspired and intrigued the chemical community [3, 4, 6, 7, 8, 9, 10, 11, 12, 13, 14, 15, 16, 17, 18, 19]. Their name stems from their violation of “Bredt's Rule,” a classic rule in organic chemistry that arose from empirical observations made in the context of dehydration reactions, wherein the alkene isomer **1** (Figure 1A) was not observed in reactions where it could have been a possible product [6]. In modern times, IUPAC defines ABOs as being bridgehead alkenes that specifically reside in rings not “large enough to accommodate the double bond without excessive strain” [20, 21, 22]. Many studies have sought to quantify how small the ring must be to satisfy this criterion, considering both the ring size [23] and olefin strain [24] of the system, with there being some variation and refinement in the literature over time [23, 25, 26]. Informed by both computations [24] and experimental results [25, 27], it is reasonable to conclude that a bridgehead alkene is likely “unstable” (and, therefore, an ABO) when the sum of non‐bridgehead atoms in the skeleton of the bridged bicycle is less than 7, or the olefin strain energy is greater than 21 kcal/mol [21, 28].

Although ABOs were largely considered inaccessible for decades, several pioneering studies demonstrated that Bredt's Rule could be violated in small rings in specific cases, particularly through groundbreaking studies from the laboratories of Keese [10, 11], Gano [12], Chan [13], Wiberg [14], and others [7]. In recent years, experimental [19] and computational efforts [29, 30, 31, 32, 33] have further expanded the horizons of ABO chemistry. Recent experimental studies include the exploitation of anti‐Bredt enones in remarkable total synthesis campaigns [34, 35, 36] and the impressive generation and trapping of 1,2‐diamantene [37]. In 2024, our laboratory leveraged non‐isolable ABOs (e.g., **2**) in chemical synthesis through in situ generation and trapping in (4+2), (3+2), and (2+2) cycloadditions [19]. The approach we relied upon [19, 38], which is also utilized in the present study, builds upon the seminal findings of Kobayashi [39], Johnson [40], and others, who showed that silyl (pseudo)halides (now commonly known as Kobayashi precursors [41]) can be used to generate strained intermediates, including arynes and cyclic allenes in situ.

With ABOs now accessible under mild reaction conditions, one can consider other modes of ABO reactivity and potentially access compounds that possess novel structures. The subject of the current study is a metal‐catalyzed process. Although such reactions are unknown for ABOs and would represent a new mode of reactivity, it is notable that metal‐catalyzed reactions of other strained intermediates, such as arynes **3** and cyclic allenes **4,** have been discovered (Figure 1B) [41, 42, 43, 44]. These reactions are unusual and challenging in that a strained intermediate, generated transiently, can undergo productive reaction with a catalytically‐generated organometallic intermediate. Stoichiometric organometallic complexes of arynes, cyclic alkynes, and cyclic allenes are also known, [45, 46, 47, 48, 49] including recent studies by Roberts [50]. Additionally, an elegant study by Takasu and coworkers describes the use of strained trans‐alkenes (generated transiently in medium‐sized rings) being leveraged in catalysis [51]. The corresponding organometallic chemistry of ABOs is less explored in small bridged bicyclic scaffolds [52, 53, 54, 55, 56], but bridged bicyclic metallocyclopropane **5**, reported in 2005, is akin to a metal‐stabilized [2.2.1]‐bridged bicyclic ABO [57]. We envisioned species related to **5** could plausibly be generated in situ and trapped in catalytic processes, validating the use of ABOs in catalytic transformations and furnishing value‐added products.

## 2 Results and Discussion

For the current study, we focus efforts on ABO **2** (Figure 1B). This ABO possesses the [2.2.1] (1‐norbornene) ring system much like Bredt's early studies, and can be generated from a readily‐accessible Kobayashi precursor, which itself stems from a concise, scalable five‐step sequence [19]. The structure and properties of **2** have been studied previously using computations, showing that **2** has significant geometric distortion compared to a typical alkene. Key features include an elongated C1═C2 bond (1.35 Å vs a typical C═C bond at ∼1.32 Å), a high degree of pyramidalization at C1 (20.1°), and significant twisting (35.5°) [19]. As such, ABO **2** has weakened π‐bonding and only exists transiently. The olefin strain energy (OSE) of **2** is 55 kcal/mol, which exceeds the strain energy of both arynes (∼50 kcal/mol) and cyclic allenes (∼30 kcal/mol) [58, 59, 60]. Overall, ABO **2** presents a challenging and intriguing target for use in a metal‐catalyzed transformation.

**FIGURE 2:**
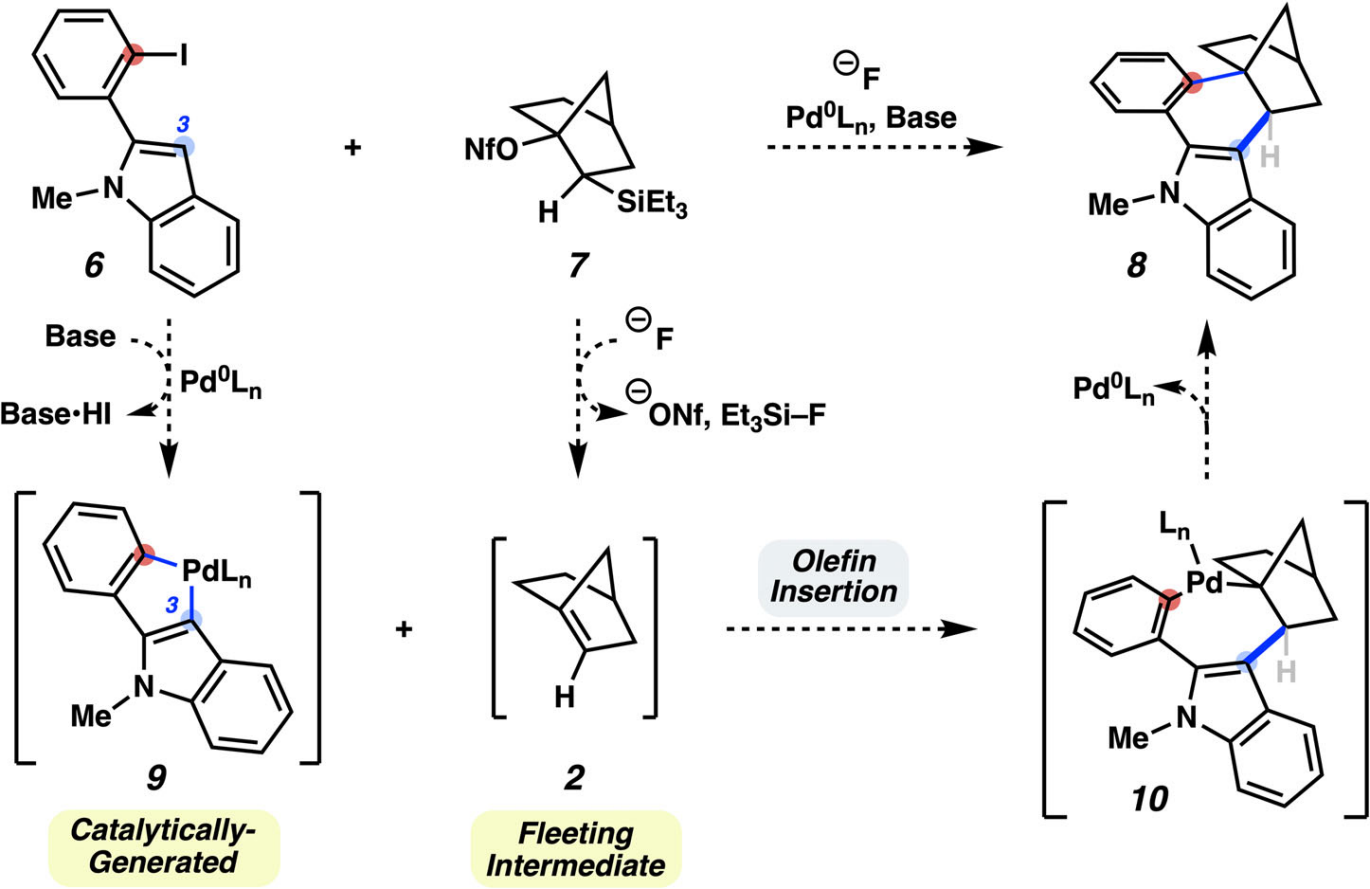
Reaction design for the metal‐catalyzed reactions of an ABO, which relies on catalytically‐generated species **9** and fleeting ABO **2** being generated concurrently and undergoing a productive reaction. ONf = nonafluorobutanesulfonate and L_n_ = ligand.

To establish the use of ABOs in transition metal‐catalyzed reactions, we designed the reaction sequence shown in Figure 2. Inspired by the Pd‐catalyzed annulation of arynes with biaryl halides developed by Larock and coworkers [61, 62], we envisioned that treatment of biaryl halide **6** and silyl nonaflate **7** with a fluoride source and a palladium catalyst could yield annulated product **8**. Mechanistically, in the presence of Pd (0) and a suitable base, indolyl iodide **6** would be converted to palladacycle **9** through oxidative addition into the C─I bond, followed by indole cyclization at C3. The heightened nucleophilicity of the indole compared to a typical aryl ring [63] could facilitate the palladacycle formation, while also enabling access to heterocyclic products. Simultaneously, fluoride‐mediated 1,2‐elimination of the silyl nonaflate **7**, driven by the favorable formation of the Si─F bond in Et_3_Si─F and excellent leaving group ability of the nonaflate anion [38], would generate the highly reactive, fleeting intermediate **2** [19]. The concurrent generation of **9** and **2** in sufficient concentrations would enable coordination and olefin insertion to form palladacycle **10**, provided that this process outcompetes decomposition of both species. Reductive elimination would then afford annulated product **8** and regenerate the active Pd(0) catalyst.

With the aforementioned reaction design in mind, we studied the reaction of biaryl iodide **6** with ABO precursor **7** [19] in the presence of a fluoride source and palladium catalyst (Table 1). A number of variables were probed, including stoichiometry, Pd precatalyst, ligand, fluoride source, additives, solvent, temperature, and time, and select results are depicted (see Supporting Information, Section I.B for further optimization experiments). The conditions shown in entry 1 utilize two equivalents of ABO precursor **7**, Pd(dba)_2_ as precatalyst, P(*o*‐tol)_3_ as ligand, CsF as fluoride source, and toluene‐acetonitrile as solvent in an effort to parallel conditions used in aryne annulations [61, 62, 64]. Unfortunately, neither of the expected annulated products **8** or **iso‐8** were observed. As complete consumption of the silyl nonaflate **7** was not seen, we tested the additive *n*Bu_4_NBr that would serve as a fluoride‐solubilizing agent [19]. Upon addition of three equivalents of this additive, we were delighted to observe the desired products **8** and **iso‐8** in a combined yield of 39% (1.1:1 ratio; entry 2). Minor modifications were then identified to increase the yield, including removing the acetonitrile cosolvent (entry 3) and increasing the equivalents of ABO precursor **7** to three (entry 4). Each of these changes led to some improvement, presumably by further modulating the rate of ABO generation and, therefore, the amount of ABO in solution available to react with the putative palladacyclic intermediate (see **9**, Figure 2). Finally, it was found that the addition of Cs_2_CO_3_ as a mild base gave our optimal conditions, delivering **8** and **iso‐8** in 54% yield [65, 66]. This yield was reasonable, given the complexity of the transformation and the challenges associated with harnessing two transient, in situ‐generated species.

**TABLE 1:** Select results for optimization of ABO annulation to give **8** and **iso‐8**.

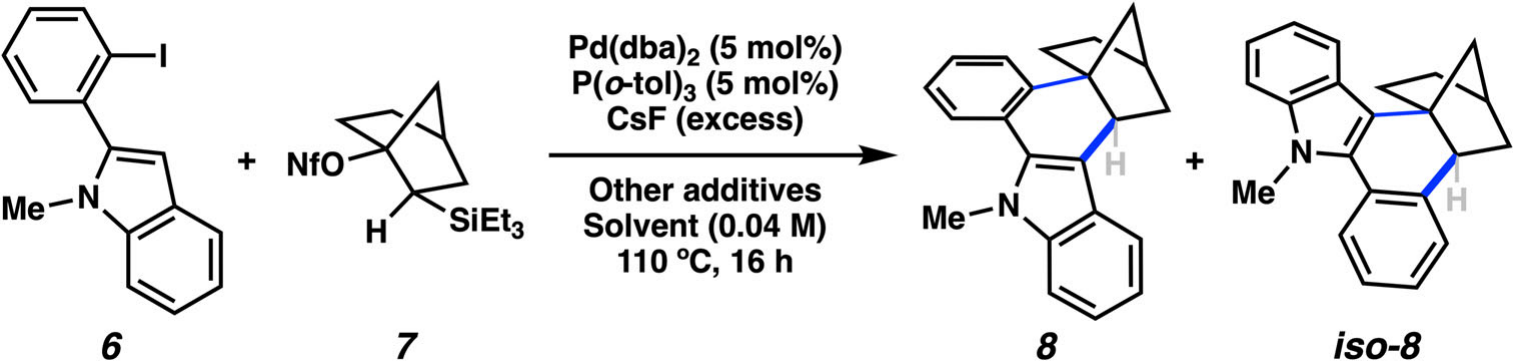				
Entry	Equiv 7	Solvent	Other additives	Yield 8 + iso‐8 (ratio)
1	2	PhMe/MeCN (9:1)	—	0%
2	2	PhMe/MeCN (9:1)	*n*Bu_4_NBr (3 equiv)	39% (1.1:1)
3	2	PhMe	*n*Bu_4_NBr (3 equiv)	44% (1.3:1)
4	3	PhMe	*n*Bu_4_NBr (3 equiv)	50% (1.3:1)
5	3	PhMe	*n*Bu_4_NBr (3 equiv) Cs_2_CO_3_ (3 equiv)	54% (1.3:1)

ratio = ratio of constitutional isomers formed, ONf = nonafluorobutanesulfonate, dba = dibenzylideneacetone, P(*o*‐tol)_3_ = tri(*o*‐tolyl)phosphine, PhMe = toluene, MeCN = acetonitrile, *n*Bu_4_NBr = tetra‐*n*‐butylammonium bromide.

Several control experiments merit discussion, some of which explore the role of ligands, as well as challenges that were observed. With regard to the former, both the Pd catalyst and fluoride are essential for the desired transformation to take place (see Supporting Information, Section I.B). Furthermore, if the annulation is performed in the presence of a competent diene, the corresponding Diels–Alder cycloadduct is formed, which is consistent with the generation of ABO **2** under the reaction conditions [19, 67]. Ligand effects are also notable. In the absence of a phosphine ligand, the annulation still occurs, albeit in lower yields (ca. 35%). Variation of the ligand generally led to similar or lower yields, without impacting regioselectivity (see Supporting Information, Section I.B for details). Thus, we suspect that the ligand is likely not bound to Pd in the key C─C bond formation step that governs regioselectivity, as is further discussed below (see Figure 4). Some practical limitations are also apparent, namely, the requirement for the use of excess ABO precursor **7** [64] and the formation of two isomeric products in nearly equimolar ratios [68]. Along with the aforementioned investigation of ligand effects, other parameters that might impact selectivity were altered, including reaction temperature and the precatalyst utilized. However, no changes in product ratio were observed, as we show in the Supporting Information (see Section I.B for details).

**FIGURE 3:**
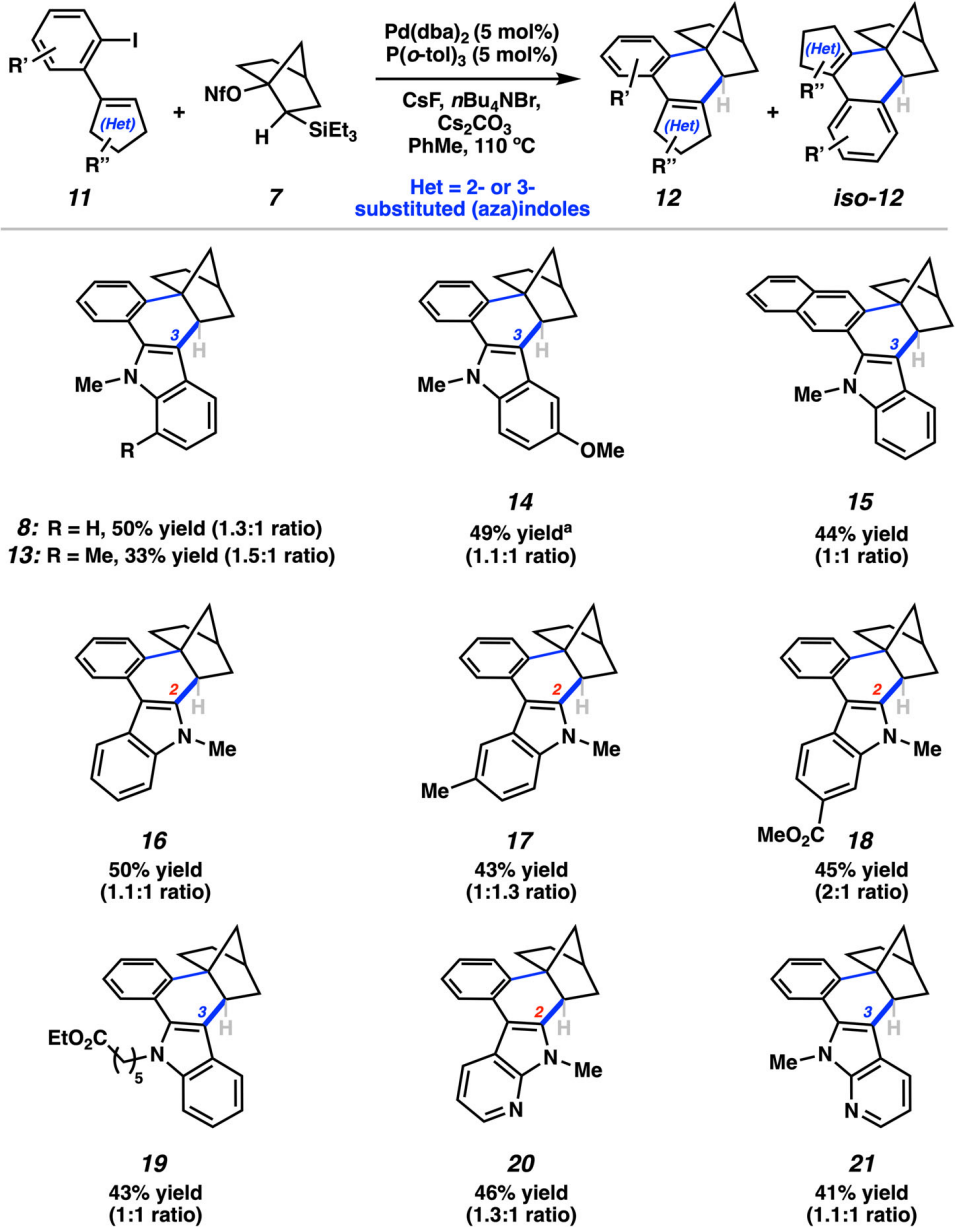
Scope of the annulation reaction. Yields reflect amounts of isolated products. Ratio refers to the ratio of **12** to **iso‐12**. Conditions, unless otherwise stated: biaryl iodide **11** (1 equiv), silyl nonaflate **7** (3 equiv), Pd(dba)_2_ (5 mol%), P(*o*‐tol)_3_ (5 mol%), CsF (30 equiv), *n*Bu_4_NBr (3 equiv), Cs_2_CO_3_ (3 equiv), toluene (0.04 M), 110°C for 16 h. ^a^Reaction was conducted at 125°C in a sealed vessel. ONf = nonafluorobutanesulfonate, dba = dibenzylideneacetone, P(*o*‐tol)_3_ = tri(*o*‐tolyl)phosphine, PhMe = toluene, *n*Bu_4_NBr = tetra‐*n*‐butylammonium bromide.

The scope of the annulation reaction is shown in Figure 3 [69]. Several 2‐arylindoles were evaluated and found to undergo the desired annulation at C3 of the indole. Products obtained include the parent system **8**, methyl‐substituted product **13**, methoxy‐substituted scaffold **14,** and naphthyl ring‐containing product **15** (and their respective constitutional isomers). Isomeric substrates with respect to the biaryl linkage could also be employed, leading to C2‐annulated products **16** [70], **17**, and **18**. Notably, the latter example highlights the tolerance of the electron‐withdrawing ester functional group. Additionally, as shown by **19**, a substrate bearing an *N*‐substituent analogous to linkers commonly seen in molecules such as cyanine dyes [71] could be employed. Lastly, isomeric azaindole substrates were subjected to the transformation, delivering **20** and **21**.

An attractive feature of this methodology is that it delivers unusual product structures, namely previously unknown heterocycles that would be difficult to access by other methods [72]. Each product contains both aromatic and aliphatic elements, featuring six interconnected rings, a conjugated heterocyclic biaryl linkage, a bridged [2.2.1] bicycle, and three stereocenters, one of which is benzylic and quaternary. Compounds that have both aromatic and aliphatic qualities have shown interesting solubility and optical properties [73, 74], as well as potentially being valuable in medicinal contexts [75], thus setting the stage for potential future applications of the products accessible from metal‐catalyzed ABO annulations.

**FIGURE 4:**
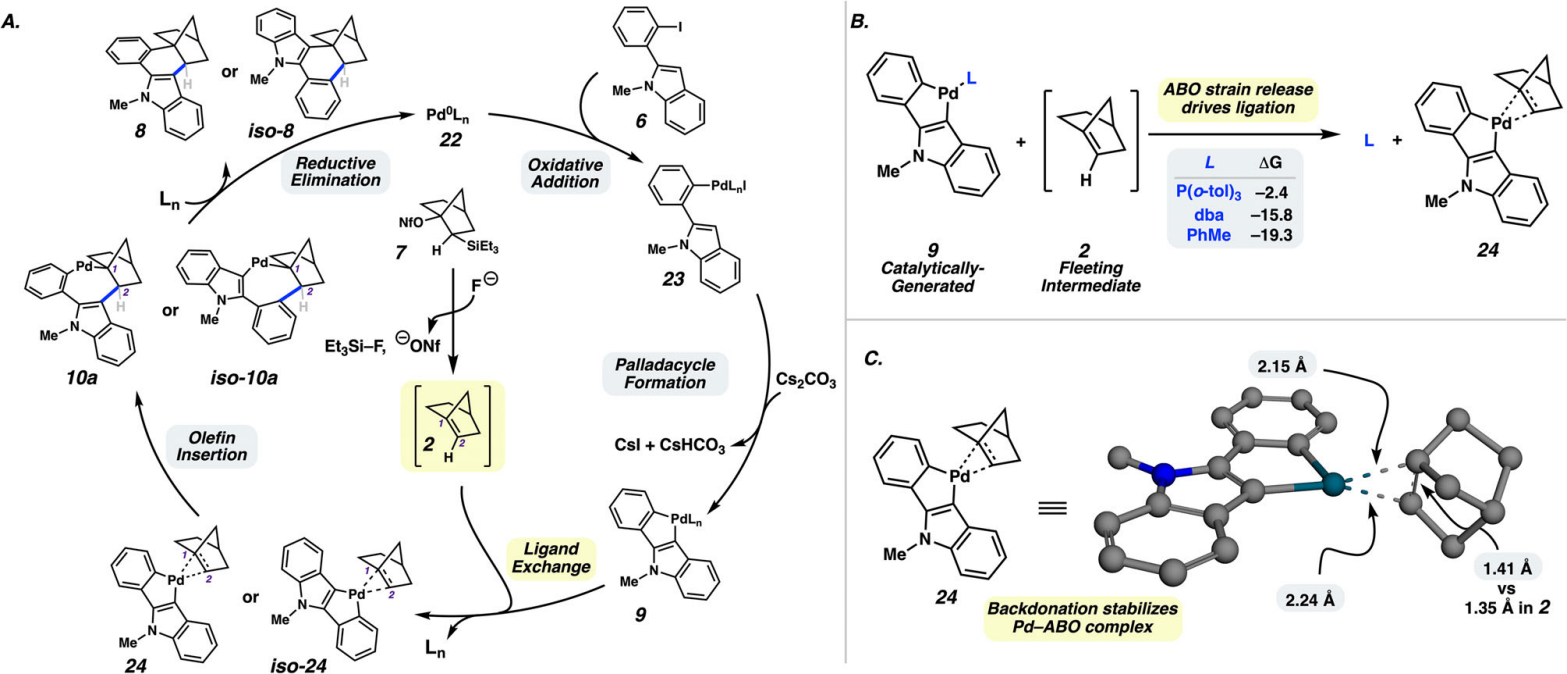
(A) Plausible catalytic cycle for the metal‐catalyzed annulation of ABO **2**; L_n_ is used to describe a ligand, such as phosphine, dba, or solvent. (B) Calculated thermodynamics show that ligand exchange with ABO **2** is energetically favorable. Energies in kcal/mol at the M06‐L/6‐311++G(d,p)/SDD(Pd)//B3LYP‐D3(BJ)/6‐31G(d)/LANL2DZ(Pd) level of theory. (C) Computed structure of complex **24** at the M06‐L/6‐311++G(d,p)/SDD(Pd)//B3LYP‐D3(BJ)/6‐31G(d)/LANL2DZ(Pd) level of theory suggests that metal complexation relieves ABO strain. L_n_ = ligand, ONf = nonafluorobutanesulfonate, dba = dibenzylideneacetone, P(*o*‐tol)_3_ = tri(*o*‐tolyl)phosphine, and PhMe = toluene.

A number of distinct mechanistic pathways for this annulation can be envisioned, and we provide an in‐depth discussion in the Supporting Information (see Section II.C). One plausible catalytic cycle for the palladium‐catalyzed annulation of ABO **2** with iodide **6**, leading to products **8** and **iso‐8**, is depicted in Figure 4A. The active Pd(0) catalyst **22** undergoes oxidative addition with biaryl iodide **6** to furnish intermediate **23**. Next, base‐promoted palladacycle formation could occur, giving rise to **9**. Importantly, similar cyclopalladation processes of biaryl halides, including indolyl systems, are known to occur under similar conditions, lending credence to this mechanistic step [76, 77]. Concomitantly, fluoride‐mediated generation of **2** can occur from precursor **7** through direct elimination of the fluorosilicate derivative of **7** [38]. Complexation of **2** with palladacycle **9** would occur in a facially‐selective manner, guided by the bicyclic framework of ABO **2** (i.e., complexation would occur on the external face of the bridged bicycle, as the interior is sterically obstructed). This step would yield isomeric Pd‐ABO complexes **24** and **iso‐24**, which differ based on the orientation of the ABO fragment relative to the palladacycle [78]. In this step, we surmise that ligand displacement takes place to release L_n_, given that the reaction still occurs in the absence of added ligand and variation of ligand does not impact regioselectivity. Olefin insertion is then expected to give rise to **10a** and **iso‐10a**. Alternative olefin insertion pathways cannot be ruled out, such as a pathway involving direct reaction of oxidative adduct **23** with ABO **2**, and are further discussed in the Supporting Information, Section II.C. Finally, reductive elimination delivers annulated products **8** and **iso‐8** and regenerates the active catalyst **22**.

As acquiring experimental support for the proposed reaction mechanism was challenging due to the fleeting nature of ABO **2**, we performed computations on the key mechanistic step wherein palladacycle **9** and ABO **2** would unite to form Pd‐ABO complex **24**; following olefin insertion and reductive elimination, the major product, **8**, would arise (Figure 4B). As discussed earlier, this is a challenging step as both species would be present in small quantities, with at least ABO **2** being short‐lived [79]. We postulated that palladacycle **9** would be ligand‐bound to stabilize the intermediate catalytic species, but that this ligand is released upon complexation with **2,** as ligand choice does not influence regioselectivity. Thus, the thermodynamics for ligand exchange were evaluated for the displacement of plausible ligands present in the reaction, P(*o*‐tol)_3_, dba, or PhMe, from organometallic intermediate **9** [80, 81]. We found that complexation of ABO **2** to form Pd‐ABO complex **24** is thermodynamically favorable in all evaluated cases, with computed ΔG values of –2.4, –15.8, and –19.3 kcal/mol, respectively. The former case is notable, as phosphine ligands commonly displace olefin ligands, such as dba or COD, from Pd complexes [82, 83]. In our case, the high binding affinity of ABO **2** to the Pd metal likely results from a reduction in olefin strain that occurs upon ligation to give **24**. The thermodynamics of a plausible alternative ligand exchange, proceeding directly through oxidative addition adduct **23**, were found to follow the same trend (see Supporting Information, Sections II.C for discussion of this mechanism and II.G for computational findings).

The favorable thermodynamics for the formation of Pd‐ABO complex **24** can be further understood by considering the computed structure of Pd‐ABO complex **24** (Figure 4C). Importantly, in comparison to ABO **2**, the C─C bond in **24** is elongated (1.41 Å vs. 1.35 Å). Additionally, the Pd─C bonds are relatively short (2.15 and 2.24 Å in **24** vs. 2.28 Å in PdCl_2_(ethylene) [84, 85]). Both of these findings support a high degree of backdonation from Pd, which helps to stabilize the highly strained ABO. Similar strained intermediate bond lengthening effects have been reported by Roberts [50] and Vicic [57] in their experimental studies of Ni‐bound hetarynes and bridged bicyclic metallocyclopropane **5** (see Figure 1), respectively. Additionally, EDA‐NOCV analysis of the Pd–ABO bonding interactions, as well as investigation of the bond order of the C═C bond in **24** supported strong backdonation (see Supporting Information, Sections II.I and II.J for details). Thus, stabilization of the ABO via palladium complexation is likely important for the success of the palladium‐catalyzed annulation of ABO **2** by providing a driving force for palladacycle **9** and ABO **2** to react en route to **8**.

## 3 Conclusion

This study provides the first metal‐catalyzed annulation of ABO **2**, highlighting the evolution of synthetic chemistry related to Bredt's rule over the last century. We hope our findings will prompt the further development of unconventional reactions, including metal‐catalyzed processes, of ABOs and other transient olefins.

## Author Contributions

**Zach G. Walters**: conceptualization, investigation, writing – original draft, methodology, validation, writing – review and editing, data curation, formal analysis. **Marie Deliaval**: investigation, writing – original draft, methodology, validation, writing – review and editing, data curation, formal analysis. **Aimee Long**: investigation, writing – original draft, methodology, validation, writing – review and editing, data curation, formal analysis. **Neil K. Garg**: conceptualization, investigation, writing – original draft, funding acquisition, writing – review and editing, supervision, project administration, formal analysis.

## Conflicts of Interest

The authors declare no conflicts of interest.

## Supporting information




**Supporting File**: anie73780‐sup‐0001‐SuppMat.pdf.

## Data Availability

The data that supports the findings of this study are available in the Supporting Information of this article.
