# Supplementary material for: Palladium‐Catalyzed Annulations of an Anti‐Bredt Olefin
Source: Angew Chem Int Ed Engl. 2026 Jul 26;65(38):e3724263. doi: 10.1002/anie.3724263 (PMC13454927; doi:10.1002/anie.3724263)
Supplement: Supplementary file 1 [file ANIE-65-e3724263-s001.pdf]

Palladium-Catalyzed Annulations of an Anti-Bredt Olefin

Zach G. Walters, Marie Deliaval,† Aimee Long,† and Neil K. Garg*

Garg Laboratory, Department of Chemistry and Biochemistry, University of California Los Angeles, California 90095

Supporting Information – Table of Contents

Materials and Methods **S2**
Part I. Experimental Procedures **S4**
A. Synthesis of Biaryl Halides **S4**
B. Annulations of Anti-Bredt Olefins **S15**
C. NMR Analysis of 20 and *iso*-20 .... **S33**

NMR Spectra **S34**

Part II. Computational Section **S77**
A. Computational Methods **S77**
B. Complete Reference for Gaussian 16 .... **S77**
C. Details of Olefin Insertion Pathways **S77**
D. Comparison of Product Isomers 16 and iso-16 to Assess Steric Factors **S81**
E. Evaluation of Ligand Exchange **S82**
F. Ligand Exchange in Cyclic Complex **S83**
G. Ligand Exchange in Acyclic Oxidative Addition Complex **S84**
H. Structural Analysis of Pd–Ethylene Complex 64 .... **S86**
I. EDA-NOCV Analysis of Pd Complexes 24 and 64 .... **S86**
J. Bond Order of ABO Fragment and Pd Complex **S89**
K. Cartesian Coordinates of Optimized Structures **S90**

References **S113**

Materials and Methods. Unless stated otherwise, reactions were conducted in flame-dried glassware under an atmosphere of nitrogen, and commercially obtained reagents were used as received unless otherwise specified. Anhydrous solvents were either freshly distilled or passed through activated alumina columns, unless otherwise stated. Sparging was conducted by bubbling nitrogen gas through a solution for 15 minutes. Non-commercially available substrates were synthesized according to known preparations or following protocols specified in the Experimental Procedures. Indole **25**, sodium hydride (NaH), tetrabutylammonium fluoride (TBAF) 1.0 M THF solution, indole **36**, *n*-butyllithium 2.5 M solution in hexanes (*n*-BuLi), tri(*o*-tolyl)phosphine (P(*o*-tol)$_3$), phenylhydrazine ($PhNHNH_2$), polyphosphoric acid (PPA), anthracene, Pd[P(*o*-tol)$_3$]$_2$Cl$_2$, and mesitylene were obtained from Sigma-Aldrich. Tetrakis(triphenylphosphine)palladium(0) ($Pd(PPh_3)_4$), ($(Pd(PPh_3)_2)Cl_2$), bis(dibenzylideneacetone)palladium(0) ($Pd(dba)_2$), cesium fluoride (CsF), cesium carbonate (Cs_2CO_3), trimethylphosphine (PMe_3) and **L1**–**L6** were obtained from Strem Chemicals. Diiodobenzene (**26**), iodomethane (CH_3I), sodium nitrite ($NaNO_2$), acetic acid (AcOH), and potassium carbonate (K_2CO_3) were obtained from Thermo Fischer Scientific. Tetrabutylammonium bromide (*n*Bu$_4$NBr) was obtained from TCI Chemicals. Ethanol was obtained from Acros Organics. Trifluoroacetic acid (TFA), *p*-toluenesulfonic acid monohydrate (*p*-TsOH), and potassium iodide (KI) were obtained from Oakwood Chemical. Pd[P(*o*-tol)$_3$]$_2$, indoles **34** and **40**, ester **45**, and azaindole **47** were obtained from AmBeed.

Reaction temperatures at or above 23 °C were controlled using an IKAmag temperature modulator and, unless stated otherwise, performed at room temperature (approximately 23 °C). Thin layer chromatography (TLC) was conducted with EMD gel 60 F254 pre-coated plates (0.25 mm) and visualized using a combination of UV light and anisaldehyde or potassium permanganate staining. Preparative thin layer chromatography (TLC) was conducted with EMD gel 60 F254 pre-coated plates (0.5 mm) and visualized using UV light. Silicycle Siliaflash P60 (particle size 40 – 63 µm) was used for flash column chromatography and RediSep Gold Silica (columns (20 – 40 µm) or Redisep Gold C18 columns (20 – 40 µm) were used for automated chromatography on a CombiFlash NextGen 300+ instrument unless otherwise specified. ^{1}H NMR and 2D-NOESY spectra were recorded on Bruker spectrometers (at 400, 500 and 600 MHz) and are reported relative to the residual solvent signal. Data for ^{1}H NMR spectra are reported as follows: chemical shift (δ ppm), multiplicity, coupling constant (Hz), and integration. ^{13}C NMR spectra were

recorded on Bruker spectrometers (125 and 150 MHz) and are reported relative to the residual solvent signal. Data for ^{13}C NMR are reported in terms of chemical shift. ^{1}H NMR yields were determined by addition of an aliquot of a stock solution of mesitylene in $CDCl_3$ to the concentrated crude reaction mixture followed by further dilution with $CDCl_3$ and subsequent ^{1}H NMR analysis. Uncorrected melting points were measured using a Digimelt MPA160 melting point apparatus. IR spectra were recorded on a Perkin-Elmer UATR Two FT-IR spectrometer and are reported in terms of frequency absorption (cm^{-1}). DART-MS spectra were collected on a Thermo Exactive Plus MSD (Thermo Scientific) equipped with an ID-CUBE ion source and a Vapur Interface (IonSense Inc.). Both the source and MSD were controlled by Excalibur software v. 3.0. The analyte was spotted onto OpenSpot sampling cards (IonSense Inc.) using $CHCl_3$ or CH_2Cl_2 as the solvent. Ionization was accomplished using UHP He plasma with no additional ionization agents. The mass calibration was carried out using Pierce LTQ Velos ESI (+) and (–) Ion calibration solutions (Thermo Fischer Scientific).

Biaryl iodides **6**[1] and **52**,[1] silyl nonaflate **7**,[2] ketone **30**,[3] indole **44**,[1] azaindole **49**,[4] iodotosylate **50**,[5] and Pd(COD)DQ[6] are known compounds and were prepared according to literature procedures. The ^{1}H NMR spectral data matched those reported in the literature.

Part I. Experimental Procedures

A. Synthesis of Biaryl Halides

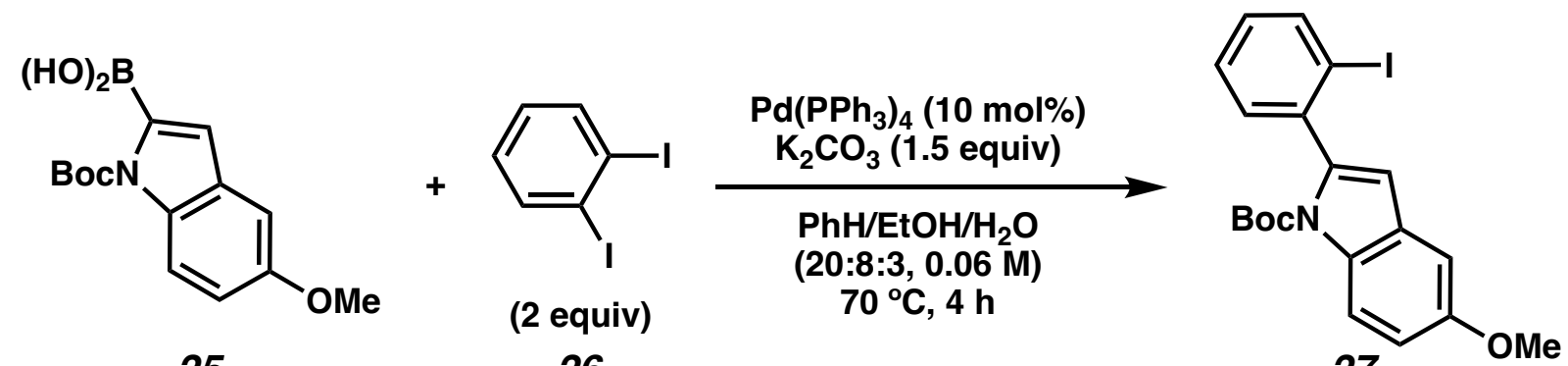

25 *26* *27*

Biaryl 27. A flask was charged with potassium carbonate (128 mg, 0.93 mmol, 1.5 equiv), **25** (180 mg, 0.62 mmol, 1.0 equiv), and **26** (408 mg, 1.24 mmol, 2.0 equiv) and placed under vacuum and backfilled with nitrogen three times. The flask was then charged with $Pd(PPh_3)_4$ (72 mg, 0.062 mmol, 10 mol%) then placed under vacuum and backfilled with nitrogen. The flask was then charged with benzene (6.2 mL, 0.1 M), sparged ethanol (2.5 mL, 0.25 M), and sparged DI water (0.92 mL, 0.67 M). The flask was fitted with an air condenser, then stirred at 70 °C for 4 h. After 4 h, the reaction was cooled to 23 °C and quenched with DI water (10 mL). The resulting mixture was extracted with diethyl ether (3 x 20 mL). The combined organic layers were dried with Na_2SO_4, filtered, and concentrated under reduced pressure. The crude residue was purified by automated flash column chromatography (SiO_2, 0 → 10% EtOAc in hexanes) to provide biaryl **27** (178 mg, 0.40 mmol, 64% yield) as a clear colorless oil. **Biaryl 27**: R_f 0.54 (9:1 hexanes:EtOAc); ^{1}H NMR (500 MHz, $CDCl_3$): δ 8.22 (d, J = 9.1 Hz, 1H), 7.88 (d, J = 8.1 Hz, 1H), 7.39 (t, J = 7.3 Hz, 1H), 7.35 (d, J = 6.9 Hz, 1H), 7.08 (t, J = 7.8 Hz, 1H), 7.07 – 7.03 (m, 1H), 6.98 (dd, J = 9.1, 2.2 Hz, 1H), 6.42 (s, 1H), 3.87 (s, 3H), 1.28 (s, 9H); ^{13}C NMR (125 MHz, $CDCl_3$): δ 156.1, 149.7, 141.5, 141.3, 138.5, 131.3, 130.6, 129.8, 129.5, 127.7, 116.7, 113.6, 110.1, 103.1, 100.9, 83.1, 55.8, 27.7; IR (film): 2979, 1726, 1482, 1454, 1218 cm^{-1}; HRMS-APCI (*m/z*) $[M + H]^+$ calcd for $C_{20}H_{21}INO_3^+$, 450.0561; found 450.0498.

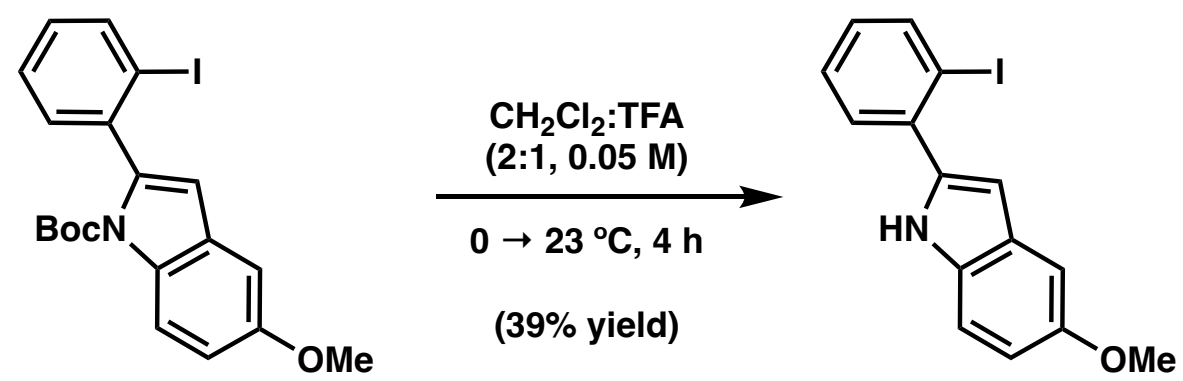

27 *28*

Biaryl 28. To a solution of **27** (151 mg, 0.35 mmol, 1.0 equiv) at 0 °C in CH_2Cl_2 (4.6 mL, 0.075 M) was added TFA (2.3 mL, 0.15 M). The solution was allowed to warm to 23 °C and stirred for 4 h. The reaction was then quenched with 1.0 M aqueous NaOH (30 mL) until the pH was >10. The aqueous layer was then extracted with CH_2Cl_2 (3 x 10 mL). The combined organic layers were washed with brine (20 mL), dried with Na_2SO_4, filtered, and concentrated under reduced pressure. The crude residue was purified by automated flash column chromatography (0 → 10% EtOAc in hexanes) to provide biaryl **28** (SiO_2, 47 mg, 0.14 mmol, 39% yield) as a clear colorless oil. **Biaryl 28**: R_f 0.32 (9:1 hexanes:EtOAc); ^{1}H NMR (500 MHz, $CDCl_3$): δ 8.25 (s, 1H), 7.99 (d, J = 7.8 Hz, 1H), 7.48 (d, J = 7.4 Hz, 1H), 7.41 (t, J = 7.4 Hz, 1H), 7.31 (d, J = 8.7 Hz, 1H), 7.16 – 7.11 (m, 1H), 7.06 (t, J = 7.6 Hz, 1H), 6.94 – 6.88 (m, 1H), 6.69 (s, 1H), 3.88 (s, 3H); ^{13}C NMR (125 MHz, $CDCl_3$): δ 154.5, 140.4, 139.5, 138.0, 131.3, 131.2, 129.6, 128.7, 128.4, 113.1, 111.9, 103.4, 102.4, 97.1, 56.0; IR (film): 3403, 2937, 1448, 1212, 1146 cm^{-1}; HRMS-APCI (*m/z*) $[M + H]^+$ calcd for $C_{15}H_{13}INO^+$, 350.0036; found 350.0020.

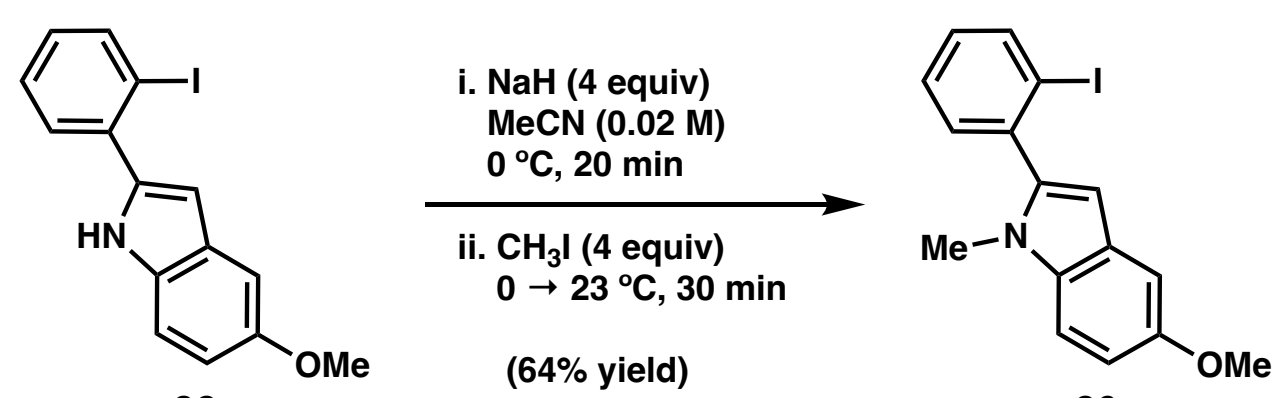

28 *29*

Biaryl 29. To a solution of **28** (92 mg, 0.26 mmol, 1.0 equiv) in MeCN (13 mL, 0.02 M) was added NaH (60% in mineral oil, 42.0 mg, 1.05 mmol, 4.0 equiv) in one portion at 23 °C. The solution was stirred at 0 °C for 20 min. After 20 min, CH_3I (0.066 mL, 1.05 mmol, 4.0 equiv) was added in one portion, and the solution was warmed to 23 °C over 30 min. After 30 min, the reaction was quenched with DI water (10 mL). The resulting mixture was extracted with ethyl acetate (3 x 20 mL). The combined organic layers were washed with brine (2 x 50 mL), dried with Na_2SO_4, filtered, and concentrated under reduced pressure. The crude residue was purified by automated flash column chromatography (SiO_2, 0 → 20% EtOAc in hexanes) to provide biaryl **29** (79 mg, 0.17 mmol, 64% yield) as a white amorphous solid. **Biaryl 29**: R_f 0.42 (9:1 hexanes:EtOAc);

^{1}H NMR (600 MHz, $CDCl_3$): δ 7.98 (d, *J* = 7.9, 1.2 Hz, 1H), 7.45 – 7.41 (m, 1H), 7.39 – 7.38 (m, 1H), 7.29 – 7.26 (overlapped with residual solvent peak, 1H), 7.16 – 7.12 (m, 2H), 6.94 (ddd, *J* = 8.8, 2.5, 1.1 Hz, 1H), 6.41 (s, 1H), 3.89 (s, 3H), 3.52 (s, 3H); ^{13}C NMR (150 MHz, $CDCl_3$): δ 154.5, 143.1, 139.1, 138.7, 132.6, 132.0, 130.2, 128.0, 127.9, 112.3, 110.4, 102.5, 101.6, 101.5, 56.1, 31.0; IR (film): 2935, 2831, 1623, 1578, 1557 cm^{-1}; HRMS-APCI (*m/z*) $[M + H]^+$ calcd for $C_{16}H_{15}INO^+$, 364.0193; found 364.0179.

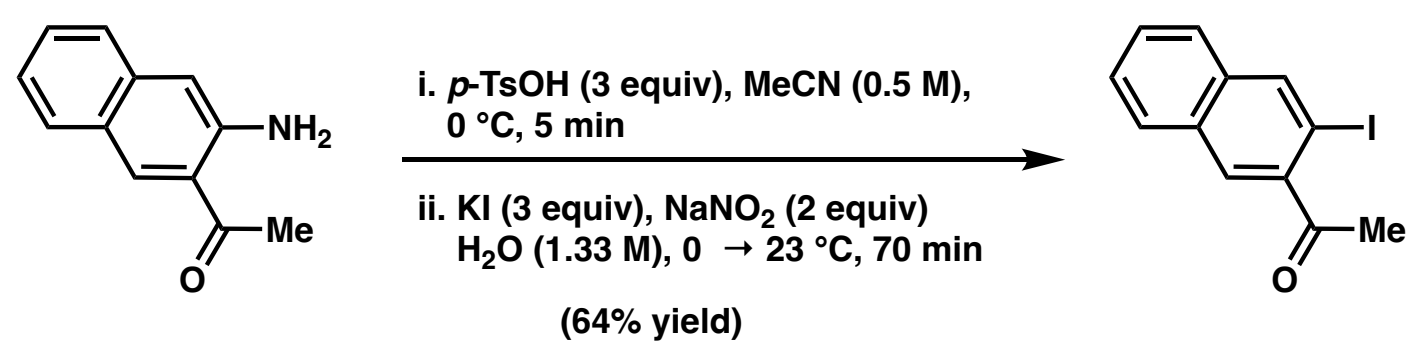

30 31

Ketone 31. To a solution of **30** (492 mg, 2.66 mmol, 1.0 equiv) and *p*-TsOH (1.52 g, 7.97 mmol, 3.0 equiv) in MeCN (5 mL, 0.5 M) at 0 °C was added $NaNO_2$ (366 mg, 5.31 mmol, 2.0 equiv) in DI water (1 mL, 2.66 M) over 1 minute. The red solution was stirred for 5 min, then KI (1.10 g, 6.64 mmol, 2.5 equiv) in DI water (1 mL, 2.66 M) was added. The mixture was stirred for 10 min, then warmed to 23 °C and stirred for an additional 1 h. After this time, the reaction was quenched by the addition of saturated aqueous $NaHCO_3$ (30 mL) and saturated aqueous $Na_2S_2O_3$ (20 mL). The resulting mixture was extracted with diethyl ether (3 x 50 mL). The combined organic layers were dried with Na_2SO_4, filtered, and concentrated under reduced pressure. The crude residue was purified by automated flash column chromatography (SiO_2, 0 → 20% EtOAc in hexanes) to provide ketone **31** (503 mg, 1.70 mmol, 64% yield) as a red amorphous solid. **Ketone 31**: R_f 0.35 (9:1 hexanes:EtOAc); ^{1}H NMR (600 MHz, $CDCl_3$) δ 8.47 (s, 1H), 8.01 (s, 1H), 7.86 (d, *J* = 7.8 Hz, 1H), 7.75 (d, *J* = 7.9 Hz, 1H), 7.60 – 7.55 (m, 2H), 2.73 (s, 3H); ^{13}C NMR (150 MHz, $CDCl_3$) δ 201.2, 140.7, 140.1, 135.5, 131.9, 128.9, 128.7, 128.7, 127.6, 126.8, 86.9, 29.7; IR (film): 3056, 1690, 1577, 1266, 749 cm^{-1}; HRMS-APCI (*m/z*) $[M + H]^+$ calcd for $C_{12}H_{10}IO^+$, 296.97708; found 296.97590.

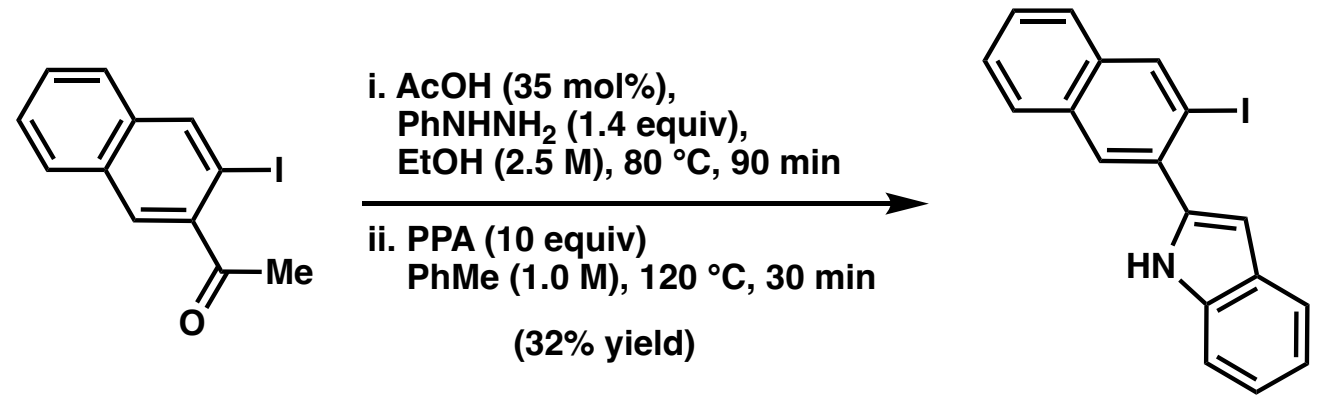

31 ***32***

Indole 32. To a solution of **31** (200 mg, 0.675 mmol, 1.0 equiv) in EtOH (0.27 mL, 2.5 M) was added $PhNHNH_2$ (102 mg, 0.946 mmol, 1.4 equiv) followed by AcOH (0.014 mL, 0.236 mmol, 35 mol%). The solution was heated to 80 °C and stirred for 90 min, then cooled to 23 °C and concentrated under reduced pressure to give a red oil. The oil was redissolved in PhMe (0.68 mL, 1.0 M) and PPA (1.20 g, 6.76 mmol, 10 equiv) was added. The suspension was then heated to 120 °C and stirred for 30 min. After this time, the reaction was cooled to 23 °C and quenched by the addition of water (10 mL). The resulting mixture was extracted with diethyl ether (3 x 10 mL). The combined organic layers were washed with brine (20 mL), dried with Na_2SO_4, filtered, and concentrated under reduced pressure. The crude residue was purified by automated flash column chromatography (SiO_2, 0 → 15% EtOAc in hexanes) to provide indole **32** (81 mg, 0.216 mmol, 32% yield) as a tan foam. **Indole 32**: R_f 0.21 (9:1 hexanes:EtOAc); ^{1}H NMR (500 MHz, $CDCl_3$) δ 8.53 (s, 1H), 8.36 (br s, 1H), 7.97 (d, *J* = 3.0 Hz, 1H), 7.84 – 7.80 (m, 1H), 7.79 – 7.74 (m, 1H), 7.71 (d, *J* = 7.9 Hz, 1H), 7.56 – 7.51 (m, 2H), 7.46 (d, *J* = 8.0 Hz, 1H), 7.28 – 7.23 (overlapped with residual solvent, 1H), 7.18 (t, *J* = 7.5 Hz, 1H), 6.82 (d, *J* = 2.1 Hz, 1H); ^{13}C NMR (125 MHz, $CDCl_3$) δ 139.7, 138.9, 136.3, 134.6, 134.4, 132.6, 130.1, 128.5, 128.1, 127.4, 127.3, 126.7, 122.7, 121.0, 120.4, 111.1, 104.1, 94.5; IR (film): 3406, 3053, 1578, 1306, 748 cm^{-1}; HRMS-APCI (*m/z*) $[M + H]^+$ calcd for $C_{18}H_{13}IN^+$, 370.00872; found 370.00752.

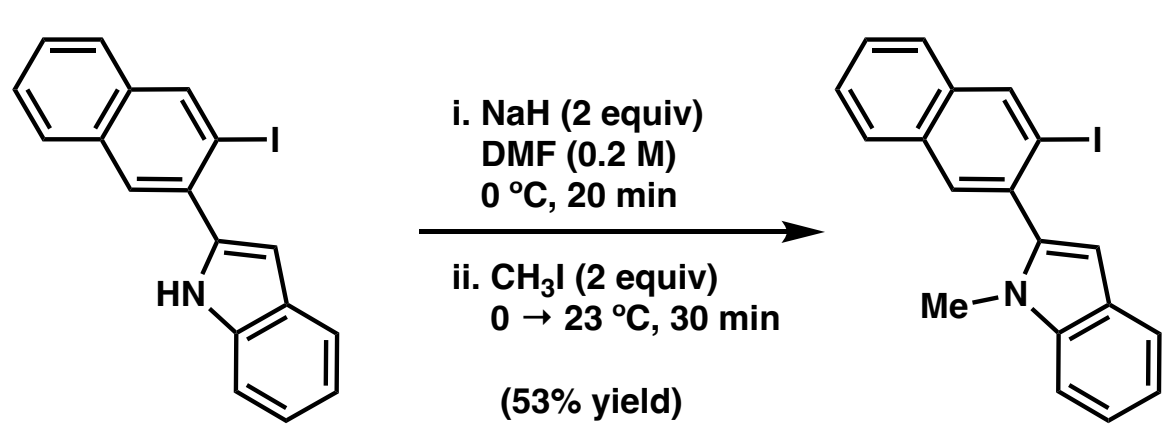

32 ***33***

Biaryl 33. To a solution of **32** (80 mg, 0.22 mmol, 1.0 equiv) in DMF (1.1 mL, 0.2 M) was added NaH (60% in mineral oil, 17 mg, 0.44 mmol, 2.0 equiv). The solution was stirred at 0 °C for 20 min. After 20 min, CH_3I (0.027 mL, 0.43 mmol, 2.0 equiv) was added, and the solution was warmed to 23 °C. After 30 min at 23 °C, the reaction was quenched with DI water (10 mL). The

resulting mixture was extracted with diethyl ether (20 mL). The organic layer was washed with brine (3 x 10 mL), dried with Na_2SO_4, filtered, and concentrated under reduced pressure. The crude residue was purified by automated flash column chromatography (SiO_2, 0 → 5% EtOAc in hexanes) to provide biaryl **33** (44 mg, 0.12 mmol, 53% yield) as a white amorphous solid. **Biaryl 33**: R_f 0.65 (9:1 hexanes:EtOAc); ^{1}H NMR (600 MHz, $CDCl_3$) δ 8.53 (s, 1H), 7.92 (s, 1H), 7.86 – 7.82 (m, 2H), 7.73 (d, *J* = 7.8 Hz, 1H), 7.60 – 7.57 (m, 2H), 7.42 (d, *J* = 8.2 Hz, 1H), 7.34 – 7.31 (m, 1H), 7.22 (t, *J* = 7.4 Hz, 1H), 6.59 (s, 1H), 3.59 (s, 3H); ^{13}C NMR (150 MHz, $CDCl_3$) δ 142.4, 138.3, 137.3, 135.1, 134.7, 132.4, 131.1, 128.2, 127.7, 127.6, 127.3, 126.7, 122.0, 120.9, 120.0, 109.7, 102.5, 98.1, 31.0; IR (film): 3053, 2936, 1453, 903, 733 cm^{-1}; HRMS-APCI (*m/z*) $[M + H]^+$ calcd for $C_{19}H_{15}IN^+$, 384.02437; found 384.02304.

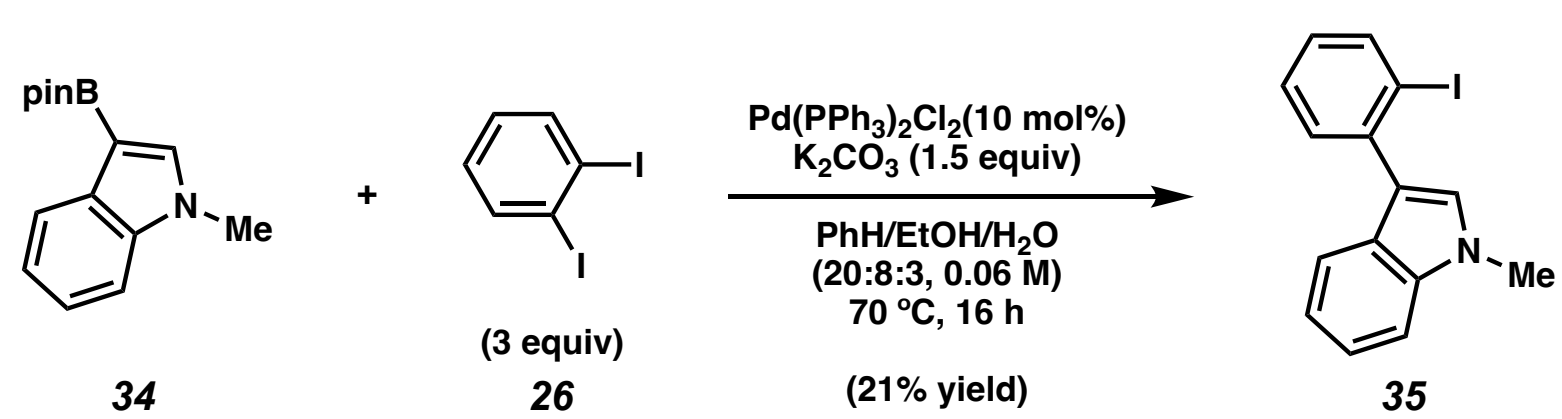

Biaryl 35: A flask was charged with potassium carbonate (161 mg, 1.17 mmol, 1.5 equiv), **34** (200 mg, 0.78 mmol, 1.0 equiv), and **26** (770 mg, 2.33 mmol, 3.0 equiv) and placed under vacuum and backfilled with nitrogen three times. The flask was then charged with $Pd(PPh_3)_2Cl_2$ (55 mg, 0.078 mmol, 10 mol%) then placed under vacuum and backfilled with nitrogen. The flask was then charged with benzene (7.8 mL, 0.1 M), sparged ethanol (3.11 mL, 0.25 M), and sparged DI water (1.16 mL, 0.67 M). The flask was fitted with an air condenser, then heated to 70 °C for 16 h. After this time, the reaction was cooled to 23 °C and quenched with DI water (30 mL). The resulting mixture was extracted with diethyl ether (3 x 30 mL). The combined organic layers were dried with Na_2SO_4, filtered, and concentrated under reduced pressure. The crude residue was purified by automated flash column chromatography (SiO_2, 0 → 15% EtOAc in hexanes) to provide biaryl **35** (54 mg, 0.16 mmol, 21% yield) as a white amorphous solid. Spectral data match those previously reported.[7] **Biaryl 35**: R_f 0.5 (9:1 hexanes:EtOAc); ^{1}H NMR (500 MHz, $CDCl_3$) δ 8.00 (d, *J* = 7.9 Hz, 1H), 7.54 (d, *J* = 8.0 Hz, 1H), 7.46 (dd, *J* = 7.5, 1.7 Hz, 1H), 7.39 (t, *J* = 8.2 Hz, 2H), 7.29 (overlapped with residual solvent, 2H), 7.15 (t, *J* = 7.5 Hz, 1H), 7.01 (t, *J* = 7.4 Hz, 1H), 3.88 (s, 3H); IR (film): 1547, 1478, 1012, 741 cm^{-1}; HRMS-APCI (m/z) $[M + H]^+$ calcd for $C_{15}H_{13}IN^+$, 334.0087 ; found 334.0070.

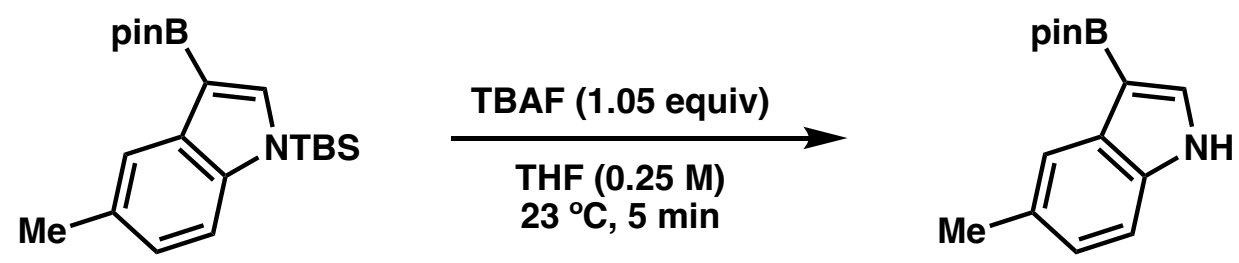

36 *37*

Indole 37. To a stirred solution of **36** (703 mg, 1.89 mmol, 1.0 equiv) in THF (7.57 mL, 0.25 M) at 23 °C was added TBAF (1.99 mL, 1.0 M in THF, 1.99 mmol, 1.05 equiv) dropwise over 5 min. The solution was stirred for an additional 5 min. After 5 min, the reaction was quenched with DI water (10 mL). The resulting mixture was extracted with diethyl ether (3 x 25 mL). The combined organic layers were washed with brine (2 x 40 mL), dried with Na_2SO_4, filtered, and concentrated under reduced pressure. The crude residue was purified by recrystallization from heptane to provide indole **37** (431 mg, 1.68 mmol, 89% yield) as a grey crystalline solid. Spectral data match those previously reported.[8] **Indole 37**: MP = 112 °C; ^{1}H NMR (600 MHz, $CDCl_3$): δ 8.34 (br s, 1H), 7.82 (s, 1H), 7.62 (d, *J* = 2.2 Hz, 1H), 7.28 (d, *J* = 8.3 Hz, 1H), 7.02 (d, *J* = 8.3, 1H), 2.48 (s, 3H), 1.37 (s, 12H).

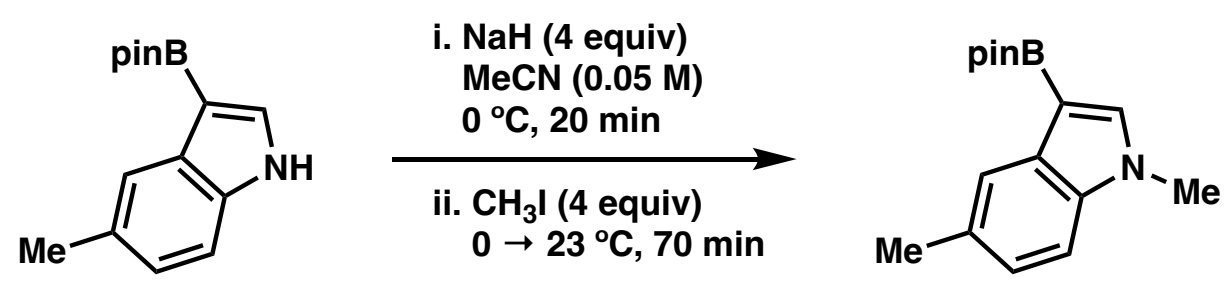

37 *38*

Indole 38. To a solution of **37** (111 mg, 0.43 mmol, 1.0 equiv) in MeCN (17.6 mL, 0.05 M) was added NaH (60% in mineral oil, 69 mg, 1.72 mmol, 4.0 equiv) at 23°C in a single portion. The solution was cooled and stirred at 0 °C for 20 min. After 20 min, CH_3I (0.11 mL, 1.72 mmol, 4.0 equiv) was added, and the solution was warmed to 23 °C over 70 min. After 70 min, the reaction was quenched with DI water (10 mL). To the resulting mixture was added 1.0 M aqueous HCl (1 mL) until the pH reached ~6. The mixture was then extracted with ethyl acetate (3 x 15 mL). The combined organic layers were washed with brine (2 x 25 mL), dried with Na_2SO_4, filtered, and concentrated under reduced pressure to provide indole **38** (116 mg, 0.43 mmol, 99% yield) as a clear yellow oil. Spectral data match those previously reported.[9] **Indole 38**: (500 MHz, $CDCl_3$): δ 7.80 (dt, *J* = 1.7, 0.8 Hz, 1H), 7.48 (s, 1H), 7.20 (d, *J* = 8.3 Hz, 1H), 7.05 (dd, *J* = 8.4, 1.7 Hz, 1H), 3.77 (s, 3H), 2.48 (s, 3H), 1.36 (s, 12H).

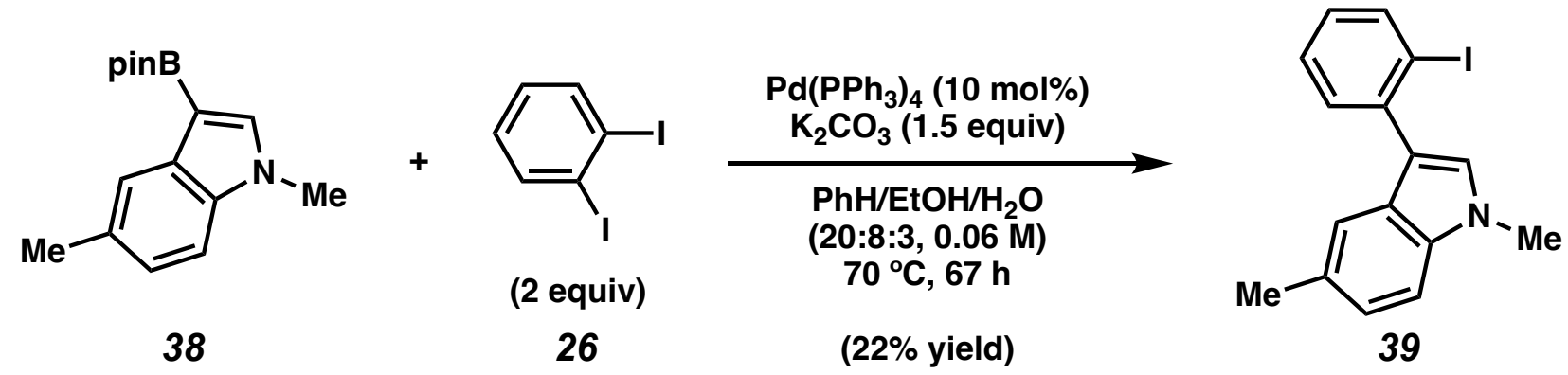

Biaryl 39. A flask was charged with potassium carbonate (64 mg, 0.46 mmol, 1.5 equiv), **38** (83 mg, 0.31 mmol, 1.0 equiv), and **26** (203 mg, 0.62 mmol, 2.0 equiv) and placed under vacuum and backfilled with nitrogen three times. The flask was then charged with $Pd(PPh_3)_4$ (36 mg, 0.031 mmol, 10 mol%) then placed under vacuum and backfilled with nitrogen three times. The flask was then charged with benzene (3.1 mL, 0.1 M), sparged ethanol (1.2 mL, 0.25 M), and sparged DI water (0.46 mL, 0.67 M). The flask was fitted with an air condenser, then stirred at 70 °C for 67 h. After 67 h, the reaction was cooled to 23 °C and quenched with DI water (5 mL). The resulting mixture was extracted with diethyl ether (3 x 10 mL). The combined organic layers were washed with brine (20 mL), dried with Na_2SO_4, filtered, and concentrated under reduced pressure. The crude residue was purified by automated flash column chromatography (SiO_2, 0 → 25% EtOAc in hexanes), then preparative thin-layer chromatography (SiO_2, 9:1 hexanes:benzene) to provide biaryl **39** (24 mg, 0.068 mmol, 22% yield) as a clear colorless oil. **Biaryl 39**: R_f 0.29 (9:1 hexanes:benzene); ^{1}H NMR (600 MHz, $CDCl_3$): δ 8.05 (d, J = 7.9 Hz, 1H), 7.52 (d, J = 7.6, 1.7 Hz, 1H), 7.45 (t, J = 7.5 Hz, 1H), 7.37 (s, 1H), 7.33 – 7.30 (m, 1H), 7.28 – 7.26 (overlapped with residual solvent, 1H), 7.15 (d, J = 8.5 Hz, 1H), 7.06 (tt, J = 7.5, 1.7 Hz, 1H), 3.89 (s, 3H), 2.50 (s, 3H); ^{13}C NMR (150 MHz, $CDCl_3$): δ 140.3, 139.9, 135.1, 131.5, 129.1, 128.5, 128.11, 128.09, 127.3, 123.7, 119.8, 118.0, 109.3, 100.9, 33.1, 21.6; IR (film): 2917, 1584, 1547, 1490, 1467 cm^{-1}; HRMS-APCI (*m/z*) $[M + H]^+$ calcd for $C_{16}H_{15}IN^+$, 348.0277; found 348.0229.

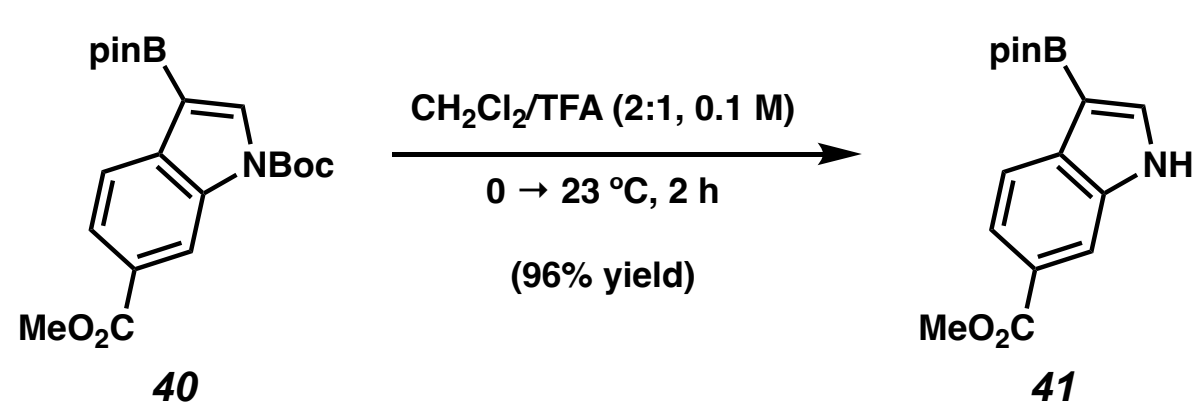

Indole 41. To a solution of **40** (473 mg, 1.18 mmol, 1.0 equiv) at 0 °C in CH_2Cl_2 (7.9 mL, 0.15 M) was added TFA (3.9 mL, 0.3 M) in a single portion. The solution was allowed to warm to 23 °C and stirred for 2 h. The reaction was then quenched with 1.0 M aqueous NaOH (20 mL) until the pH reached >10. The aqueous layer was then extracted with CH_2Cl_2 (3 x 20 mL). The combined

organic layers were dried with Na_2SO_4, filtered, and concentrated under reduced pressure to provide indole **41** (343 mg, 1.14 mmol, 96% yield) as a tan amorphous solid. Spectral data match those previously reported.[10] **Indole 41**: ^{1}H NMR (500 MHz, $CDCl_3$): δ 8.86 (s, 1H), 8.21 (s, 1H), 8.02 (s, 2H), 3.98 (s, 3H), 1.39 (s, 12H).

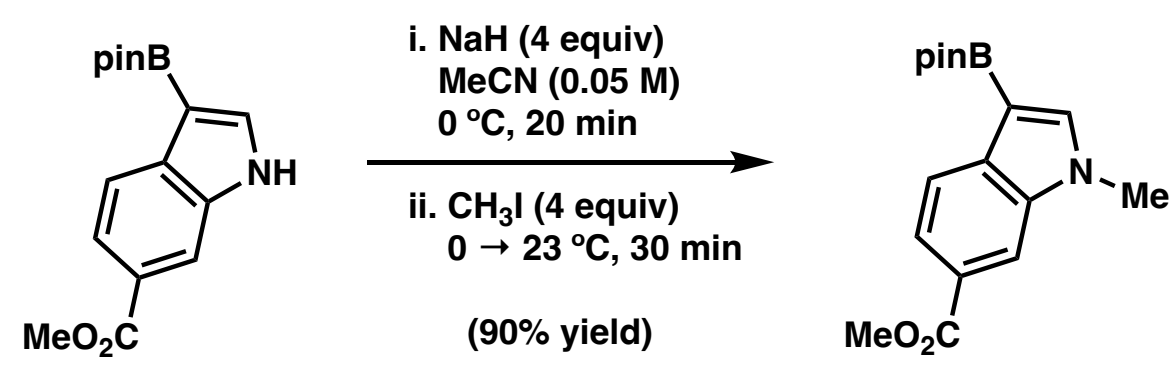

Indole 42. To a solution of **41** (730 mg, 2.42 mmol, 1.0 equiv) in MeCN (49 mL, 0.05 M) was added NaH (60% in mineral oil, 398 mg, 9.70 mmol, 4.0 equiv) in a single portion at 0 °C. The solution was stirred at 0 °C for 20 min. After 20 min, CH_3I (0.61 mL, 9.70 mmol, 4.0 equiv) was added in a single portion, and the solution was warmed to 23 °C over 30 min. After 30 min, the reaction was quenched with DI water (5 mL). To the resulting mixture was added 1.0 M aqueous HCl until the pH was ~6. The mixture was then extracted with ethyl acetate (3 x 25 mL). The combined organic layers were washed with brine (2 x 50 mL), dried with Na_2SO_4, filtered, and concentrated under reduced pressure. The crude residue was purified on a 3 cm silica gel plug in a 5 mL syringe eluting with 10 mL CH_2Cl_2 then 20 mL diethyl ether to provide indole **42** (688 mg, 2.18 mmol, 90% yield) as a clear yellow oil. Spectral data match those previously reported.[11] **Indole 42**: (500 MHz, $CDCl_3$): δ 8.09 (s, 1H), 8.02 (d, J = 8.3 Hz, 1H), 7.86 (d, J = 8.3 Hz, 1H), 7.65 (s, 1H), 3.95 (s, 3H), 3.85 (s, 3H), 1.36 (s, 12H).

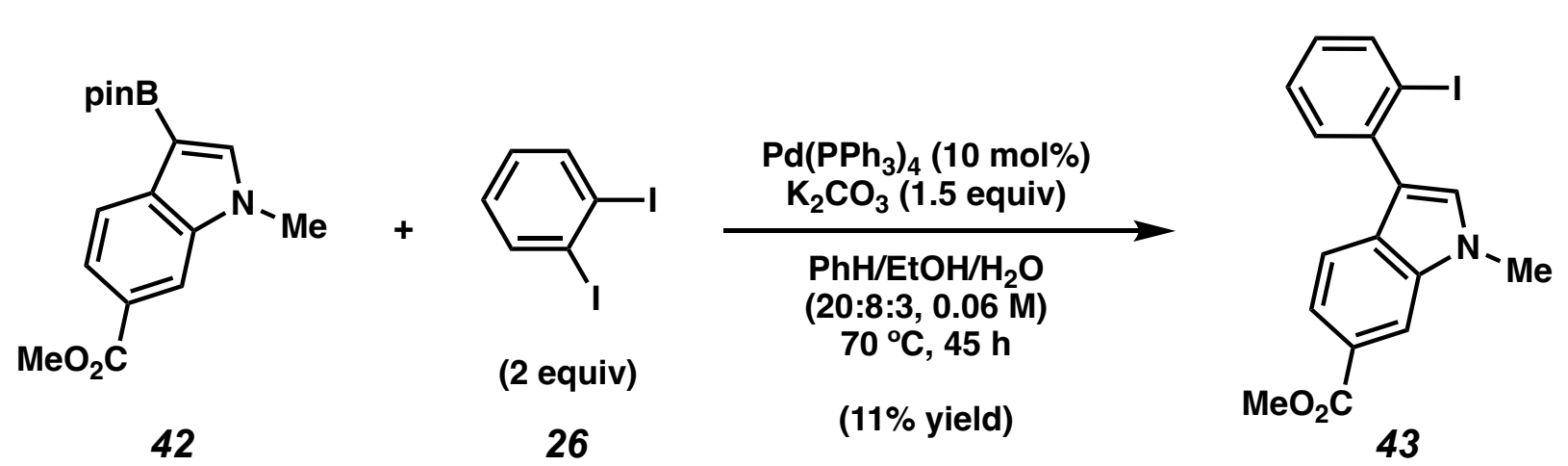

Biaryl 43. A flask was charged with potassium carbonate (452 mg, 3.27 mmol, 1.5 equiv), **42** (688 mg, 2.18 mmol, 1.0 equiv), and **26** (1.44 g, 4.37 mmol, 2.0 equiv) and placed under vacuum and backfilled with nitrogen three times. The flask was then charged with $Pd(PPh_3)_4$ (252 mg, 0.218 mmol, 10 mol%) then placed under vacuum and backfilled with nitrogen. The flask was then charged with benzene (21.8 mL, 0.1 M), sparged ethanol (8.73 mL, 0.25 M), and sparged DI water

(3.26 mL, 0.67 M). The flask was fitted with an air condenser, then heated to 70 °C for 45 h. After 45 h, the reaction was cooled to 23 °C and quenched with DI water (25 mL). The resulting mixture was extracted with diethyl ether (3 x 20 mL). The combined organic layers were washed with brine (50 mL), dried with Na_2SO_4, filtered, and concentrated under reduced pressure. The crude residue was purified by automated flash column chromatography (SiO_2, 0 → 90% EtOAc in hexanes), then preparative thin-layer chromatography (SiO_2, 18:1:1 benzene:CH_2Cl_2:diethyl ether) to provide biaryl **43** (90 mg, 0.23 mmol, 11% yield) as a clear colorless oil. **Biaryl 43**: R_f 0.11 (9:1 hexanes:ethyl acetate); ^{1}H NMR (500 MHz, $CDCl_3$): δ 8.16 (s, 1H), 8.00 (d, J = 8.0 Hz, 1H), 7.83 (d, J = 8.4 Hz, 1H), 7.53. (d, J = 8.4 Hz, 1H), 7.44 – 7.39 (m, 3H), 7.06 – 7.00 (m, 1H), 3.96 (s, 3H), 3.94 (s, 3H); ^{13}C NMR (150 MHz, $CDCl_3$): δ 168.2, 140.0, 139.5, 136.1, 131.55, 131.54, 130.5, 128.6, 128.2, 123.7, 120.8, 120.0, 119.1, 112.2, 100.7, 52.1, 33.4; IR (film): 2924, 2853, 1716, 1463, 1265 cm^{-1}; HRMS-APCI (*m/z*) [M]$^+$ calcd for $C_{17}H_{14}INO_2^+$, 391.0064; found 391.0055.

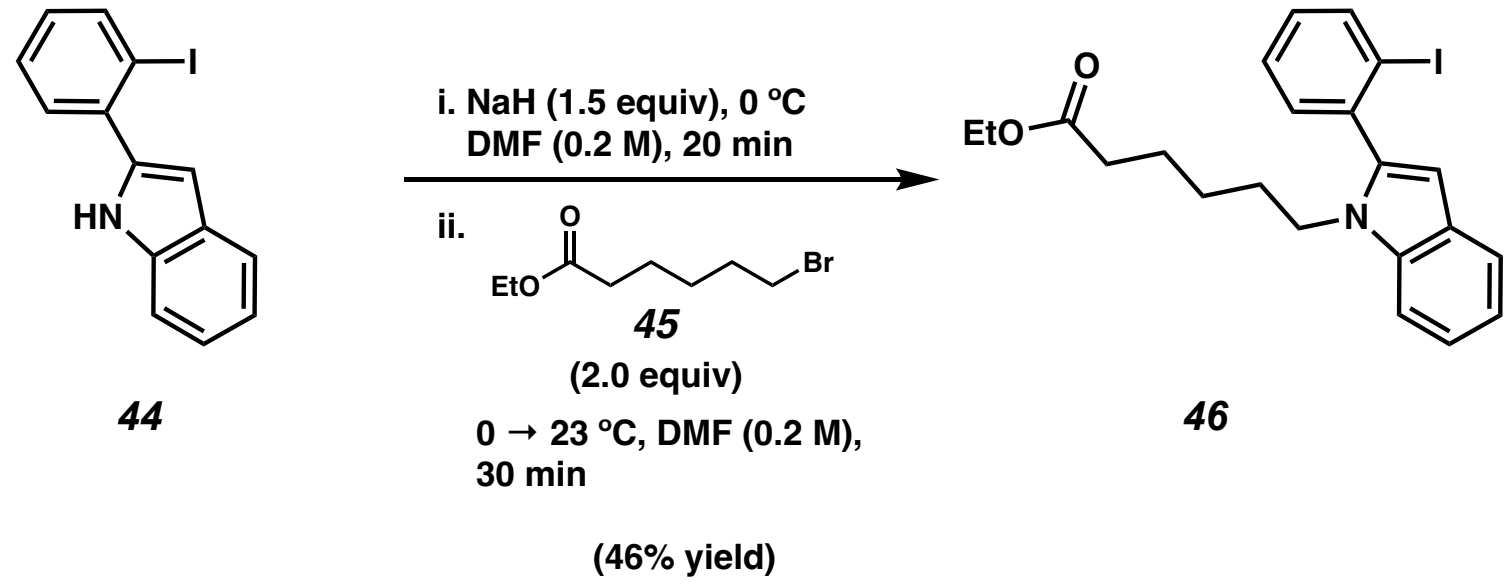

Biaryl 46. To a solution of **44** (556 mg, 1.74 mmol, 1.0 equiv) in DMF (7.0 mL, 0.2 M) was added NaH (60% in mineral oil, 105 mg, 2.61 mmol, 1.5 equiv) at 0 °C in a single portion. The solution was stirred at 0 °C for 20 min. After 20 min, **45** (0.62 mL, 3.48 mmol, 2.0 equiv) was added, and the solution was warmed to 23 °C. After 30 min at 23 °C, the reaction was quenched with DI water (10 mL). The resulting mixture was extracted with diethyl ether (20 mL). The organic layer was washed with brine (3 x 10 mL), dried with Na_2SO_4, filtered, and concentrated under reduced pressure. The crude residue was purified by automated flash column chromatography (SiO_2, 0 → 15% EtOAc in hexanes) to provide biaryl **46** (366 mg, 0.79 mmol, 46% yield) as a yellow oil. **Biaryl 46**: R_f 0.46 (9:1 hexanes:EtOAc); ^{1}H NMR (500 MHz, $CDCl_3$) δ 7.98 (dd, J = 8.0, 1.1 Hz, 1H), 7.66 (dt, J = 7.8, 1.1 Hz, 1H), 7.44 (t, J = 7.4, Hz, 1H), 7.40 – 7.36 (m, 2H), 7.26 – 7.22 (overlapped with residual solvent, 1H), 7.16 – 7.11 (m, 2H), 6.46 (d, J = 0.9 Hz, 1H), 4.10 – 4.06 (m, 3H), 3.85 (m, 1H), 2.16 (t, J = 7.5 Hz, 2H), 1.69 – 1.56 (overlapped with residual water, 2H),

1.50 – 1.42 (m, 2H), 1.21 (t, J = 7.2 Hz, 3H), 1.18 – 1.11 (m, 2H); ^{13}C NMR (150 MHz, $CDCl_3$) δ 173.6, 142.0, 139.3, 138.7, 136.4, 132.1, 130.2, 128.0, 127.9, 121.8, 121.0, 119.8, 110.0, 102.6, 101.7, 60.3, 44.1, 34.2, 29.8, 26.5, 24.5, 14.4; IR (film): 1732, 1460, 1180, 751 cm^{-1}; HRMS-APCI (m/z) $[M + H]^+$ calcd for $C_{22}H_{25}INO_2^+$, 462.0925; found 462.0904.

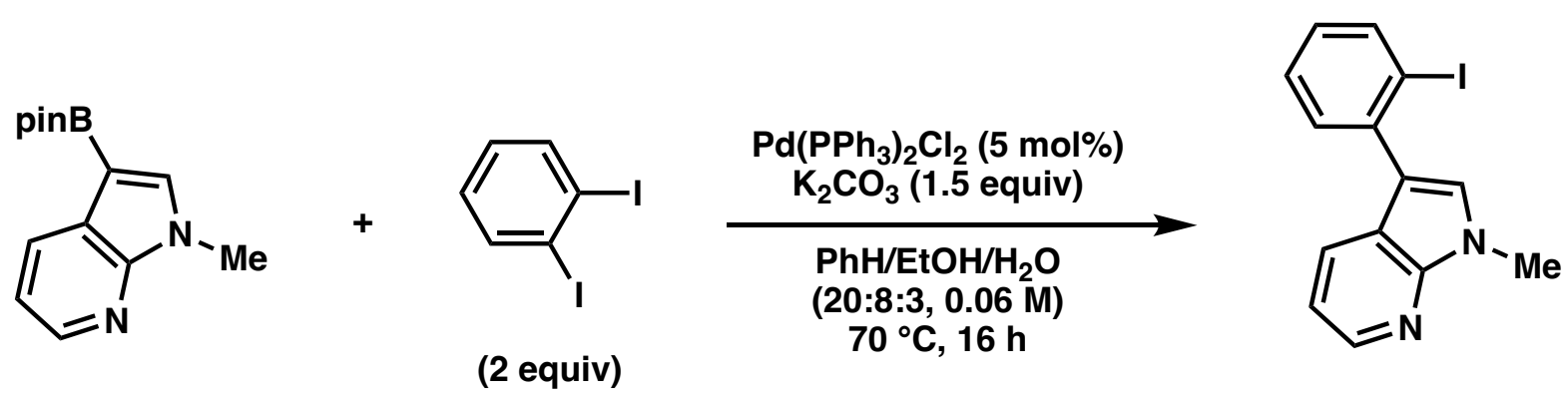

47 ***26*** ***48***

Biaryl 48. A flask was charged with potassium carbonate (482 mg, 3.49 mmol, 1.5 equiv), **47** (600 mg, 2.32 mmol, 1.0 equiv), and **26** (2.30 g, 6.96 mmol, 2.0 equiv) and placed under vacuum and backfilled with nitrogen three times. The flask was then charged with $Pd(PPh_3)_2Cl_2$ (82 mg, 0.116 mmol, 5 mol %) then placed under vacuum and backfilled with nitrogen three times. The flask was then charged with benzene (23.2 mL, 0.1 M), sparged ethanol (9.3 mL, 0.25 M), and sparged DI water (3.5 mL, 0.67 M). The flask was fitted with an air condenser, then stirred at 70 °C for 16 hours. After this time, the reaction was cooled to 23 °C and quenched with DI water (30 mL). The resulting mixture was extracted with diethyl ether (3 x 30 mL). The combined organic layers were dried with Na_2SO_4, filtered, and concentrated under reduced pressure. The crude residue was purified by automated flash column chromatography (SiO_2, 0 → 15% EtOAc in hexanes) to provide biaryl **48** (167 mg, 0.51 mmol, 22% yield) as a yellow powder. **Biaryl 48**: R_f 0.12 (9:1 hexanes:EtOAc); ^{1}H NMR (600 MHz, $CDCl_3$) δ 8.39 (d, J = 4.7 Hz, 1H), 8.01 (d, J = 8.0 Hz, 1H), 7.85 (d, J = 7.8 Hz, 1H), 7.41 (s, 1H), 7.40 (s, 1H), 7.37 (s, 1H), 7.10 (dd, J = 7.9, 4.7 Hz, 1H), 7.06 – 7.01 (m, 1H), 3.98 (s, 3H); ^{13}C NMR (150 MHz, $CDCl_3$) δ 147.6, 143.3, 140.0, 139.5, 131.5, 128.61, 128.55, 128.26, 128.23, 119.4, 117.0, 115.9, 100.3, 31.5; IR (film): 3050, 2937, 1537, 1447, 1298 cm^{-1}; HRMS-APCI (*m/z*) $[M + H]^+$ calcd for $C_{14}H_{12}IN_2^+$, 335.00397; found 335.00299.

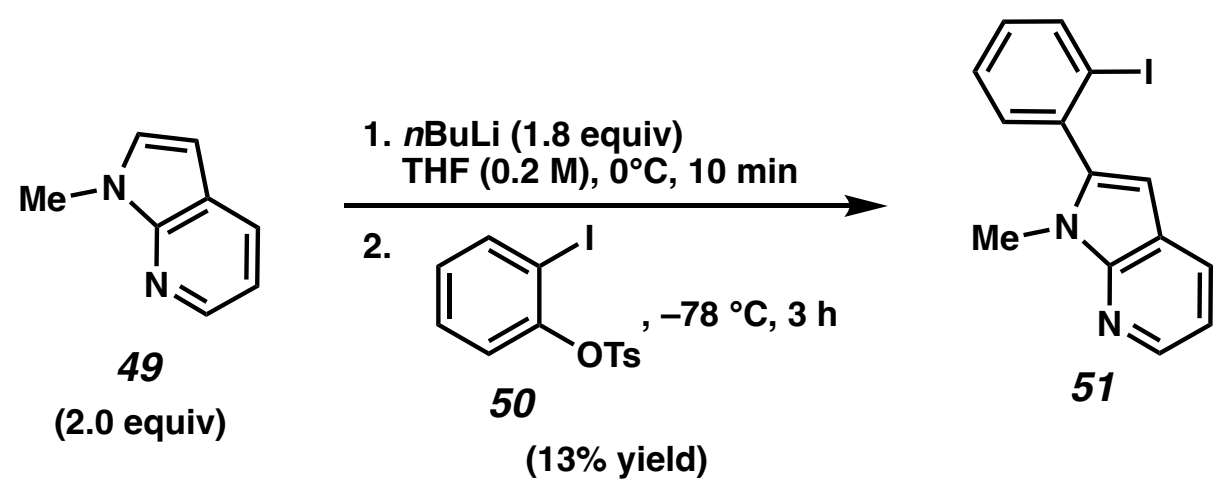

Biaryl 51. Compound **51** was prepared by a modification of the method developed by Hamura and coworkers.[12] To a stirred solution of **49** (910 mg, 6.89 mmol, 2.0 equiv) in THF (25 mL, 0.2 M) at 0 °C was added *n*-BuLi (2.77 mL, 2.24 M, 1.8 equiv) dropwise over 5 minutes. The solution was stirred for 10 mins before being cooled to –78 °C. Compound **50** (1.29 g, 3.44 mmol, 1.0 equiv) was then added as a solution in THF (25 mL, 0.2 M) over 5 minutes. The solution was then stirred for 3 hours at –78 °C. After this time, the reaction was quenched by the addition of DI water (20 mL) and warmed to 23 °C. The resulting mixture was extracted with diethyl ether (3 x 20 mL). The combined organic layers were dried with Na_2SO_4, filtered, and concentrated under reduced pressure. The crude residue was purified by automated flash column chromatography (SiO_2, 0 → 15% EtOAc in hexanes) followed by flash column chromatography (SiO_2, 0 → 5% EtOAc in benzene) to provide biaryl **51** (145 mg, 0.45 mmol, 13% yield) as a white powder. **Biaryl 51**: R_f 0.24 (9:1 hexanes:EtOAc); ^{1}H NMR (600 MHz, $CDCl_3$) δ 8.38 (dd, *J* = 4.8, 1.6 Hz, 1H), 7.99 (dd, *J* = 8.0, 1.2 Hz, 1H), 7.95 (dd, *J* = 7.8, 1.6 Hz, 1H), 7.46 (td, *J* = 7.4, 1.2 Hz, 1H), 7.39 (dd, *J* = 7.6, 1.7 Hz, 1H), 7.16 (td, *J* = 7.7, 1.7 Hz, 1H), 7.11 (dd, *J* = 7.8, 4.7 Hz, 1H), 6.44 (s, 1H), 3.67 (s, 3H); ^{13}C NMR (150 MHz, $CDCl_3$) δ 148.3, 143.1, 142.9, 139.3, 138.2, 131.8, 130.6, 128.7, 128.1, 120.3, 116.2, 101.0, 100.0, 29.5; IR (film): 3050, 1462, 1404, 1296, 767 cm^{-1}; HRMS-APCI (*m/z*) [M + H]$^+$ calcd for $C_{14}H_{12}IN_2^+$, 335.00397; found 335.00293.

B. Annulation of Anti-Bredt Olefins

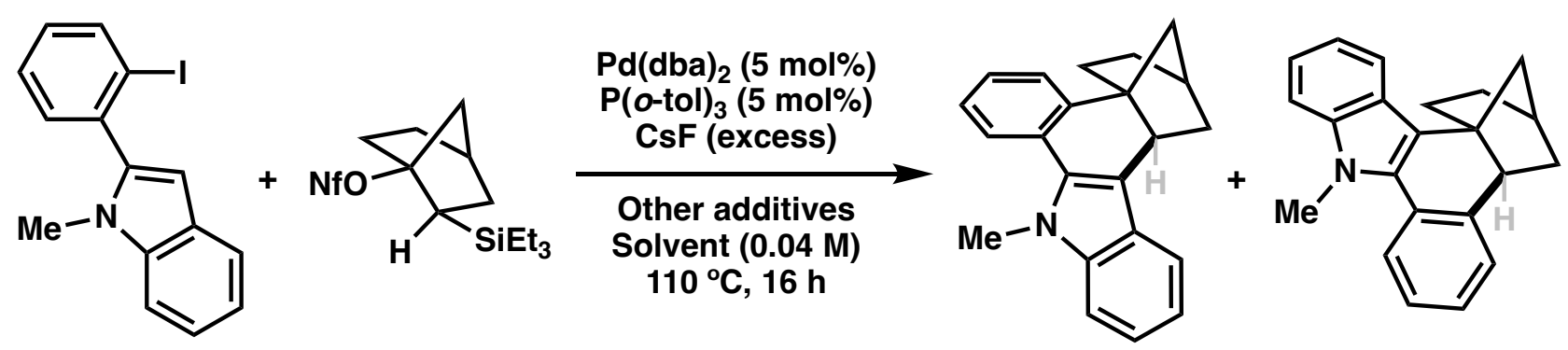

General Procedure A for the optimization of ABO annulation. A 1-dram vial (A) was charged with biaryl **6** (0.016 mmol, 1 equiv). A separate 1-dram vial (B) was charged with ABO precursor **7** (25 mg, 0.05 mmol, 3.0 equiv). A third (C) and fourth (D) 1-dram vial were each charged with a magnetic stir bar. All four vials (A–D) were brought into a nitrogen-filled glovebox, and further procedural steps were conducted within the glovebox. To prepare the catalyst stock solution, vial C (containing a stir bar) was charged with $Pd(dba)_2$ (10 mg, 0.017 mmol), P(*o*-tol)$_3$ (5 mg, 0.017 mmol), and toluene (1 mL). Vial C, containing the catalyst solution, was capped with a Teflon-lined cap and stirred at 80 °C for 15 min.

The reaction vial was prepared by adding to vial D (containing a stir bar): *n*Bu_4NBr (16 mg, 0.05 mmol, 3.0 equiv), Cs_2CO_3 (16 mg, 0.05 mmol, 3.0 equiv), and CsF (75 mg, 0.48 mmol, 30 equiv), sequentially. To vial A containing biaryl **6** was added toluene (0.2 mL) and this solution was transferred to vial D. Then, to vial B containing ABO precursor **7** was added toluene (0.15 mL) and this solution was transferred to vial D. Then, a portion of the catalyst solution (0.05 mL, 5 mol%) from vial C was then added to reaction vial D. Vial D was capped with a Teflon-lined cap, removed from the glovebox, placed in a preheated aluminum block at 110 °C, and stirred at 750 rpm for 16 h. After this time, the reaction was cooled to 23 °C, then filtered through a 2 cm pad of silica gel in a monster pipette, eluting with diethyl ether (6 mL). The eluate was concentrated to provide a crude residue, which was analyzed by ^{1}H NMR using mesitylene as an external standard. *Any modification of the conditions shown in the general procedure above are specified in the following tables.*

Note: The large excess of CsF utilized is primarily due to experimental setup. When using CsF in a non-polar solvent (such as toluene) in small vessels, solid CsF can collect on the sides of the vessel out of the reaction solution. Thus, using CsF in a large excess ensures the necessary concentration of fluoride is maintained, leading to improved reproducibility.

Optimization of Trapping Reactions of ABO 2.

Results from the optimization of the reaction of silyl nonaflate **7** and biaryl **6** are included below. Experiments were conducted on 0.005–0.016 mmol scales. ^{1}H NMR yields and ratios of isomers were determined from the crude reaction mixture by using mesitylene as an external standard. Reactions were set up according to General Procedure A (see above), with modification as indicated in the table.

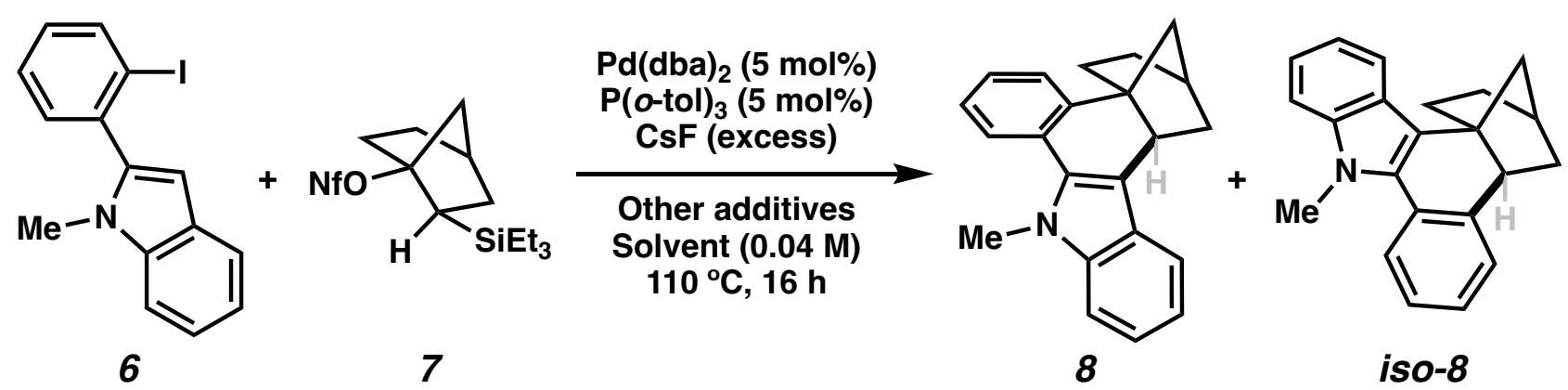

Entry	Equiv 7	Solvent	Other Additives	Yield 8 + iso-8 (ratio)
1	2	PhMe/MeCN (9:1)	–	0%
2	2	PhMe/MeCN (9:1)	*n*Bu_4NBr (3 equiv)	39% (1.1:1)
3	2	PhMe	*n*Bu_4NBr (3 equiv)	44% (1.3:1)
4	3	PhMe	*n*Bu_4NBr (3 equiv)	50% (1.3:1)
5	3	PhMe	*n*Bu_4NBr (3 equiv)<br>Cs_2CO_3 (3 equiv)	54% (1.3:1)

Table S1. ABO equivalents, solvent, and additive screening results. The minor difference in isomer ratio in entry 2 is presumably due to an artifact of measuring selectivity by NMR on small scale.

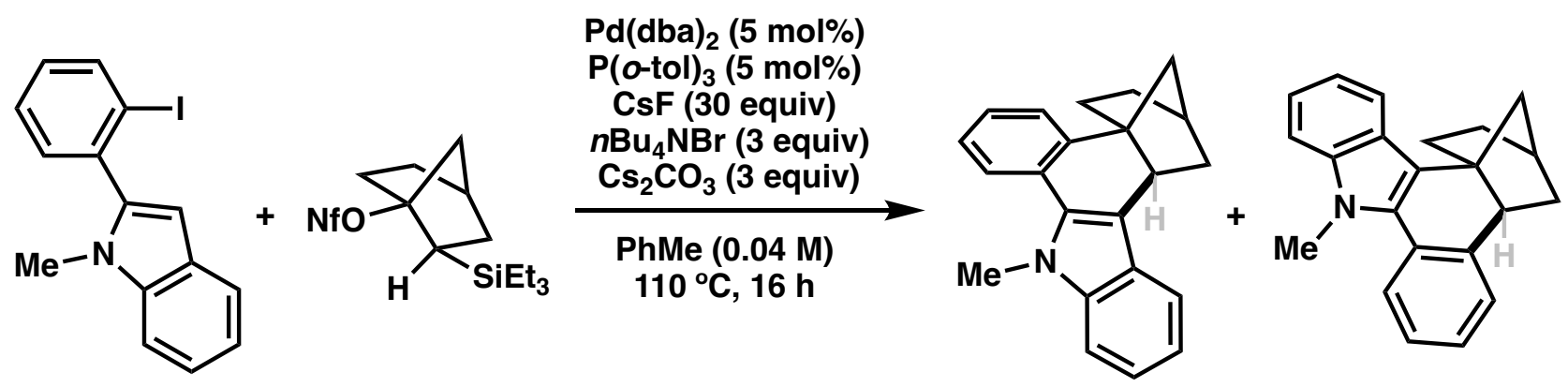

6 *7* *8* *iso-8*

Entry	*Modification to Conditions*	*Yield 8 + iso-8 (ratio)*	*Notes*
1	No CsF	0%	–
2	No $Pd(dba)_2$	0%	Recovered *6*
3	No $P(o\text{-tol})_3$	39% (1.3:1)	–
4	Anthracene (15 equiv) added	0%	[4+2] adduct *52* formed in 62% yield

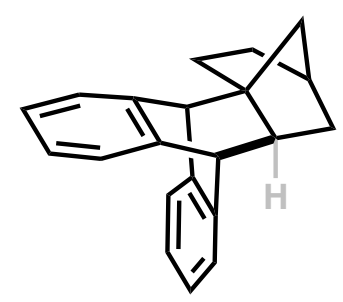

52

Table S2. Key control experiment results. Experiments were performed to validate the role of CsF, Pd, and ligand. Anthracene was added to validate the formation of ABO **2**, which was evidenced by the formation of [4+2] cycloadduct **52**.[2]

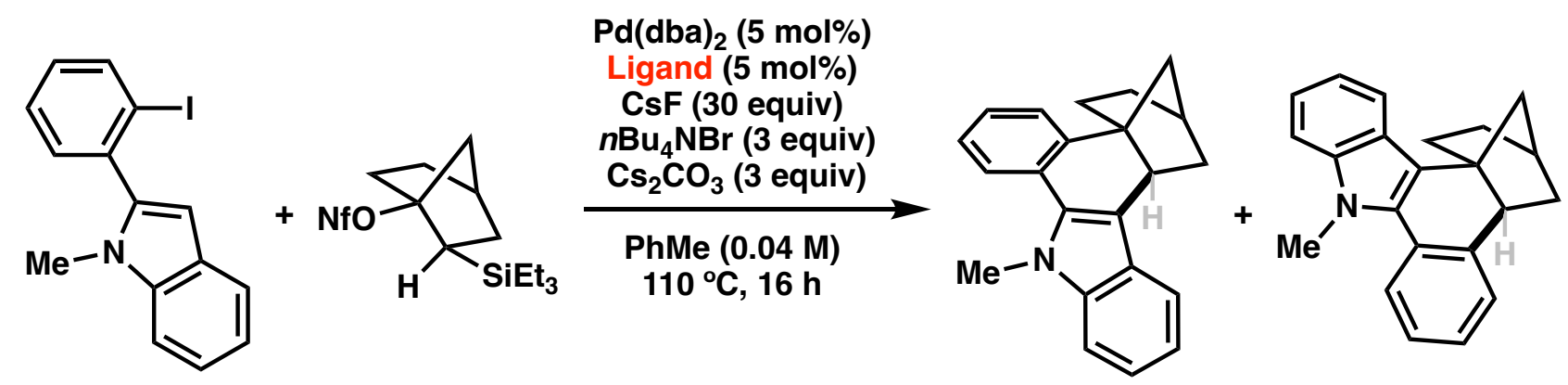

6 + 7 → 8 + iso-8

Entry	Ligand	Yield 8 + iso-8 (ratio)
1	L1	54% yield (1.3:1)
2	L2	54% yield (1.3:1)
3	L3	54% yield (1.3:1)
4	L4	22% yield (1.3:1)
5	L5	12% yield (1.3:1)
6	L6	5% yield (1.3:1)
7	PMe_3	36% yield (1.3:1)

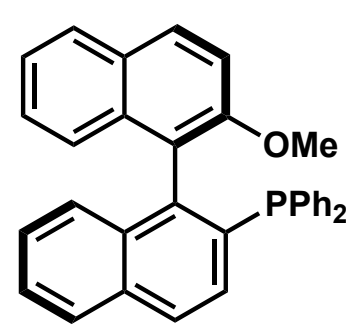

L1

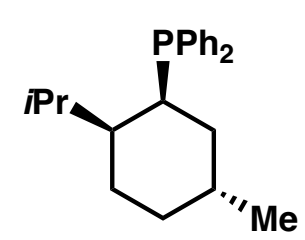

L2

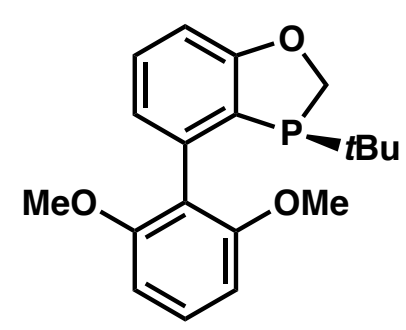

L3

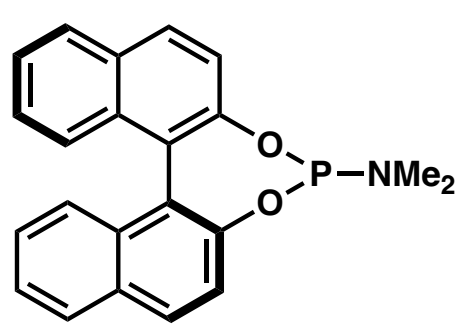

L4

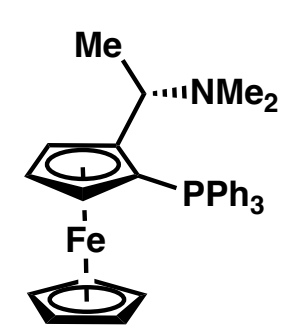

L5

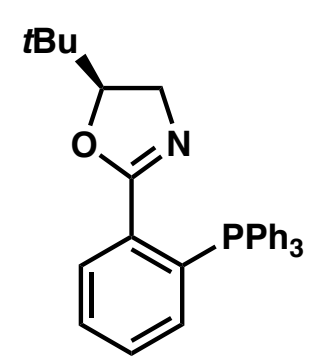

L6

Table S3. Ligand screening results. All ligands were premixed in PhMe with Pd(dba)$_2$ as indicated in General Procedure A.

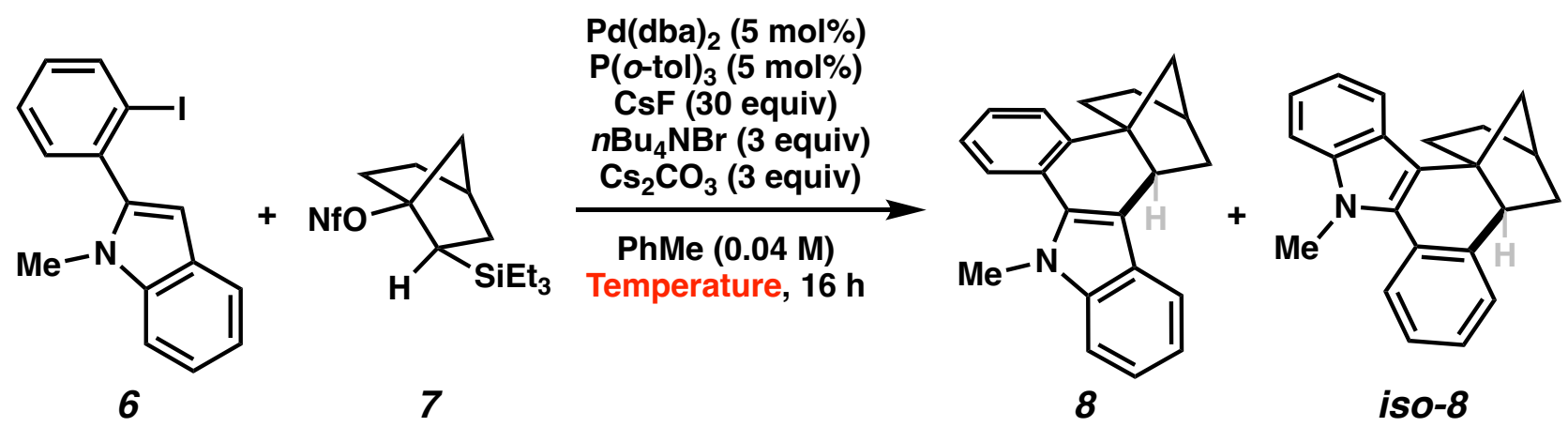

Entry	*Temperature*	*Yield 8 + iso-8 (ratio)*
1[a]	90 °C	53% yield (1.3:1)
2	110 °C	54% yield (1.3:1)
3	125 °C	46% yield (1.3:1)

Table S4. Reaction temperature screening results. Although 90 °C conditions provided similar yields, and these conditions were used for screening of several variables, 110 °C conditions were found to be optimal with regard to reproducibility and applicability to other substrates. [a]Entry 1 was run for 28 h to ensure reaction completion.

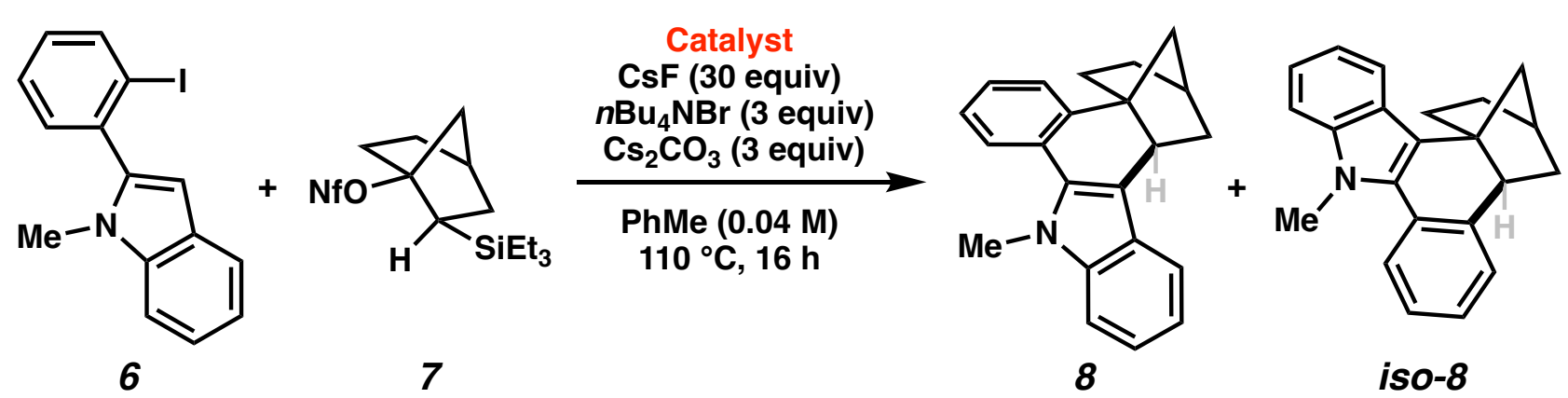

Entry	*Catalyst*	*Yield 8 + iso-8 (ratio)*
1	$Pd(dba)_2$ (5 mol%)/$P(o\text{-tol})_3$ (5 mol%)	53% yield (1.3:1)
2	Pd(COD)DQ (5 mol%)/$P(o\text{-tol})_3$ (5 mol%)	48% yield (1.3:1)
3	Pd(COD)DQ (5 mol%)	43% yield (1.3:1)
4	$Pd[P(o\text{-tol})_3]_2$ (5 mol%)	40% yield (1.3:1)
5[a]	$Pd[P(o\text{-tol})_3]_2Cl_2$ (5 mol%)	35% yield (1.3:1)

Table S5. Pd source screening results. [a]Entry 5 was conducted at 90 °C and ran for 28 h.

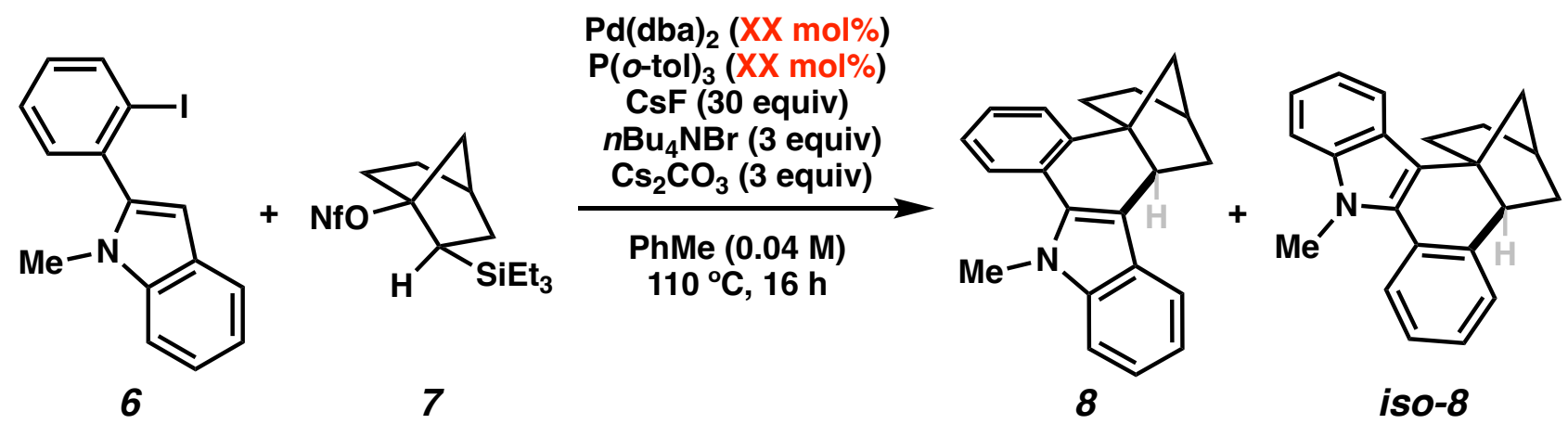

Entry	*Catalyst Loading*	*Ligand Loading*	*Yield 8 + iso-8*
1	2.5 mol%	2.5 mol%	34%
2	5 mol%	5 mol%	54%
3	10 mol%	10 mol%	52%
4	5 mol%	10 mol%	53%
5	10 mol%	5 mol%	42%

Table S6. Results from ligand and Pd loading screening.

Annulations of ABO 2

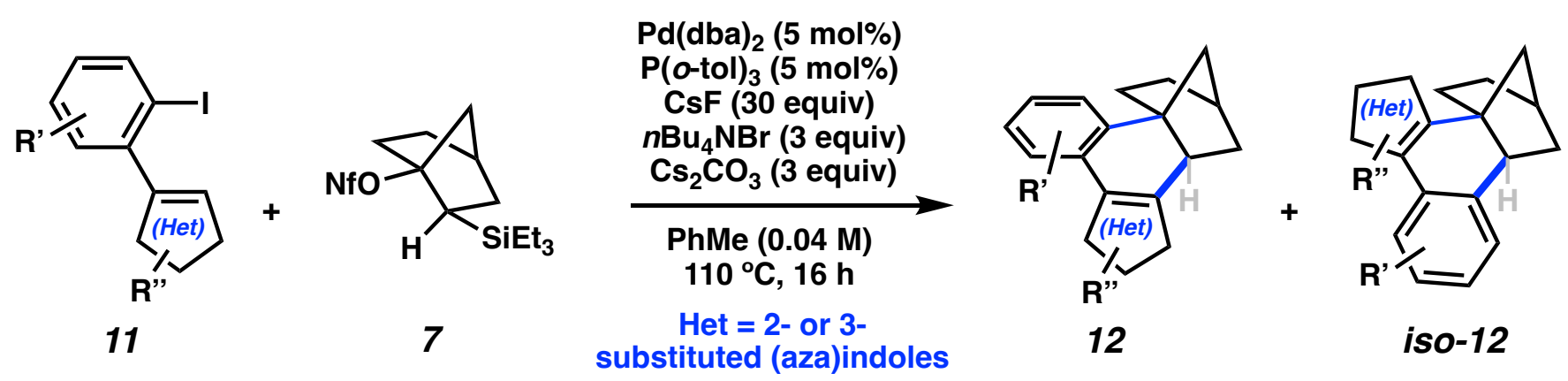

General Procedure B for the 0.05 mmol scale ABO annulation.

A 1-dram vial (A) was charged with biaryl 11 (0.05 mmol, 1 equiv). A separate 1-dram vial (B) was charged with ABO precursor **7** (75 mg, 0.15 mmol, 3.0 equiv). A third (C) and fourth (D) 1-dram vial were each charged with a magnetic stir bar. All four vials (A–D) were brought into a nitrogen-filled glovebox, and further procedural steps were conducted within the glovebox.

To prepare the catalyst stock solution, vial C (containing a stir bar) was charged with $Pd(dba)_2$ (10 mg, 0.017 mmol), $P(o\text{-tol})_3$ (5 mg, 0.017 mmol), and toluene (1 mL). Vial C, containing the catalyst solution, was capped with a Teflon-lined cap and stirred at 80 °C for 15 min. The reaction vial was prepared by adding to vial D (containing a stir bar): $n Bu_4NBr$ (48 mg, 0.15 mmol, 3.0 equiv), Cs_2CO_3 (48 mg, 0.15 mmol, 3.0 equiv), and CsF (220 mg, 1.50 mmol, 30 equiv), sequentially. To vial A containing biaryl **11** was added toluene (0.6 mL) and this solution was transferred to vial D containing the solid reagents. To vial B containing ABO precursor **7** was added toluene (0.6 mL) and this solution was transferred to vial D. The catalyst solution (0.15 mL, 5 mol%) from vial C was then added to vial D. Vial D was capped with a Teflon-lined cap, removed from the glovebox,

placed in a preheated aluminum block at 110 °C, and stirred at 750 rpm for 16 h. After this time, the reaction was cooled to 23 °C, then filtered through a 2 cm pad of silica gel in a monster pipette, eluting with ethyl acetate (10 mL). The eluate was concentrated to provide a crude residue, which was analyzed by ^{1}H NMR using mesitylene as an external standard. The crude material was purified by reverse phase column chromatography. In some cases, subsequent normal phase chromatography was used to remove traces of remaining impurities. Yields represent isolated material, and product ratios are reported as determined from the crude reaction mixture unless otherwise noted. Ratios of isolated products may vary slightly due to minor material loss during purification.

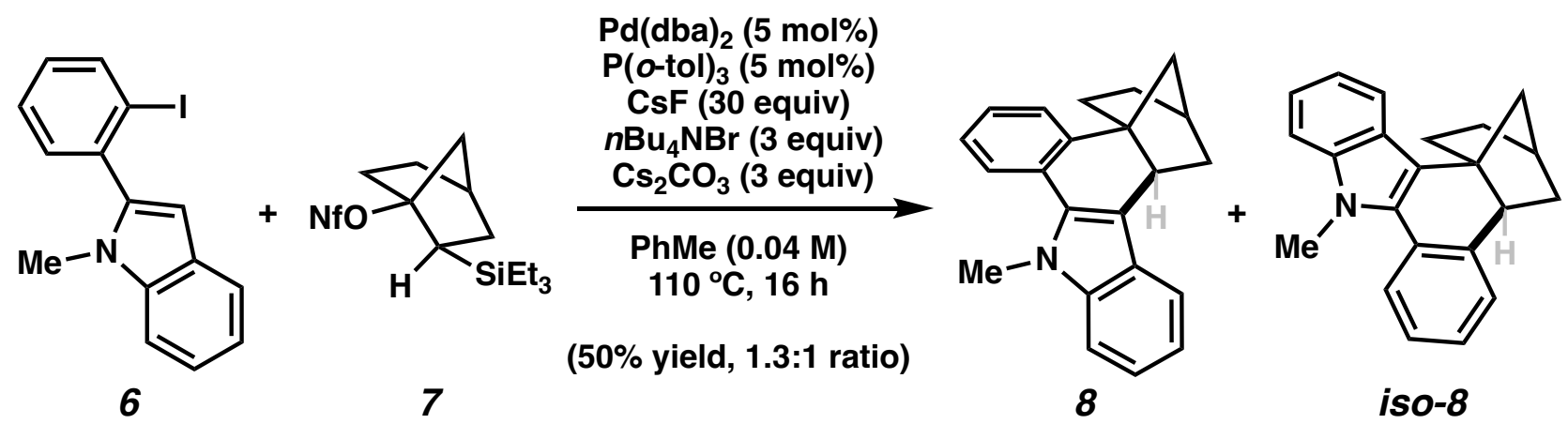

Annulation products 8 and *iso*-8. Followed General Procedure B, utilizing **6**. Purification by automated reverse phase flash column chromatography (C18, 40 → 100% MeCN in H_2O), followed by automated flash column chromatography (SiO_2, 0 → 10% EtOAc in hexanes) yielded an inseparable mixture of annulation products **8** and ***iso*-8** (50% yield, 1.3:1 ratio by ^{1}H NMR, average of two experiments) as a clear colorless film. **Annulation product 8**: R_f 0.60 (9:1 hexanes:EtOAc); ^{1}H NMR (500 MHz, $CDCl_3$): δ 7.77 (dd, J = 8.0, 1.3 Hz, 1H), 7.64 (dd, J = 7.5, 1.4 Hz, 1H), 7.56 (dt, J = 7.9, 0.8 Hz, 1H), 7.38 – 7.34 (m, 1H), 7.31 (td, J = 7.7, 1.4 Hz, 1H), 7.28 – 7.21 (overlapped with residual solvent, 2H), 7.13 – 7.07 (m, 1H), 4.05 (s, 3H), 3.16 (dd, J = 7.6, 4.6 Hz, 1H), 2.56 – 2.45 (m, 1H), 2.36 – 2.17 (m, 3H), 1.95 – 1.85 (m, 1H), 1.81 (dq, J = 9.6, 1.6 Hz, 1H), 1.56 – 1.45 (overlapped with residual water, 2H), 1.27 – 1.23 (m, 1H); **Annulation product *iso*-8**: R_f 0.60 (9:1 hexanes:EtOAc); ^{1}H NMR (500 MHz, $CDCl_3$): δ 7.77 (d, J = 8.0 Hz, 1H), 7.72 – 7.69 (m, 1H), 7.38 – 7.34 (m, 1H), 7.28 – 7.21 (overlapped with residual solvent, 4H), 7.13 – 7.07 (m, 1H), 4.06 (s, 3H), 3.05 – 3.00 (m, 1H), 2.94 – 2.85 (m, 1H), 2.36 – 2.17 (m, 2H), 2.06 – 1.99 (m, 1H), 1.95 – 1.85 (m, 1H), 1.62 – 1.58 (m, 1H), 1.56 – 1.45 (overlapped with residual water, 2H), 1.40 (dq, J = 9.5, 2.0 Hz, 1H); **Annulation products 8 and iso-8**: ^{13}C NMR (125 MHz, $CDCl_3$, combined): δ 142.3, 140.32, 140.30, 140.29, 134.6, 132.7, 129.2, 128.0, 127.8, 126.8, 126.4, 126.3, 126.2, 126.1, 125.9, 125.8, 123.0, 122.18, 122.15, 122.0, 120.7, 120.0, 119.4, 119.3, 118.3, 114.9, 109.8, 109.7, 50.3, 46.6, 46.58, 43.9, 41.4, 40.4, 40.2, 39.8, 37.0, 36.4, 36.1, 34.3, 33.7, 33.6, 29.9, 29.2; IR (film): 2925, 1738, 1466, 1366, 736 cm^{-1}; HRMS-APCI (*m/z*) [M + H]$^+$ calcd for $C_{22}H_{22}N^+$, 300.17468; found 300.17435.

The structures of **8** and ***iso*-8** were verified by 2D-NOESY of the mixture, as the following interactions were observed:

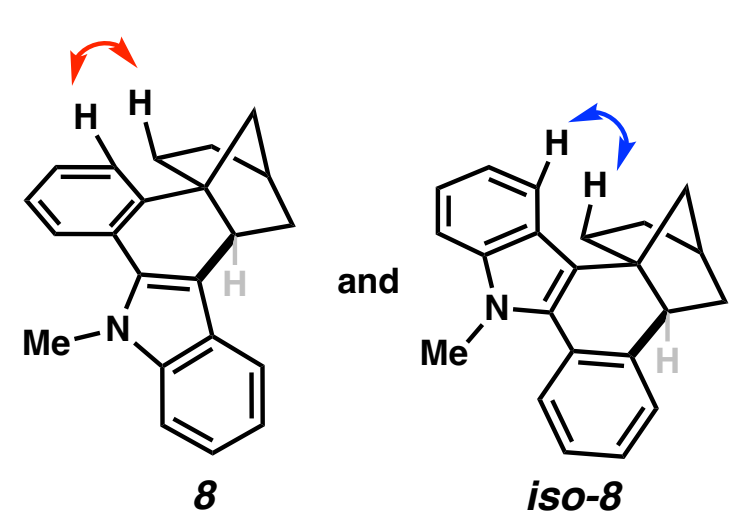

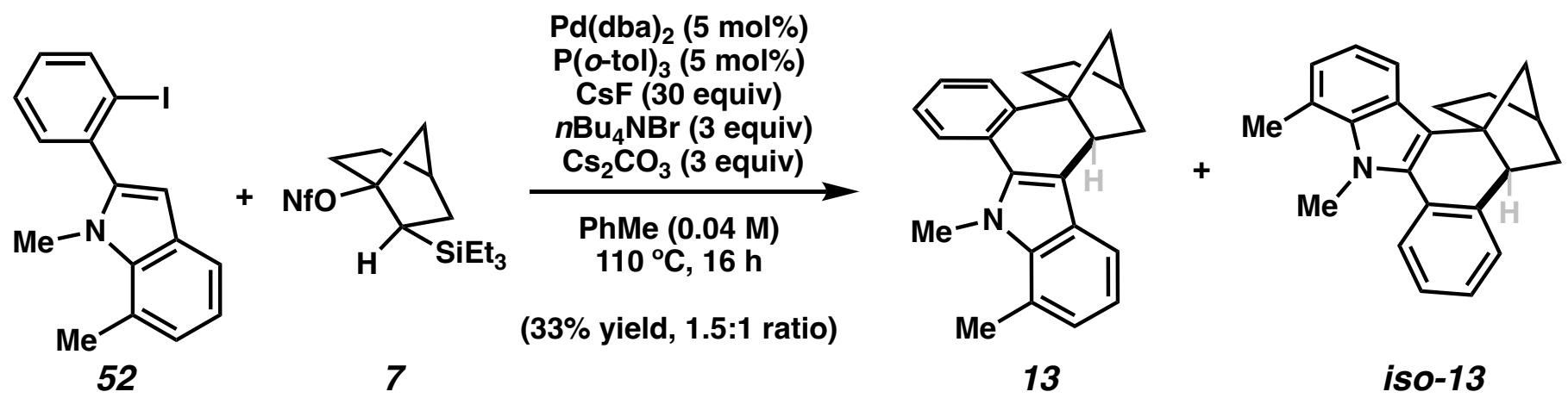

Annulation products 13 and *iso*-13. Followed General Procedure B, utilizing **52**. Purification by automated reverse phase flash column chromatography (C18, 20 → 100% MeCN in H_2O), followed by automated flash column chromatography (SiO_2, 100% hexane) yielded an inseparable mixture of annulation products **13** and ***iso*-13** (33% yield, 1.5:1 ratio by ^{1}H NMR, average of two experiments) as a pale yellow oil. **Annulation product 13**: R_f 0.69 (9:1 hexanes:EtOAc); ^{1}H NMR (600 MHz, $CDCl_3$): δ 7.63 (d, J = 7.8 Hz, 1H), 7.60 (d, J = 7.8 Hz, 2H), 7.31 (t, J = 7.5 Hz, 1H), 7.28 – 7.20 (overlapped with residual solvent, 1H), 7.02 – 6.98 (m, 1H), 6.97 – 6.94 (m, 1H), 4.09 (s, 3H), 3.01 (dd, J = 8.4, 5.8 Hz, 1H), 2.79 (s, 3H), 2.55 – 2.45 (m, 1H), 2.35 – 2.30 (m, 1H), 2.30 – 2.22 (m, 1H), 2.21 – 2.16 (m, 1H), 2.03 – 1.97 (m, 1H), 1.86 – 1.82 (m, 1H), 1.54 – 1.50 (overlapped with residual water, 2H), 1.29 – 1.24 (m, 1H); **Annulation product *iso*-13**: R_f 0.69 (9:1 hexanes:EtOAc); ^{1}H NMR (600 MHz, $CDCl_3$): 7.52 (d, J = 7.8 Hz, 1H), 7.38 (d, J = 7.1 Hz, 1H), 7.28 – 7.20 (overlapped with residual solvent, 3H), 7.03 – 6.98 (m, 1H), 6.97 – 6.94 (m, 1H), 4.10 (s, 3H), 3.08 (dd, J = 8.9, 5.1 Hz, 1H), 2.78 (s, 3H), 2.35 – 2.30 (m, 1H), 2.30 – 2.22 (m, 1H), 2.21– 2.16 (m, 1H), 1.94 – 1.86 (m, 2H), 1.54 – 1.50 (overlapped with residual water, 2H), 1.47 – 1.43 (m, 1H), 1.36 – 1.32 (m, 1H); **Annulation product 13 and *iso*-13**: ^{13}C NMR (125 MHz, $CDCl_3$, combined, 45 of 46 signals observed): δ 142.0, 141.9, 141.7, 140.3, 137.4, 135.3, 128.8, 127.8, 127.7, 127.6, 127.3, 126.7, 126.3, 126.1, 125.7, 125.4, 125.3, 123.8, 123.0, 122.2, 122.1, 120.12, 120.07, 119.7, 118.6, 118.0, 116.3, 50.3, 46.7, 46.6, 43.7, 40.8, 40.2, 40.1, 39.6, 37.1, 37.0, 36.7, 36.5, 36.1, 34.0, 29.8, 29.2, 20.5, 20.4; IR (film): 2948, 2869, 1494, 1456, 1397 cm^{-1}; HRMS-APCI (m/z) $[M + H]^+$ calcd for $C_{23}H_{24}N^+$, 314.1903; found 314.1894.

The structure of **13** and ***iso*-13** were verified by 2D-NOESY of the mixture, as the following interactions were observed:

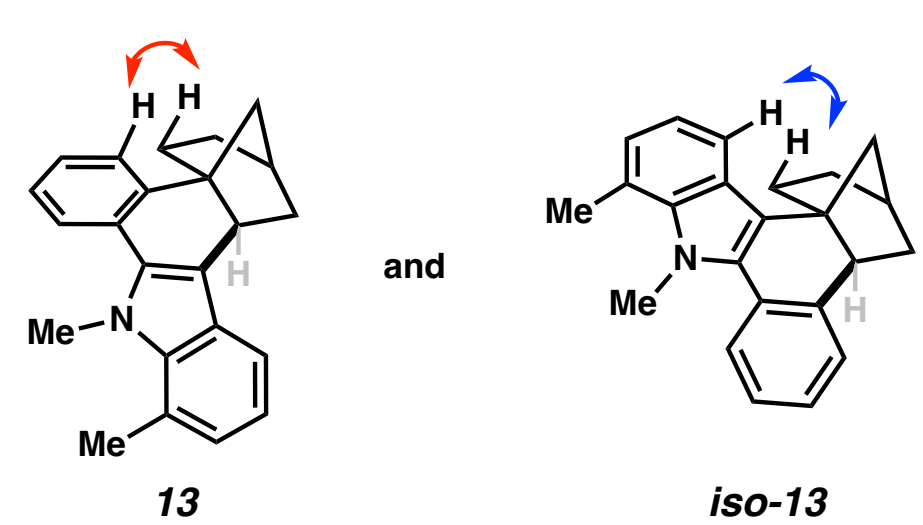

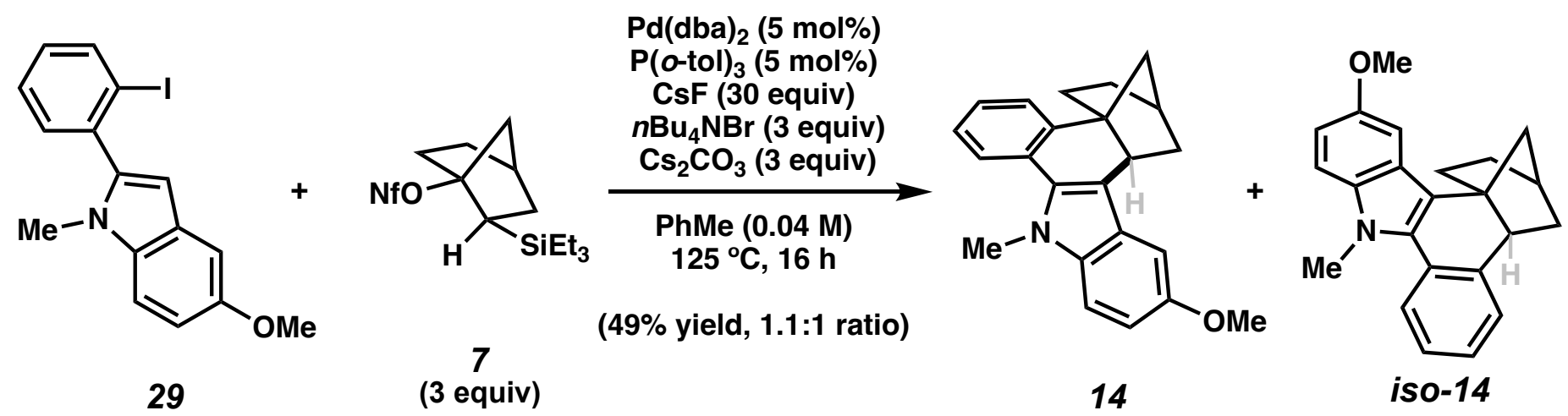

Annulation products 14 and *iso*-14. Followed General Procedure B but run at 125 °C, utilizing **29**. Purification by automated reverse phase flash column chromatography (C18, 40 → 100% MeCN in H_2O), followed by automated flash column chromatography (SiO_2, 0 → 20% EtOAc in hexanes) yielded an inseparable mixture of annulation products **14** and ***iso*-14** (49% yield, 1.1:1 ratio by ^1H NMR, average of two experiments) as a colorless oil. **Annulation product 14**: R_f 0.44 (1:1 hexanes:benzene); ^1H NMR (600 MHz, $CDCl_3$) δ 7.74 (d, J = 7.8 Hz, 1H), 7.64 (d, J = 7.7 Hz, 1H), 7.30 (t, J = 7.6 Hz, 1H), 7.27 – 7.11 (overlapped with residual solvent, 2H), 7.00 (s, 1H), 6.90 (dd, J = 8.6, 2.4 Hz, 1H), 4.01 (s, 3H), 3.88 (s, 3H), 3.13 (dd, J = 8.8, 5.1 Hz, 1H), 2.51 (dd, J = 12.3, 8.3 Hz, 1H), 2.37 – 2.16 (m, 3H), 1.95 – 1.86 (m, 1H), 1.82 (d, J = 9.7 Hz, 1H), 1.55 – 1.51 (overlapped with residual water, 2H), 1.30 – 1.23 (overlapped with residual solvent, 1H); **Annulation product *iso*-14**: R_f 0.44 (1:1 hexanes:benzene); ^1H NMR (600 MHz, $CDCl_3$): δ 7.70 – 7.66 (m, 1H), 7.27 – 7.11 (overlapped with residual solvent, 5H), 6.92 (dd, J = 8.6, 2.4 Hz, 1H), 4.02 (s, 3H), 3.89 (s, 3H), 3.00 (dd, J = 8.7, 6.2 Hz, 1H), 2.85 (td, J = 12.0, 3.8 Hz, 1H), 2.37 – 2.16 (m, 2H), 2.04 – 1.98 (m, 1H), 1.95 – 1.86 (m, 1H), 1.60 (d, J = 9.8, 1H), 1.55 – 1.51 (overlapped with residual water, 1H), 1.51 – 1.44 (m, 1H), 1.39 (d, J = 9.9 Hz, 1H); **Annulation product 14 and *iso*-14**: ^{13}C NMR (150 MHz, $CDCl_3$, combined) δ 153.9, 153.8, 142.3, 140.2, 135.9, 135.8, 135.4, 133.4, 129.2, 128.0, 127.9, 126.8, 126.4, 126.31, 126.28, 126.2, 126.1, 125.8, 122.9, 122.1, 117.8, 114.3, 112.0, 111.6, 110.38, 110.36, 103.5, 102.1, 56.4, 56.2, 50.3, 46.60, 46.58, 44.0, 41.2, 40.3, 40.2, 39.6, 37.0, 36.4, 35.8, 34.3, 33.8, 33.7, 29.9, 29.2; IR (film): 2945, 2869, 1736, 1619, 1487 cm^{-1}; HRMS-APCI (*m/z*) $[M + H]^+$ calcd for $C_{23}H_{24}NO^+$, 330.1852; found 330.1834.

The structures of **14** and ***iso*-14** were verified by 2D-NOESY of the mixture, as the following interactions were observed:

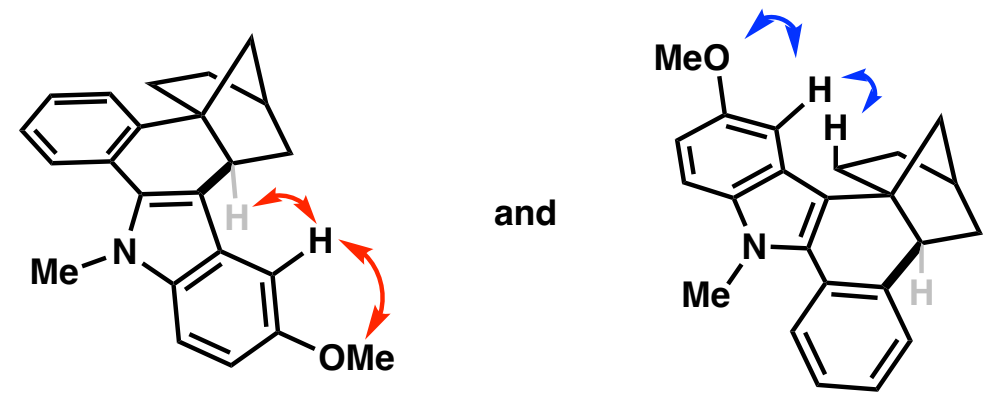

14 and ***iso*-14**

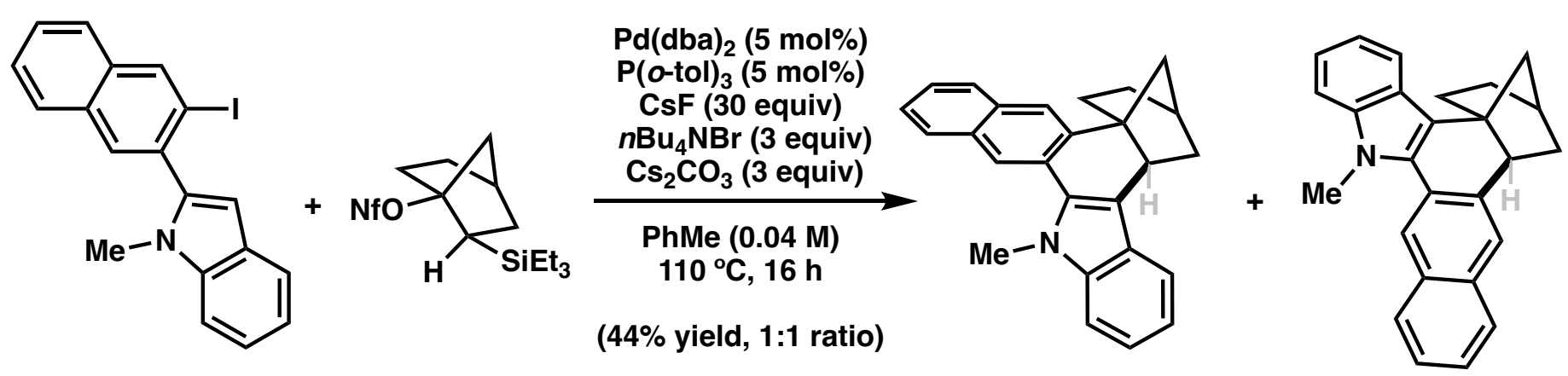

33, **7**, **15**, ***iso*-15**

Annulation products 15 and *iso*-15. Followed General Procedure B, utilizing **33**. Purification by automated reverse phase flash column chromatography (C18, 40 → 100% MeCN in H_2O), followed by automated flash column chromatography (SiO_2, 0 → 10% EtOAc in hexanes) yielded an inseparable mixture of annulation products **15** and ***iso*-15** (44% yield, 1:1 ratio by ^{1}H NMR, average of two experiments) as a pale yellow oil. **Annulation product 15**: R_f 0.59 (9:1 hexanes:EtOAc); ^{1}H NMR (500 MHz, $CDCl_3$) δ 8.08 (s, 1H), 8.06 (s, 1H), 7.86 – 7.74 (m, 2H), 7.60 (d, J = 7.9 Hz, 1H), 7.46 – 7.38 (m, 3H), 7.30 – 7.26 (overlapped with residual solvent, 1H), 7.15 – 7.10 (m, 1H), 4.18 (s, 3H), 3.21 (dd, J = 8.8, 5.1 Hz, 1H), 2.70 (td, J = 12.2, 4.2 Hz, 1H), 2.39 – 2.36 (m, 1H), 2.36 – 2.31 (m, 1H), 2.29 – 2.22 (m, 1H), 2.01 – 1.90 (m, 1H), 1.90 – 1.85 (m, 1H), 1.69 – 1.62 (m, 1H), 1.62 – 1.48 (overlapped with residual water, 1H), 1.32 – 1.28 (m, 1H); **Annulation product *iso*-15**: R_f 0.59 (9:1 hexanes:EtOAc); ^{1}H NMR (500 MHz, $CDCl_3$) δ 8.13 (s, 1H), 7.86 – 7.74 (m, 3H), 7.67 (s, 1H), 7.44 – 7.38 (m, 3H), 7.30 – 7.26 (overlapped with residual solvent, 1H), 7.15 – 7.10 (m, 1H), 4.17 (s, 3H), 3.16 – 3.10 (m, 1H), 2.98 – 2.89 (m, 1H), 2.42 – 2.39 (m, 1H), 2.36 – 2.31 (m, 1H), 2.22 – 2.16 (m, 1H), 2.01 – 1.90 (m, 1H), 1.69 – 1.62 (m, 1H), 1.62 – 1.48 (overlapped with residual water, 2H), 1.41 – 1.37 (m, 1H); **Annulation products 15 and *iso*-15**: ^{13}C NMR (125 MHz, $CDCl_3$, combined): δ 141.3, 140.8, 140.7, 138.7, 134.6, 132.64, 132.61, 132.34, 132.27, 132.1, 127.8, 127.7, 127.58, 127.54, 127.3, 126.5, 126.4, 126.1, 126.00, 125.96, 125.84, 125.80, 125.79, 124.8, 122.6, 122.5, 121.2, 120.9, 120.5, 120.2, 119.9, 119.5, 119.4, 116.4, 109.9, 109.8, 50.5, 46.9, 46.8, 44.8, 41.8, 40.4, 40.3, 39.8, 37.6, 37.0,

35.9, 34.3, 33.9, 33.8, 30.0, 29.5; IR (film): 3051, 2946, 2868, 1469, 730 cm^{-1}; HRMS-APCI (*m/z*) $[M + H]^+$ calcd for $C_{26}H_{24}N^+$, 350.19033; found 350.18815.

The structure of **15** and ***iso*-15** were verified by 2D-NOESY of the mixture, as the following interactions were observed:

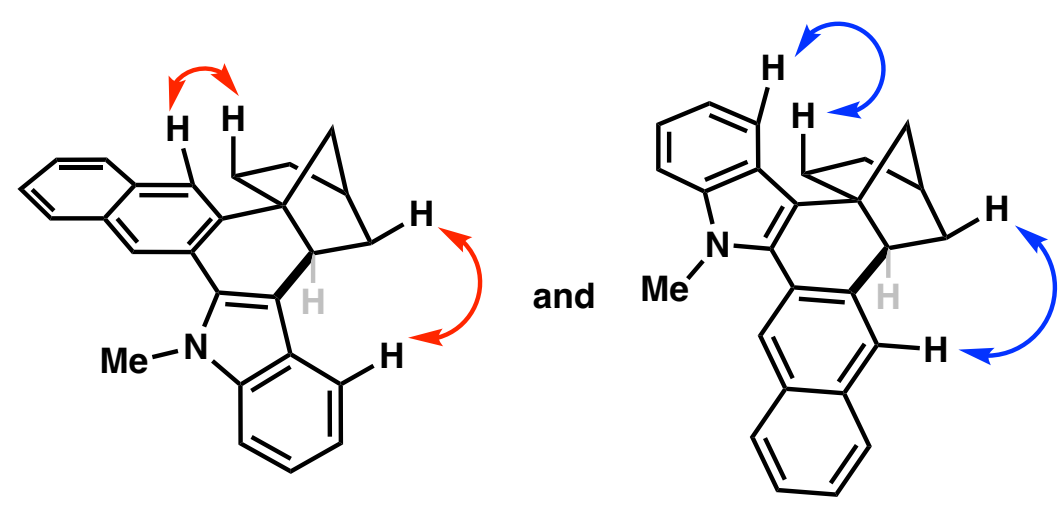

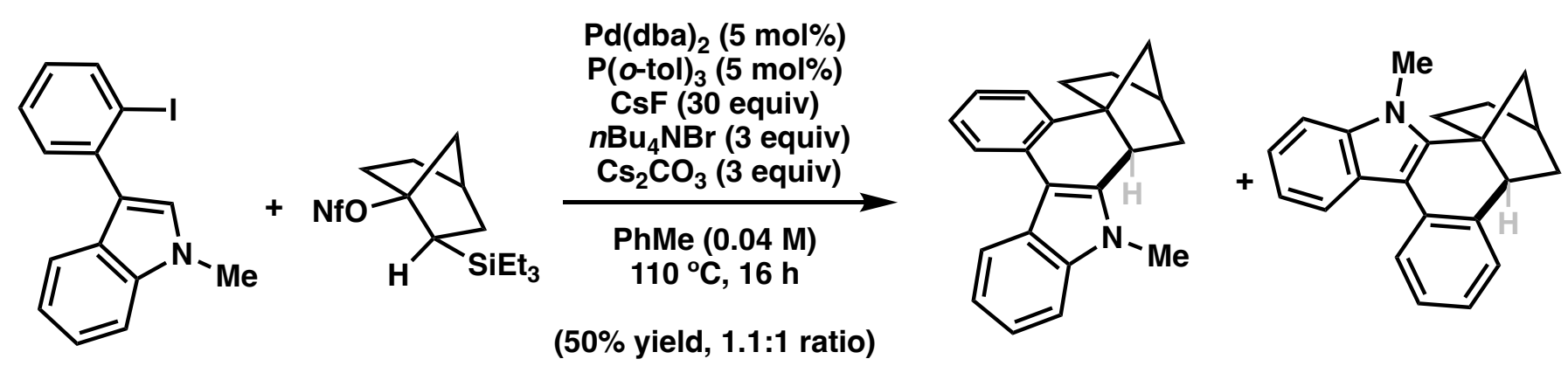

Annulation products 16 and *iso*-16. Followed General Procedure B, utilizing **35**. Purification by automated reverse phase flash column chromatography (C18, 20 → 100% MeCN in H_2O) yielded an inseparable mixture of annulation products **16** and ***iso*-16** (50% yield, 1.1:1 ratio by ^{1}H NMR, average of two experiments) as a pale yellow oil. **Annulation product 16**: R_f 0.57 (9:1 hexanes:EtOAc); ^{1}H NMR (500 MHz, $CDCl_3$): δ 8.13–8.10 (m, 1H), 8.03 (d, *J* = 7.7 Hz, 1H), 7.53 (d, *J* = 7.7 Hz, 1H), 7.34 – 7.31 (m, 1H), 7.26 – 7.20 (overlapped with residual solvent, 3H), 7.13 – 7.09 (m, 1H), 3.72 (s, 3H), 3.19 (dd, *J* = 14.7, 7.6 Hz, 1H), 2.42 (s, 1H), 2.33 – 2.32 (m, 1H), 1.97 – 1.92 (m, 1H), 1.91 – 1.85 (m, 1H), 1.68 (d, *J* = 9.8 Hz, 1H), 1.60 – 1.50 (overlapped with residual water, 3H), 1.26 (s, 1H); **Annulation product *iso*-16**: R_f 0.57 (9:1 hexanes:EtOAc); ^{1}H NMR (500 MHz, $CDCl_3$): δ 8.14 (d, *J* = 8.1 Hz, 1H), 7.98 (d, *J* = 7.7 Hz, 1H), 7.35 (d, *J* = 7.9 Hz, 1H), 7.30 (d, *J* = 7.5 Hz, 1H), 7.26 – 7.20 (overlapped with residual solvent, 2H), 7.16 (d, *J* = 7.6 Hz, 1H), 7.13 – 7.09 (m, 1H), 3.88 (s, 3H), 3.15 (t, *J* = 15.3 Hz, 1H), 2.70 – 2.63 (m, 1H), 2.58 – 2.51(m, 1H), 2.32 – 2.30 (m, 1H), 2.29 – 2.27 (m, 1H), 2.07 – 2.02 (m, 1H), 2.02 – 1.97 (m, 1H), 1.80 (d, *J* = 10.1 Hz, 1H), 1.60 – 1.50 (overlapped with residual water, 1H), 1.33 (d, *J* = 10.1 Hz, 1H); **Annulation products 16 and *iso*-16**: ^{13}C NMR (125 MHz, $CDCl_3$, combined): δ 142.8, 140.1, 139.1, 138.5, 137.6, 135.5, 133.8, 132.6, 126.7, 126.5, 126.2, 124.80, 124.78, 124.6, 124.5,

124.0, 122.6, 121.9, 121.4, 121.1, 120.4, 120.32, 120.31, 120.25, 110.2, 109.8, 109.30, 109.27, 107.9, 51.1, 48.2, 46.3, 45.7, 41.2, 40.7, 39.8, 39.7, 36.08, 36.06, 34.3, 32.7, 31.2, 29.3, 29.1; IR (film): 2949, 1470, 1369, 1022, 748 cm^{-1}; HRMS-APCI (*m/z*) $[M + H]^+$ calcd for $C_{22}H_{22}N^+$, 300.1747; found 300.1741.

The structure of **16** and ***iso*-16** were verified by 2D-NOESY of the mixture, as the following interactions were observed:

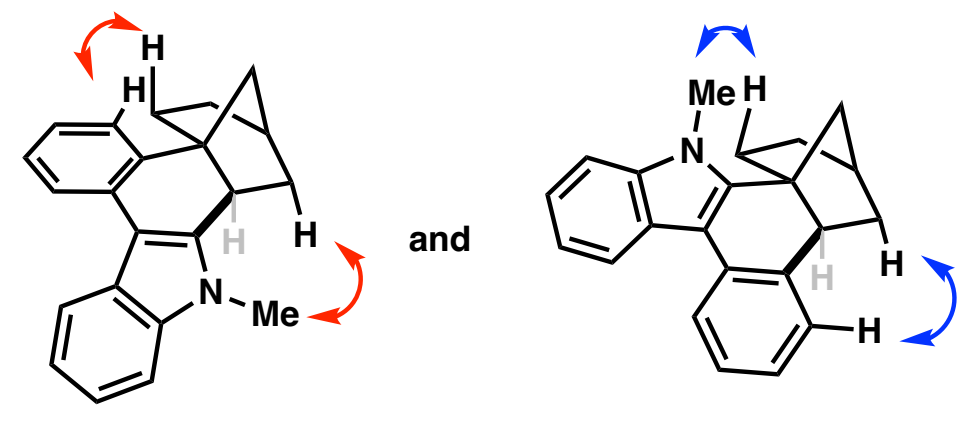

16 ***iso-16***

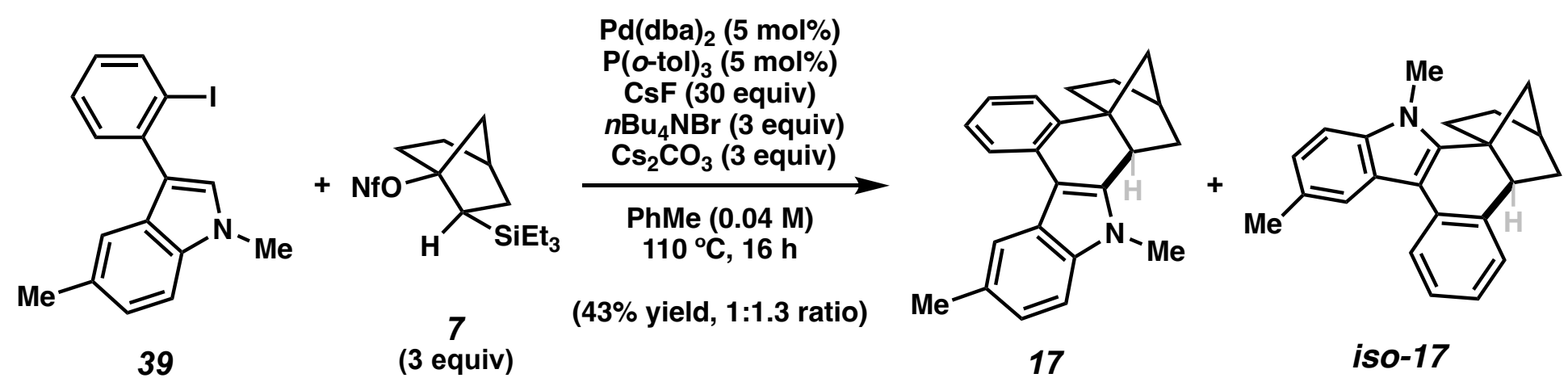

Annulation products 17 and *iso*-17. Followed General Procedure B, utilizing **39**. Purification by automated reverse phase flash column chromatography (C18, 20 → 100% MeCN in H_2O) followed by automated flash column chromatography (SiO_2, 0 → 20% EtOAc in hexanes) yielded an inseparable mixture of annulation products **17** and ***iso*-17** (43% yield, 1:1.3 ratio by ^{1}H NMR, average of two experiments) as a colorless amorphous solid. Annulation products were isolated in a 1:1.9 ratio due to minor material loss during purification. **Annulation product 17**: R_f 0.92 (18:1:1 benzene:CH_2Cl_2:diethyl ether); ^{1}H NMR (600 MHz, $CDCl_3$) δ 8.01 (d, J = 7.7 Hz, 1H), 7.90 (s, 1H), 7.52 (d, J = 7.7 Hz, 1H), 7.30 (t, J = 7.5 Hz, 1H), 7.26 – 7.20 (overlapped with residual solvent, 1H), 7.11 – 7.03 (m, 2H), 3.69 (s, 3H), 3.18 – 3.11 (m, 1H), 2.56 – 2.51 (m, 4H), 2.33 – 2.24 (m, 2H), 2.00 – 1.90 (m, 2H), 1.78 (d, J = 10.0 Hz, 1H), 1.58 – 1.49 (overlapped with residual water, 1H), 1.31 (d, J = 10.1 Hz, 1H), 1.26 (t, J = 7.0 Hz, 1H); **Annulation product *iso*-17**: R_f 0.92 (18:1:1 benzene:CH_2Cl_2:diethyl ether); ^{1}H NMR (600 MHz, $CDCl_3$) δ 7.97 (d, J = 7.7 Hz, 1H), 7.92 (s, 1H), 7.26 – 7.20 (overlapped with residual solvent, 2H), 7.14 (d, J = 7.6 Hz, 1H), 7.11 – 7.03 (m, 2H), 3.85 (s, 3H), 3.18 – 3.11 (m, 1H), 2.68 – 2.61 (m, 1H), 2.56 – 2.51 (m, 3H), 2.42 – 2.39 (m, 1H), 2.33 – 2.24 (m, 1H), 2.06 – 2.00 (m, 1H), 1.90 – 1.83 (m, 1H), 1.66 (d, J =

9.8 Hz, 1H), 1.58 – 1.49 (overlapped with residual water, 3H); **Annulation products 17 and *iso*-17**: ^{13}C NMR (125 MHz, $CDCl_3$, combined, 45 of 46 signals observed) δ 143.0, 140.2, 137.59, 137.56, 136.9, 135.5, 134.0, 132.8, 129.6, 129.5, 126.7, 126.5, 126.2, 125.0, 124.8, 124.7, 124.3, 123.9, 122.8, 122.53, 122.46, 121.9, 120.24, 120.15, 109.7, 108.94, 108.90, 107.5, 51.0, 48.2, 46.3, 45.7, 41.2, 40.7, 39.8, 39.7, 36.09, 36.06, 34.3, 32.7, 31.2, 29.3, 29.1, 21.89, 21.88; IR (film): 2923, 1547, 1490, 1375, 1301 cm^{-1}; HRMS-APCI (*m/z*) [M + H]$^+$ calcd for $C_{23}H_{24}N^+$, 314.1903; found 314.1898.

The structures of **17** and ***iso*-17** were verified by 2D-NOESY of the mixture, as the following interactions were observed:

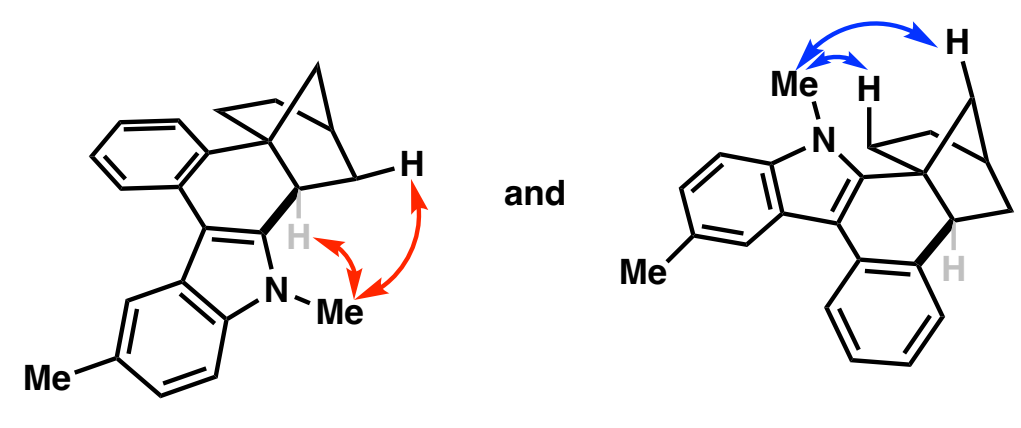

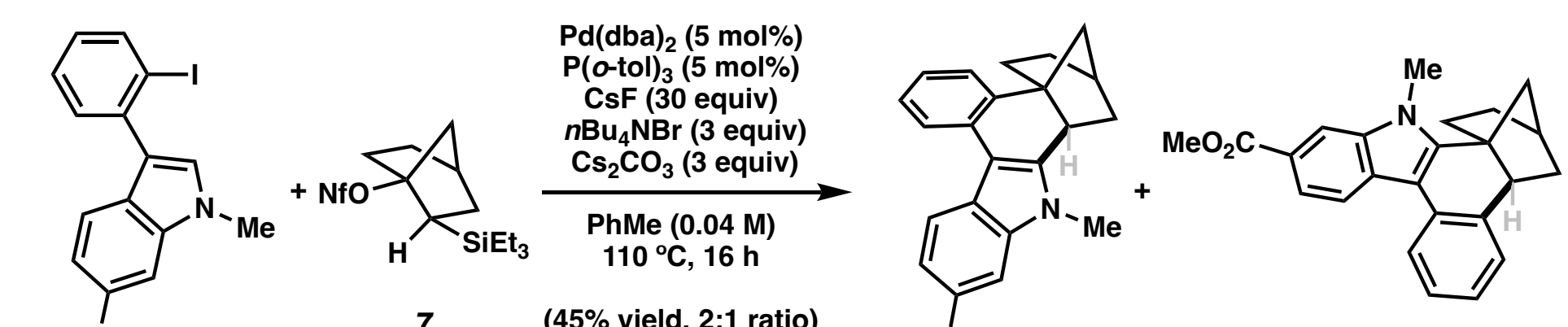

Annulation products 18 and *iso*-18. Followed General Procedure B, utilizing **43**. Purification by automated reverse phase flash column chromatography (C18, 20 → 100% MeCN in H_2O), followed by automated flash column chromatography (SiO_2, 0 → 20% EtOAc in hexanes) yielded an inseparable mixture of annulation products **18** and ***iso*-18** (45% yield, 2:1 ratio by ^{1}H NMR, average of two experiments) as a colorless oil. **Annulation product 18**: R_f 0.61 (18:1:1 benzene:CH_2Cl_2:diethyl ether); ^{1}H NMR (600 MHz, $CDCl_3$) δ 8.15 – 8.06 (m, 2H), 8.01 (d, *J* = 7.7 Hz, 1H), 7.90 (s, 1H), 7.54 (d, *J* = 7.7 Hz, 1H), 7.32 (t, *J* = 7.6 Hz, 1H), 7.19 – 7.12 (m, 1H), 3.96 (s, 3H), 3.78 (s, 3H), 3.21 – 3.13 (m, 1H), 2.55 (dd, *J* = 12.3, 7.3 Hz, 1H), 2.37 – 2.27 (m, 2H), 2.11 – 1.86 (m, 2H), 1.76 (d, *J* = 10.1 Hz, 1H), 1.61 – 1.52 (overlapped with residual water, 2H), 1.35 (d, *J* = 10.1 Hz, 1H); **Annulation product *iso*-18**: R_f 0.61 (18:1:1 benzene:CH_2Cl_2:diethyl ether); ^{1}H NMR (600 MHz, $CDCl_3$) δ 8.15 – 8.06 (m, 2H), 7.97 (d, *J* = 7.8 Hz, 1H), 7.89 (s, 1H), 7.29 – 7.27 (overlapped with residual solvent, 1H), 7.19 – 7.12 (m, 2H),

3.96 (s, 3H), 3.95 (s, 3H), 3.21 – 3.13 (m, 1H), 2.68 (t, *J* = 11.1 Hz, 1H), 2.44 (s, 1H), 2.37 – 2.27 (m, 1H), 2.11 – 1.86 (m, 2H), 1.70 (d, *J* = 9.8 Hz, 1H), 1.61 – 1.52 (overlapped with residual water, 2H), 1.26 (s, 1H); **Annulation products 18 and *iso*-18**: ^{13}C NMR (150 MHz, $CDCl_3$, 47 of 48 signals observed) δ 168.33, 168.31, 146.2, 143.8, 138.4, 137.9, 137.7, 135.6, 133.0, 131.8, 128.2, 128.0, 126.9, 126.7, 126.3, 125.1, 125.0, 124.7, 122.8, 122.7, 122.4, 122.1, 121.5, 119.7, 119.6, 111.51, 111.49, 110.8, 108.6, 52.11, 52.10, 51.0, 48.1, 46.4, 45.9, 41.3, 40.6, 39.9, 39.8, 36.2, 36.1, 36.0, 34.3, 32.9, 31.4, 29.2, 29.1; IR (film): 2950, 1712, 1915, 1464, 1434 cm^{-1}; HRMS-APCI (*m/z*) $[M]^+$ calcd for $C_{24}H_{23}NO_2^+$, 357.1723; found 357.1724.

The structures of **18** and ***iso*-18** were verified by 2D NOESY of the mixture, as the following interactions were observed:

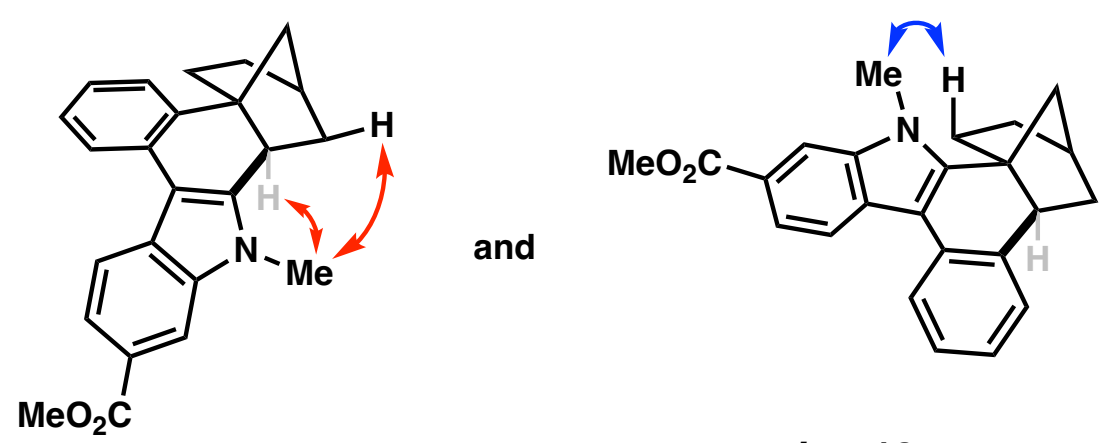

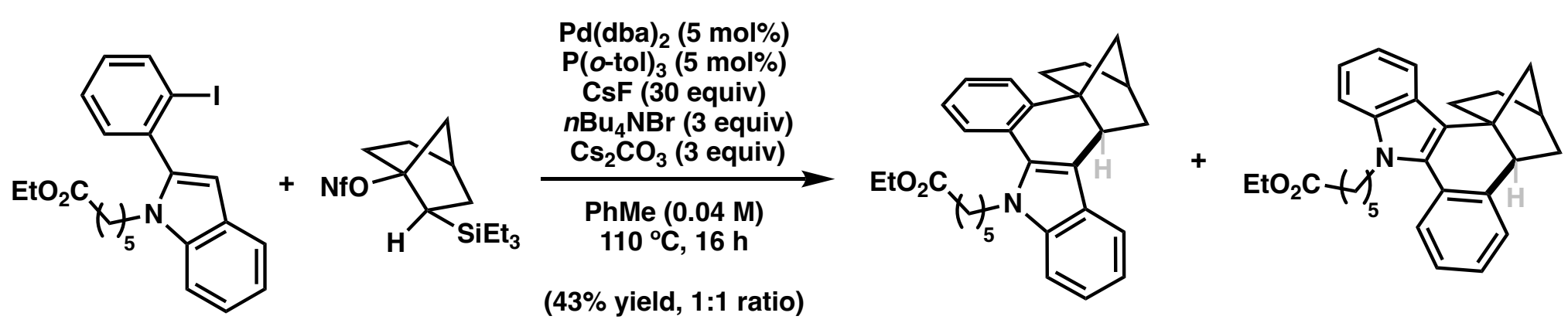

Annulation products 19 and *iso*-19. Followed General Procedure B, utilizing **46**. Purification by automated reverse phase flash column chromatography (C18, 20 → 100% MeCN in H_2O) followed by automated flash column chromatography (SiO_2, 100% hexanes) yielded an inseparable mixture of annulation products **19** and ***iso*-19** (43% yield, 1:1 ratio by ^1H NMR, average of two experiments) as a pale yellow oil. **Annulation product 19**: R_f 0.48 (9:1 hexanes:EtOAc); ^1H NMR (500 MHz, $CDCl_3$, **19**): δ 7.64 (dd, *J* = 12.0, 7.8 Hz, 2H), 7.56 (d, *J* = 7.6 Hz, 1H), 7.36 (dd, *J* = 11.0, 8.3 Hz, 1H), 7.28 – 7.20 (overlapped with residual solvent, 2H), 7.10 (q, *J* = 7.20 Hz, 2H), 4.50 – 4.42 (m, 1H), 4.39 – 4.30 (m, 1H), 4.13 (q, *J* = 7.63 Hz, 2H), 3.02 – 2.98 (m, 1H), 2.51 (dd, *J* = 12.7, 8.2 Hz, 1H), 2.35 – 2.28 (m, 4H), 2.23 – 2.19 (m, 1H), 2.11 – 2.05 (m, 1H), 1.93 – 1.86 (m, 3H), 1.79 – 1.76 (m, 1H), 1.76 – 1.72 (m, 1H), 1.72 – 1.68 (m, 1H), 1.53 (d, *J* = 8.3 Hz, 1H), 1.52 – 1.45 (m, 1H), 1.26 (q, *J* = 7.2 Hz, 5H); **Annulation**

product *iso*-19: ^{1}H NMR (500 MHz, $CDCl_3$): 7.78 (d, *J* = 8 Hz, 1H), 7.56 (d, *J* = 7.6 Hz, 1H), 7.36 (dd, *J* = 11.0, 8.3 Hz, 1H), 7.30 (t, *J* = 7.6 Hz, 1H), 7.28 – 7.20 (overlapped with residual solvent, 4H), 4.50 – 4.42 (m, 1H), 4.39 – 4.30 (m, 1H), 4.13 (q, *J* = 7.63 Hz, 2H), 3.15 (dd, *J* = 8.9, 5.0 Hz, 1H), 2.90 (td, *J* = 11.9, 4.0 Hz, 1H), 2.35 – 2.28 (m, 3H), 2.28 – 2.23 (m, 1H), 2.05 – 1.98 (m, 2H), 1.76 – 1.72 (m, 1H), 1.72 – 1.68 (m, 1H), 1.60 (d, *J* = 9.8 Hz, 1H), 1.53 (d, *J* = 8.3 Hz, 1H), 1.52 – 1.45 (m, 3H), 1.45 – 1.39 (m, 3H), 1.26 (q, *J* = 7.2, 3H); **Annulation products 19 and *iso*-19**: ^{13}C NMR (125 MHz, $CDCl_3$, combined, 56 of 58 signals observed): δ 173.64, 173.63, 142.4, 140.3, 139.9, 139.8, 133.7, 131.9, 129.2, 128.1, 127.8, 126.8, 126.45, 126.40, 126.3, 126.24, 126.16, 125.9, 122.4, 122.2, 122.0, 121.5, 120.8, 120.1, 119.44, 119.39, 118.8, 115.3, 110.0 (for two carbons), 60.45, 60.43, 50.2, 46.53, 46.45, 45.73, 45.66, 43.9, 41.5, 40.5, 40.2, 39.9, 37.0, 36.3, 36.1, 34.33, 34.31, 34.26, 30.15, 30.13, 29.9, 29.2, 26.61, 26.57, 24.78, 24.77, 14.40, 14.39; IR (film): 2944, 1733, 1464, 1349, 739 cm^{-1}; HRMS-APCI (*m/z*) $[M + H]^+$ calcd for $C_{29}H_{34}NO_2^+$, 428.2584; found 428.2571.

The structure of **19** and ***iso*-19** were verified by 2D-NOESY of the mixture, as the following interactions were observed:

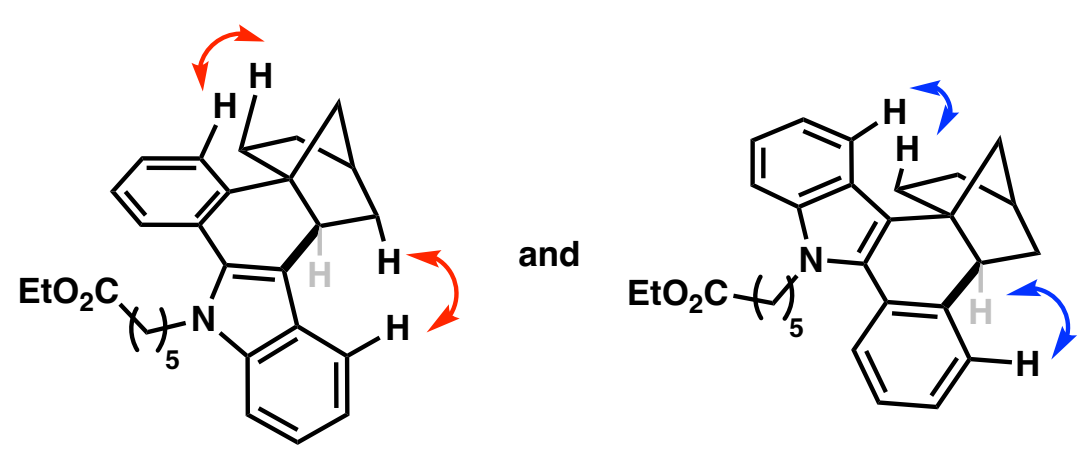

19 ***iso-19***

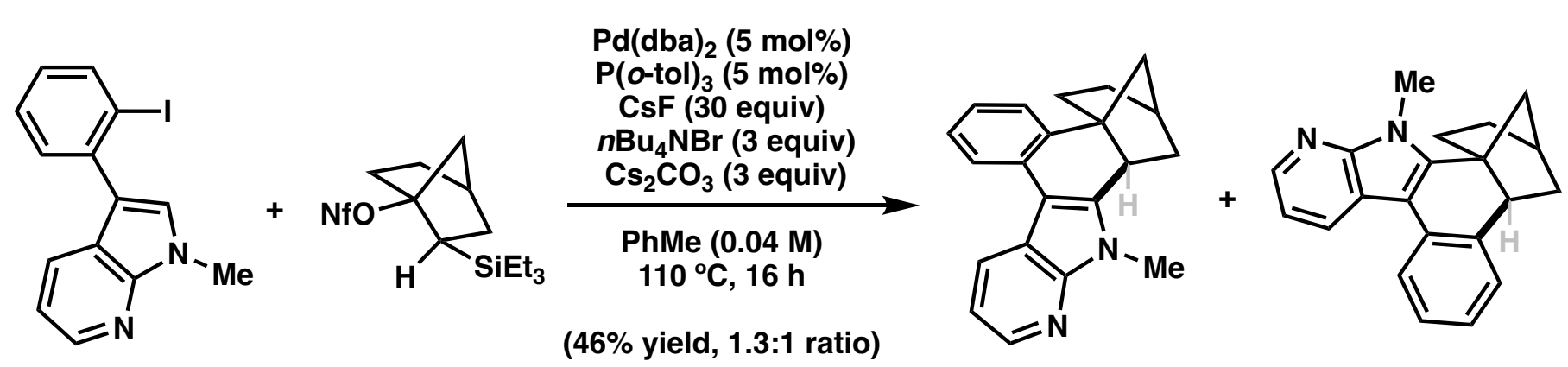

48 ***7*** ***20*** ***iso-20***

Annulation products 20 and *iso*-20. Followed General Procedure B, utilizing **48**. Purification by automated reverse phase flash column chromatography (C18, 30 → 100% MeCN in H_2O) yielded a mixture of annulation products **20** and ***iso*-20** (46% yield, 1.3:1 ratio by ^{1}H NMR, average of two experiments) as a clear colorless film. Analytically pure samples of each isomer could be obtained by preparative thin-layer chromatography (9:1 benzene:EtOAc). **Annulation product 20**: R_f 0.18 (9:1 hexanes:EtOAc); ^{1}H NMR (500 MHz, $CDCl_3$) δ 8.33 (dd, *J* = 7.8, 1.5 Hz, 1H), 8.29 (dd, *J* =

4.7, 1.5 Hz, 1H), 7.89 (dd, J = 7.8, 1.3 Hz, 1H), 7.54 (dd, J = 7.8, 1.4 Hz, 1H), 7.30 (dt, J = 7.5, 1.3 Hz, 1H), 7.16 – 7.12 (m, 2H), 3.84 (s, 3H), 3.22 (dd, J = 9.0, 6.0 Hz, 1H), 2.58 – 2.50 (m, 1H), 2.37 – 2.30 (m, 2H), 2.06 – 2.00 (m, 1H), 2.00 – 1.91 (m, 1H), 1.78 (dd, J = 10.0, 1.9 Hz, 1H), 1.60 – 1.55 (overlapped with residual water, 2H), 1.36 (ddt, J = 9.9, 2.7, 1.4 Hz, 1H); ^{13}C NMR (125 MHz, $CDCl_3$) δ 149.5, 142.9, 141.7, 135.7, 132.9, 127.8, 126.8, 125.0, 124.6, 122.5, 117.5, 116.3, 106.3, 50.9, 46.1, 40.3, 39.8, 35.9, 34.4, 29.7, 29.2; IR (film): 2949, 1720, 1593, 1460, 759 cm^{-1}; HRMS-APCI (m/z) $[M + H]^+$ calcd for $C_{21}H_{21}N_2^+$, 301.16993; found 301.16830.

The structure of **20** was verified by 2D-NOESY, as the following interaction was observed:

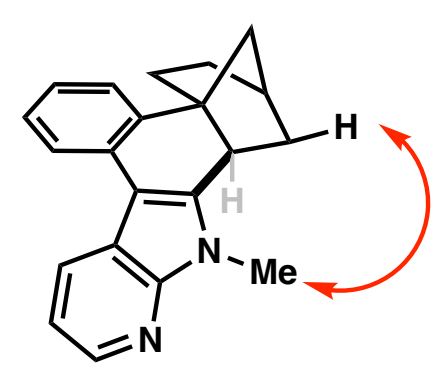

20

Annulation product *iso*-20: R$_f$ 0.25 (9:1 hexanes:EtOAc); ^{1}H NMR (500 MHz, $CDCl_3$) δ 8.36 (dd, J = 7.9, 1.5 Hz, 1H), 8.31 (dd, J = 4.7, 1.5 Hz, 1H), 7.85 (dd, J = 7.8, 1.3 Hz, 1H), 7.26 – 7.23 (overlapped with residual solvent, 1H), 7.18 – 7.11 (m, 3H), 4.03 (s, 3H), 3.18 (t, J = 8.9, 1H), 2.80 – 2.70 (m, 1H), 2.43 (t, J = 4.3 Hz, 1H), 2.35 – 2.29 (m, 1H), 2.07 – 2.00 (m, 1H), 1.94 – 1.84 (m, 1H), 1.71 (dq, J = 9.5, 1.8 Hz, 1H), 1.60 – 1.55 (overlapped with residual water, 3H); ^{13}C NMR (125 MHz, $CDCl_3$) δ 149.8, 142.0, 140.7, 137.7, 131.7, 127.8, 126.8, 126.3, 125.1, 121.8, 117.3, 116.4, 108.5, 47.7, 46.4, 41.5, 40.0, 36.0, 35.5, 31.1, 29.0; IR (film): 2948, 2869, 1599, 1458, 749 cm^{-1}; HRMS-APCI (m/z) $[M + H]^+$ calcd for $C_{21}H_{21}N_2^+$, 301.16993; found 301.16806.

The structure of ***iso*-20** was verified by 2D-NOESY, as the following interaction was observed:

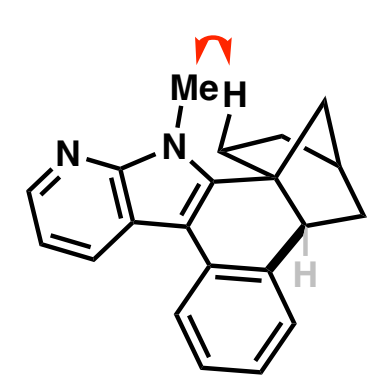

iso-20

Complete assignment and further NOE correlations for compounds **20** and ***iso*-20** are discussed in Section I.C

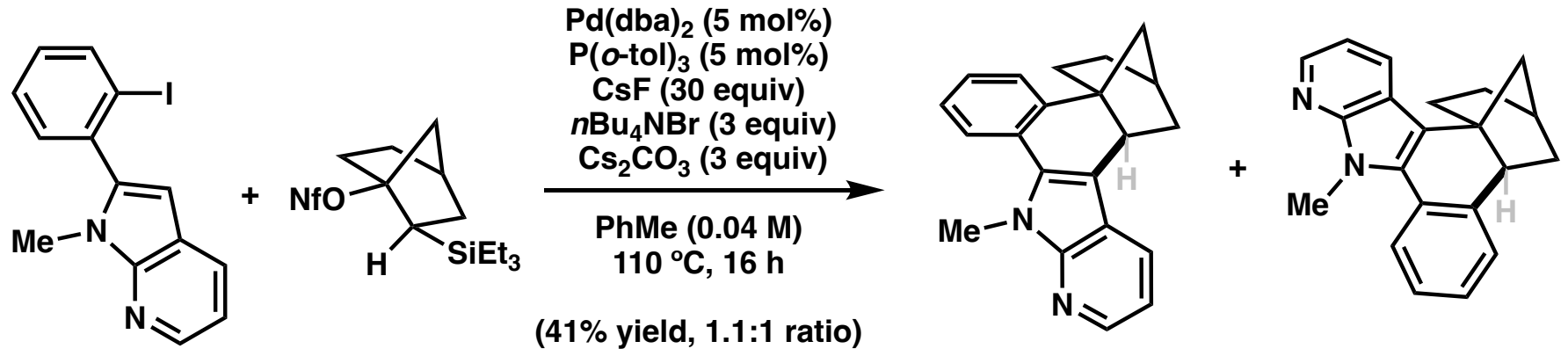

51 **7** **21** ***iso*-21**

Annulation products 21 and *iso*-21. Followed General Procedure B, utilizing **51**. Purification by automated reverse phase flash column chromatography (C18, 20 → 100% MeCN in H_2O) yielded an inseparable mixture of annulation products **21** and ***iso*-21** (41% yield, 1.1:1 ratio by ^{1}H NMR, average of two experiments) as a pale yellow oil. **Annulation product 21**: R_f 0.28 (9:1 hexanes:EtOAc); ^{1}H NMR (500 MHz, $CDCl_3$) δ 8.31 (dd, J = 4.7, 1.5 Hz, 1H), 7.87 – 7.83 (m, 1H), 7.82 (dd, J = 7.8, 1.6 Hz, 1H), 7.65 (dd, J = 7.8, 1.5 Hz, 1H), 7.35 – 7.26 (overlapped with residual solvent, 2H), 7.05 – 7.00 (m, 1H), 4.23 (s, 3H), 3.14 (dd, J = 8.9, 5.0 Hz, 1H), 2.56 – 2.46 (m, 1H), 2.36 – 2.23 (m, 2H), 2.15 – 2.09 (m, 1H), 1.96 – 1.85 (m, 1H), 1.78 (dq, J = 9.8, 2.0 Hz, 1H), 1.55 – 1.48 (m, 2H), 1.28 – 1.25 (m, 1H); **Annulation product *iso*-21**: R_f 0.28 (9:1 hexanes:EtOAc); ^{1}H NMR (500 MHz, $CDCl_3$) δ 8.31 (dd, J = 4.7, 1.5 Hz, 1H), 8.02 (dd, J = 7.9, 1.5 Hz, 1H), 7.90 (dd, J = 7.7, 1.5 Hz, 1H), 7.35 – 7.26 (overlapped with residual solvent, 3H), 7.05 – 7.00 (m, 1H), 4.21 (s, 3H), 3.03 (dd, J = 9.0, 5.7 Hz, 1H), 2.80 – 2.70 (m, 1H), 2.36 – 2.23 (m, 2H), 2.06 – 2.00 (m, 1H), 1.96 – 1.85 (m, 1H), 1.55 – 1.48 (m, 3H), 1.44 (dq, J = 9.9, 2.1 Hz, 1H); **Annulation products 21 and *iso*-21**: ^{13}C NMR (125 MHz, $CDCl_3$, 41 of 42 signals observed) δ 150.5, 150.4, 142.8, 142.6, 142.4, 140.4, 134.5, 132.6, 128.7, 128.21, 128.16, 127.6, 127.5, 127.4, 127.1, 126.5, 126.3, 126.0, 123.2, 122.4, 118.9, 118.7, 115.7, 115.6, 112.7, 50.1, 46.20, 46.19, 44.1, 41.9, 40.5, 39.77, 39.76, 36.9, 36.4, 36.1, 34.3, 32.1, 31.9, 29.8, 29.2; IR (film): 2946, 2869, 1567, 1461, 763 cm^{-1}; HRMS-APCI (m/z) $[M + H]^+$ calcd for $C_{21}H_{21}N_2^+$, 301.16993; found 301.17086.

The structure of **21** and ***iso*-21** were verified by 2D-NOESY of the mixture, as the following interactions were observed:

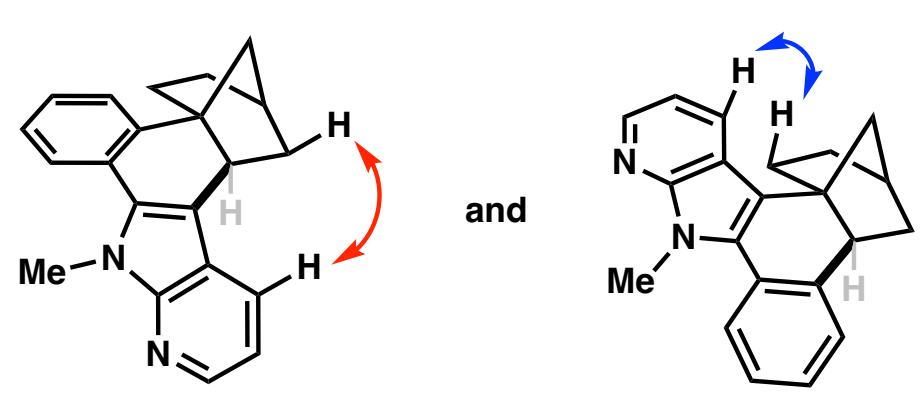

21 ***iso*-21**

C. NMR Analysis of 20 and *iso*-20

As compounds **20** and ***iso*-20** are separable, we were able to perform full 2D NMR analysis on these compounds, including HSQC, HMBC, 2D NOESY and COSY experiments. This has allowed us to assign all proton and carbon NMR signals for both constitutional isomers formed. Below we depict these assignments, in addition to NOESY correlations that were observed. For the latter, key correlations are shown in magenta, and supporting correlations are shown in red. As noted in the earlier sections, 2D NOESY was performed on product mixtures for other compounds, which allowed for the analogous structural assignments to be made.

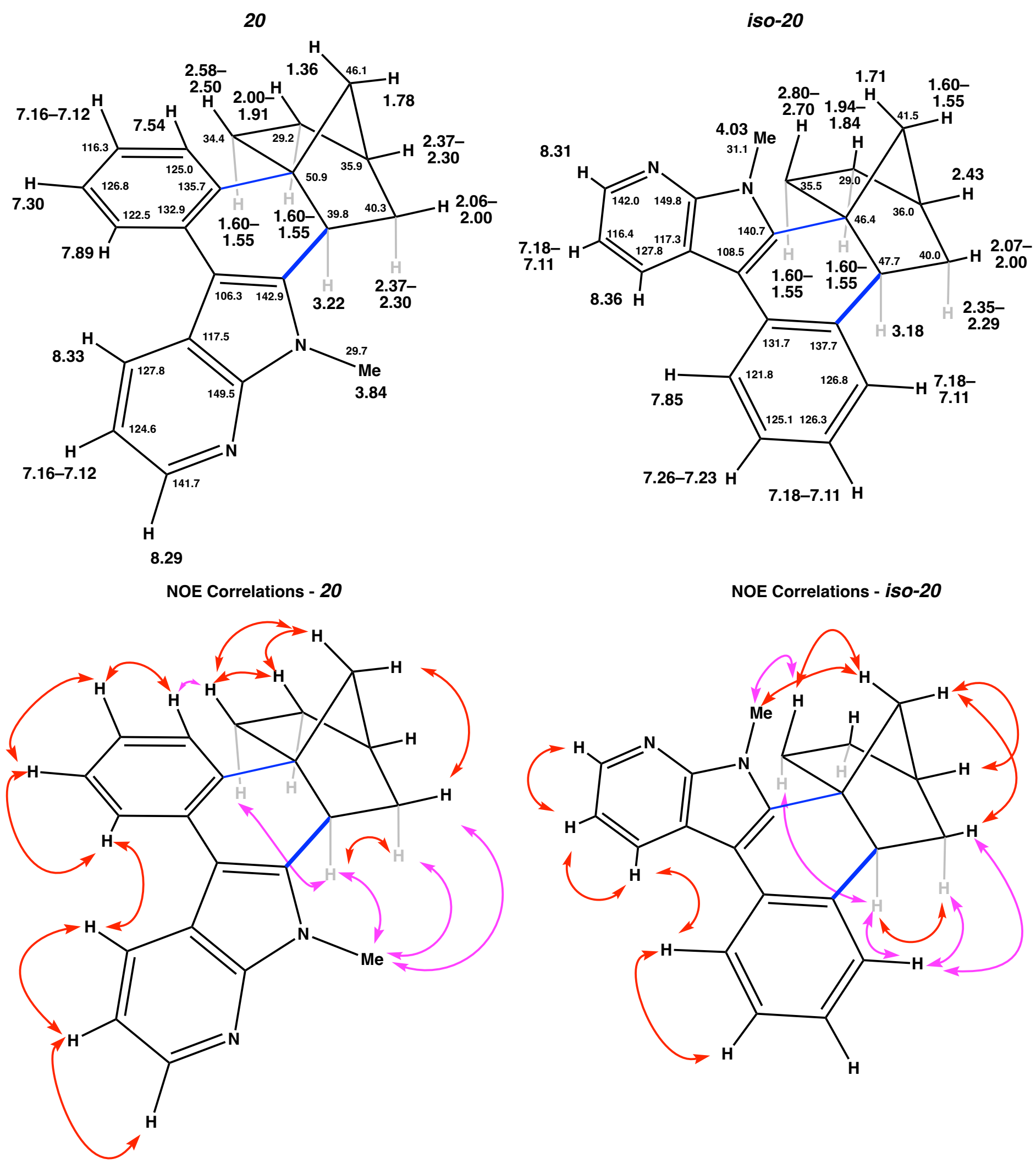

Figure S1. Fully assigned NMR signals and key NOE correlations for **20** and ***iso*-20**.

NMR Spectra

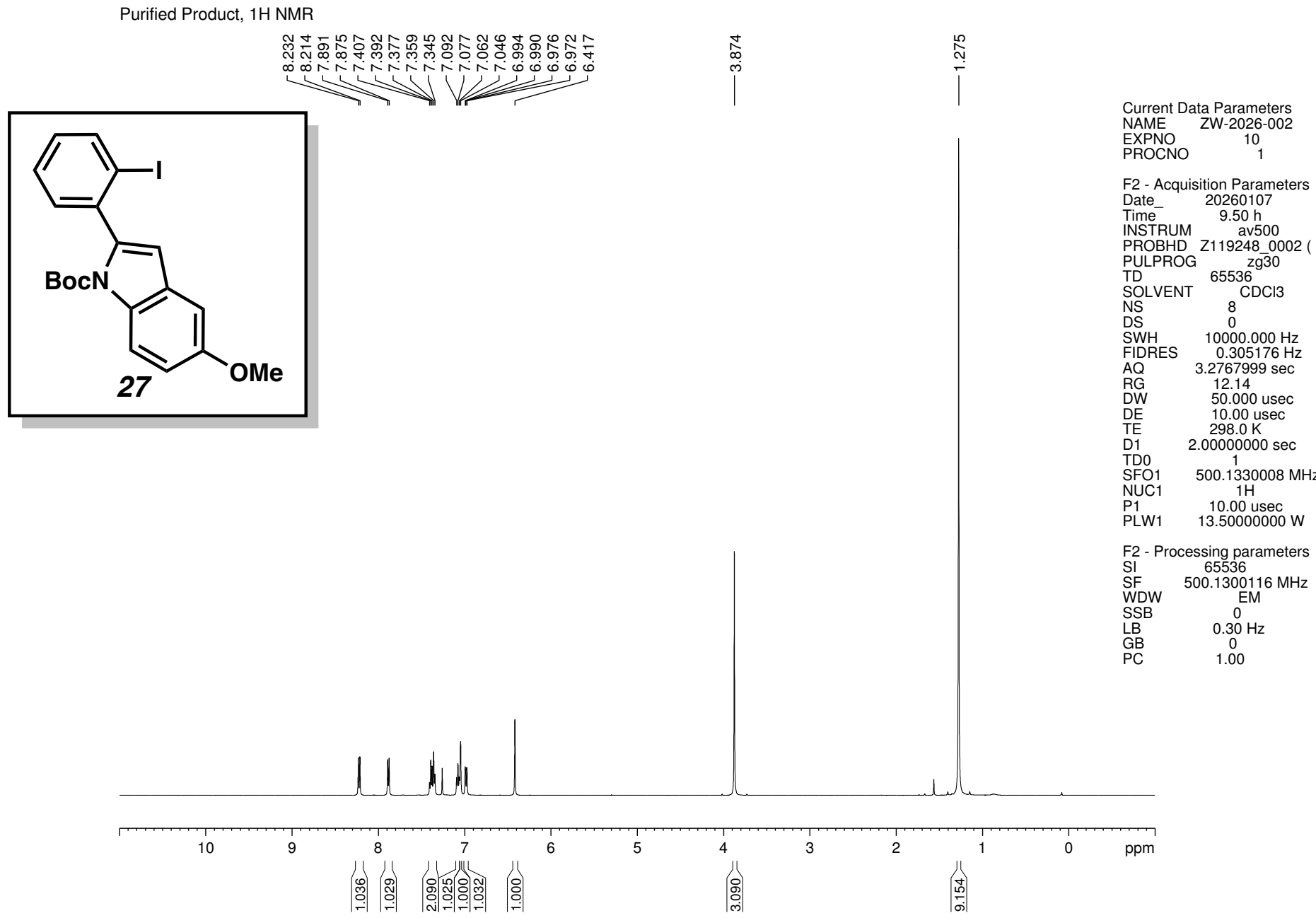

Figure S2. ^{1}H NMR (500 MHz, $CDCl_3$) of compound **27**.

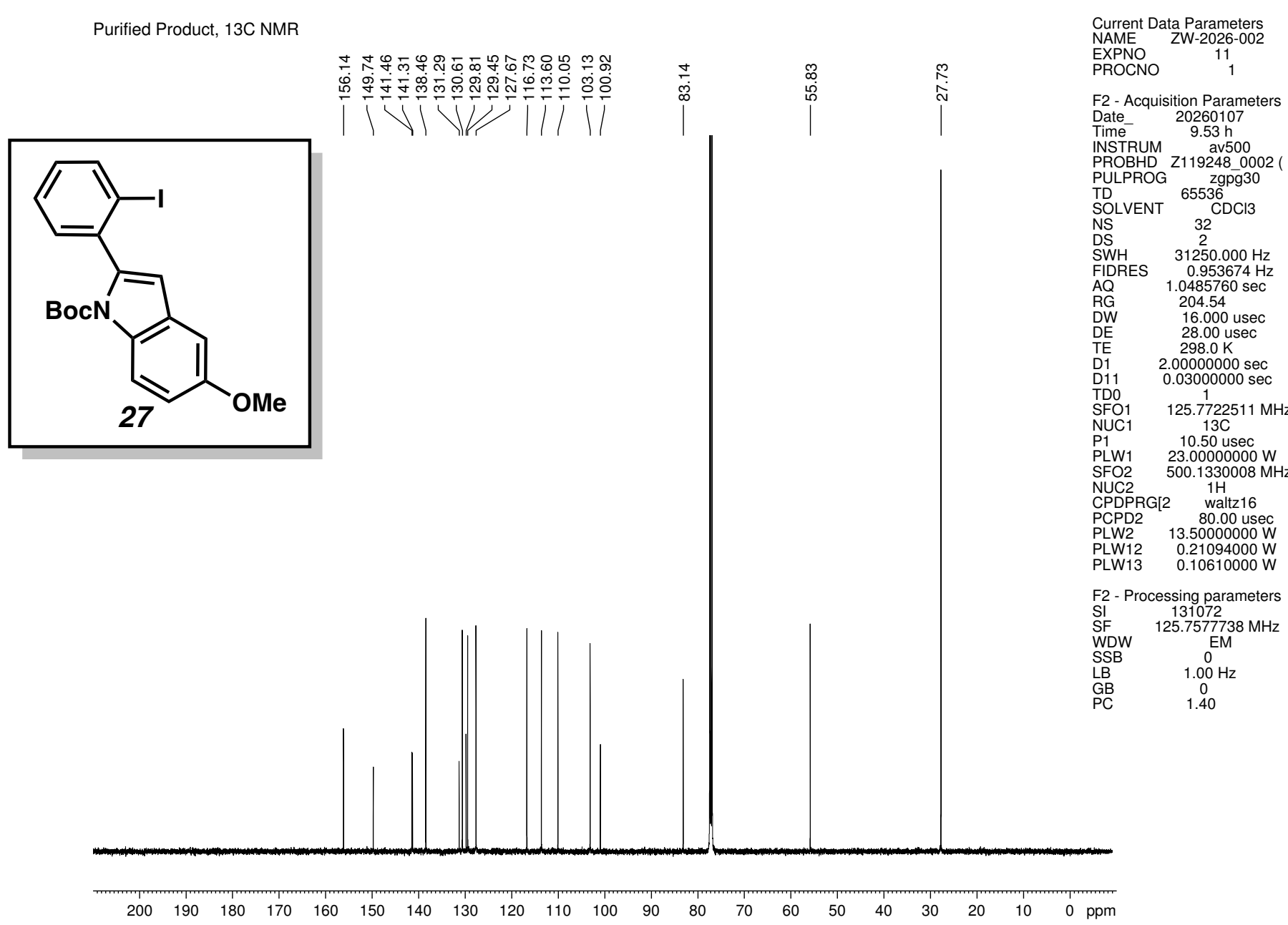

Figure S3. ^{13}C NMR (125 MHz, $CDCl_3$) of compound **27**.

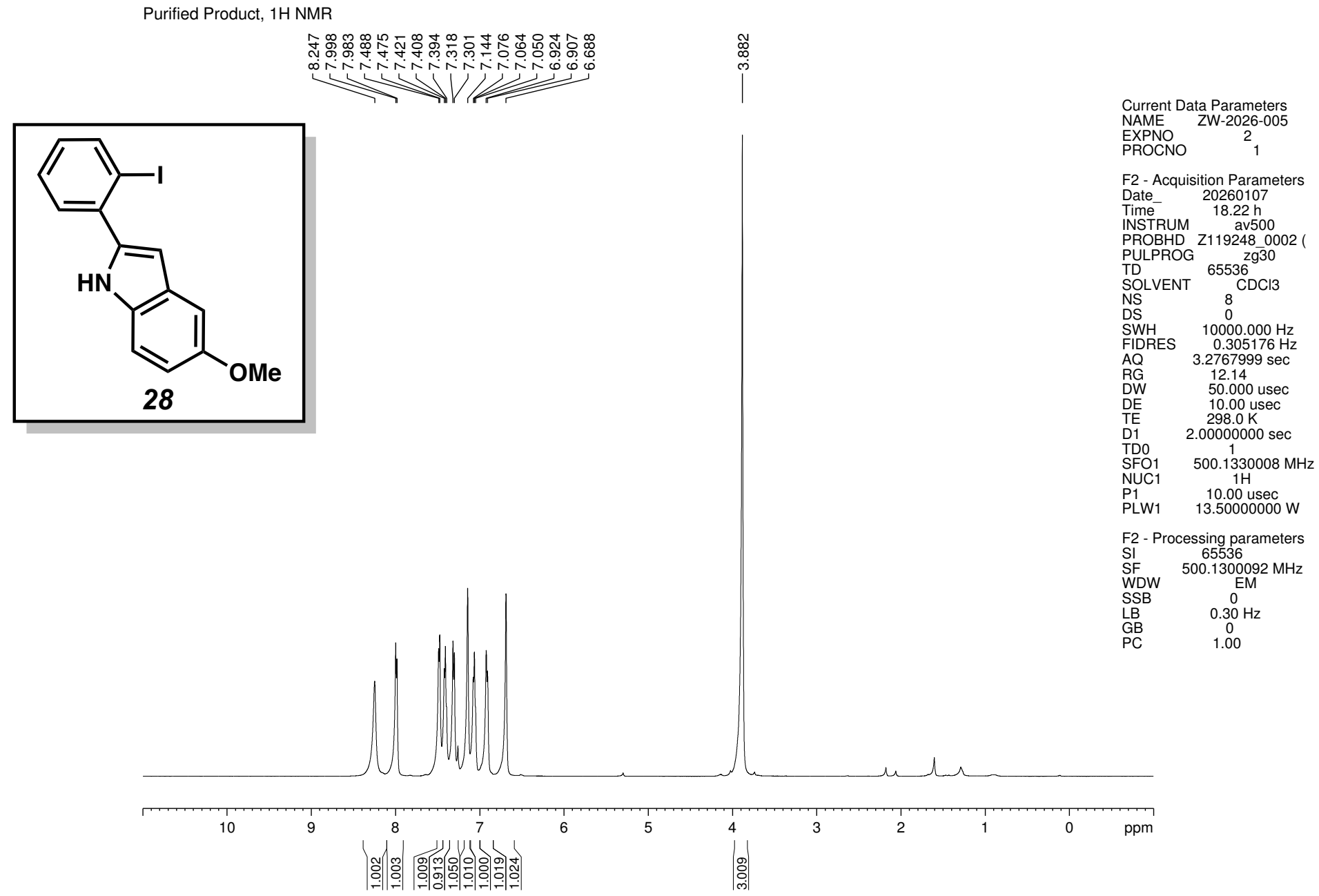

Figure S4. ^{1}H NMR (500 MHz, $CDCl_3$) of compound **28**.

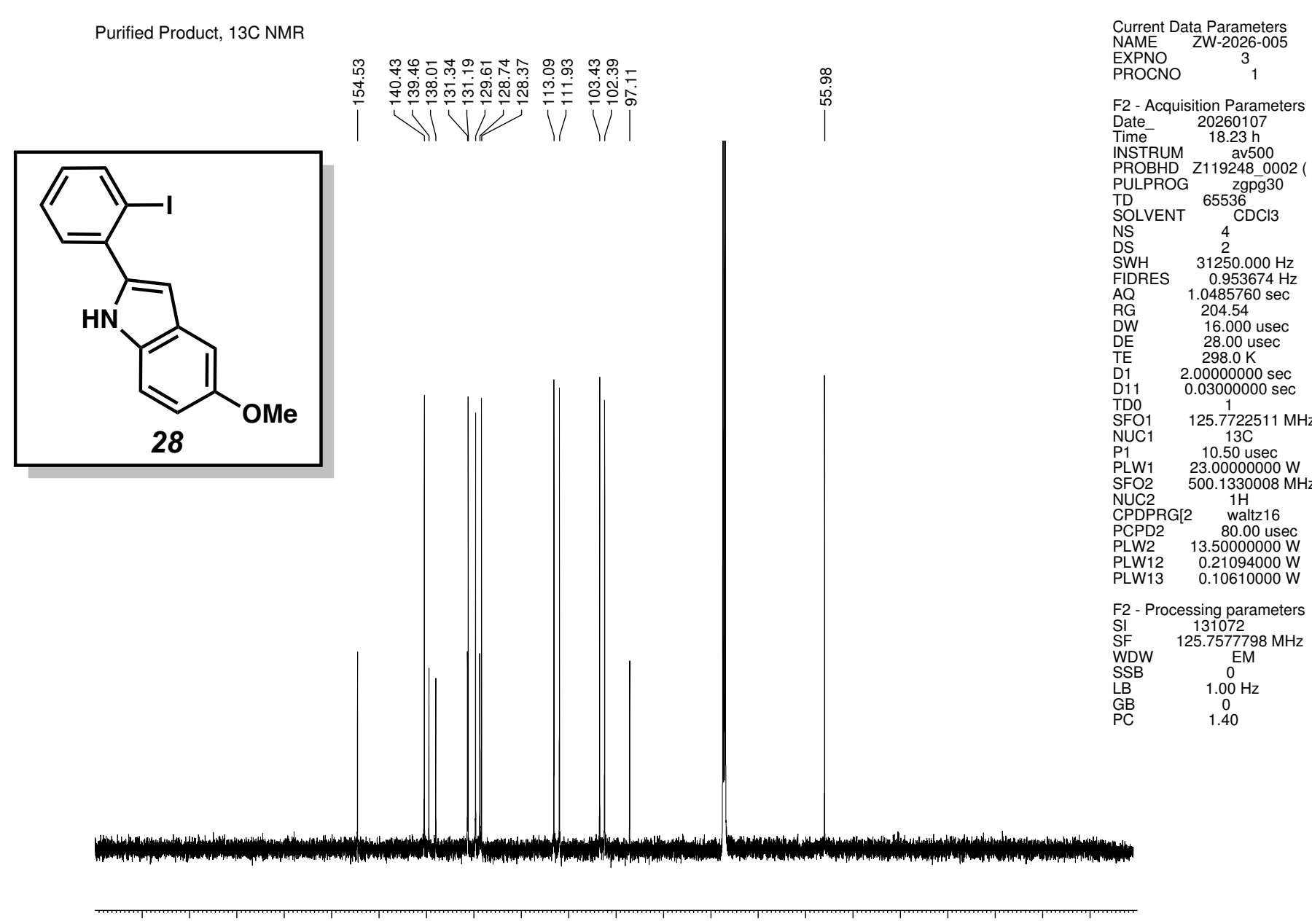

Figure S5. ^{13}C NMR (125 MHz, $CDCl_3$) of compound **28**.

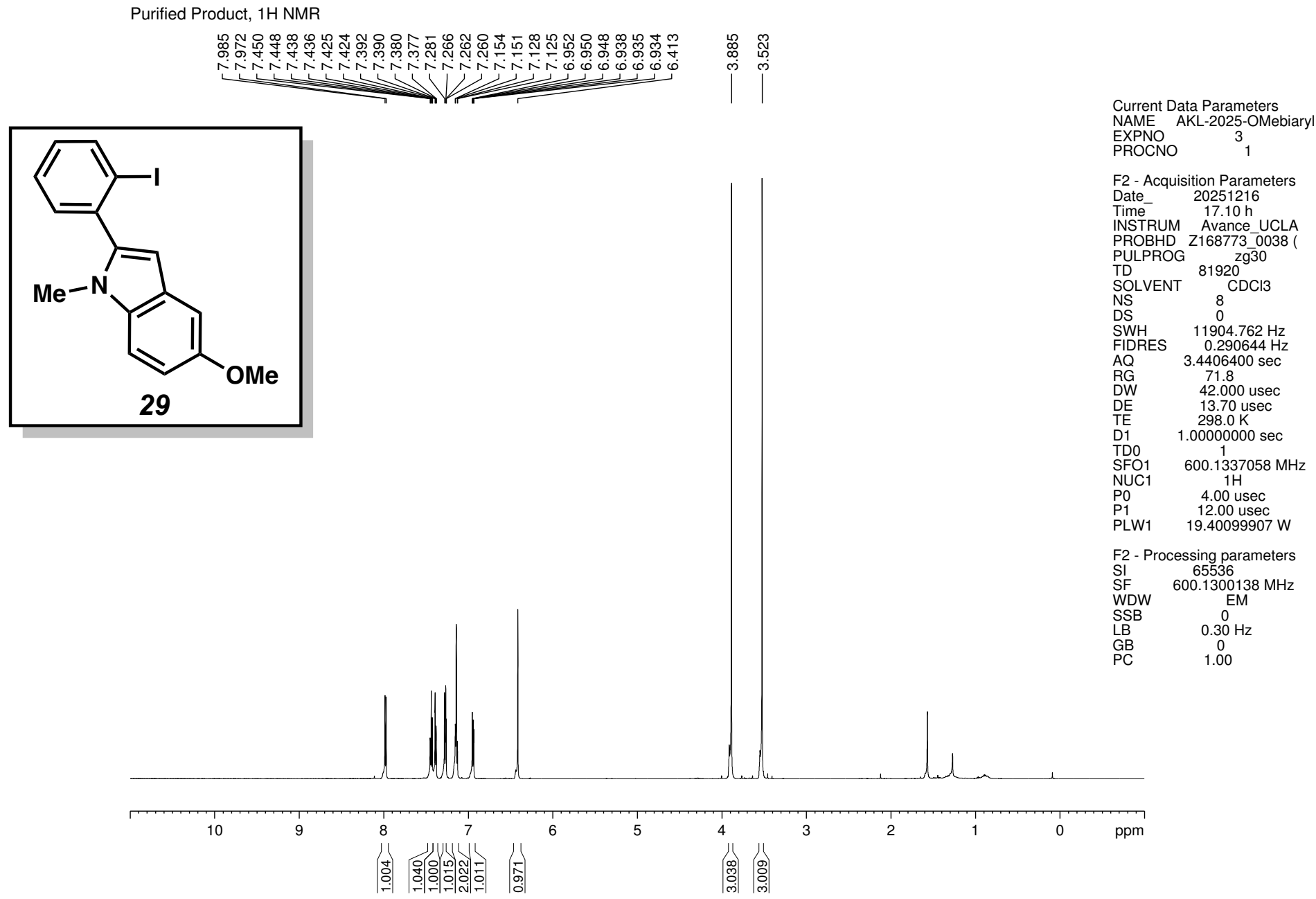

Figure S6. ^{1}H NMR (600 MHz, $CDCl_3$) of compound **29**.

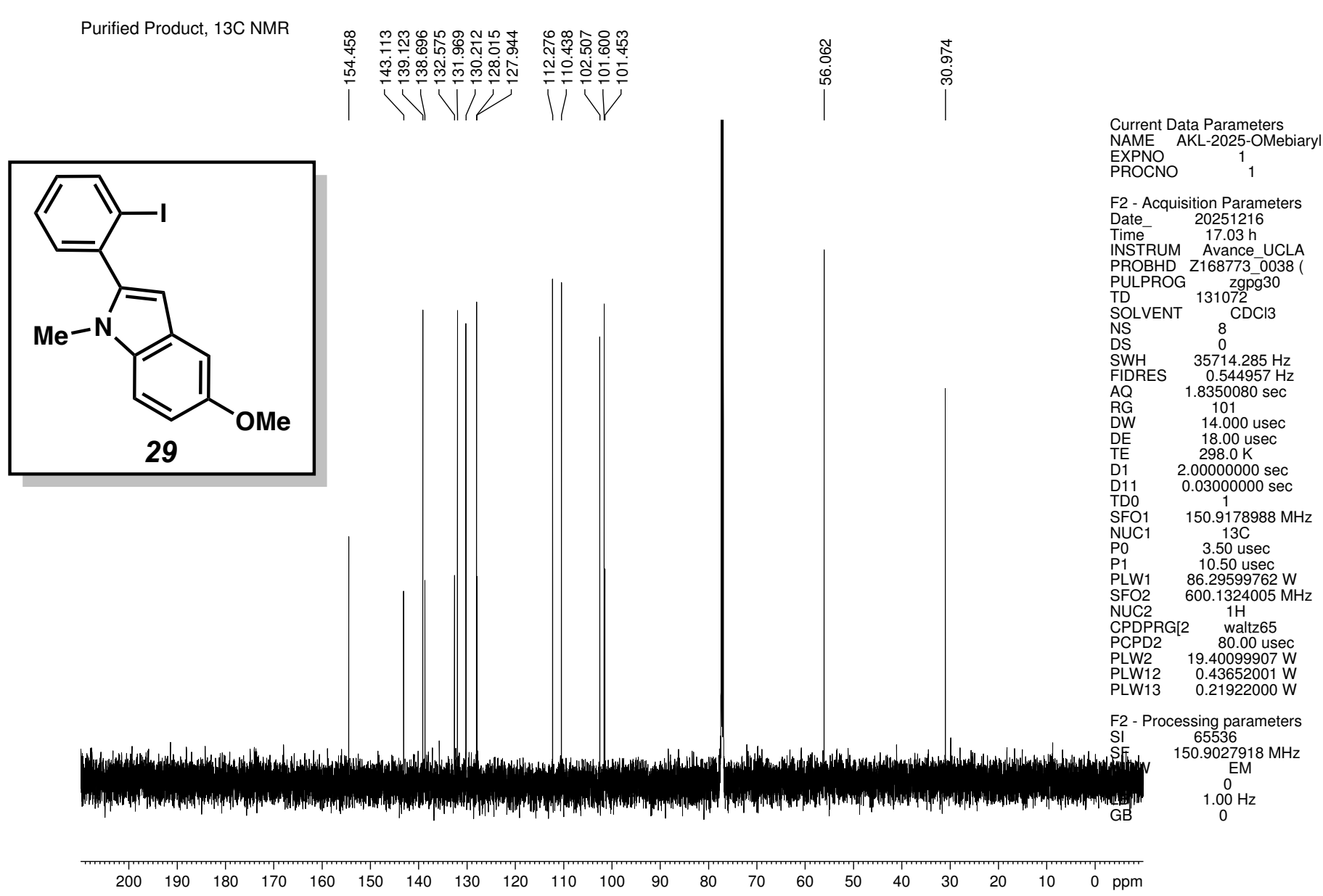

Figure S7. ^{13}C NMR (150 MHz, $CDCl_3$) of compound **29**.

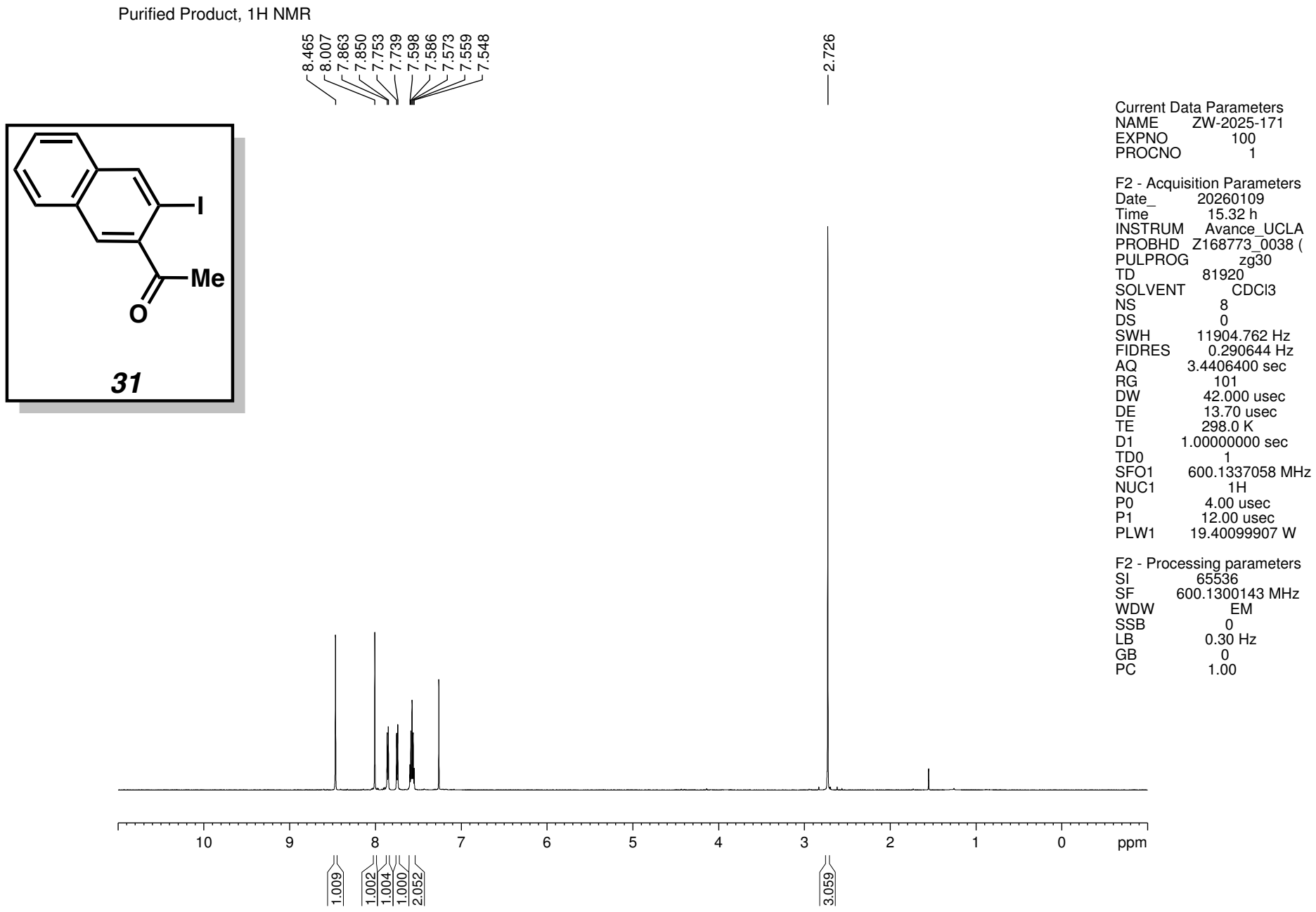

Figure S8. ^{1}H NMR (600 MHz, $CDCl_3$) of compound **31**.

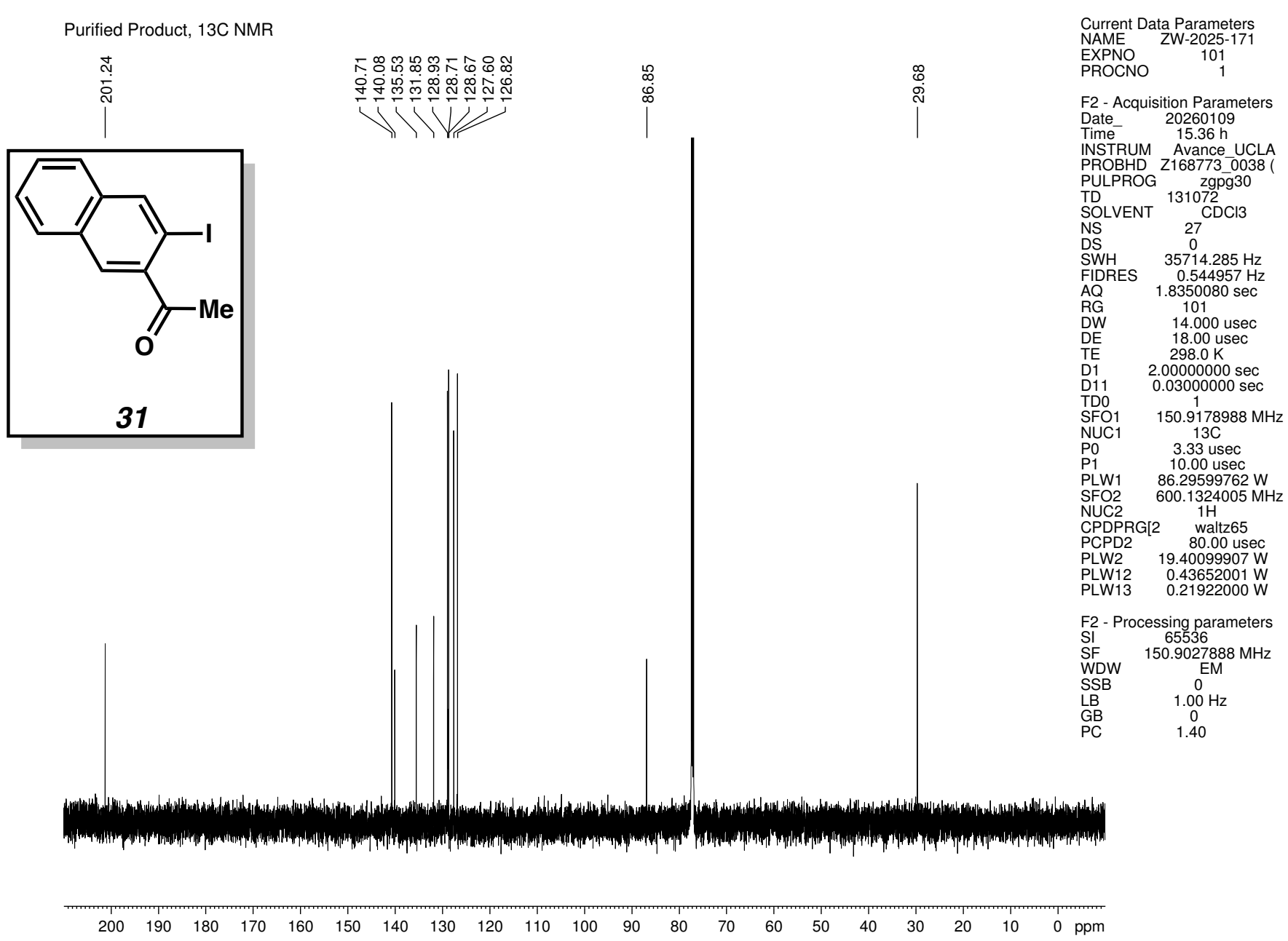

Figure S9. ^{13}C NMR (150 MHz, $CDCl_3$) of compound **31**.

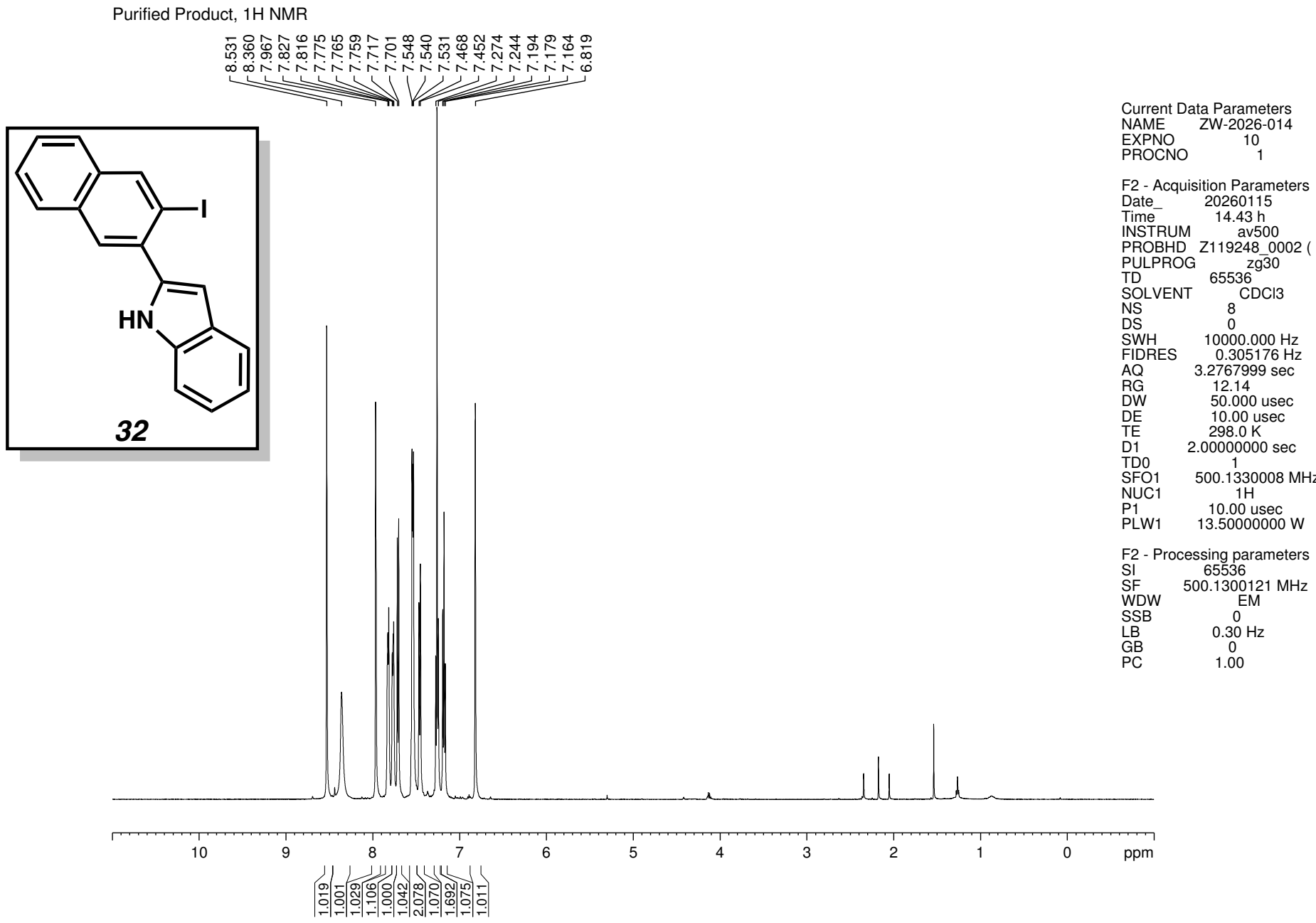

Figure S10. ^{1}H NMR (500 MHz, $CDCl_3$) of compound **32**.

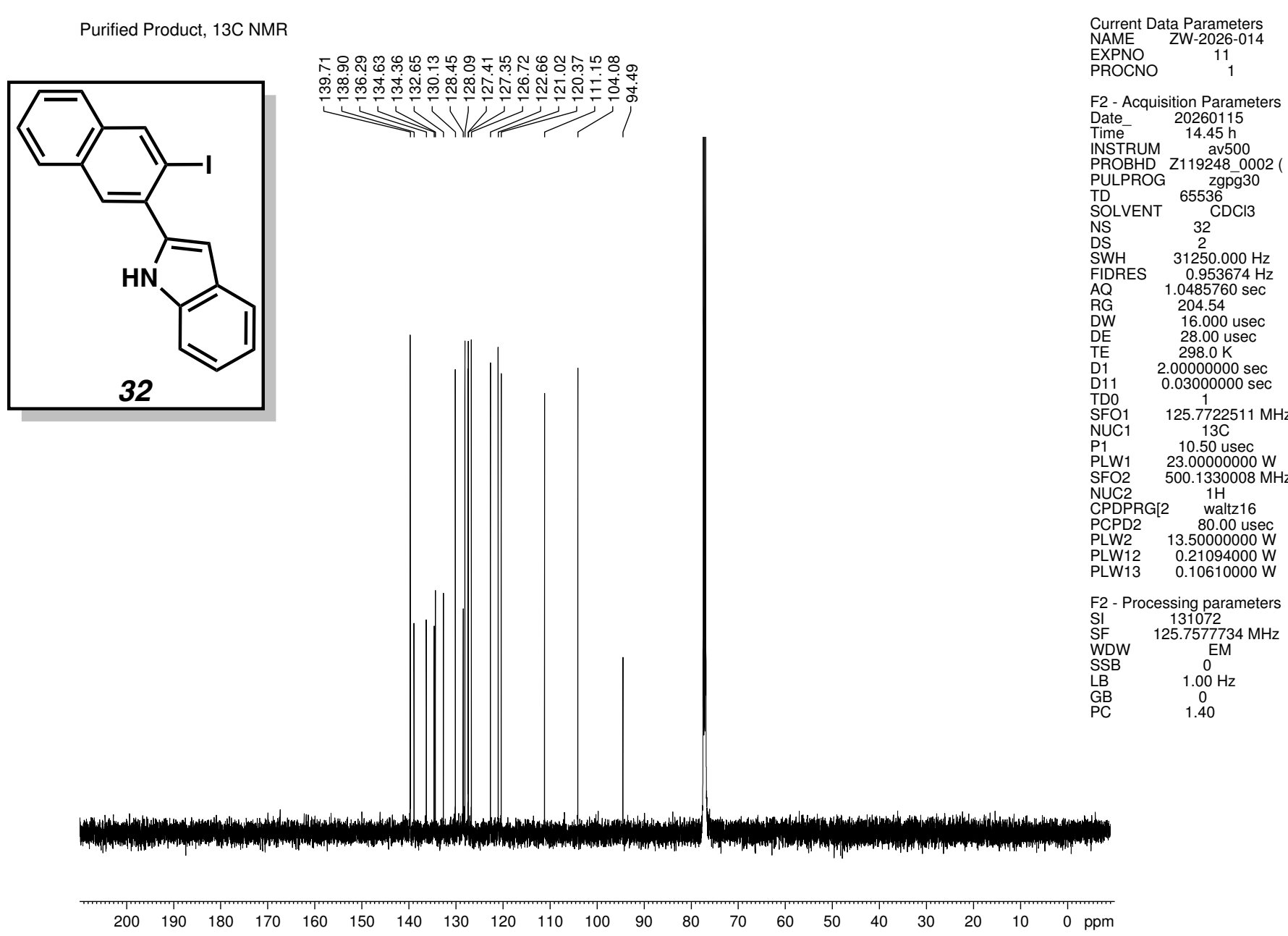

Figure S11. ^{13}C NMR (125 MHz, $CDCl_3$) of compound **32**.

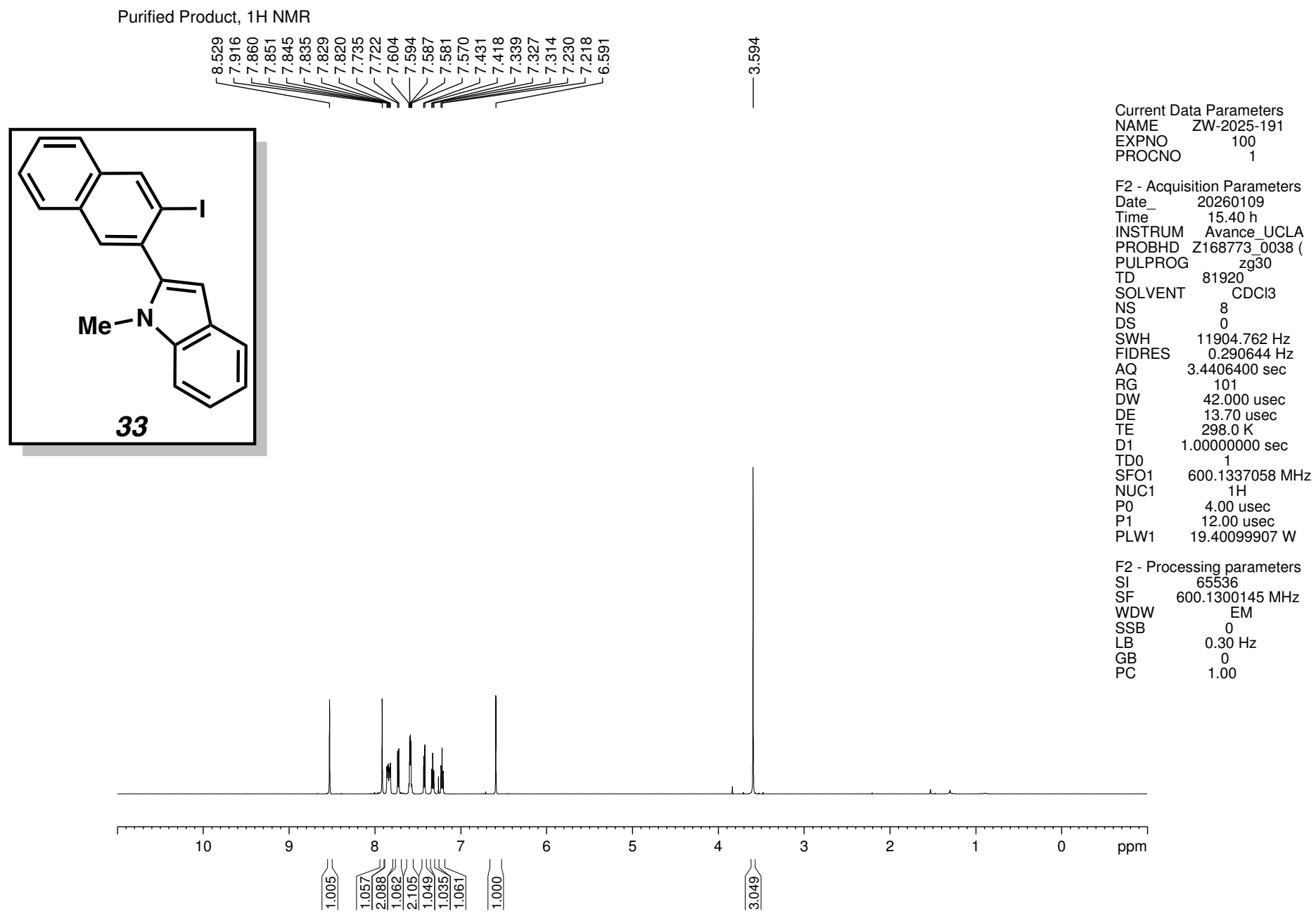

Figure S12. ^{1}H NMR (600 MHz, $CDCl_3$) of compound **33**.

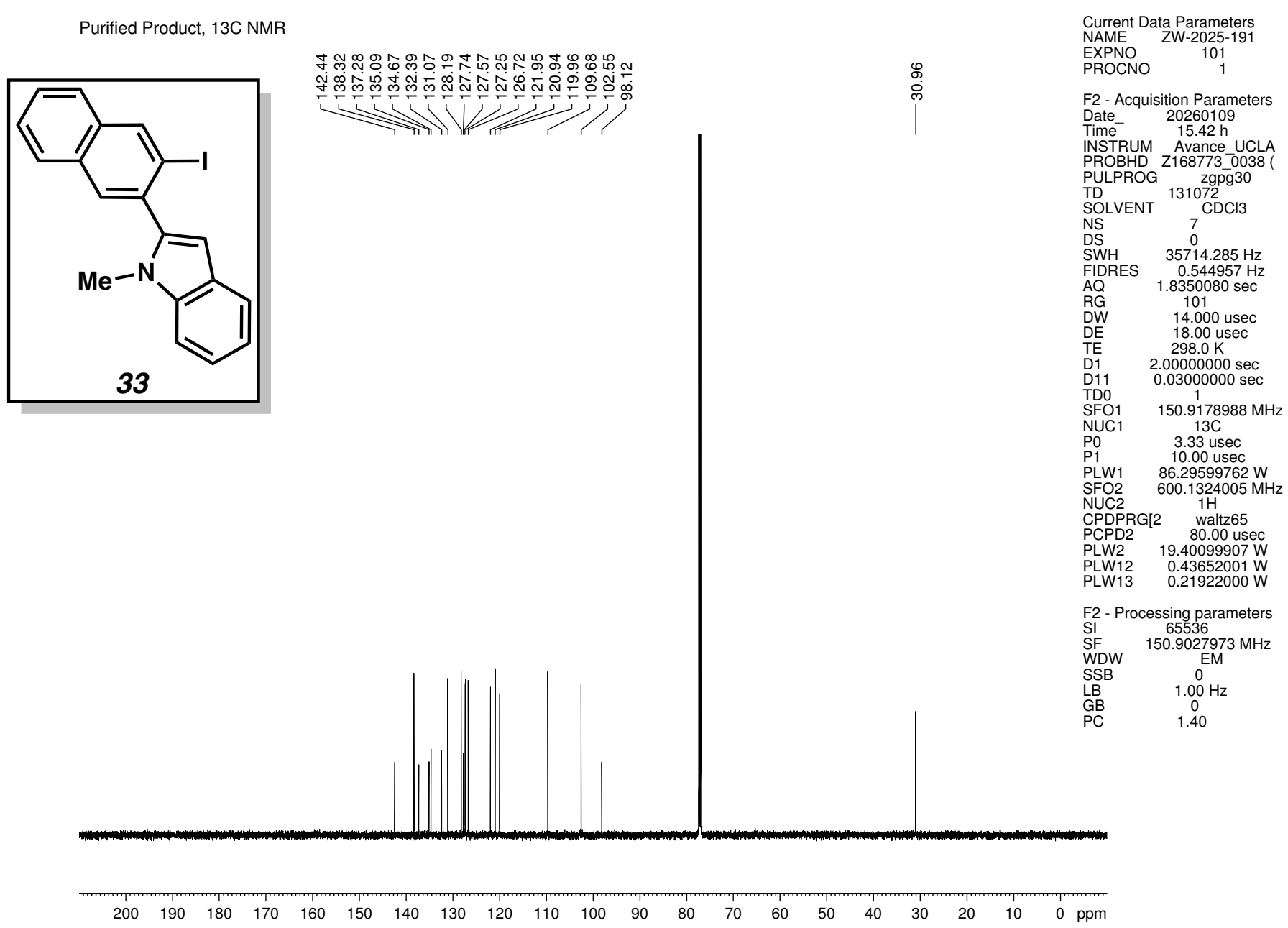

Figure S13. ^{13}C NMR (150 MHz, $CDCl_3$) of compound **33**.

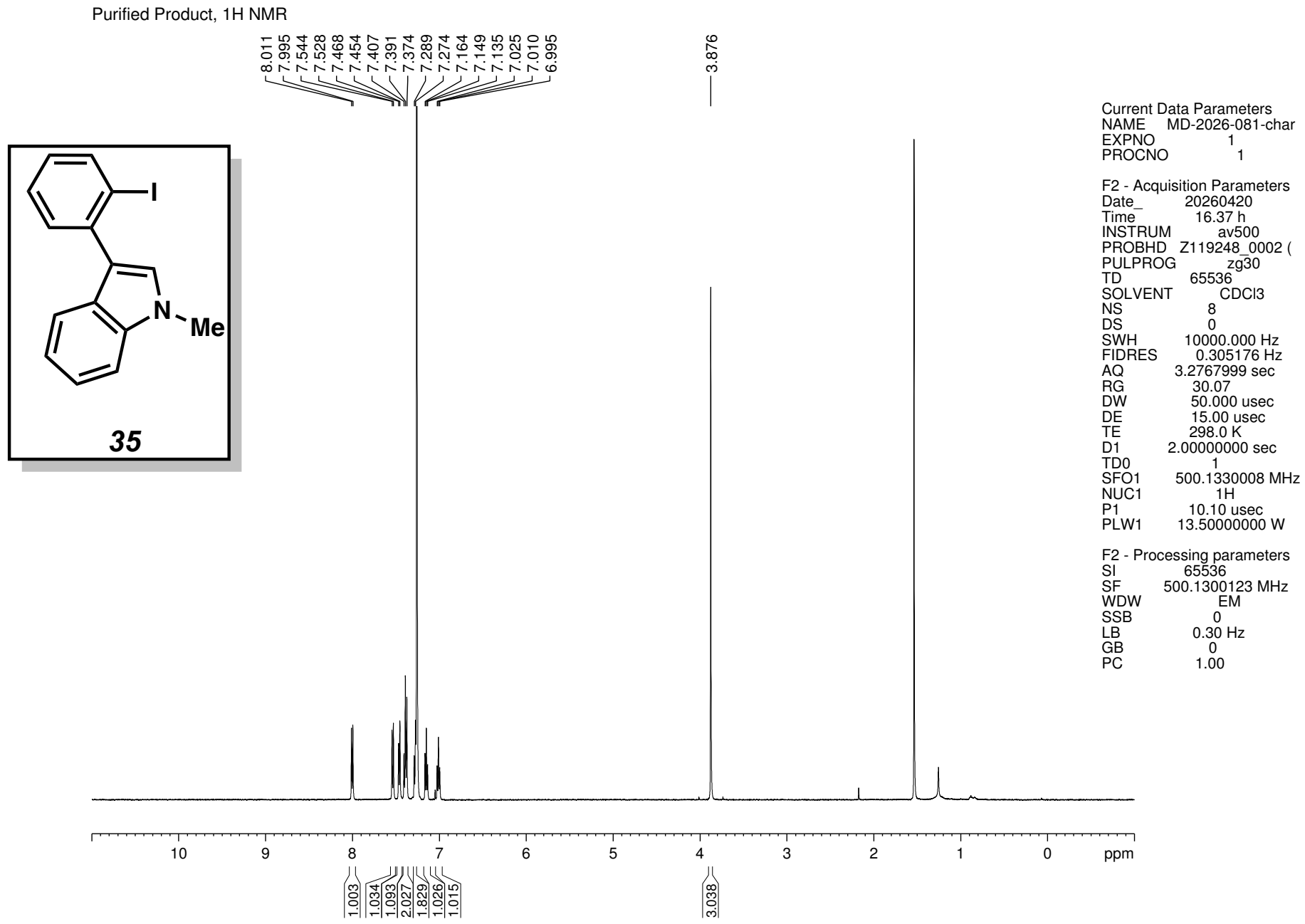

Figure S14. ^{1}H NMR (600 MHz, $CDCl_3$) of compound **35.**

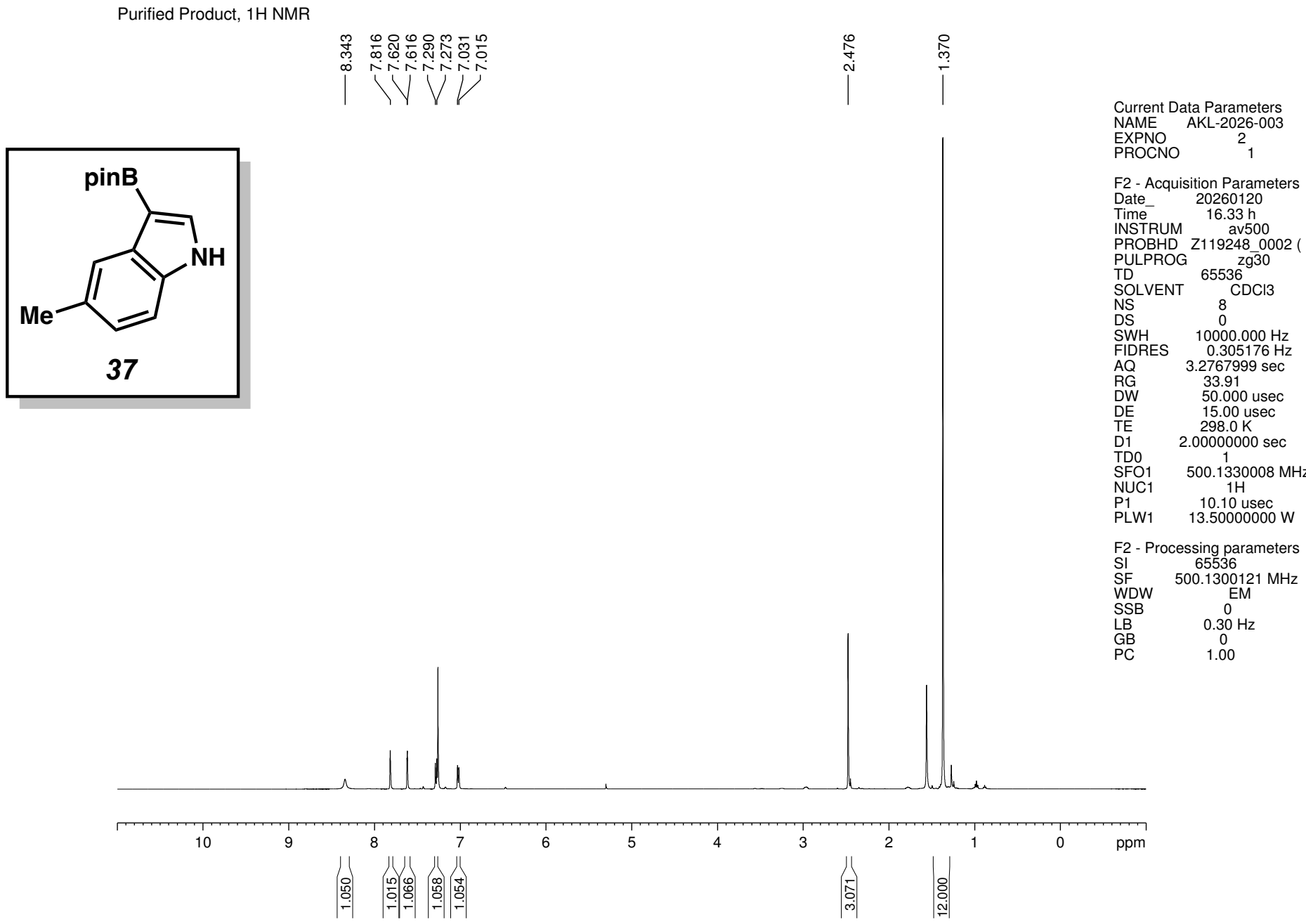

Figure S15. ^{1}H NMR (600 MHz, $CDCl_3$) of compound **37**.

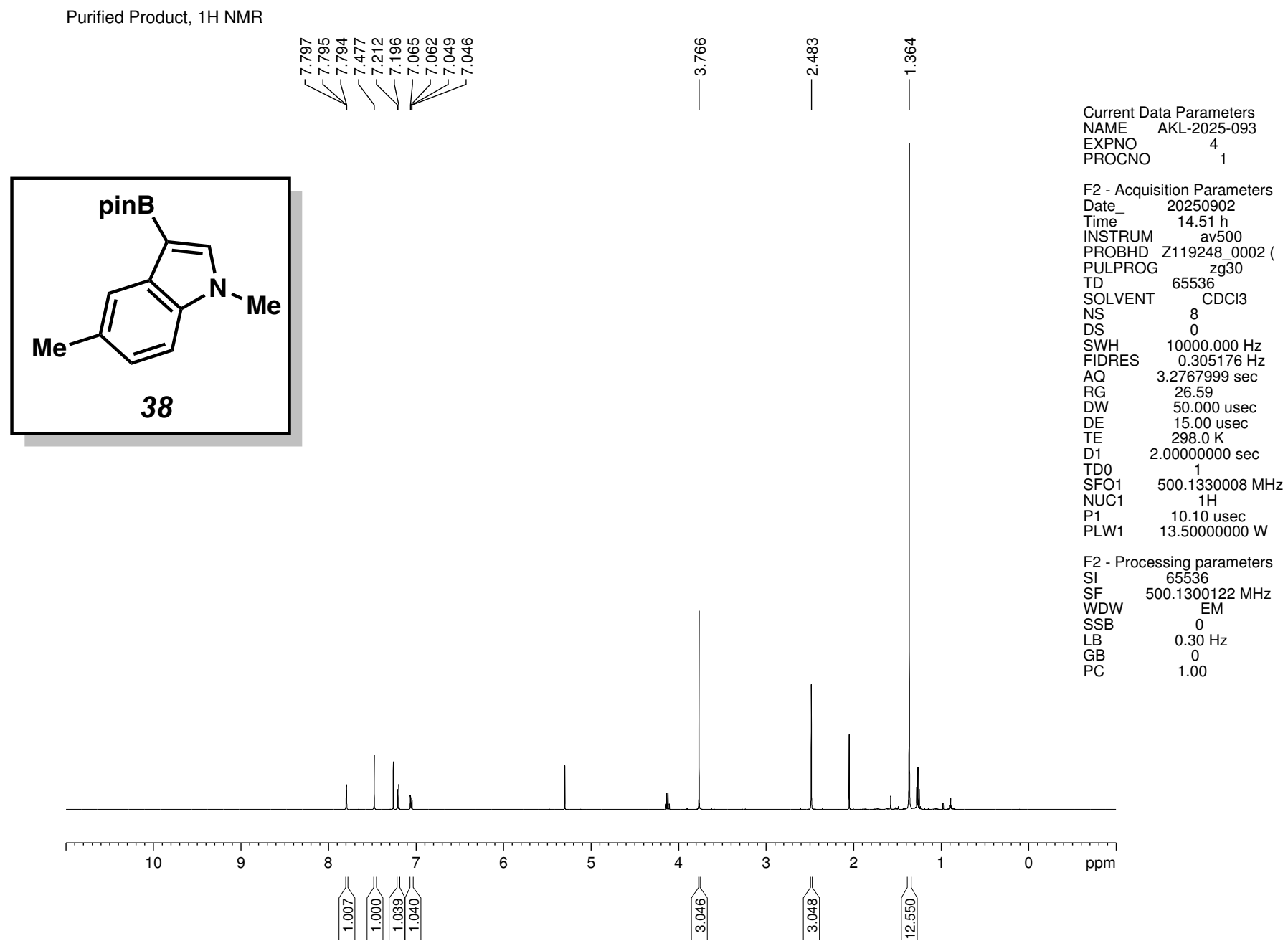

Figure S16. ^{1}H NMR (500 MHz, $CDCl_3$) of compound **38**.

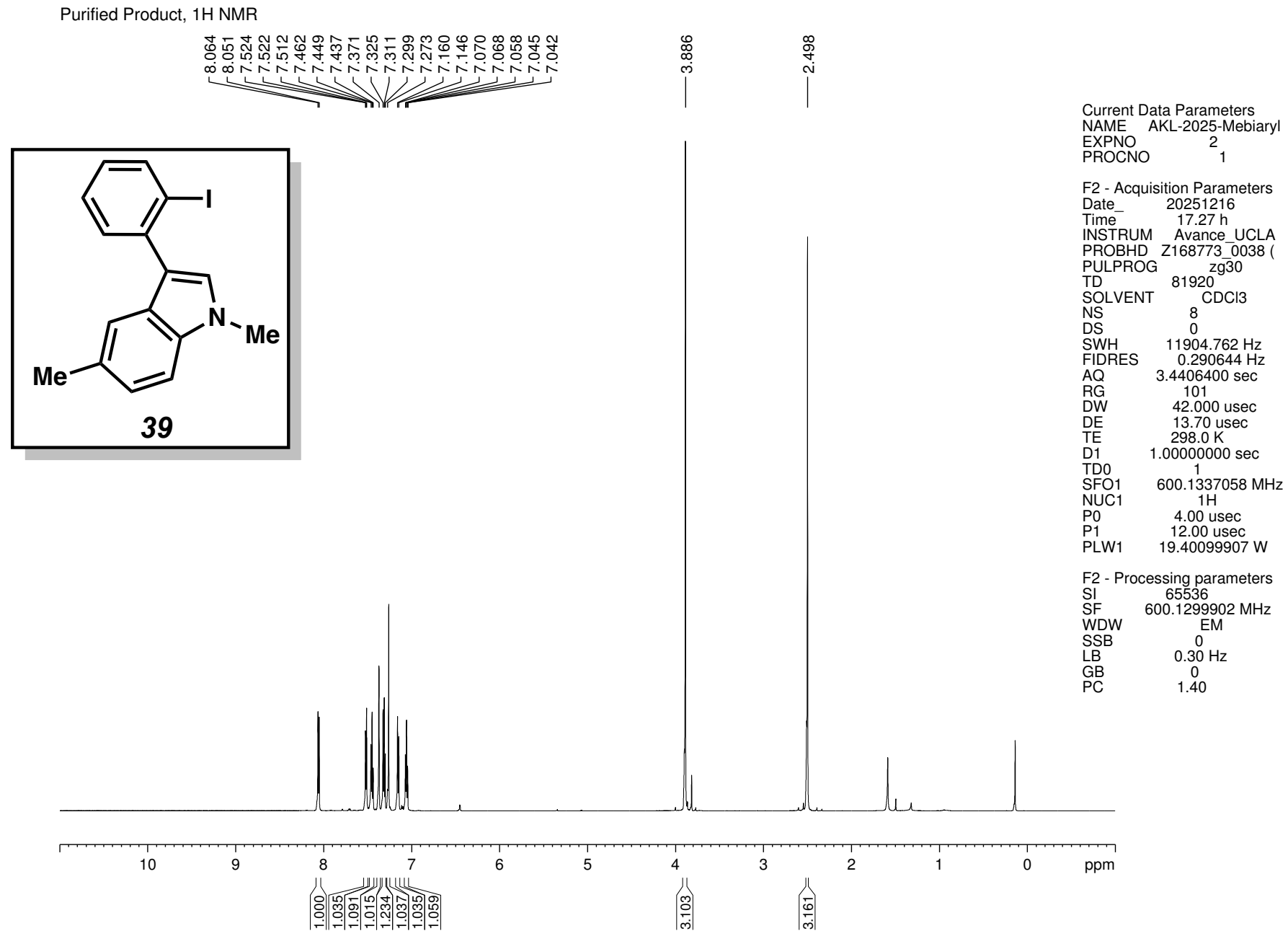

Figure S17. ^{1}H NMR (600 MHz, $CDCl_3$) of compound **39.**

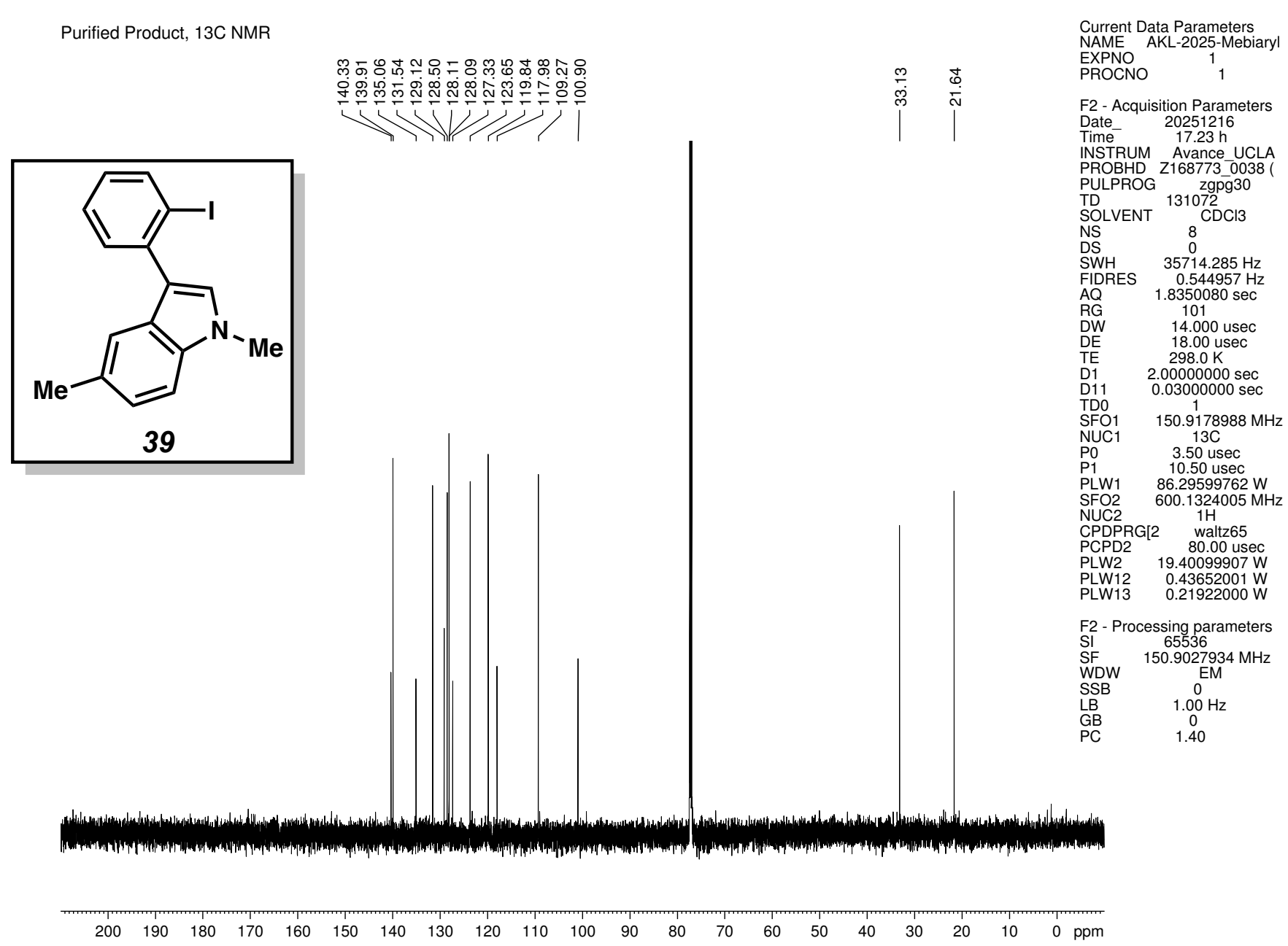

Figure S18. ^{13}C NMR (150 MHz, $CDCl_3$) of compound **39.**

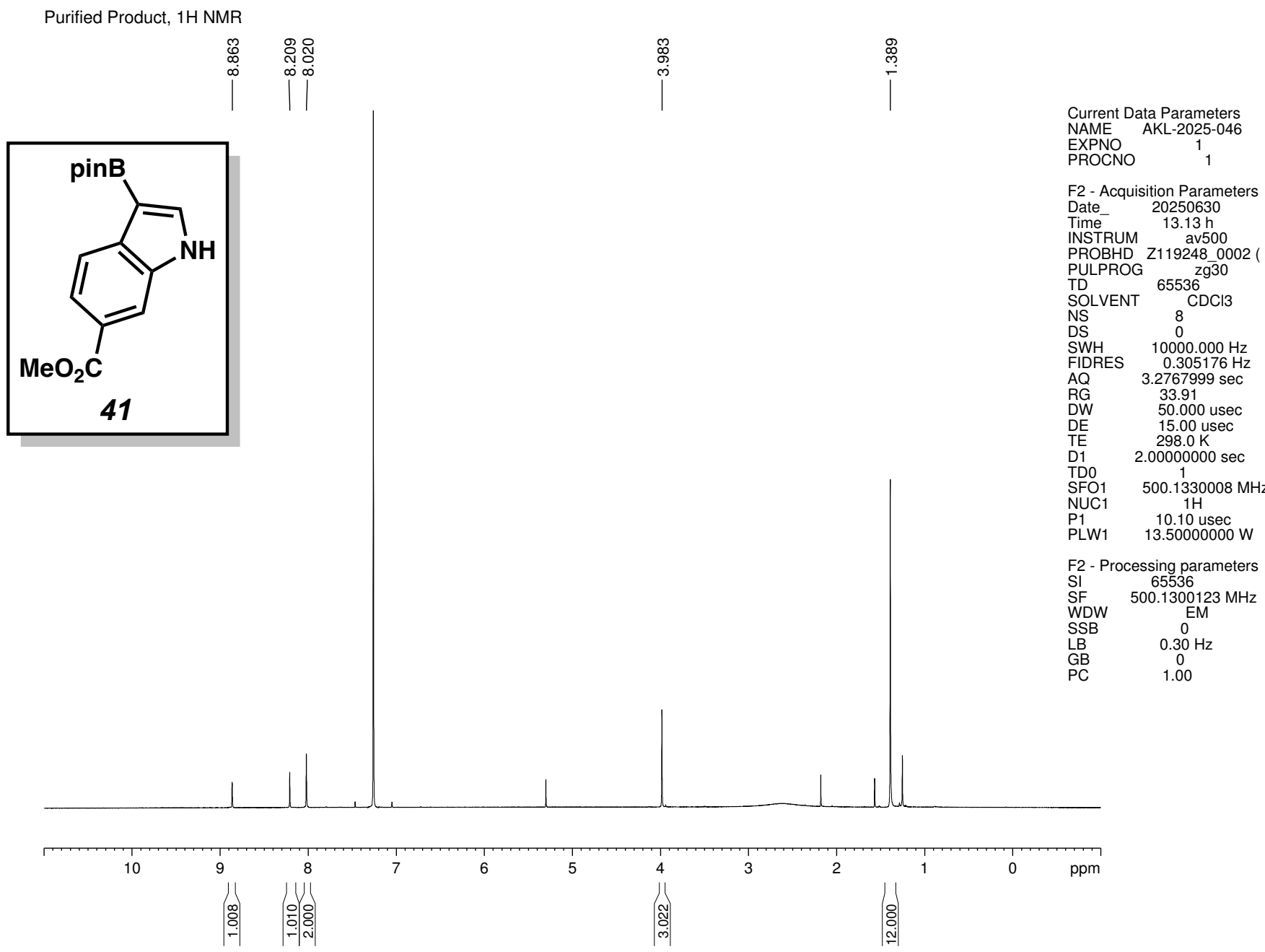

Figure S19. ^{1}H NMR (500 MHz, $CDCl_3$) of compound **41.**

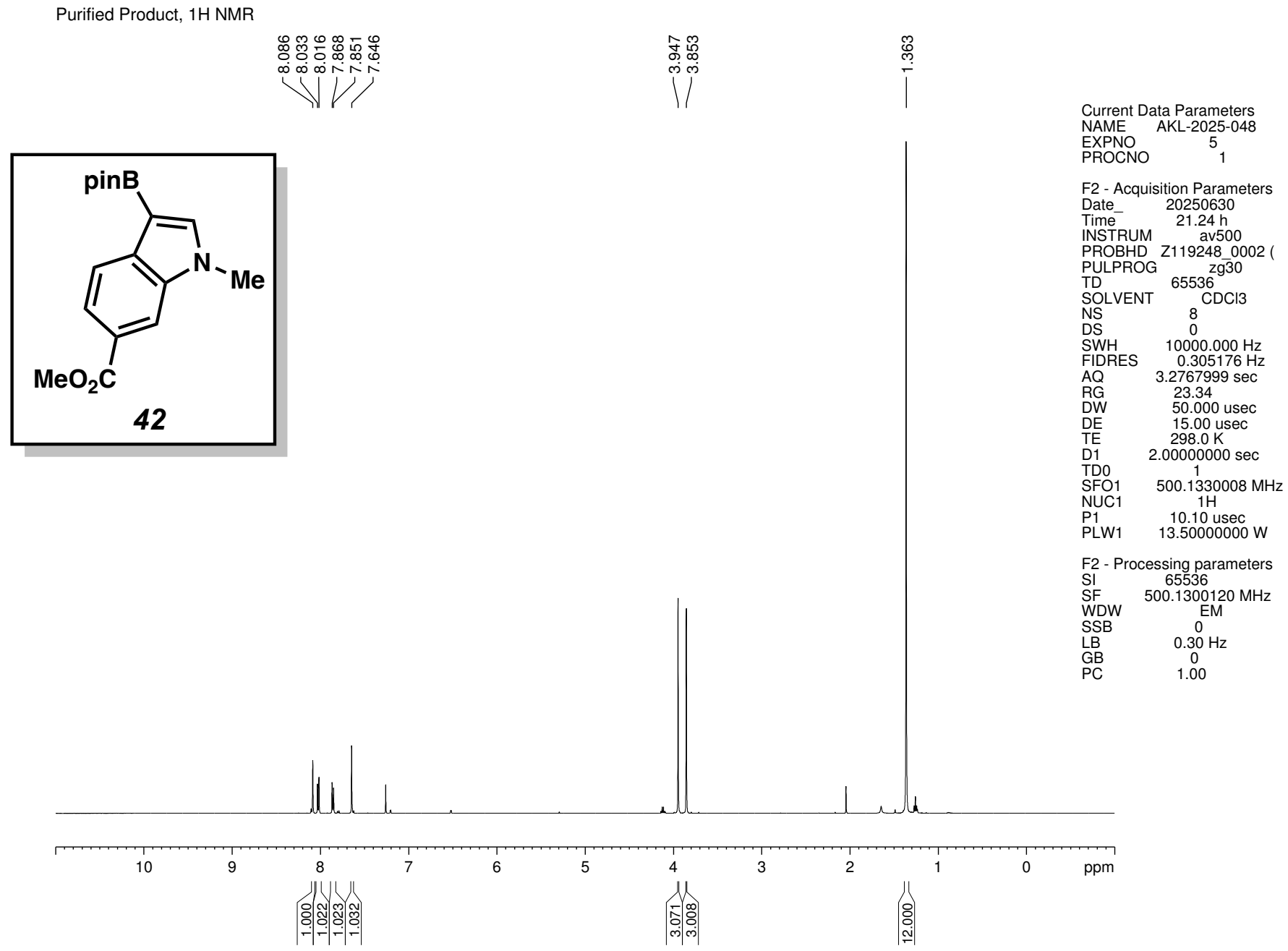

Figure S20. ^{1}H NMR (500 MHz, $CDCl_3$) of compound **42.**

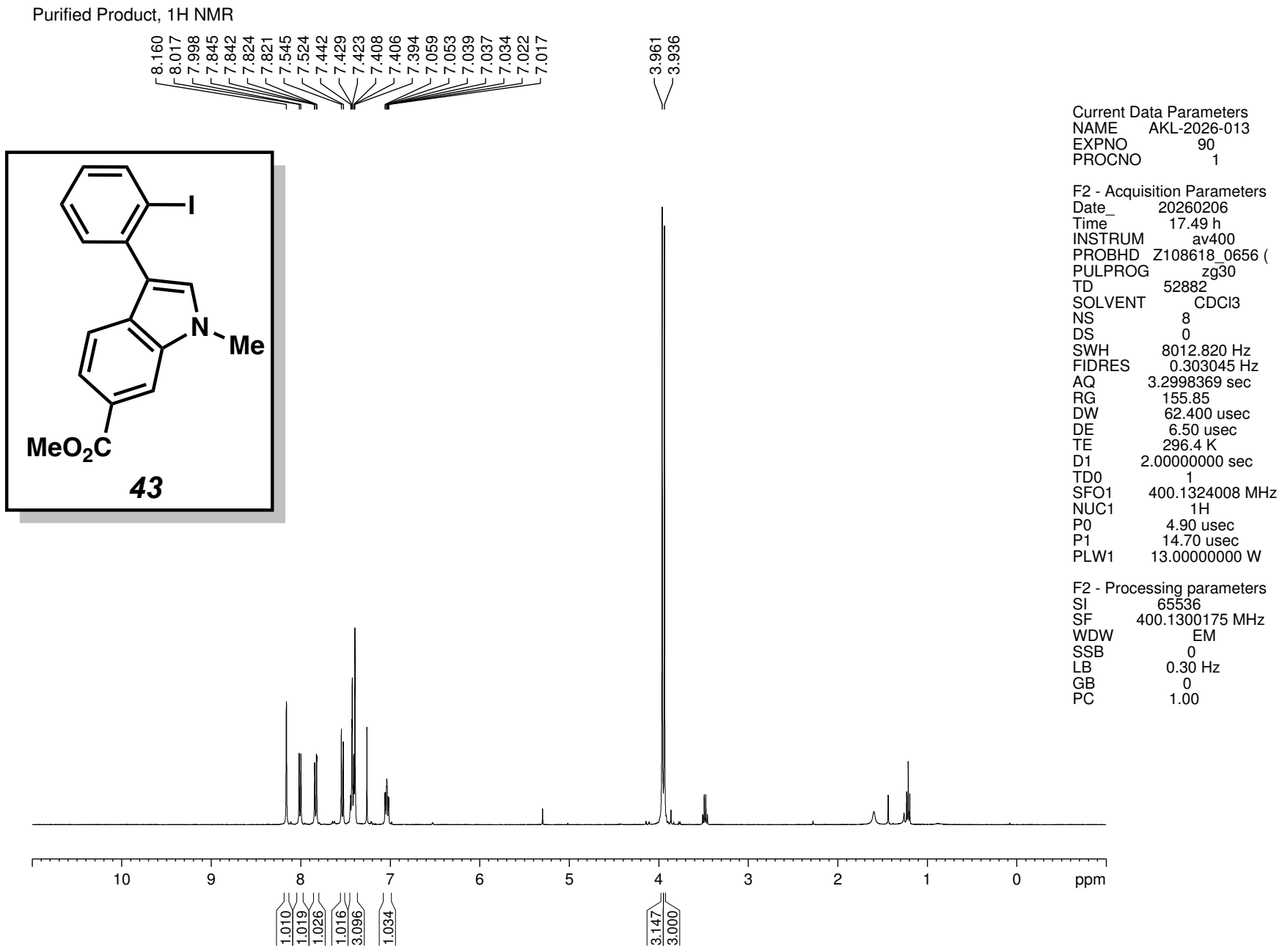

Figure S21. ^{1}H NMR (400 MHz, $CDCl_3$) of compound **43.**

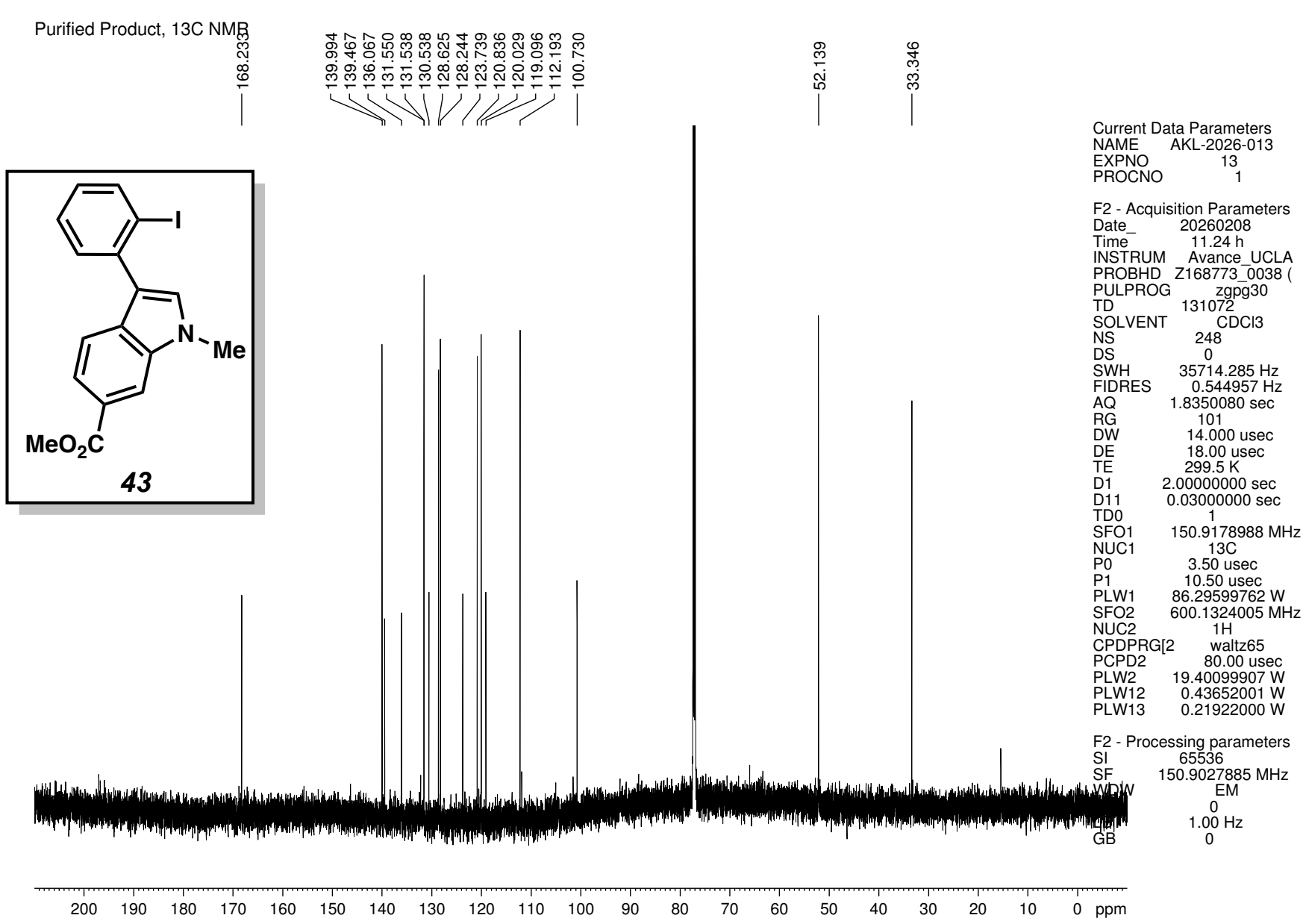

Figure S22. ^{13}C NMR (150 MHz, $CDCl_3$) of compound **43.**

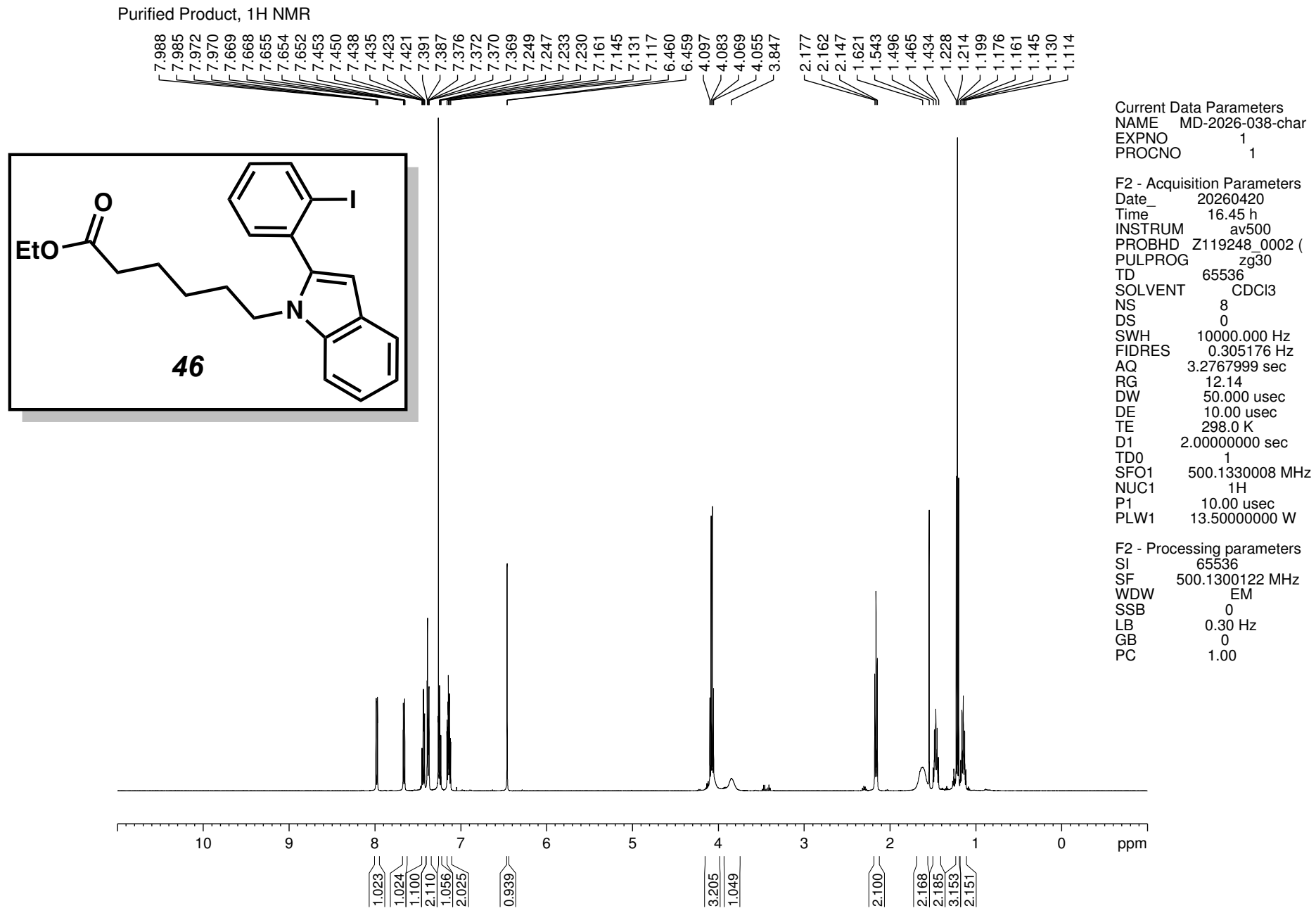

Figure S23. ^{1}H NMR (500 MHz, $CDCl_3$) of compound **46.**

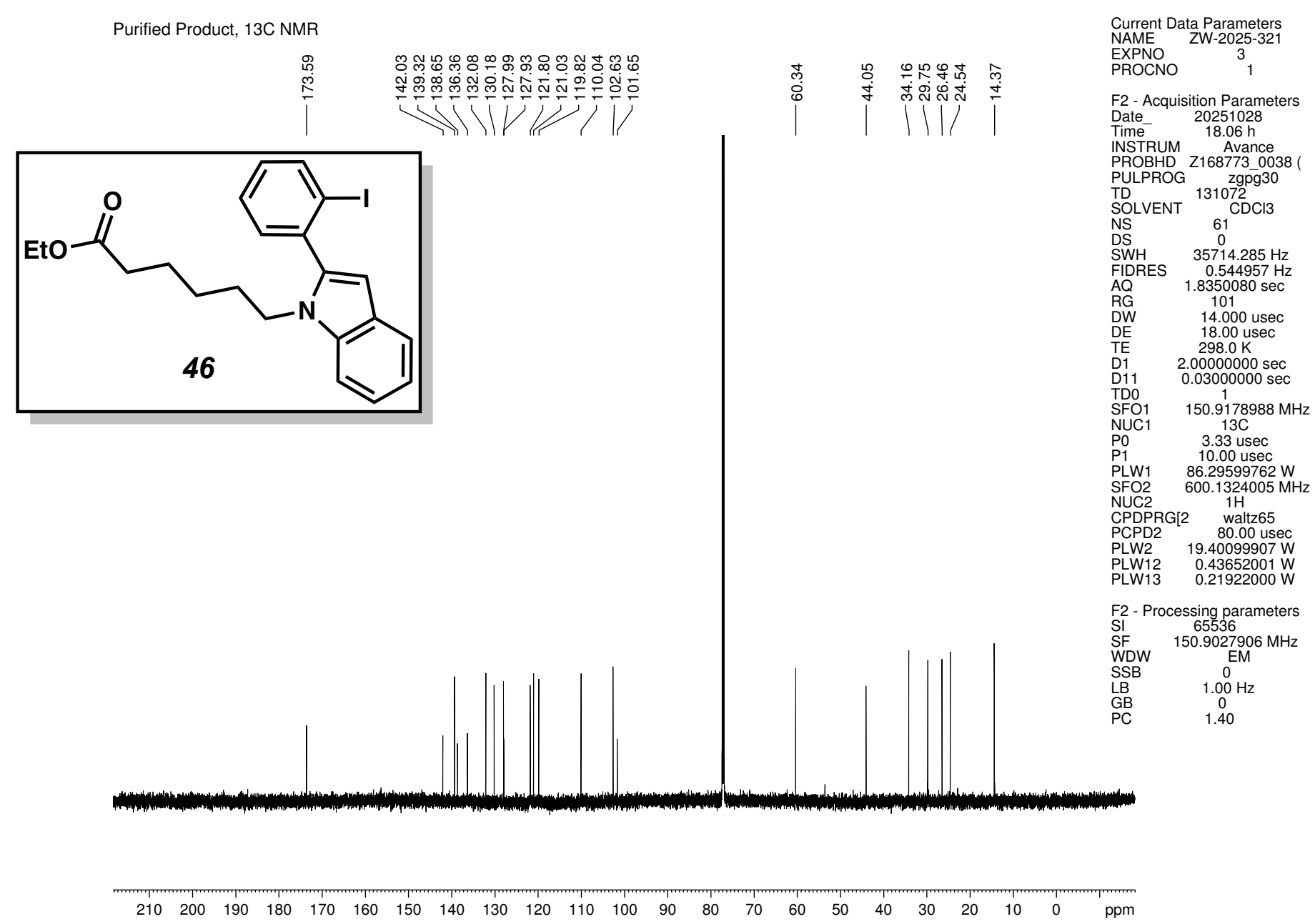

Figure S24. ^{13}C NMR (125 MHz, $CDCl_3$) of compound **46.**

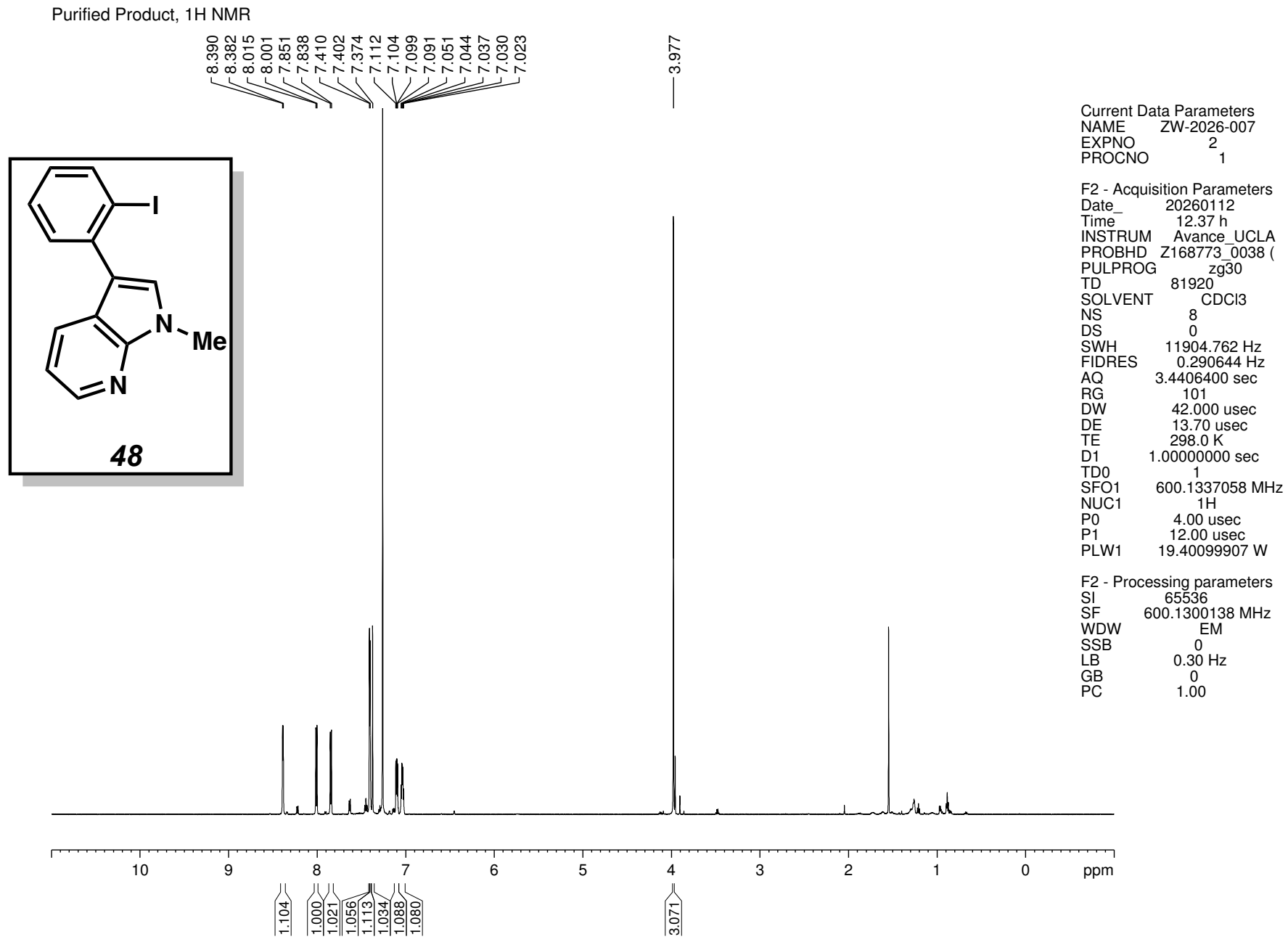

Figure S25. ^{1}H NMR (500 MHz, $CDCl_3$) of compound **48.**

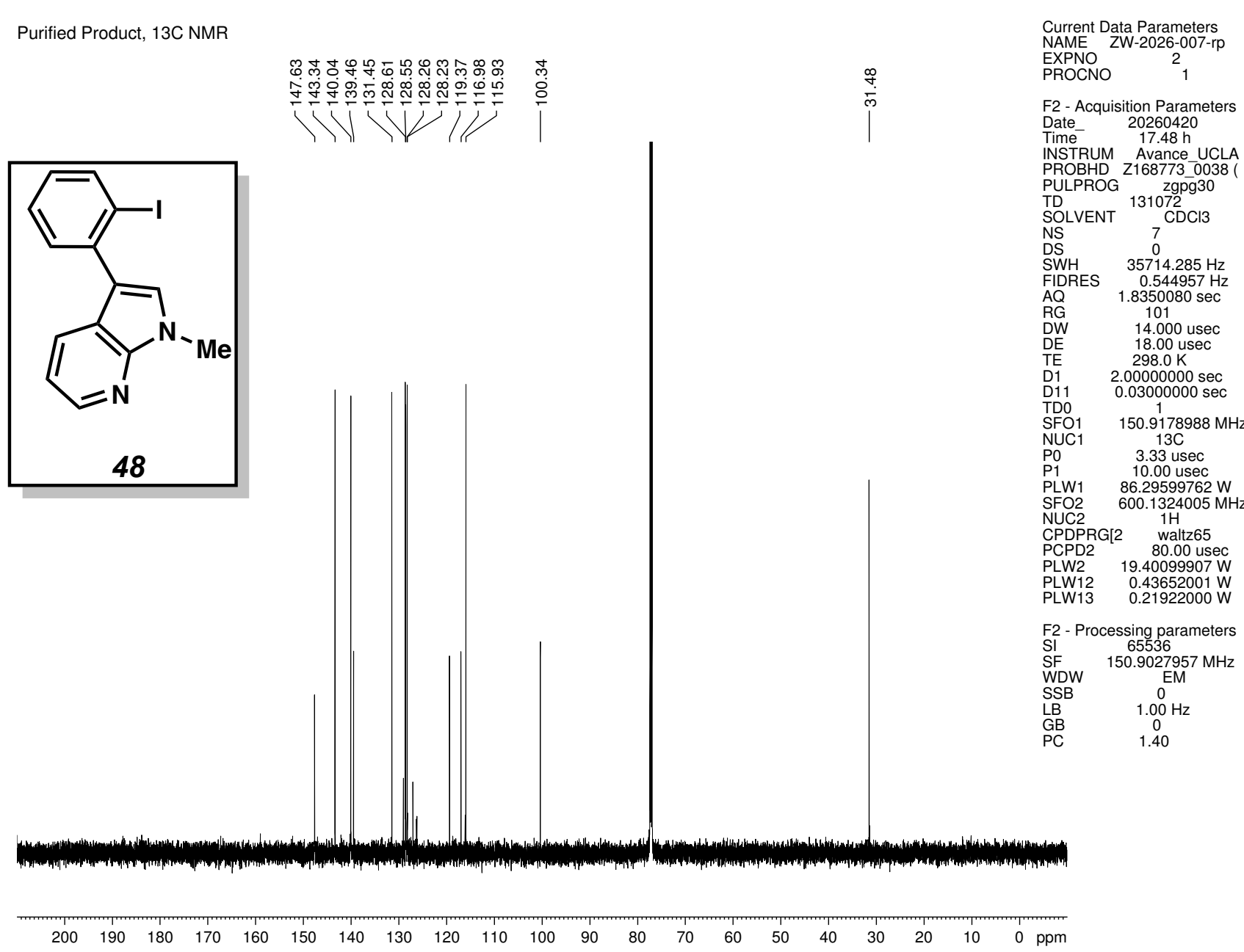

Figure S26. ^{13}C NMR (150 MHz, $CDCl_3$) of compound **48.**

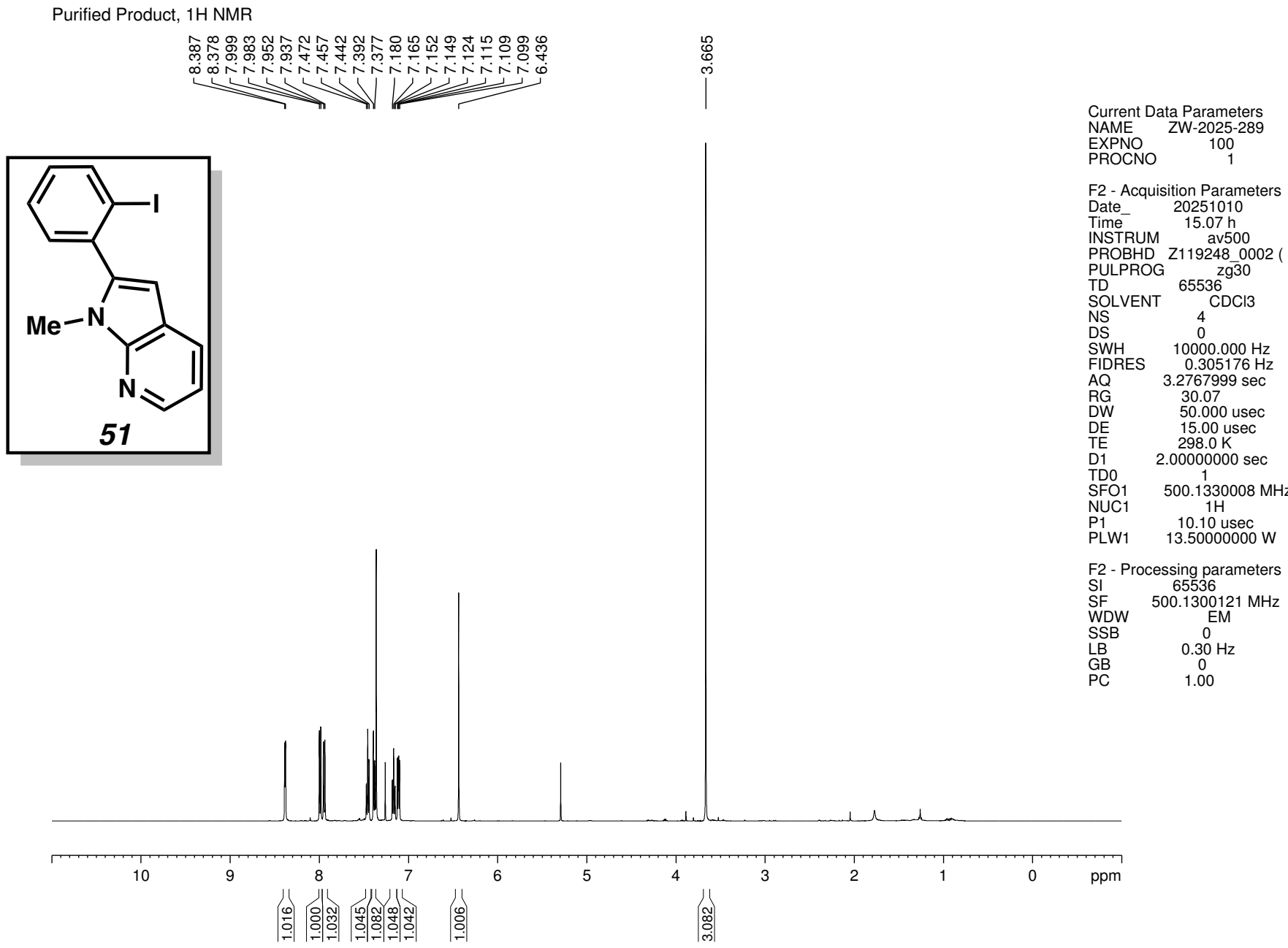

Figure S27. ^{1}H NMR (500 MHz, $CDCl_3$) of compound **51.**

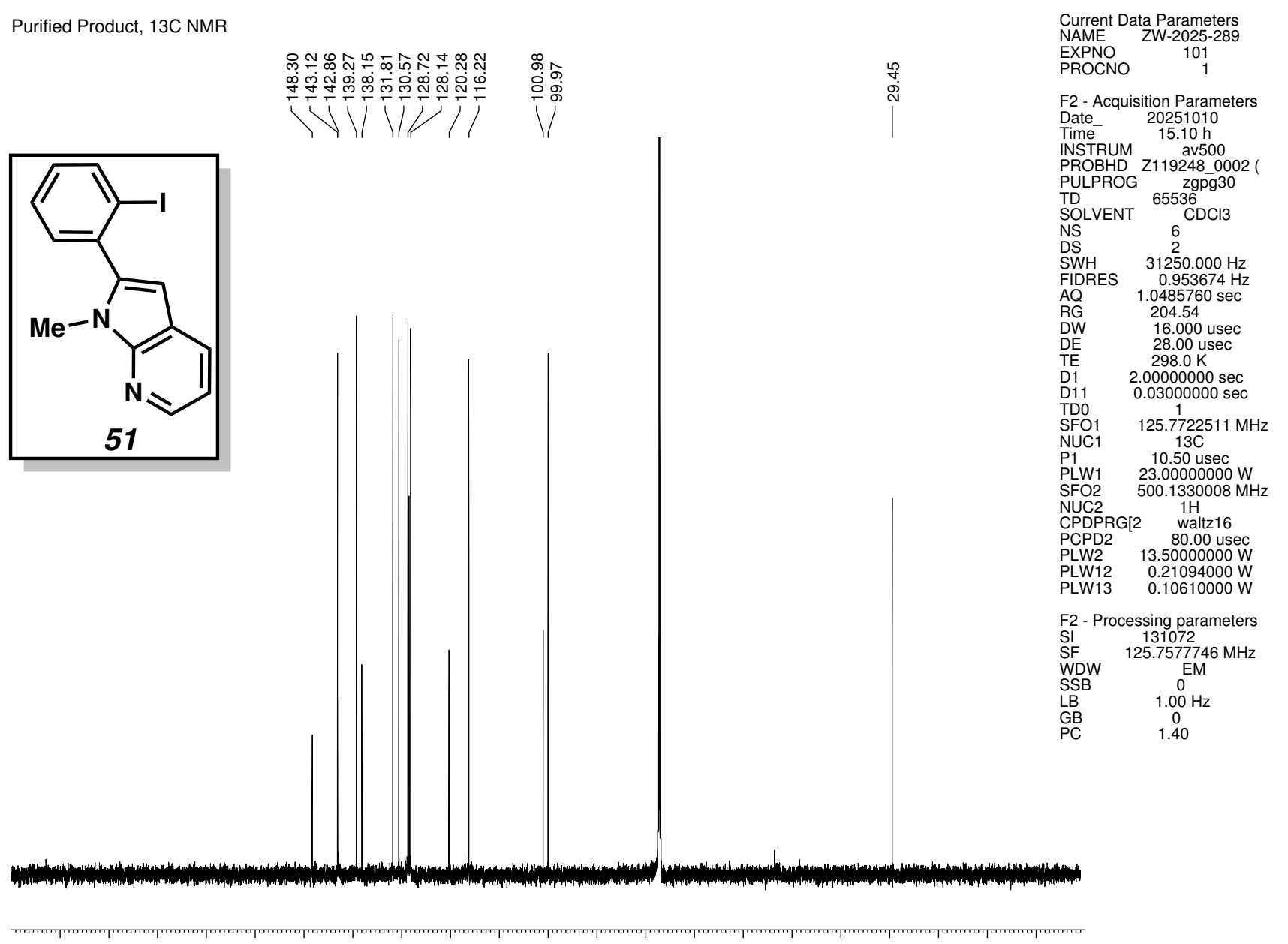

Figure S28. ^{13}C NMR (125 MHz, $CDCl_3$) of compound **51.**

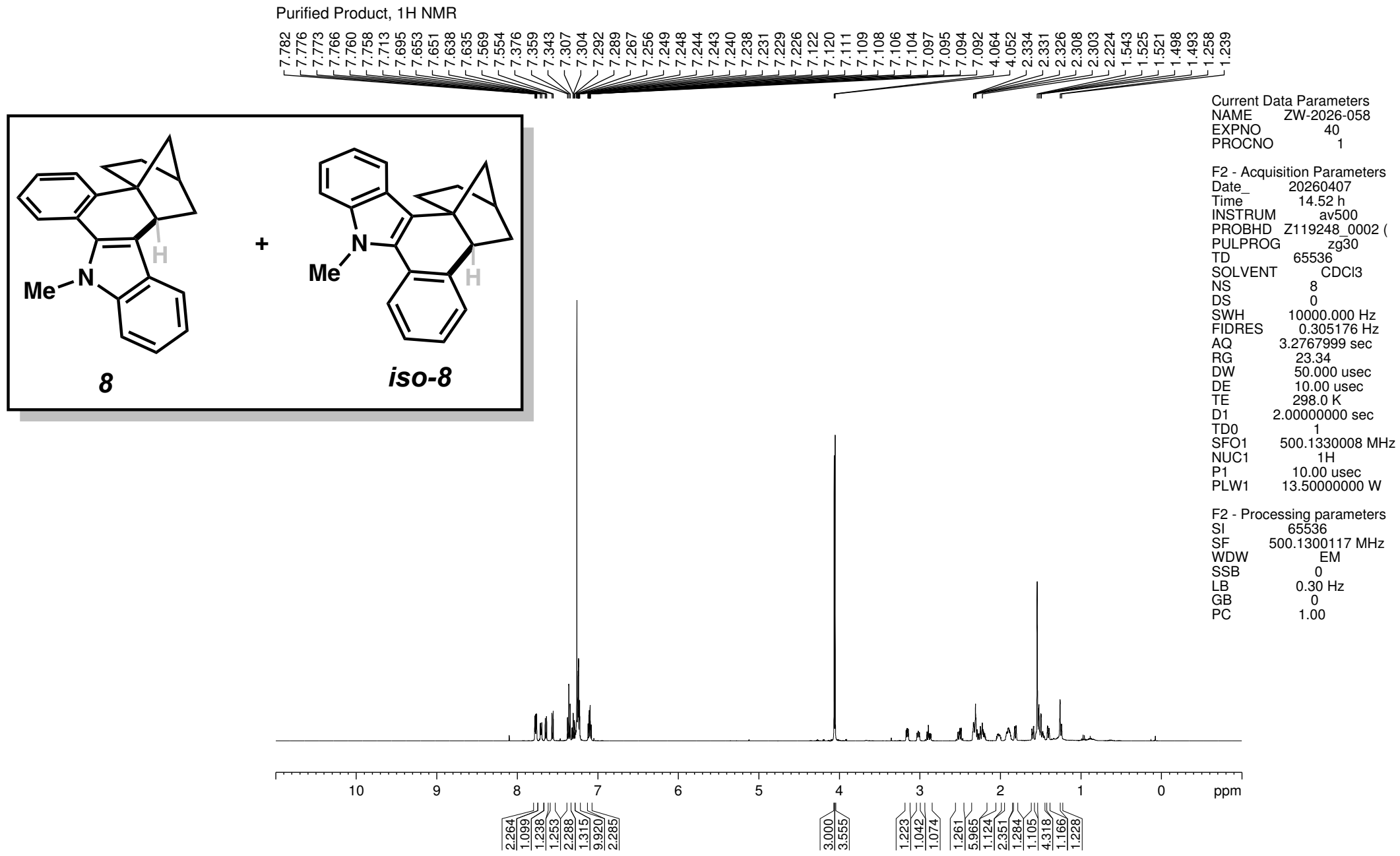

Figure S29. ^{1}H NMR (500 MHz, $CDCl_3$) of annulation products **8** and ***iso*-8**.

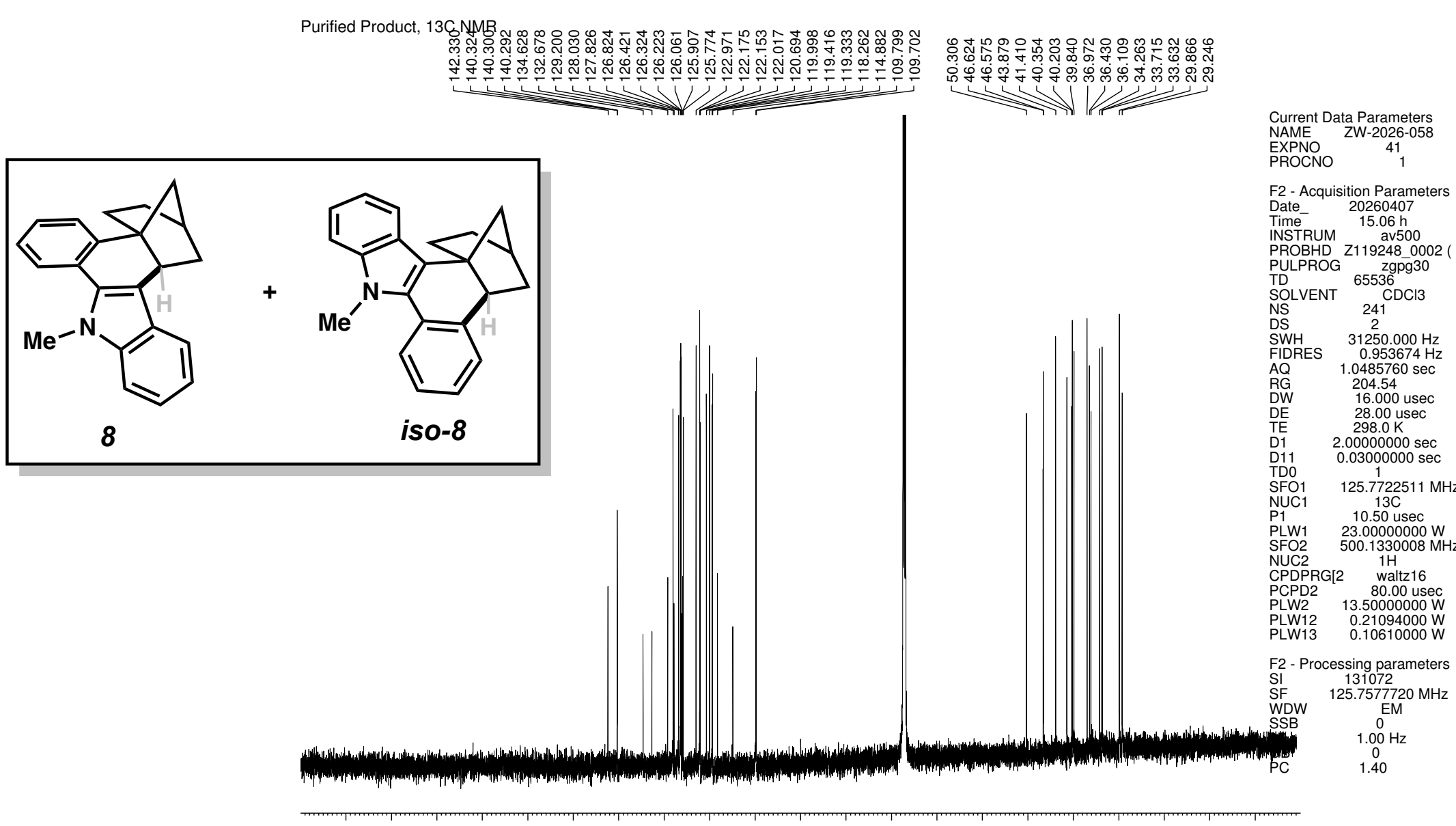

Figure S30. ^{13}C NMR (125 MHz, $CDCl_3$) of annulation products **8** and ***iso*-8**.

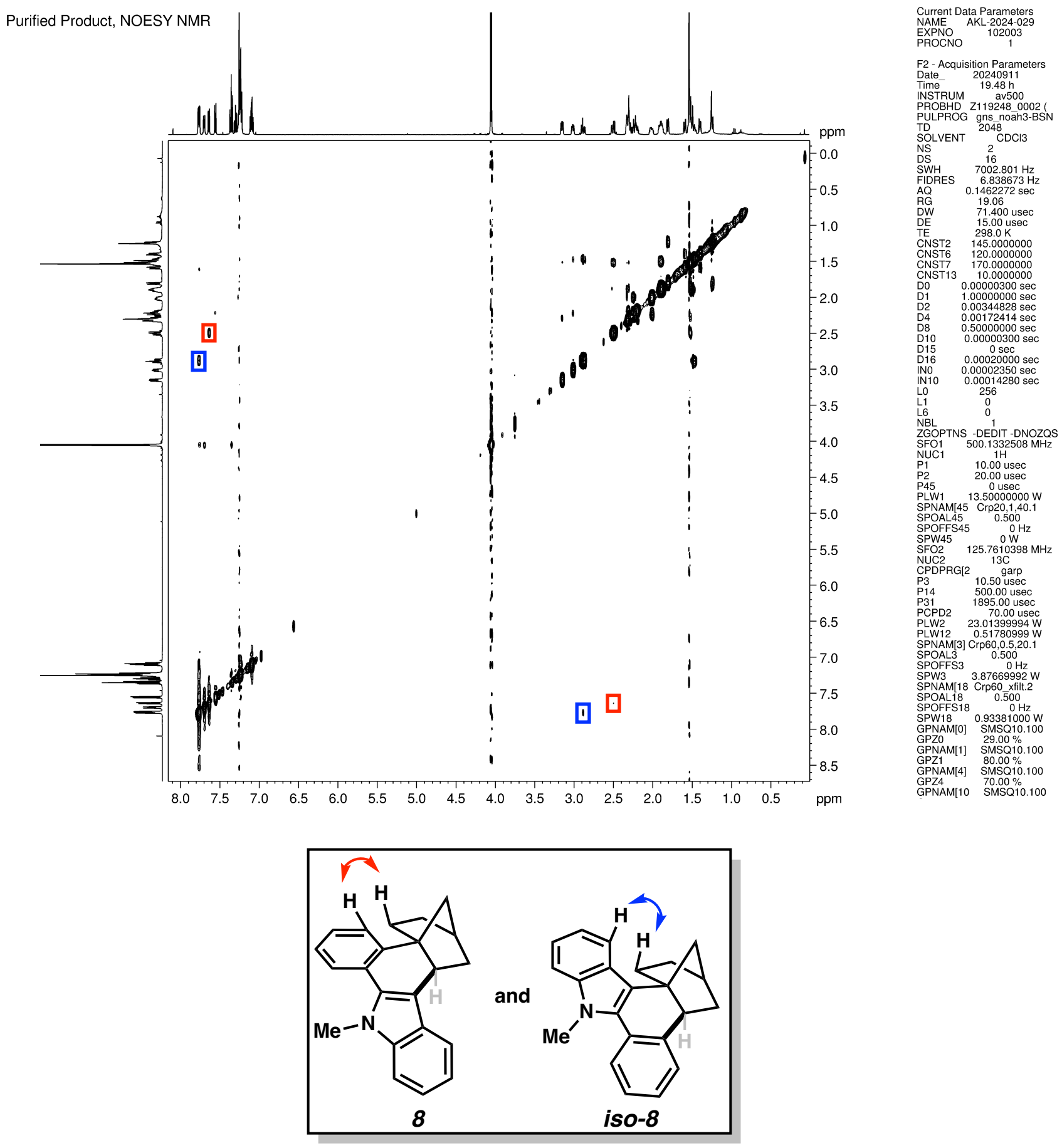

Figure S31. 2D NOESY (500 MHz, $CDCl_3$) of annulation products **8** and ***iso*-8**.

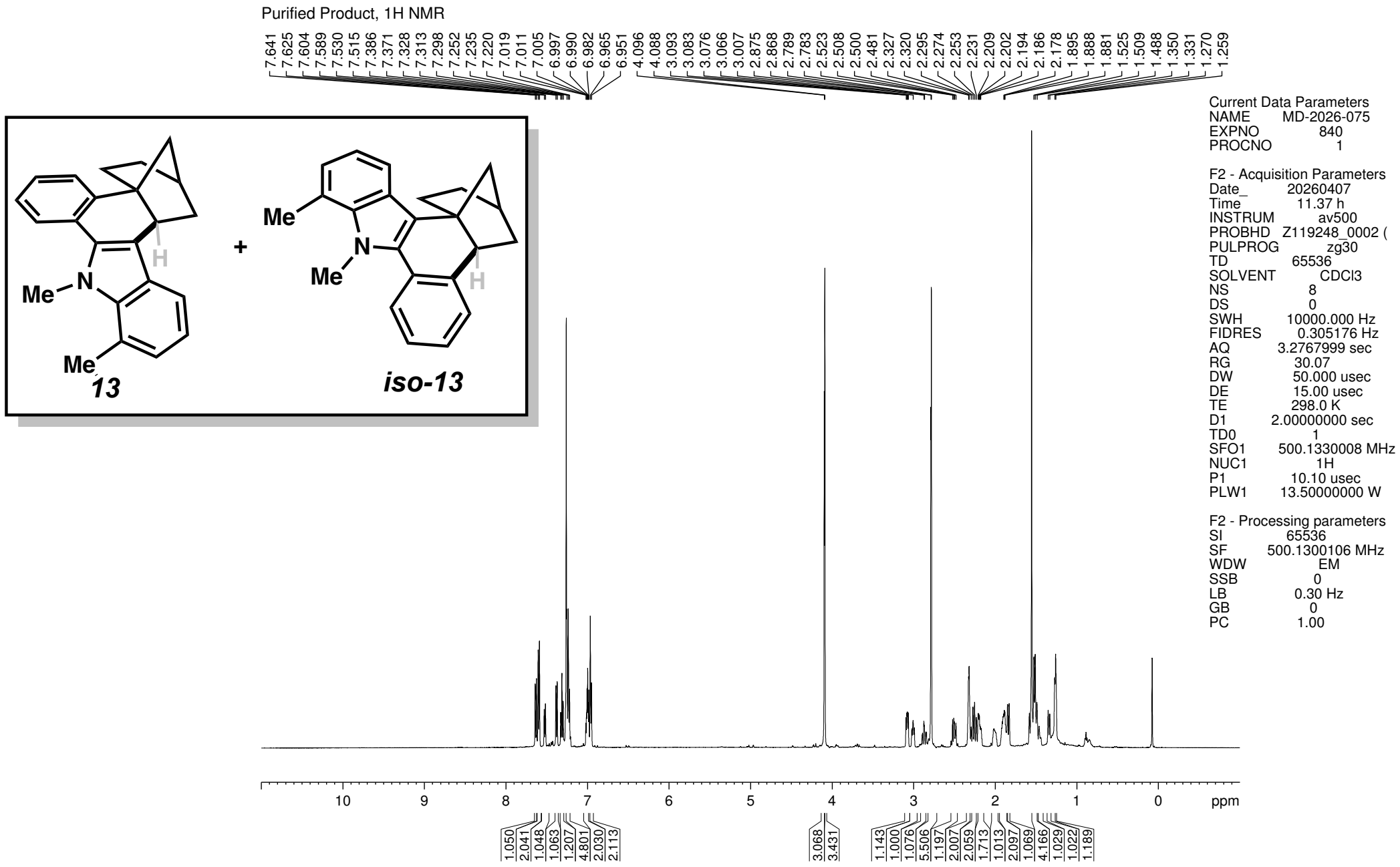

Figure S32. ^{1}H NMR (500 MHz, $CDCl_3$) of annulation products **13** and ***iso*-13**.

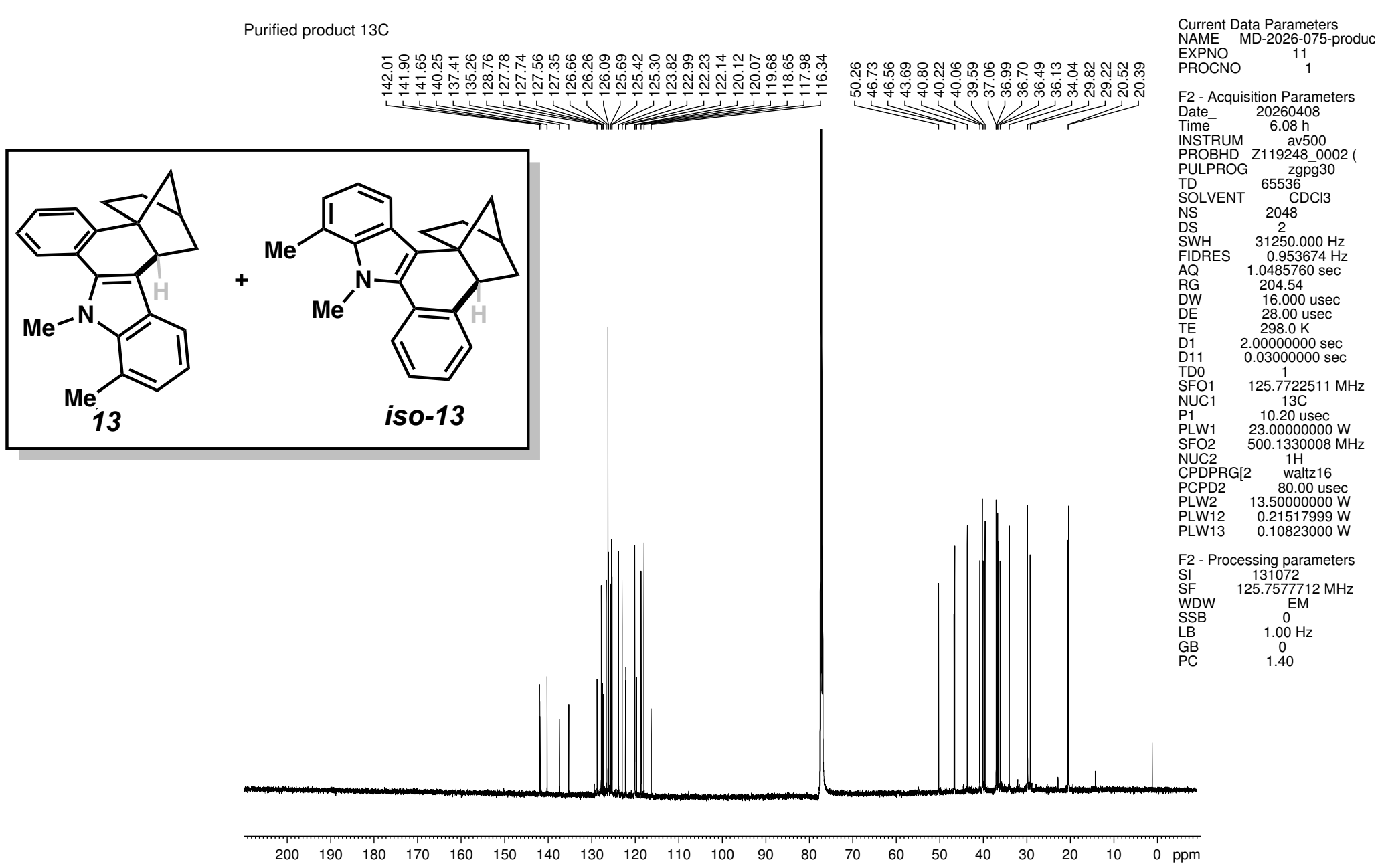

Figure S33. ^{13}C NMR (125 MHz, $CDCl_3$) of annulation products **13** and ***iso*-13**.

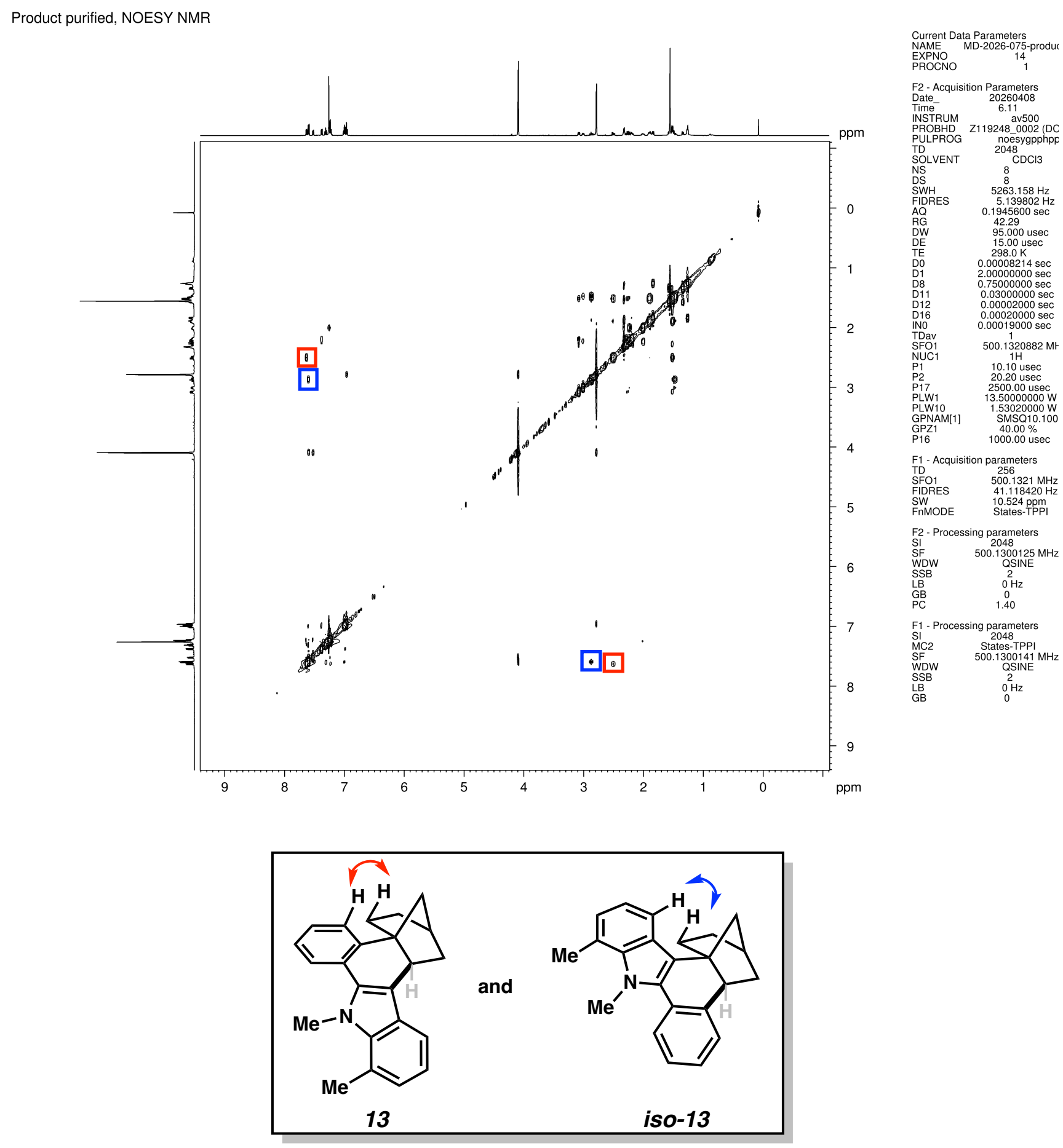

Figure S34. NOESY NMR (500 MHz, $CDCl_3$) of annulation products **13** and ***iso*-13**.

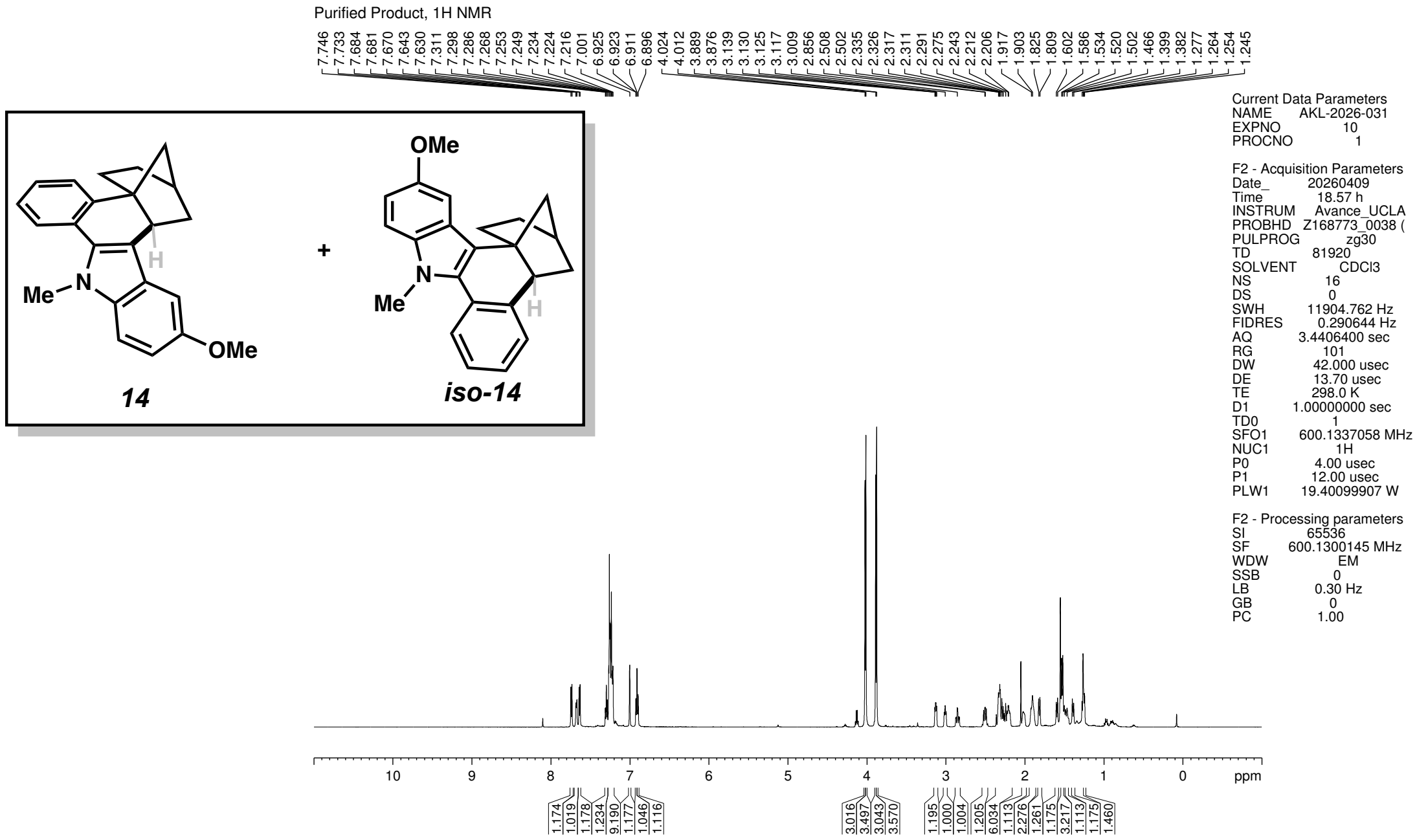

Figure S35. ^{1}H NMR (600 MHz, $CDCl_3$) of annulation products **14** and ***iso*-14**.

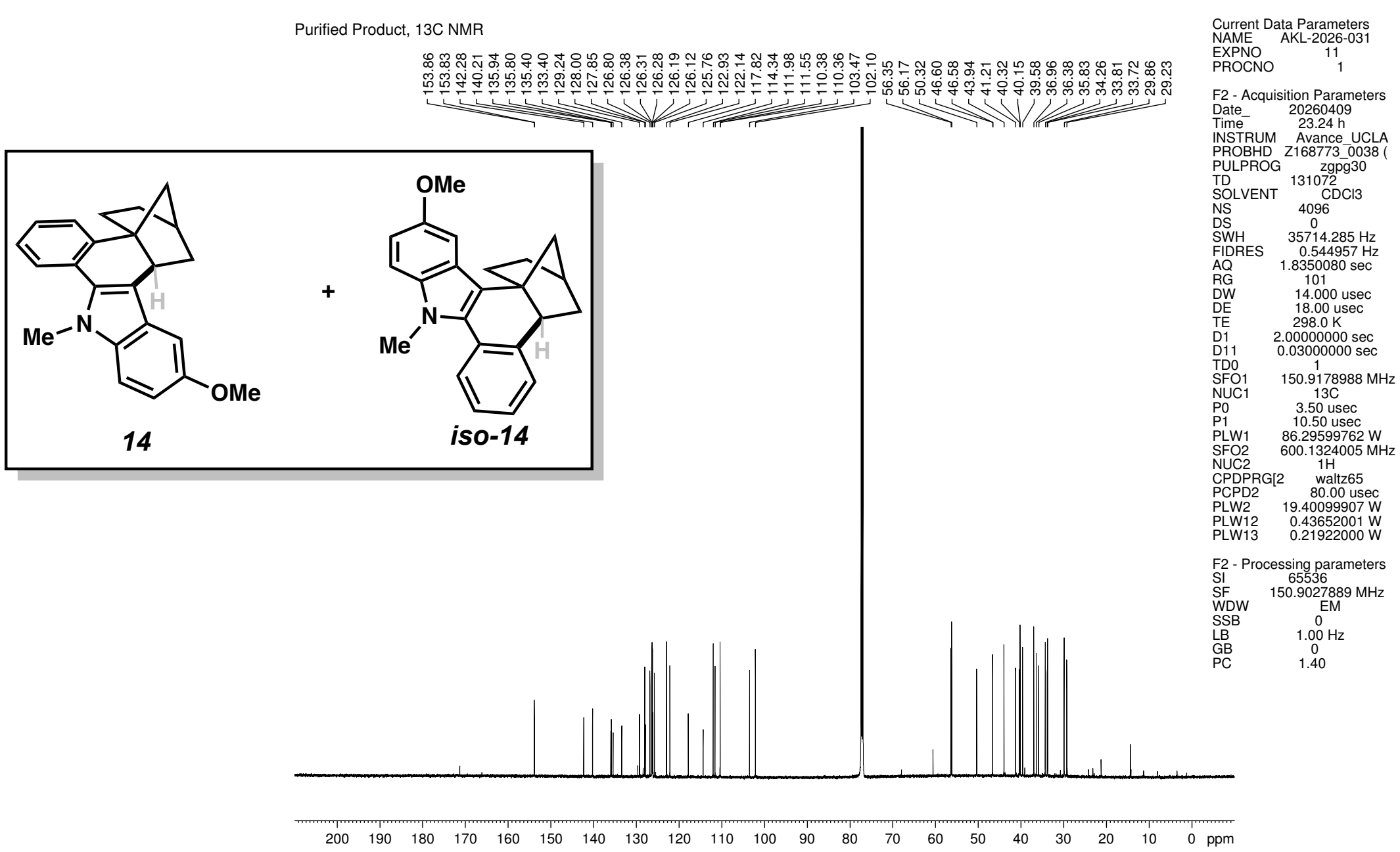

Figure S36. ^{13}C NMR (150 MHz, $CDCl_3$) of annulation products **14** and ***iso*-14**.

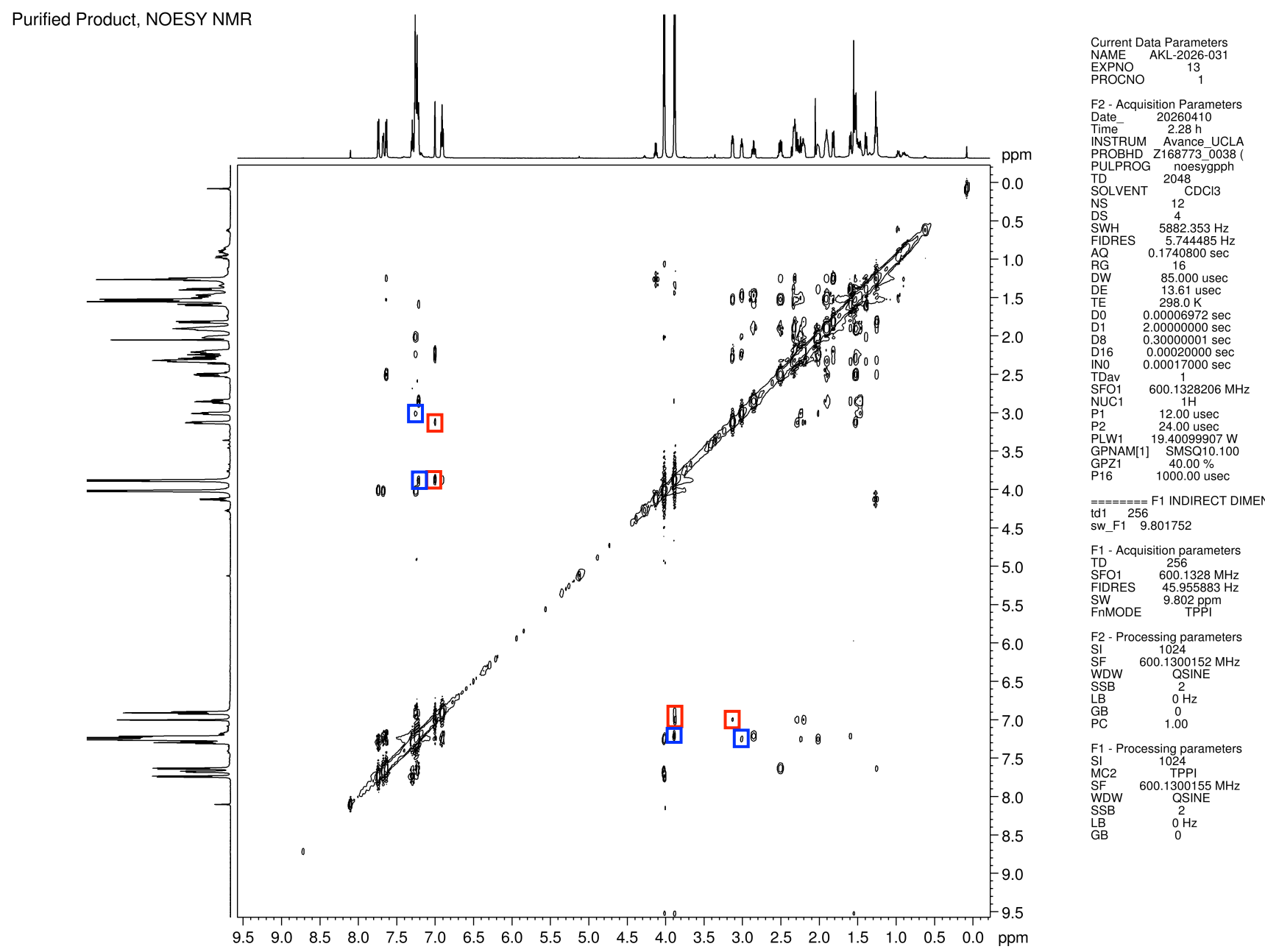

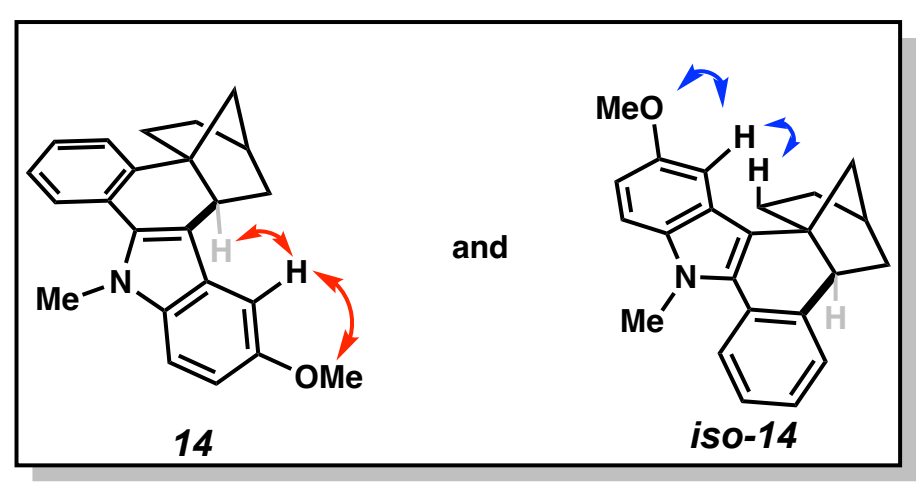

Figure S37. NOESY NMR (600 MHz, $CDCl_3$) of annulation products **14** and ***iso*-14**.

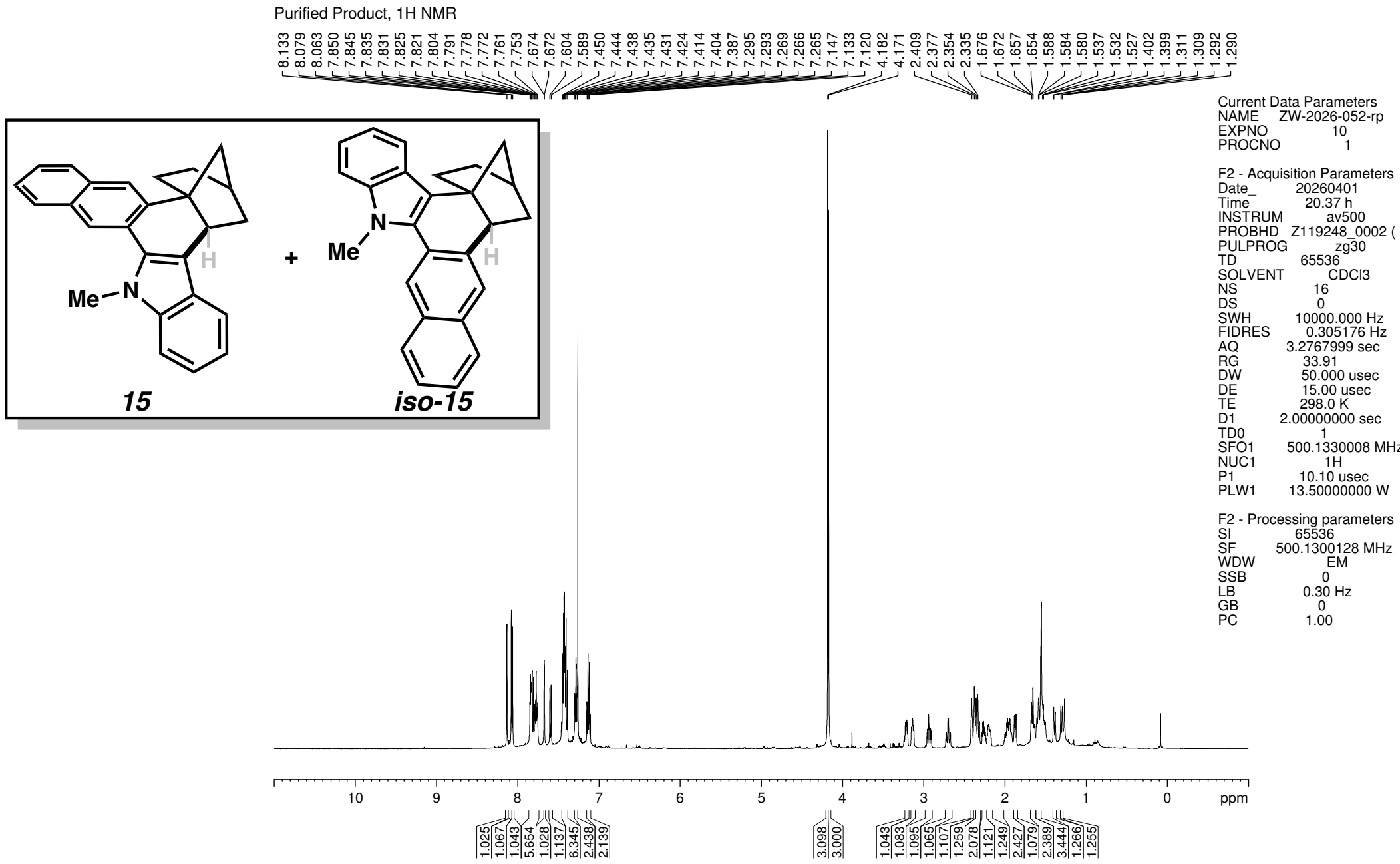

Figure S38. ^{1}H NMR (500 MHz, $CDCl_3$) of annulation products **15** and ***iso*-15**.

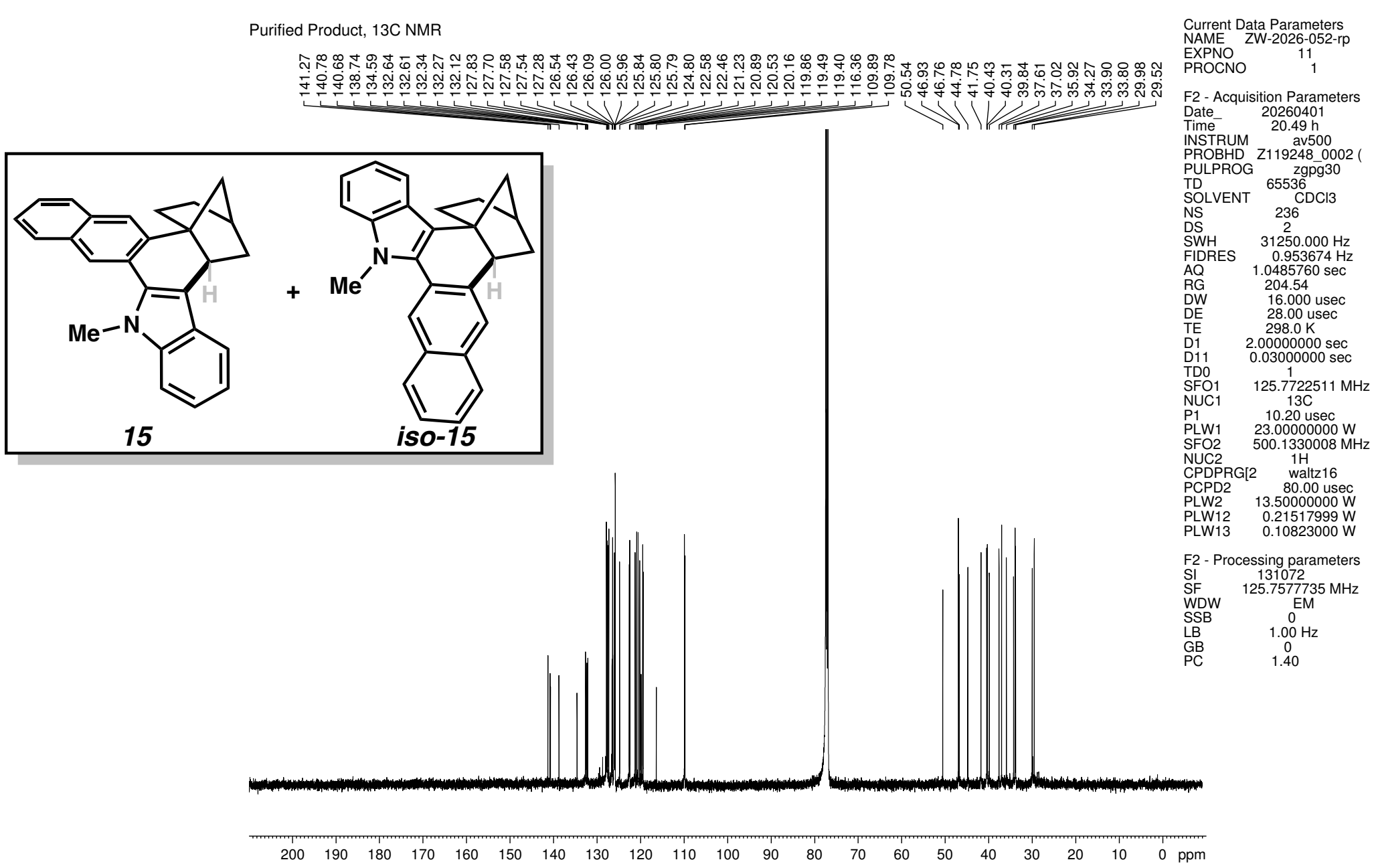

Figure S39. ^{13}C NMR (125 MHz, $CDCl_3$) of annulation products **15** and ***iso*-15**.

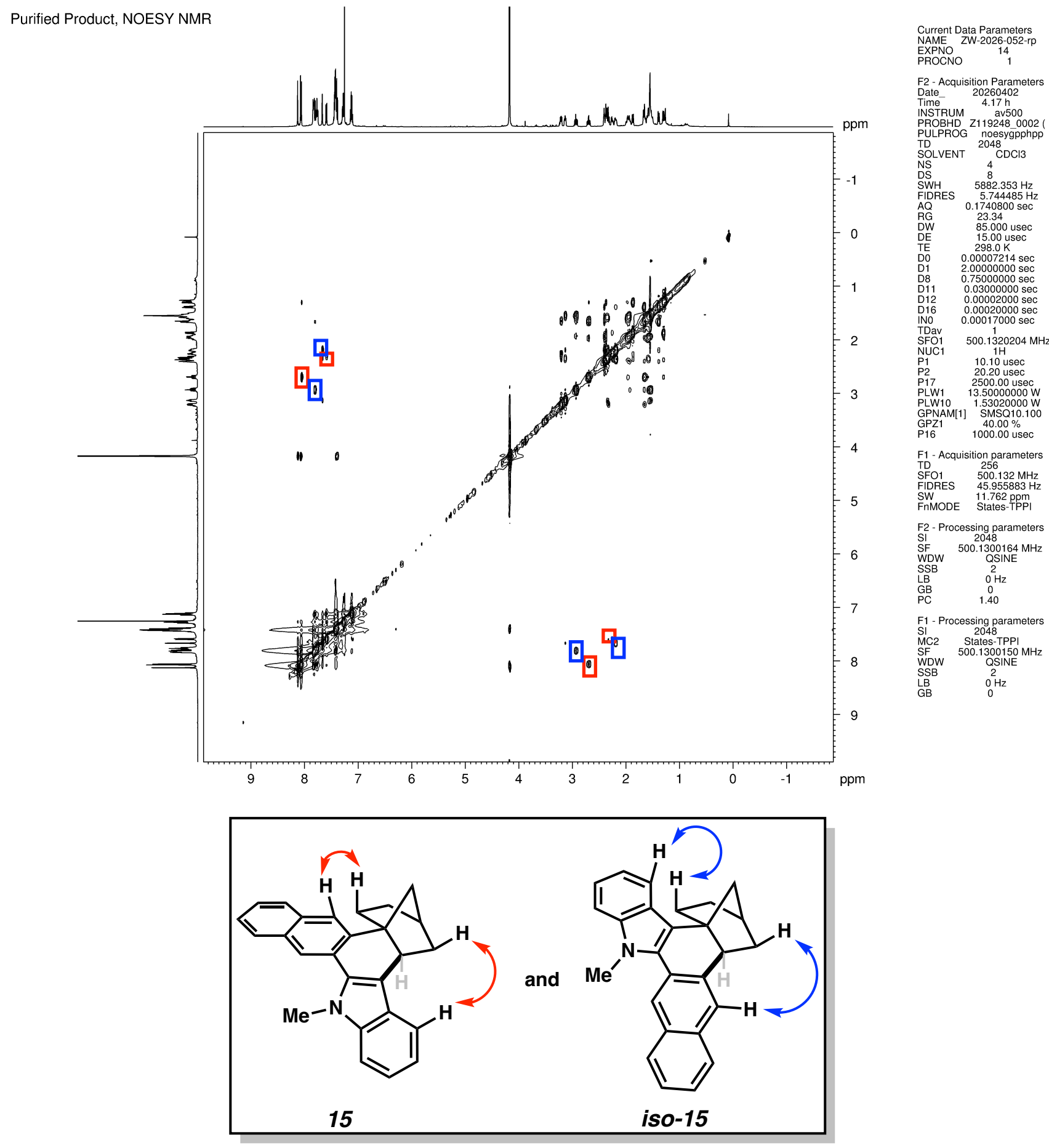

Figure S40. 2D NOESY (500 MHz, $CDCl_3$) of annulation products **15** and ***iso*-15**.

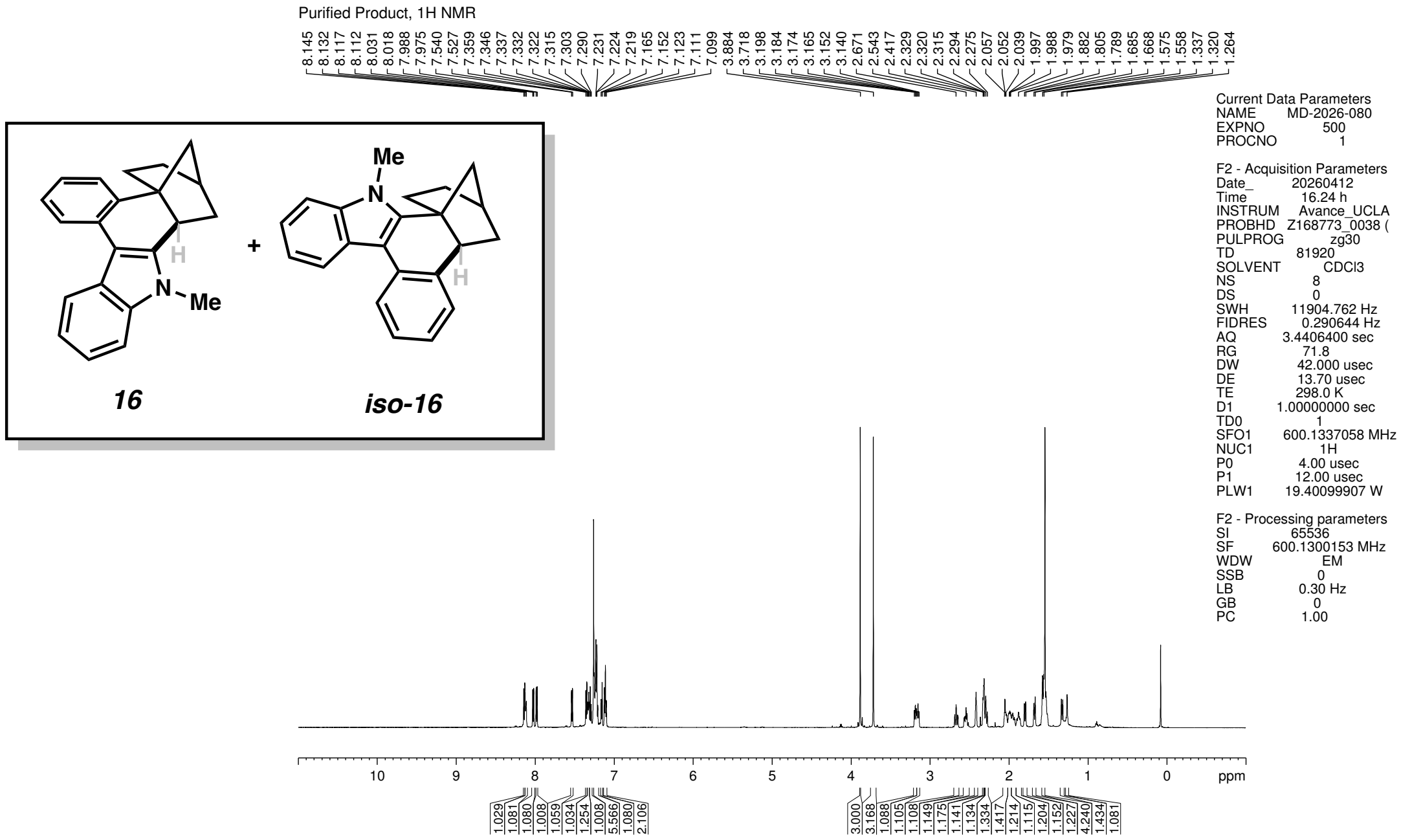

Figure S41. ^{1}H NMR (600 MHz, $CDCl_3$) of annulation products **16** and ***iso*-16**.

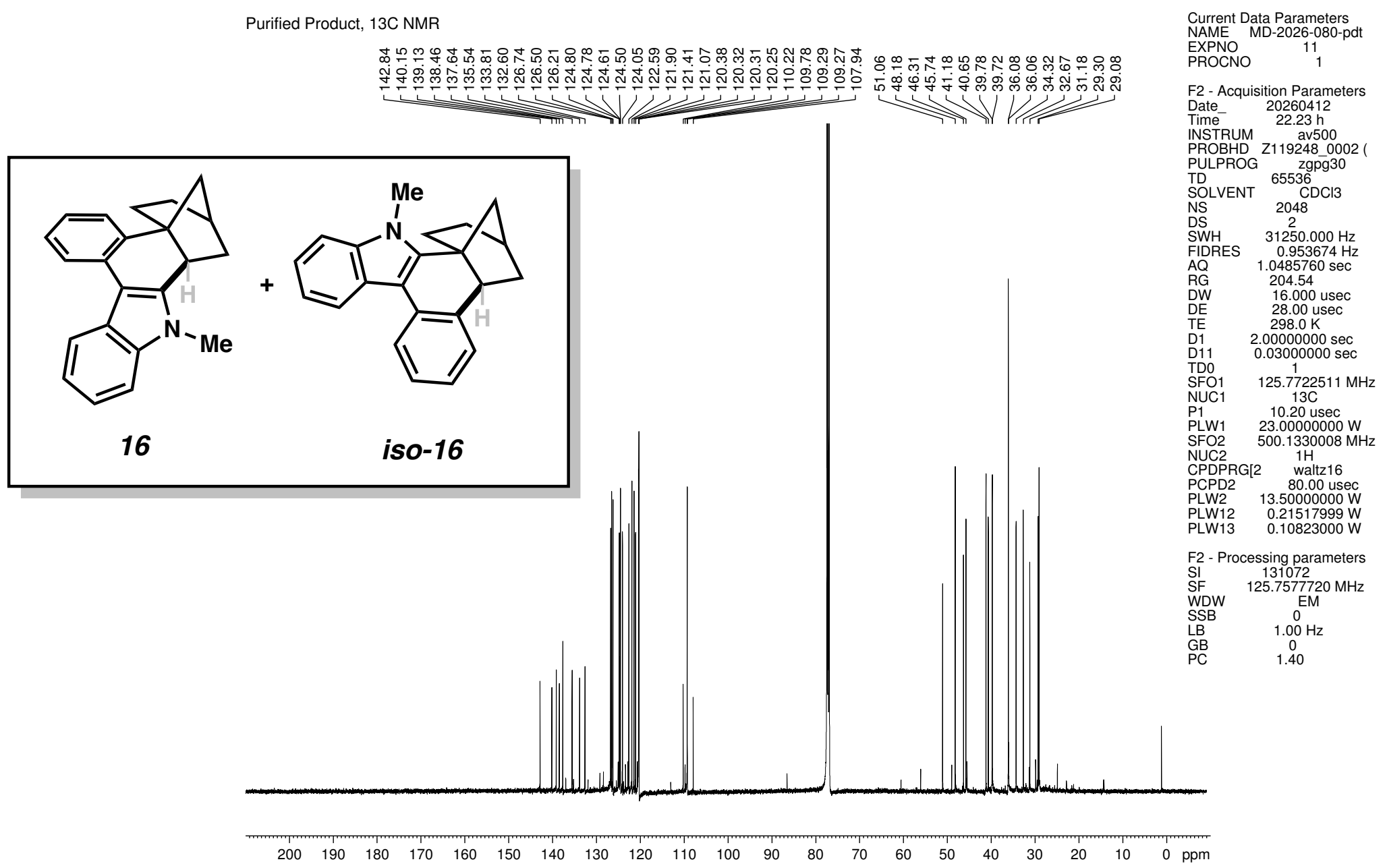

Figure S42. ^{13}C NMR (125 MHz, $CDCl_3$) of annulation products **16** and ***iso*-16**.

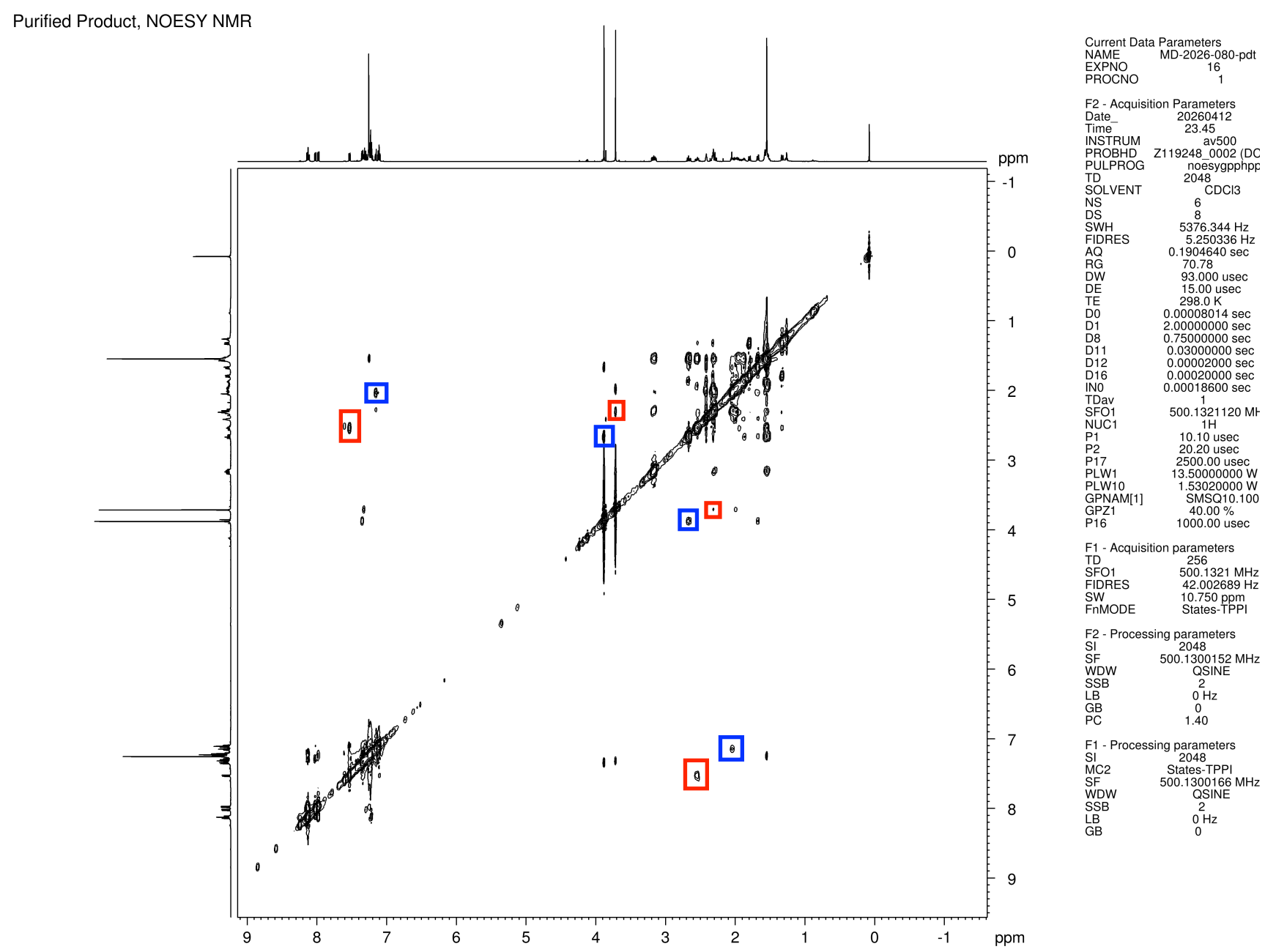

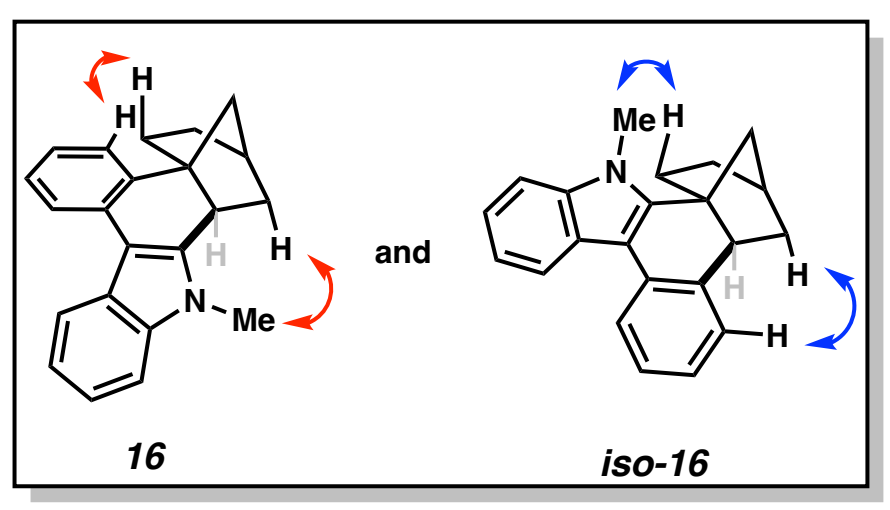

Figure S43. 2D NOESY (500 MHz, $CDCl_3$) of annulation products **16** and ***iso*-16**.

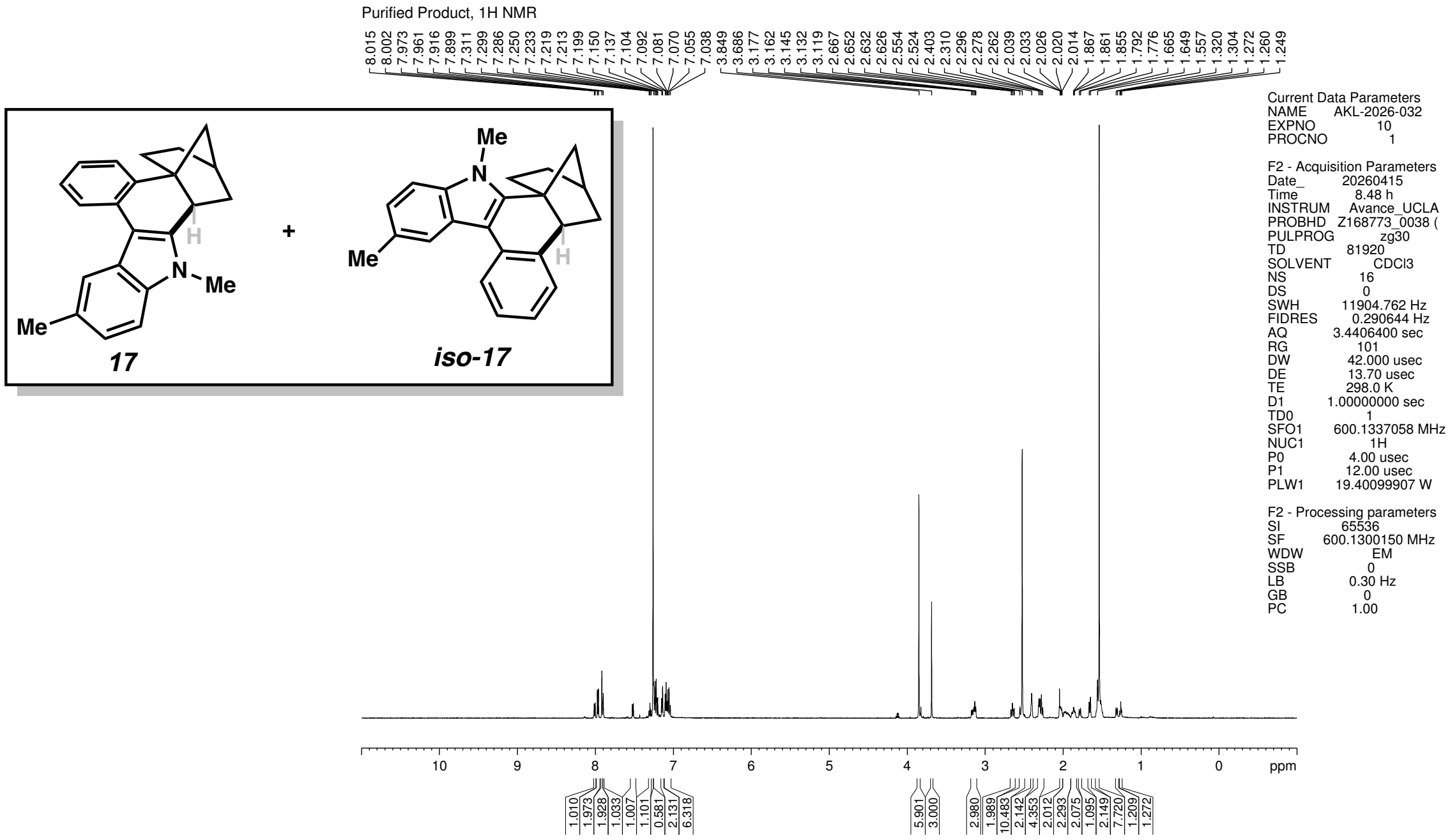

Figure S44. ^{1}H NMR (600 MHz, $CDCl_3$) of annulation product **17** and ***iso*-17**.

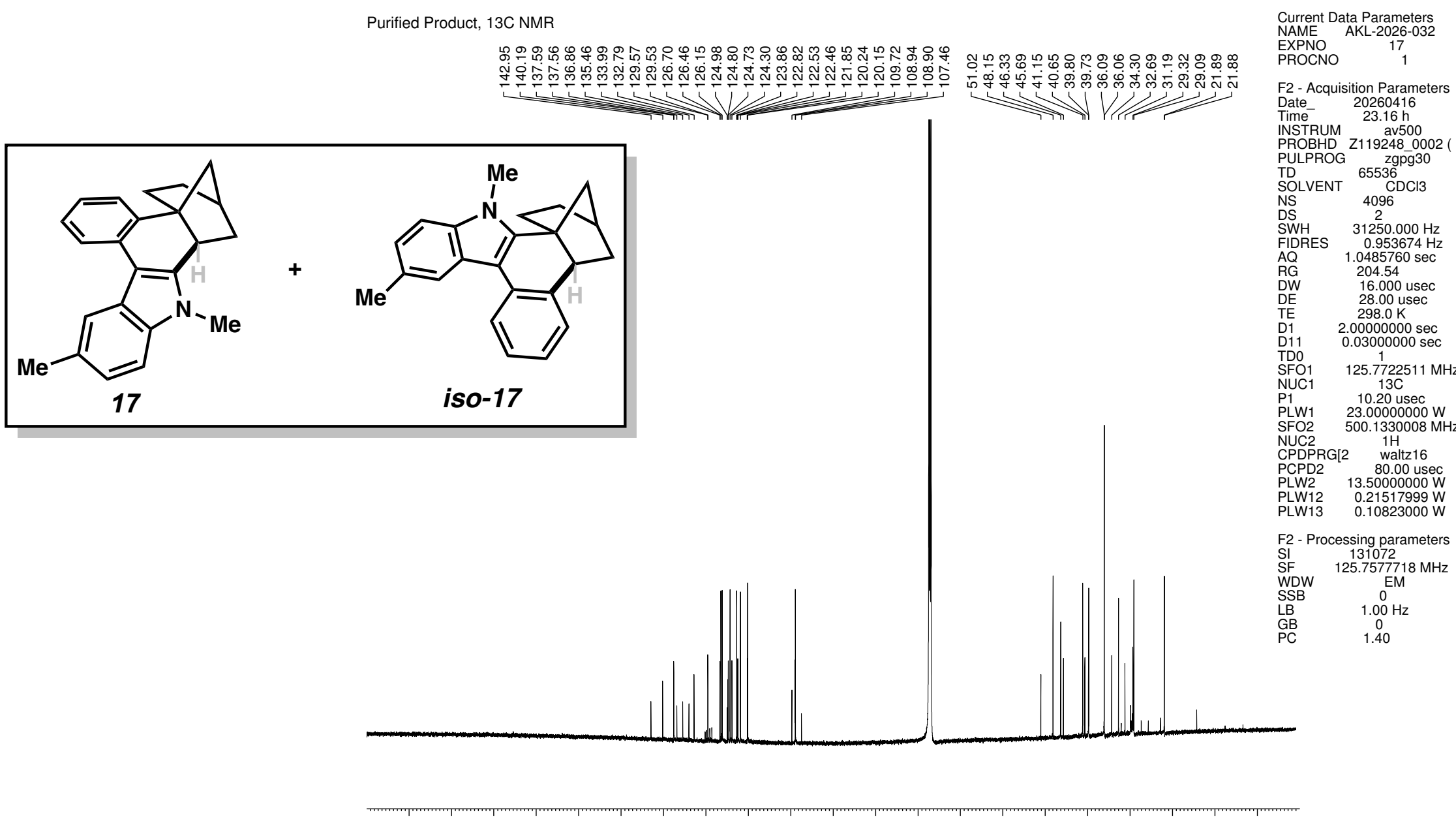

Figure S45. ^{13}C NMR (125 MHz, $CDCl_3$) of annulation product **17** and ***iso*-17**.

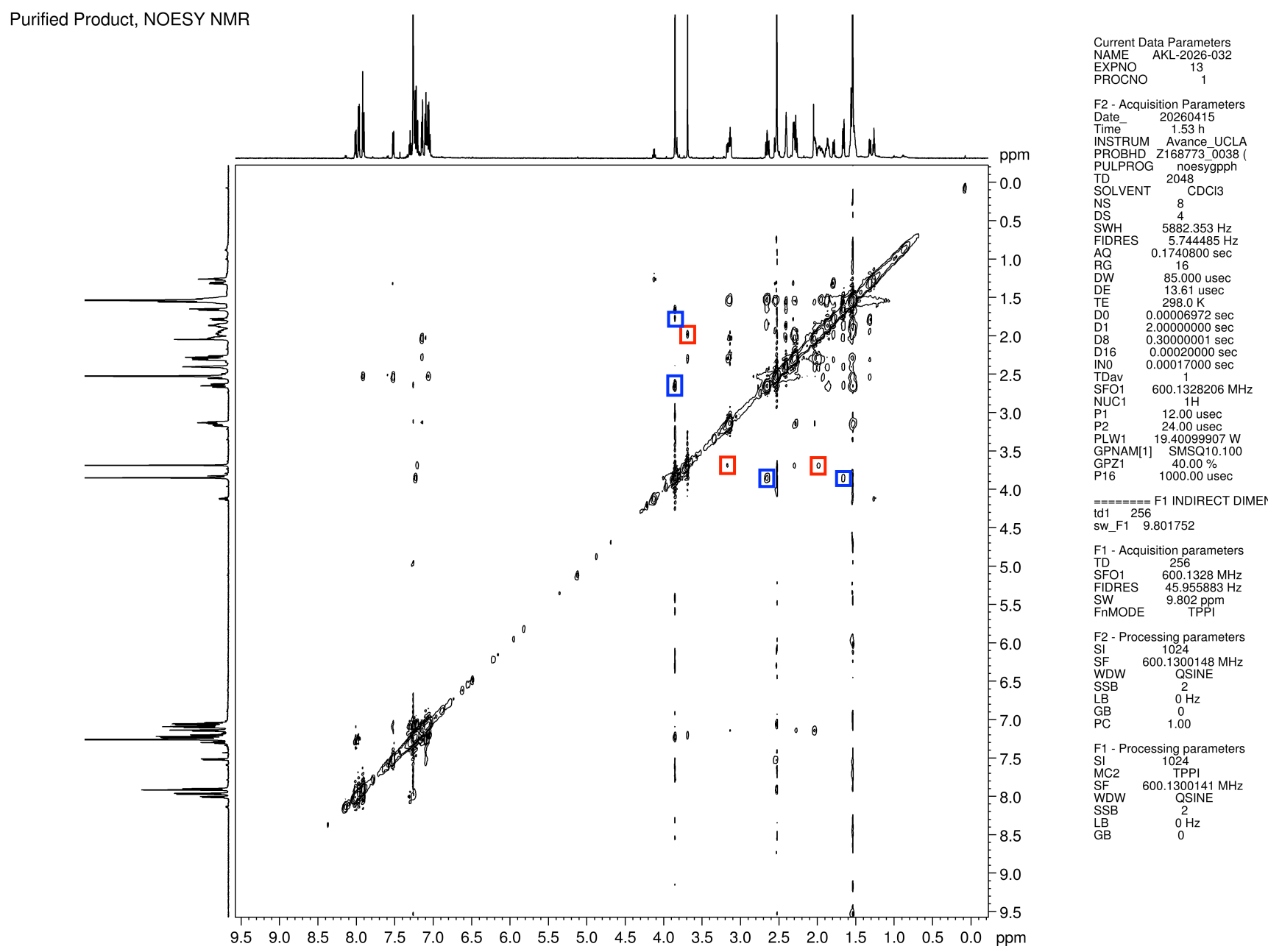

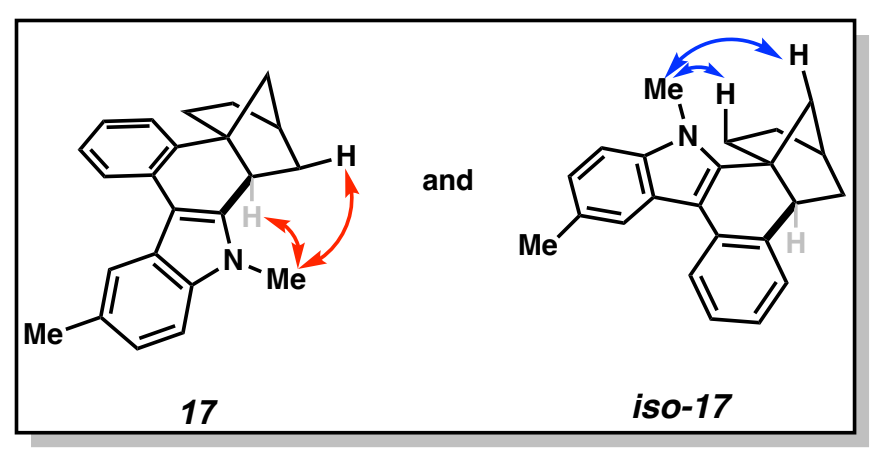

Figure S46. NOESY NMR (600 MHz, $CDCl_3$) of annulation products **17** and ***iso*-17**.

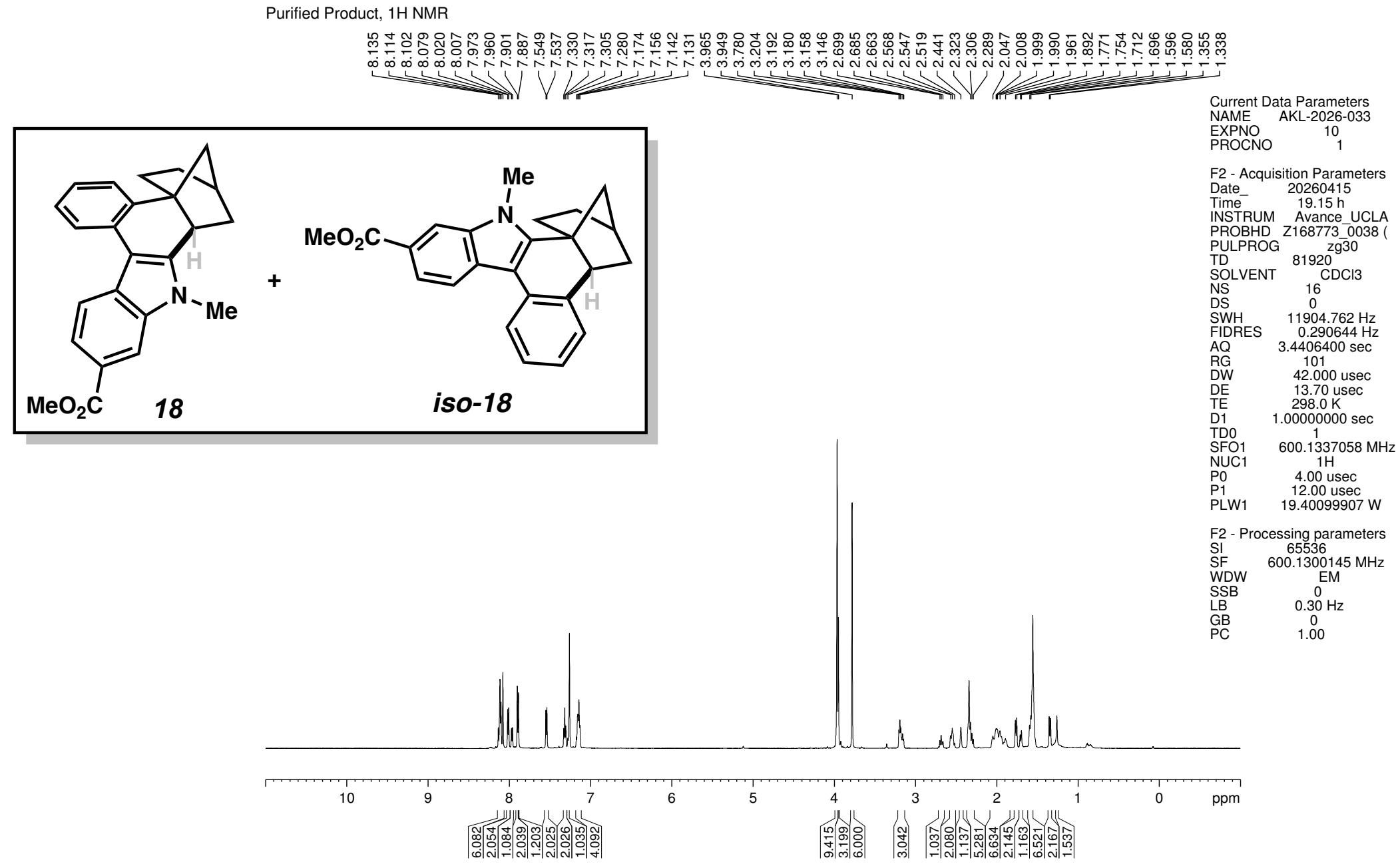

Figure S47. ^{1}H NMR (600 MHz, $CDCl_3$) of annulation products **18** and ***iso*-18**.

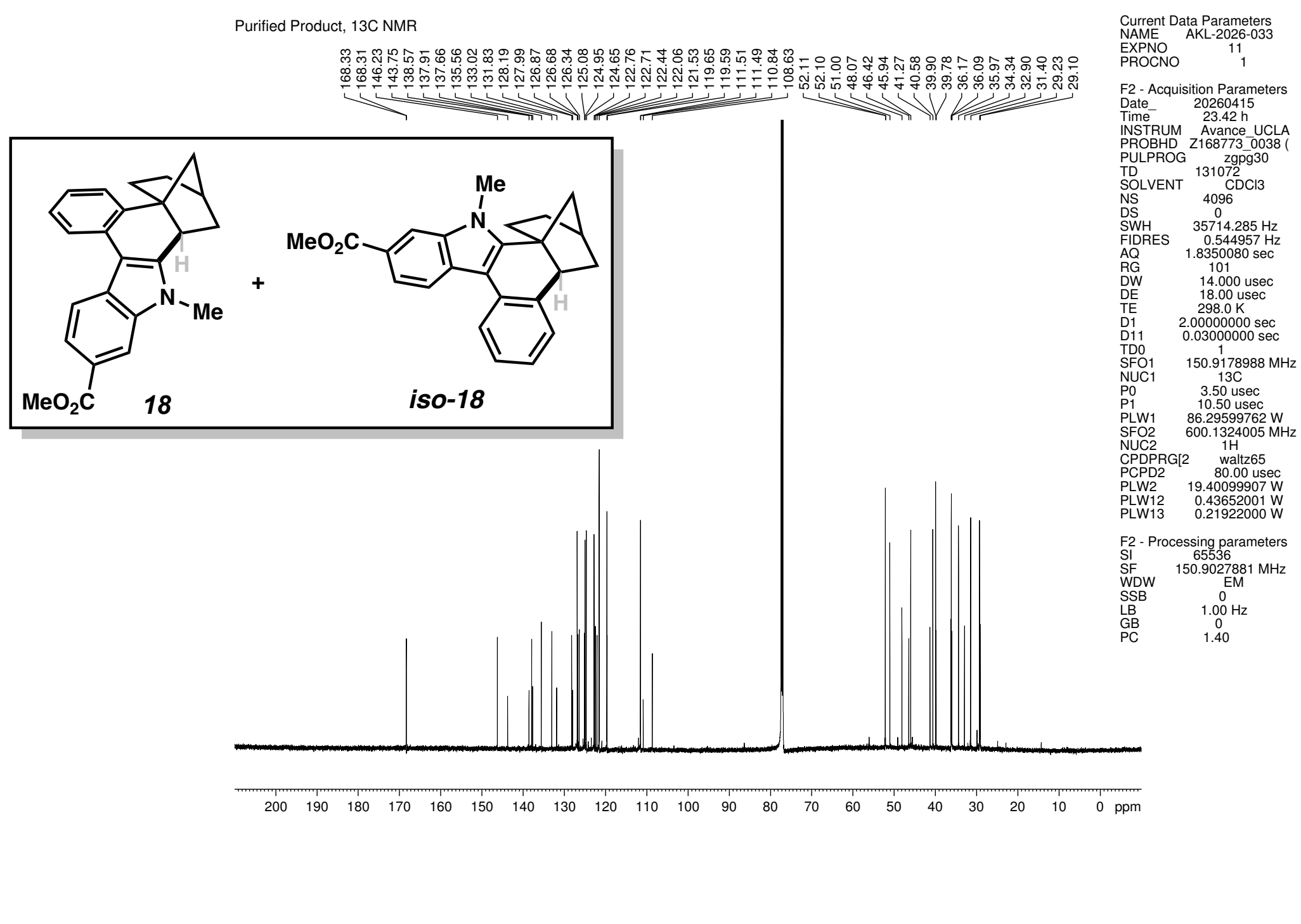

Figure S48. ^{13}C NMR (150 MHz, $CDCl_3$) of annulation products **18** and ***iso*-18**.

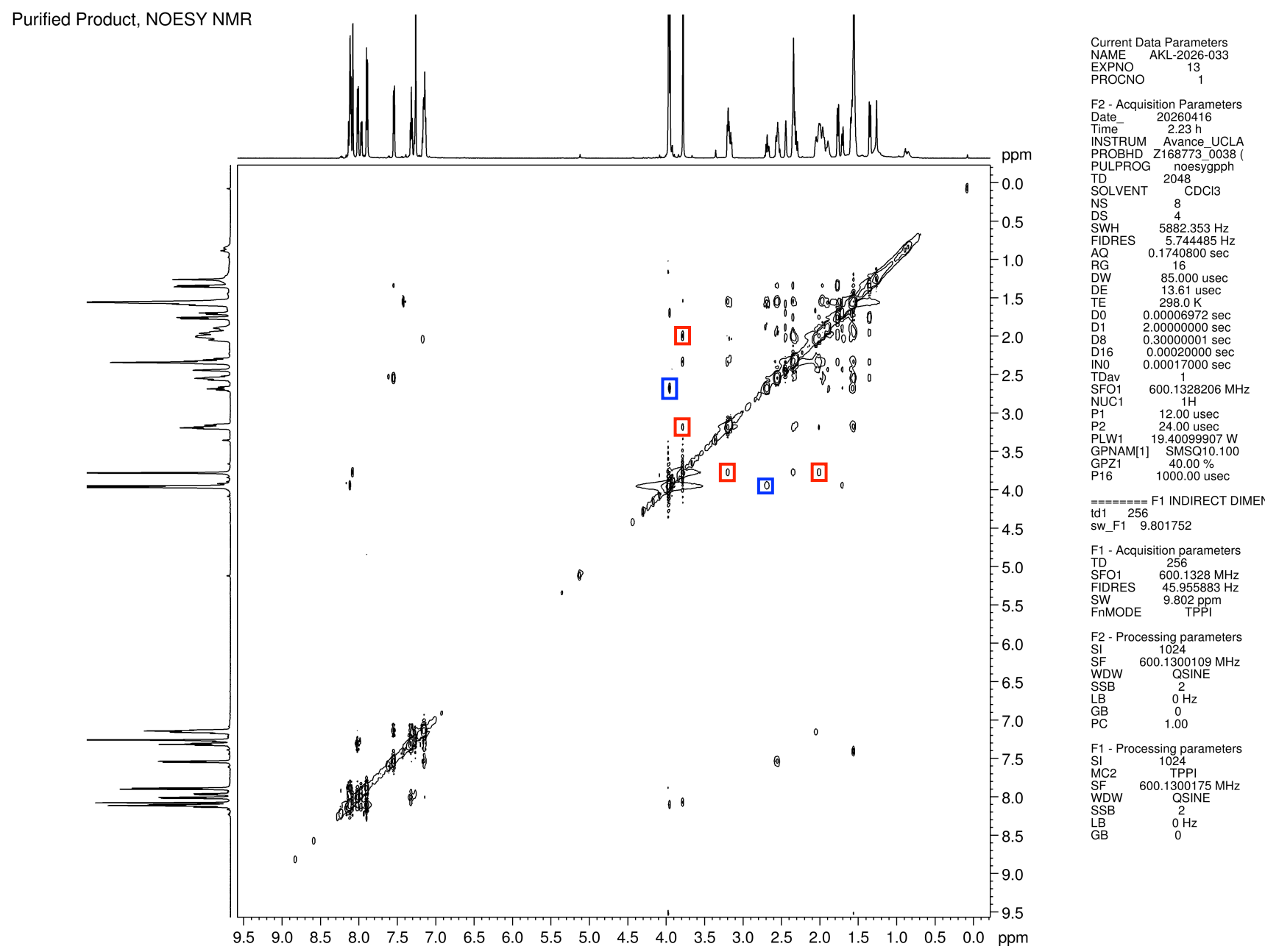

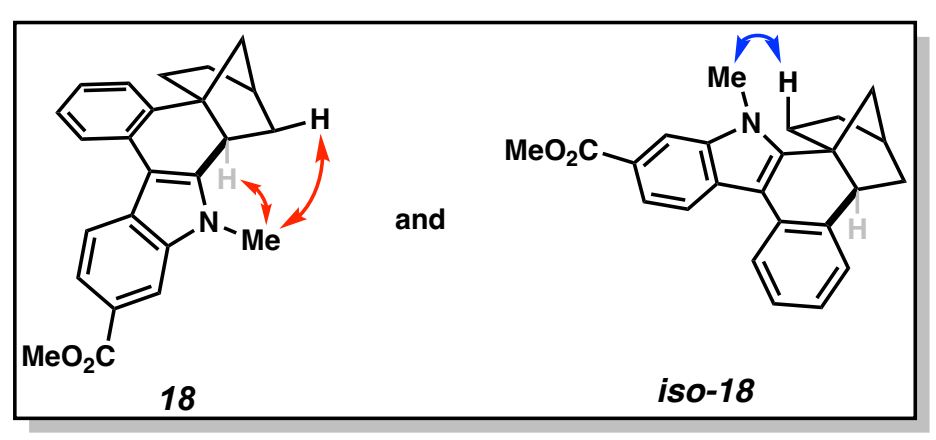

Figure S49. NOESY NMR (600 MHz, $CDCl_3$) of annulation products **18** and ***iso*-18**.

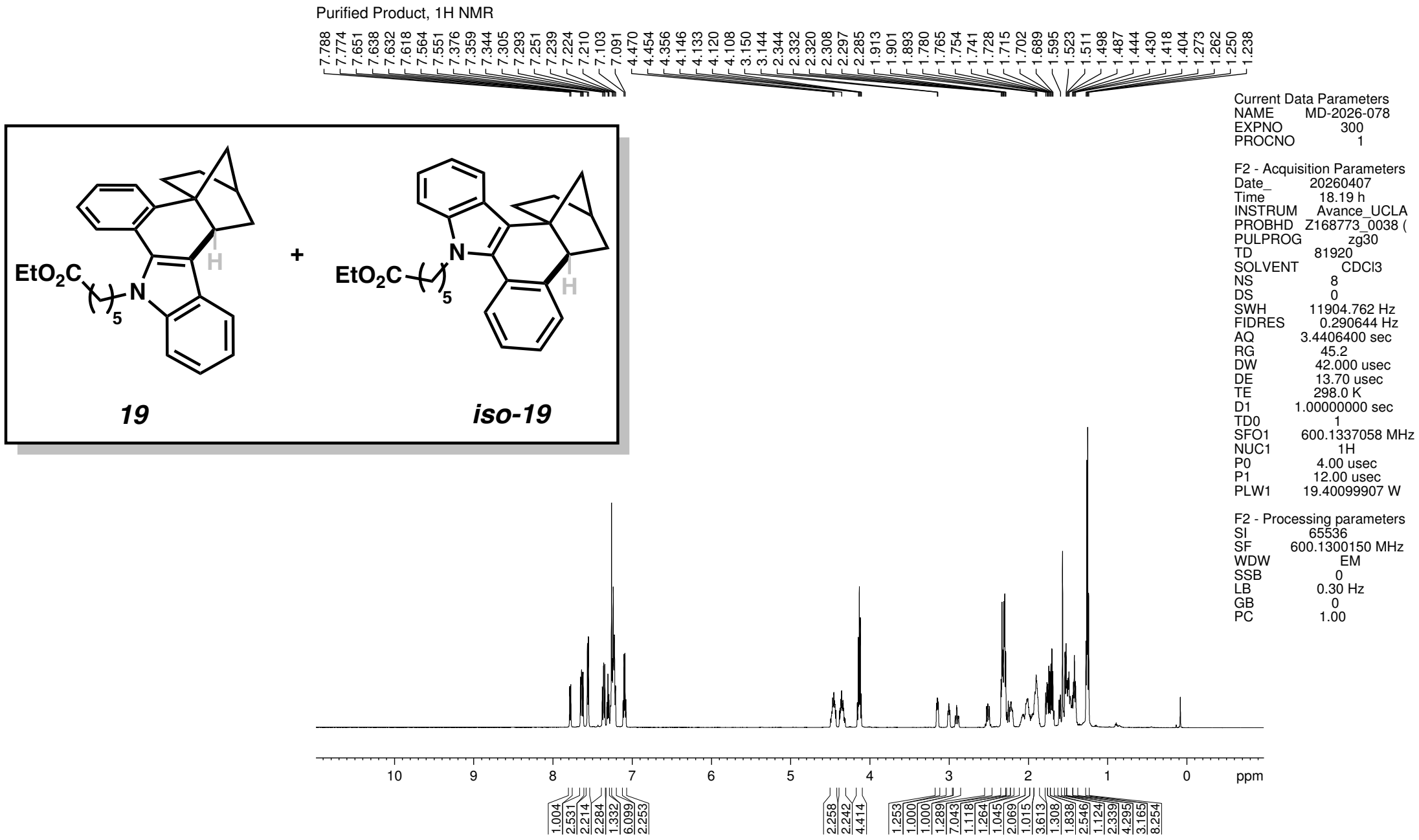

Figure S50. ^{1}H NMR (600 MHz, $CDCl_3$) of annulation products **19** and ***iso*-19**.

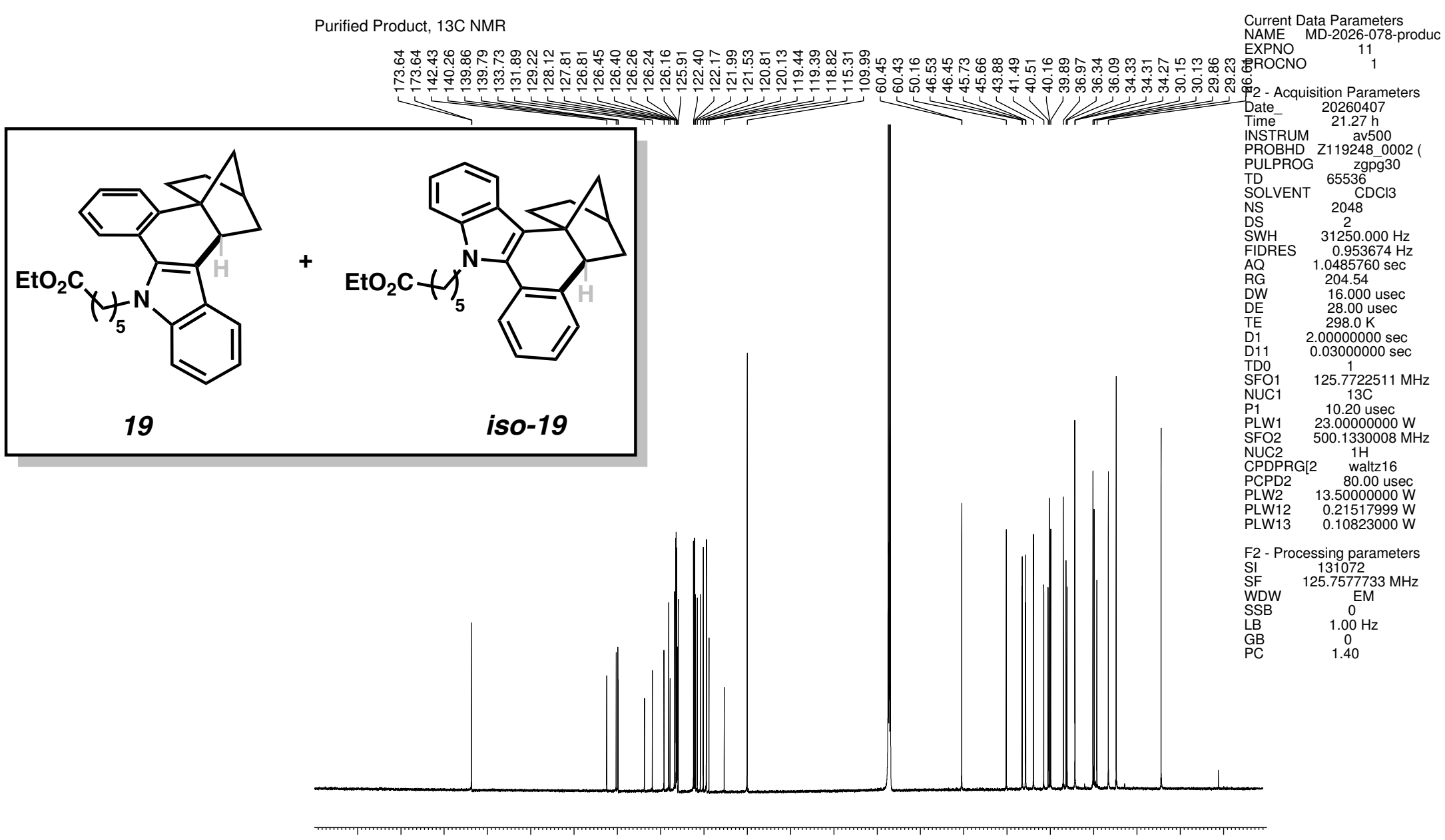

Figure S51. ^{13}C NMR (125 MHz, $CDCl_3$) of annulation products **19** and ***iso*-19**.

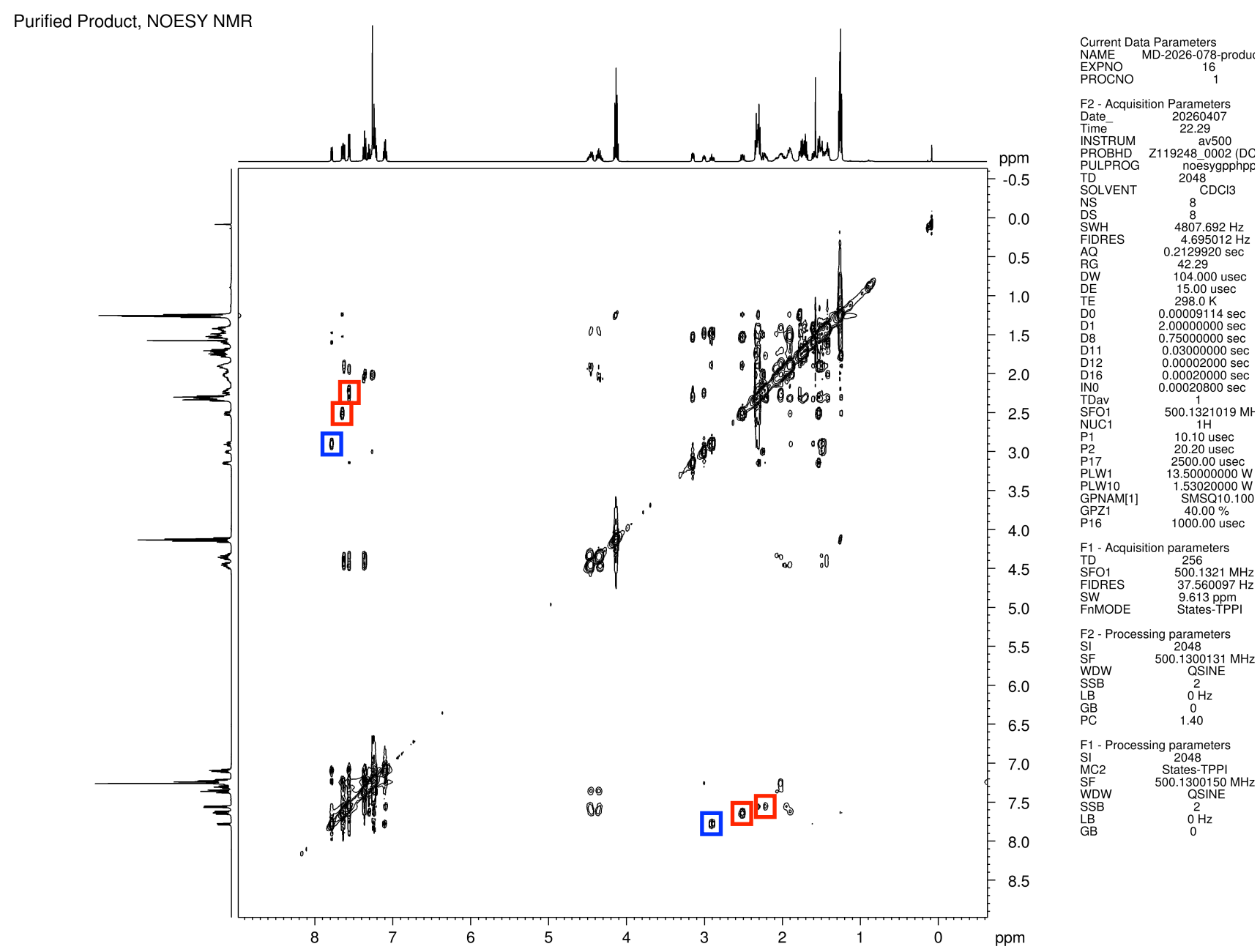

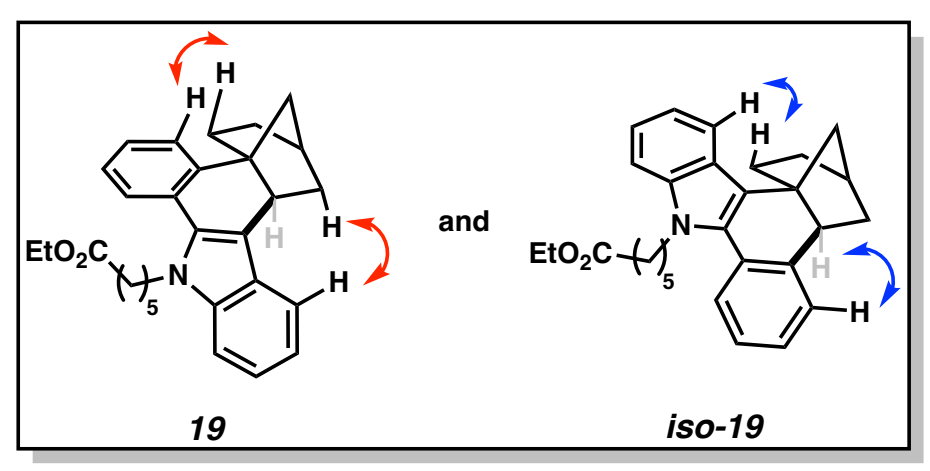

Figure S52. NOESY NMR (500 MHz, $CDCl_3$) of annulation products **19** and ***iso*-19**.

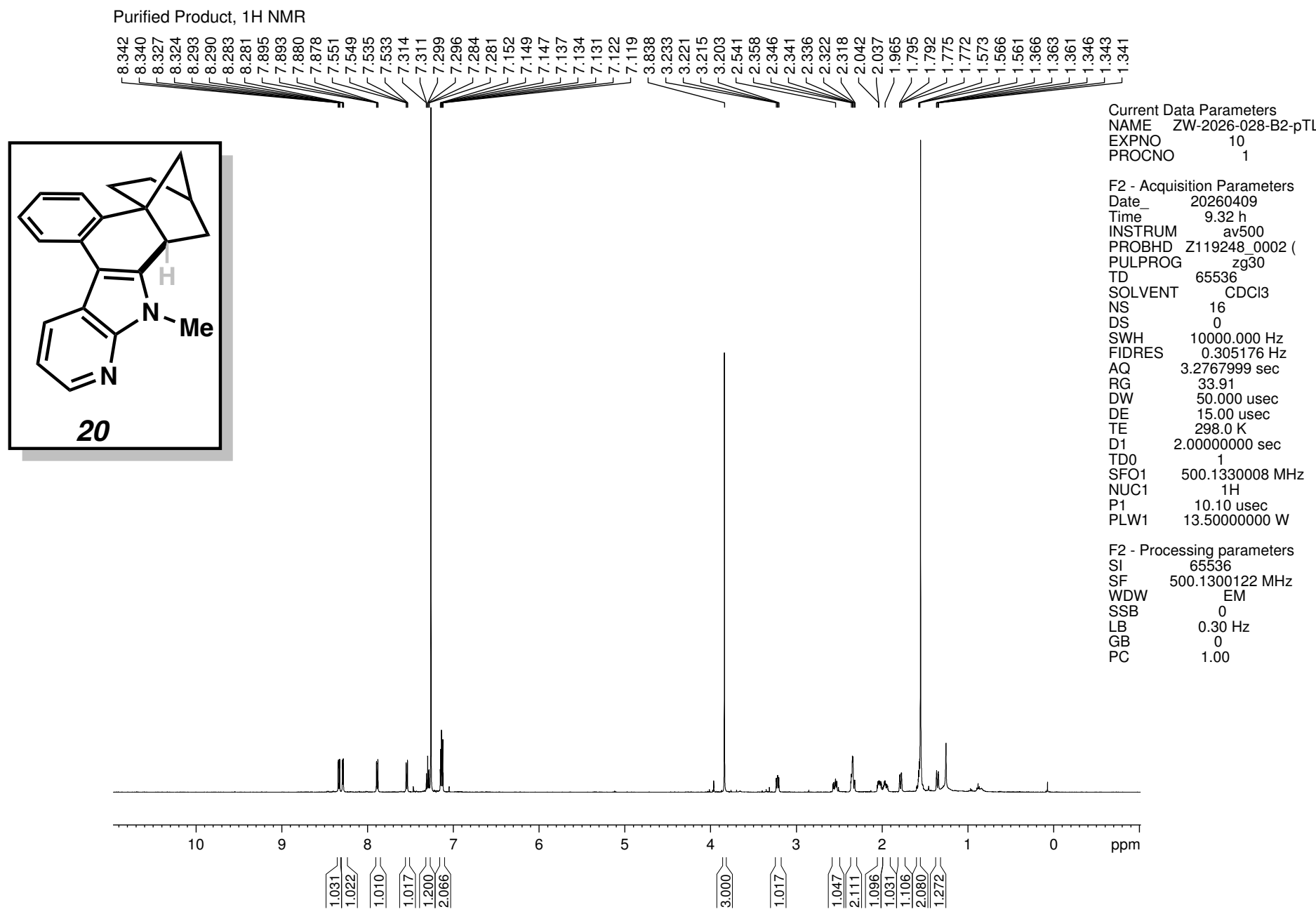

Figure S53. ^{1}H NMR (500 MHz, $CDCl_3$) of annulation product **20**.

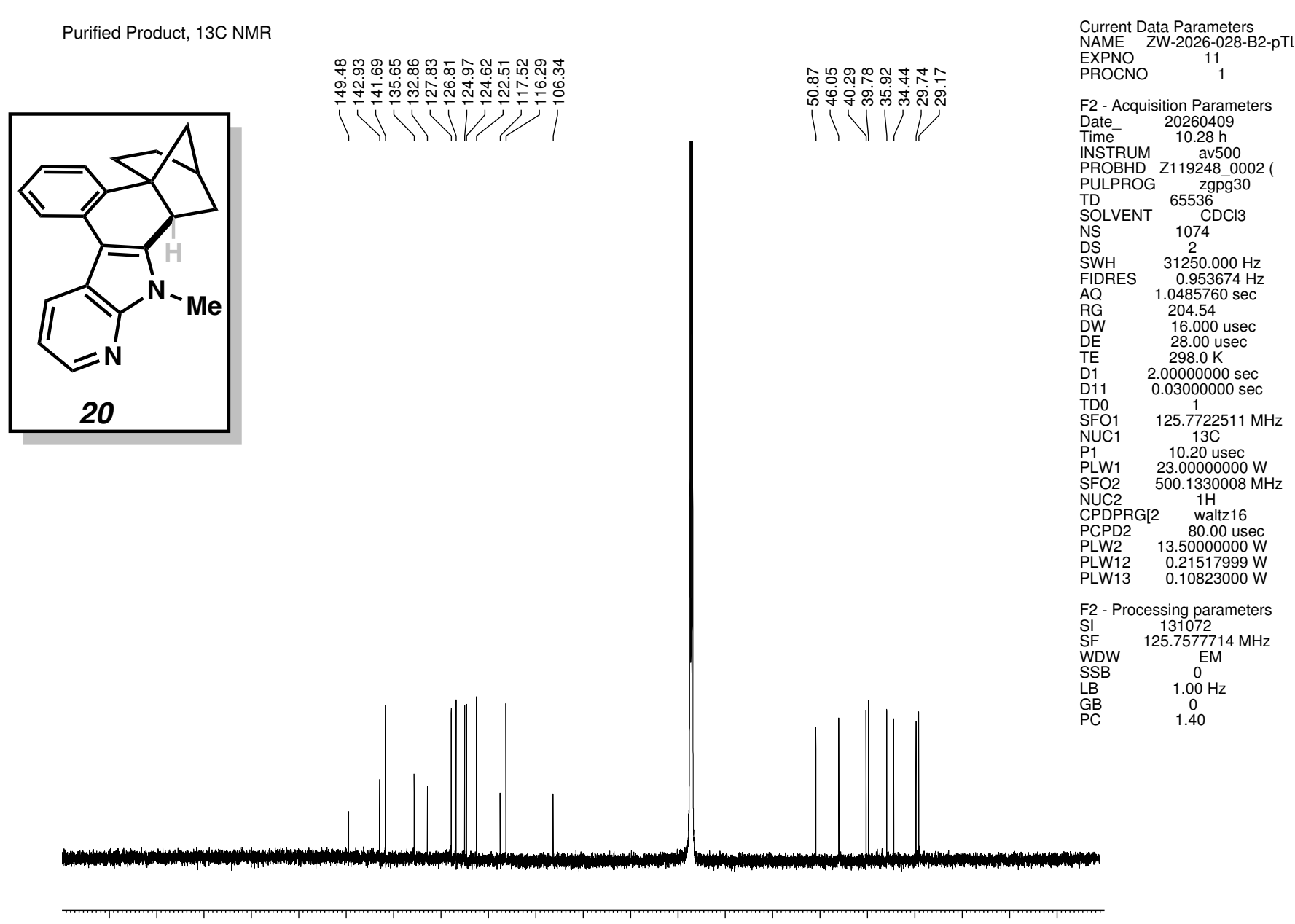

Figure S54. ^{13}C NMR (125 MHz, $CDCl_3$) of annulation product **20**.

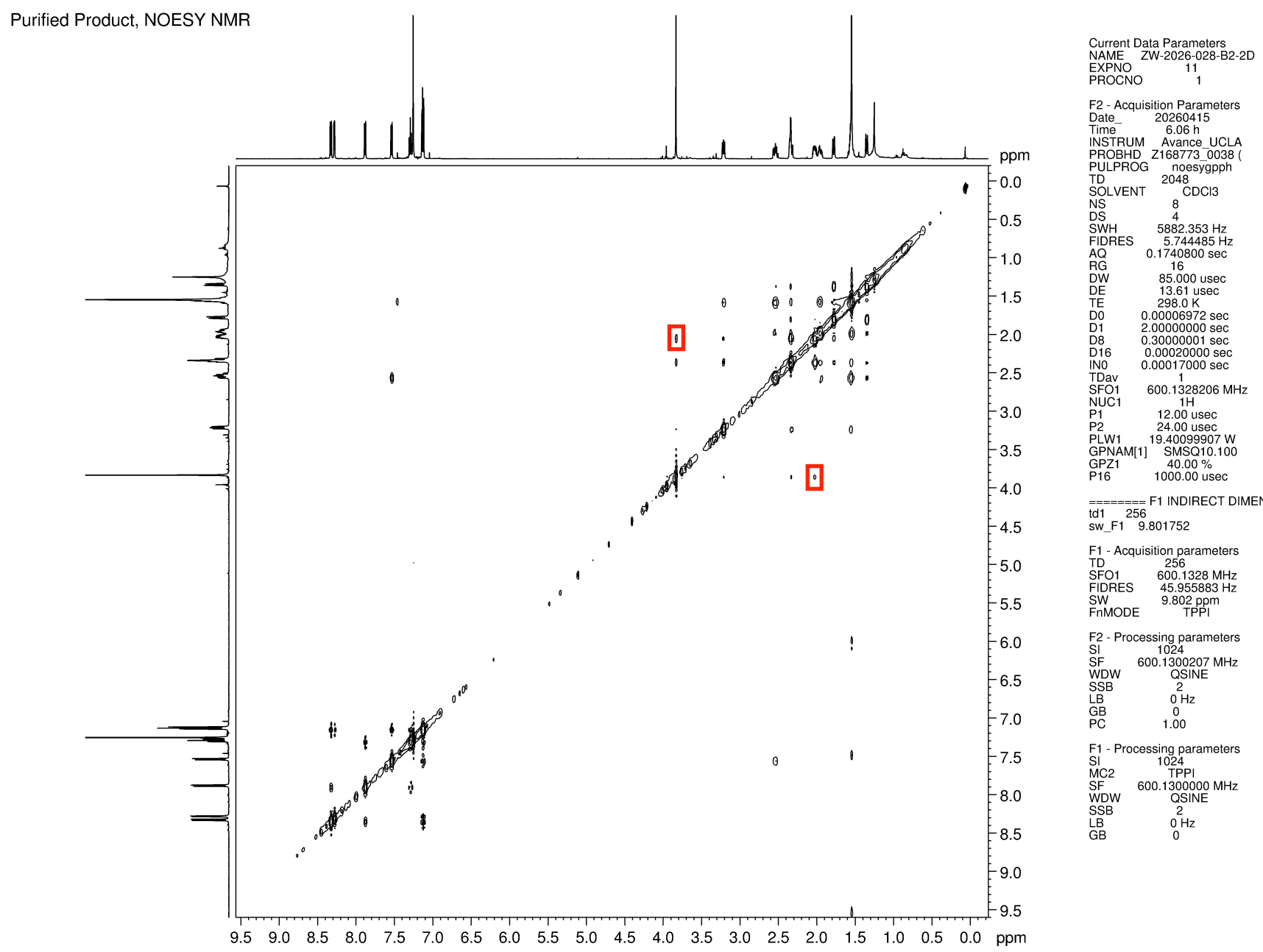

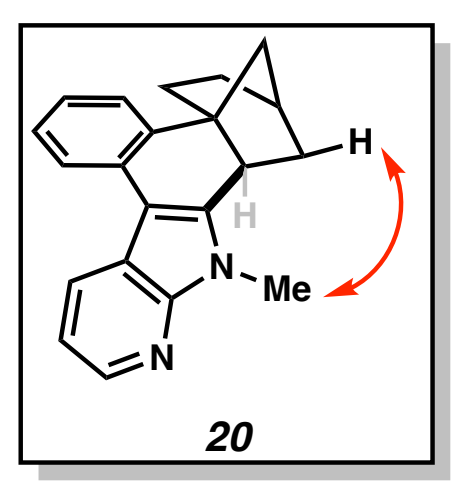

Figure S55. 2D NOESY (600 MHz, $CDCl_3$) of annulation product **20**.

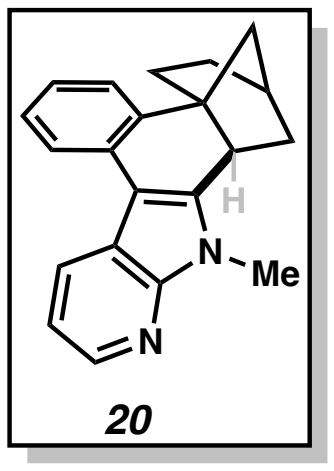

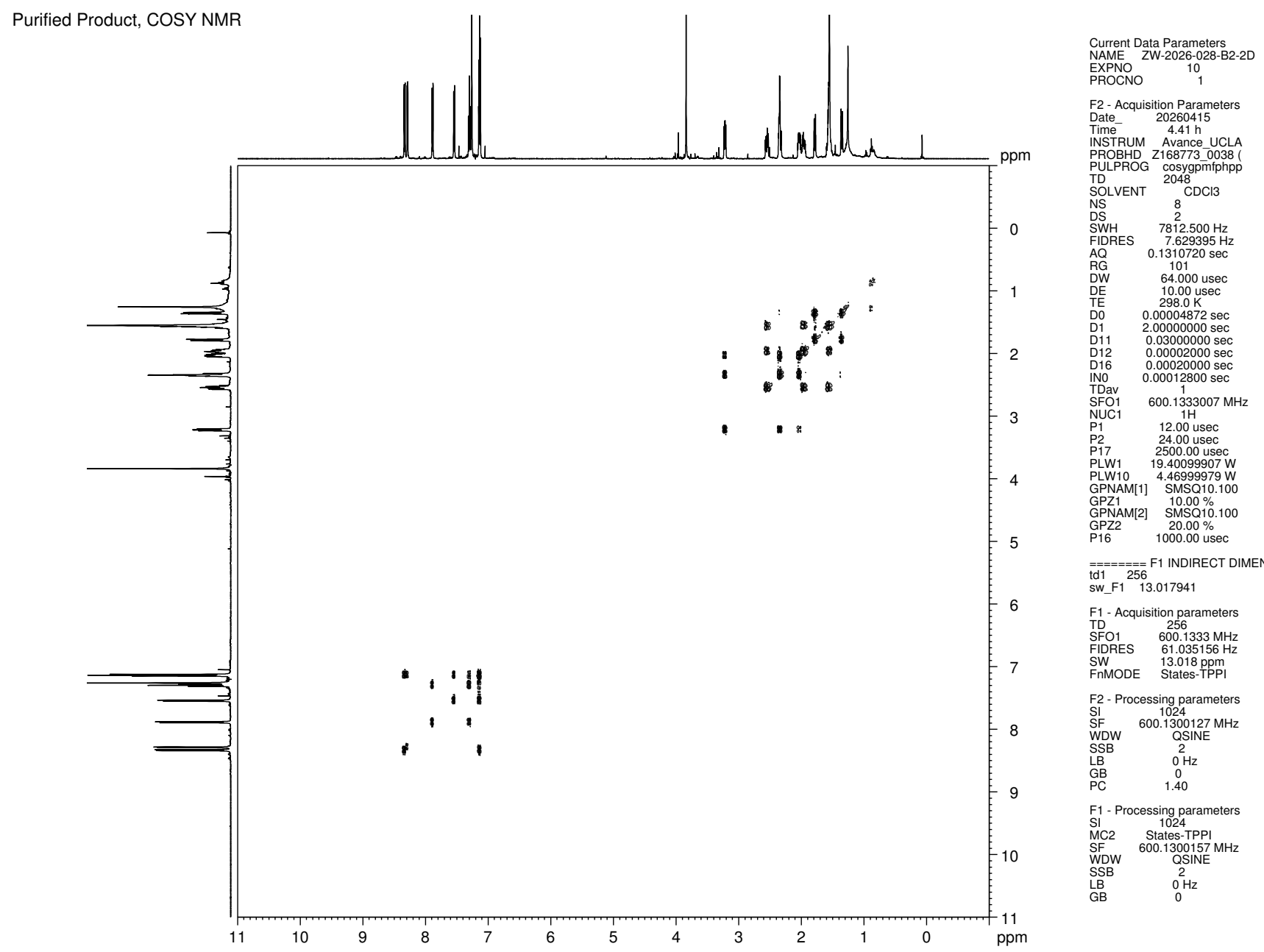

Figure S56. COSY (600 MHz, $CDCl_3$) of annulation product **20**.

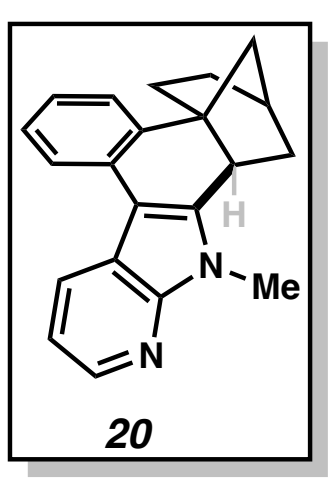

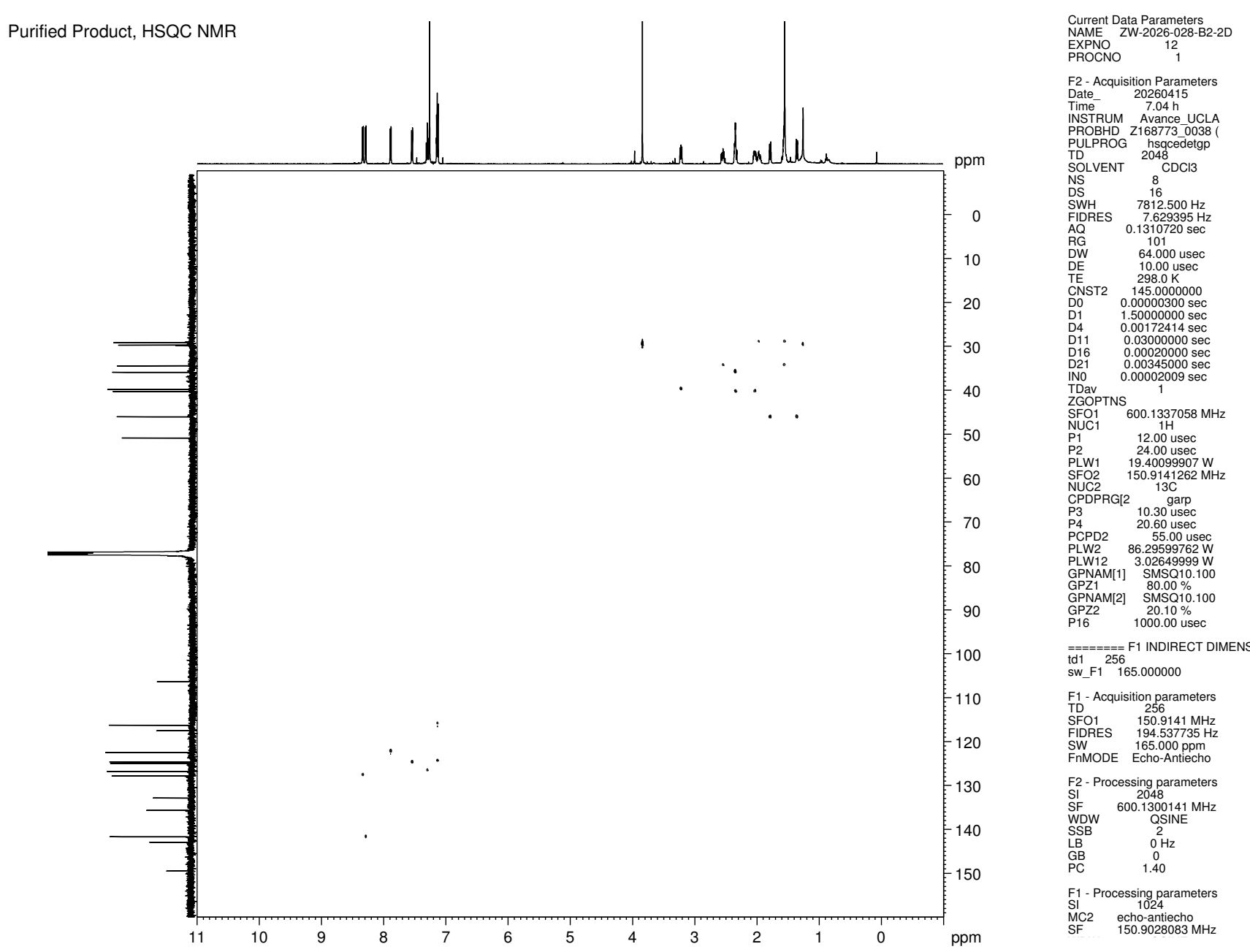

Figure S57. HSQC (600 MHz, $CDCl_3$) of annulation product **20**.

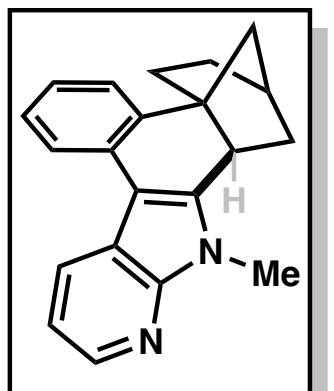

20

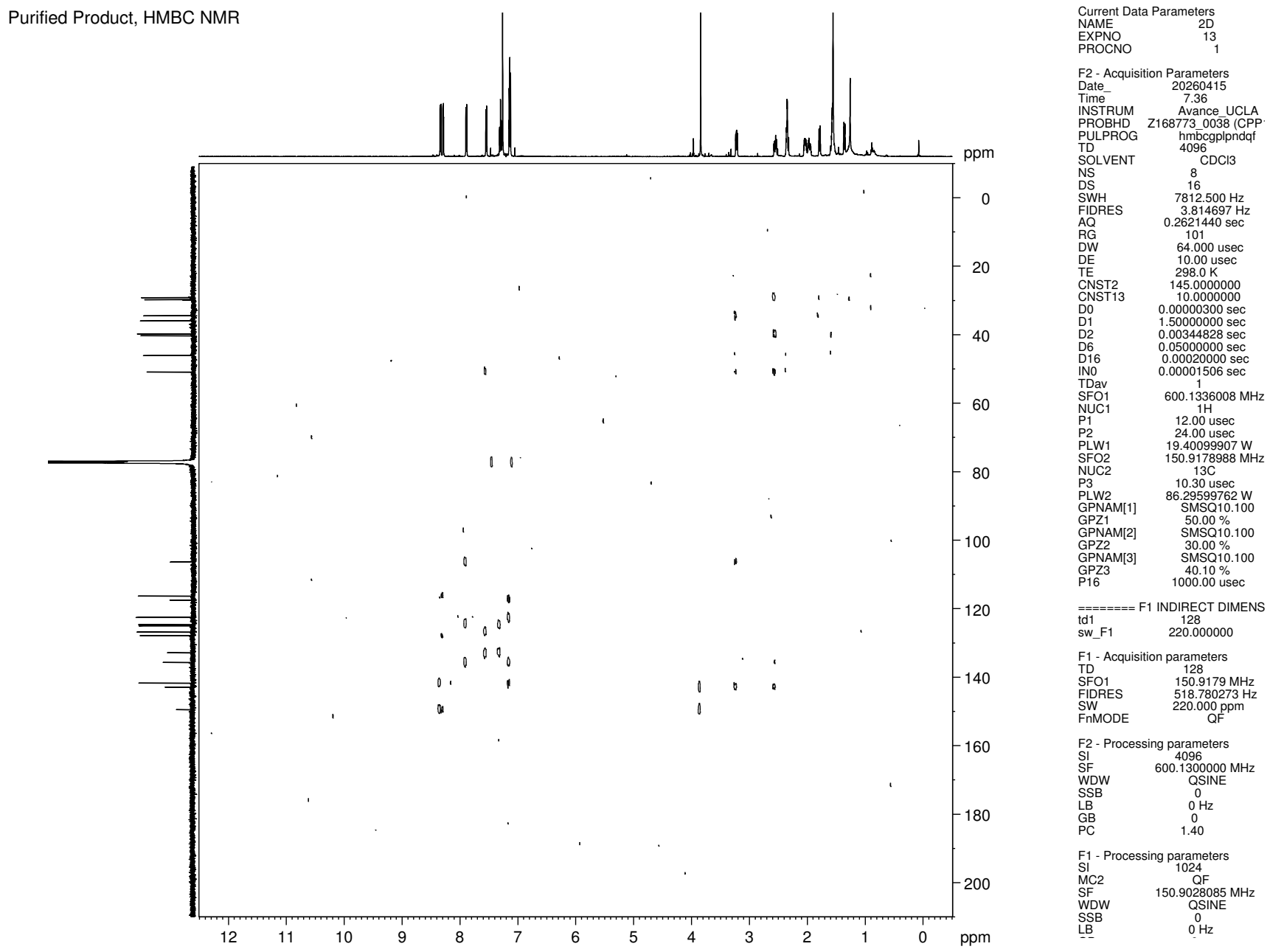

Figure S58. HMBC (600 MHz, $CDCl_3$) of annulation product **20**.

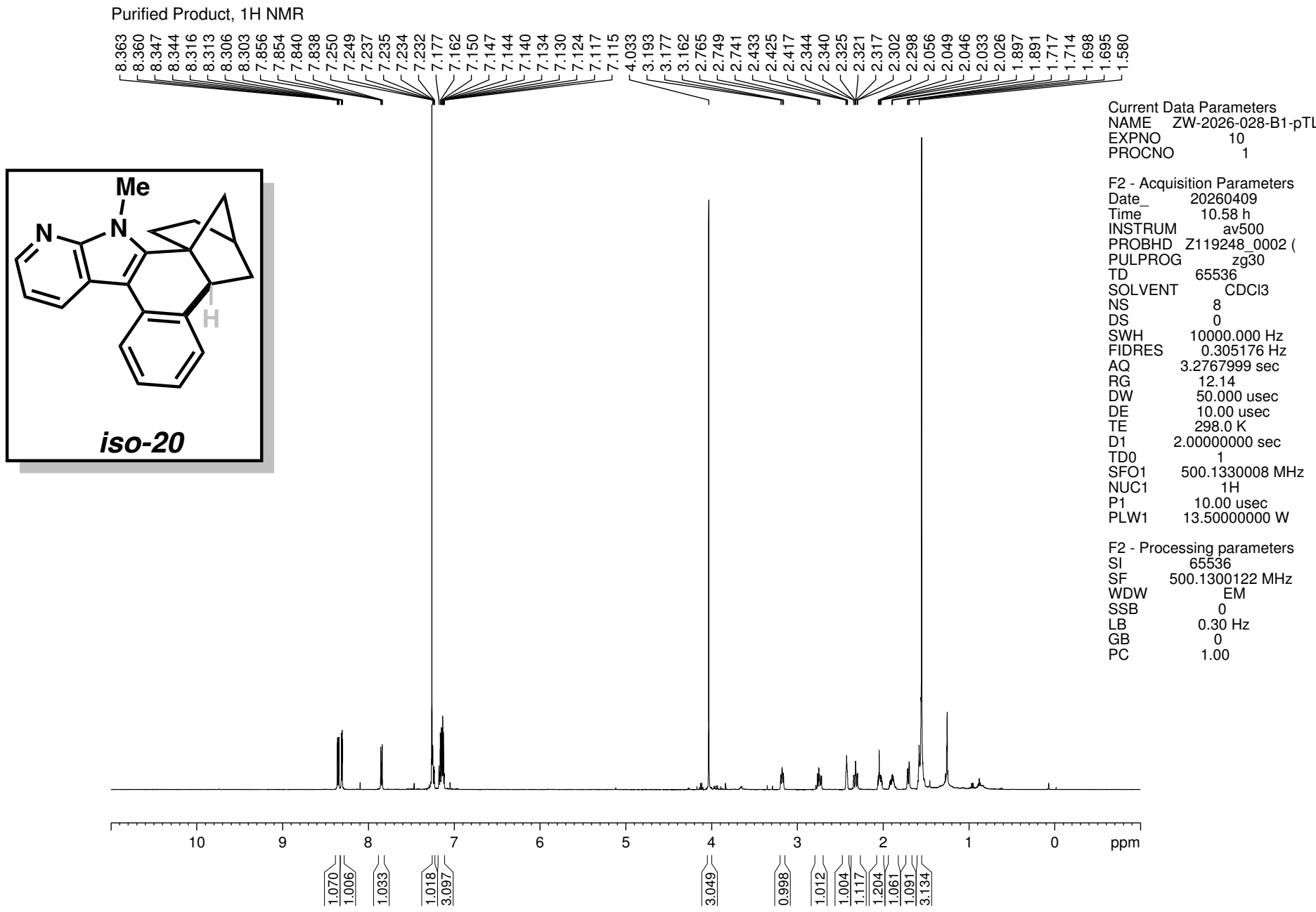

Figure S59. ^{1}H NMR (500 MHz, $CDCl_3$) of annulation product ***iso*-20**.

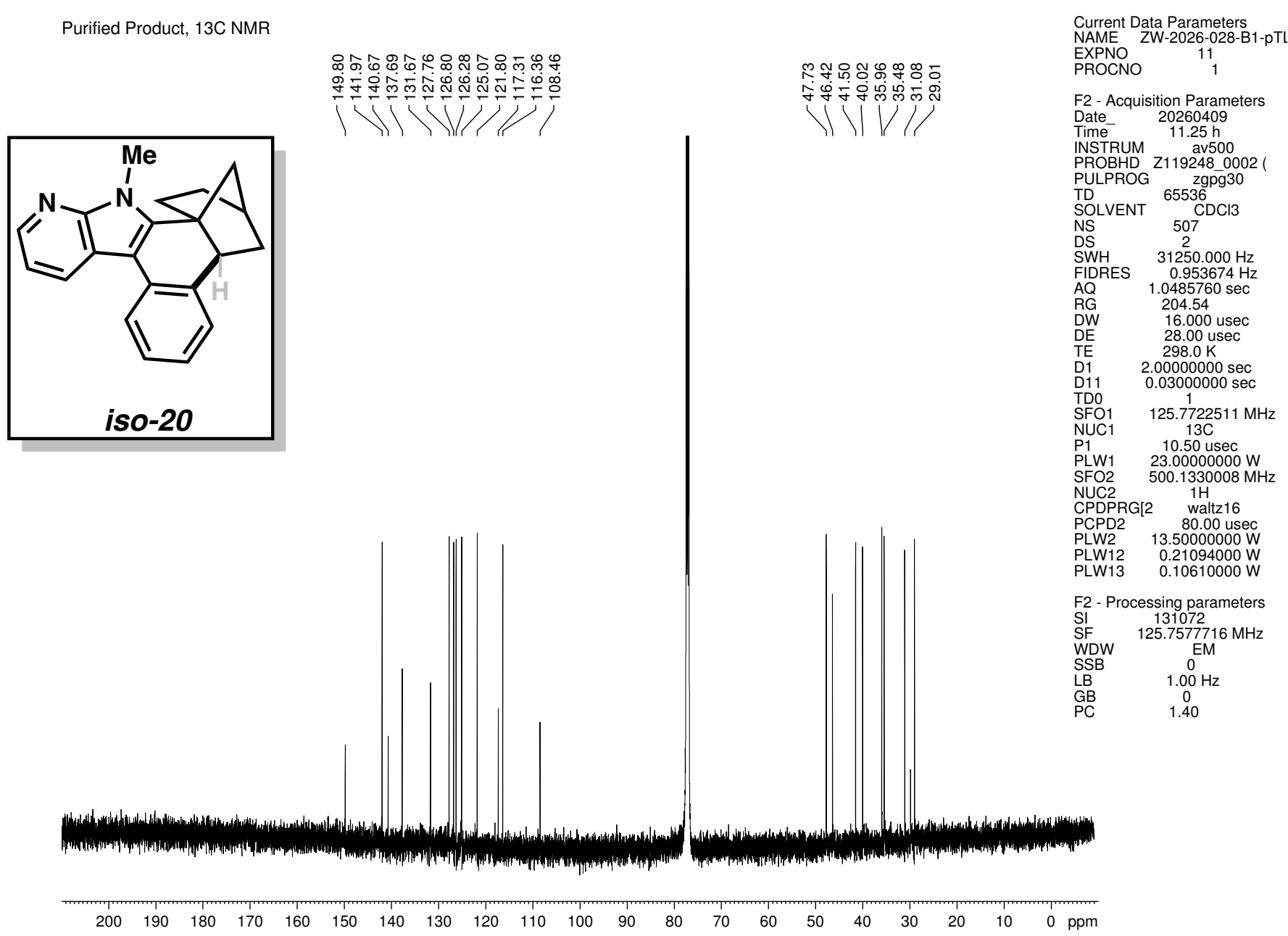

Figure S60. ^{13}C NMR (125 MHz, $CDCl_3$) of annulation product ***iso*-20**.

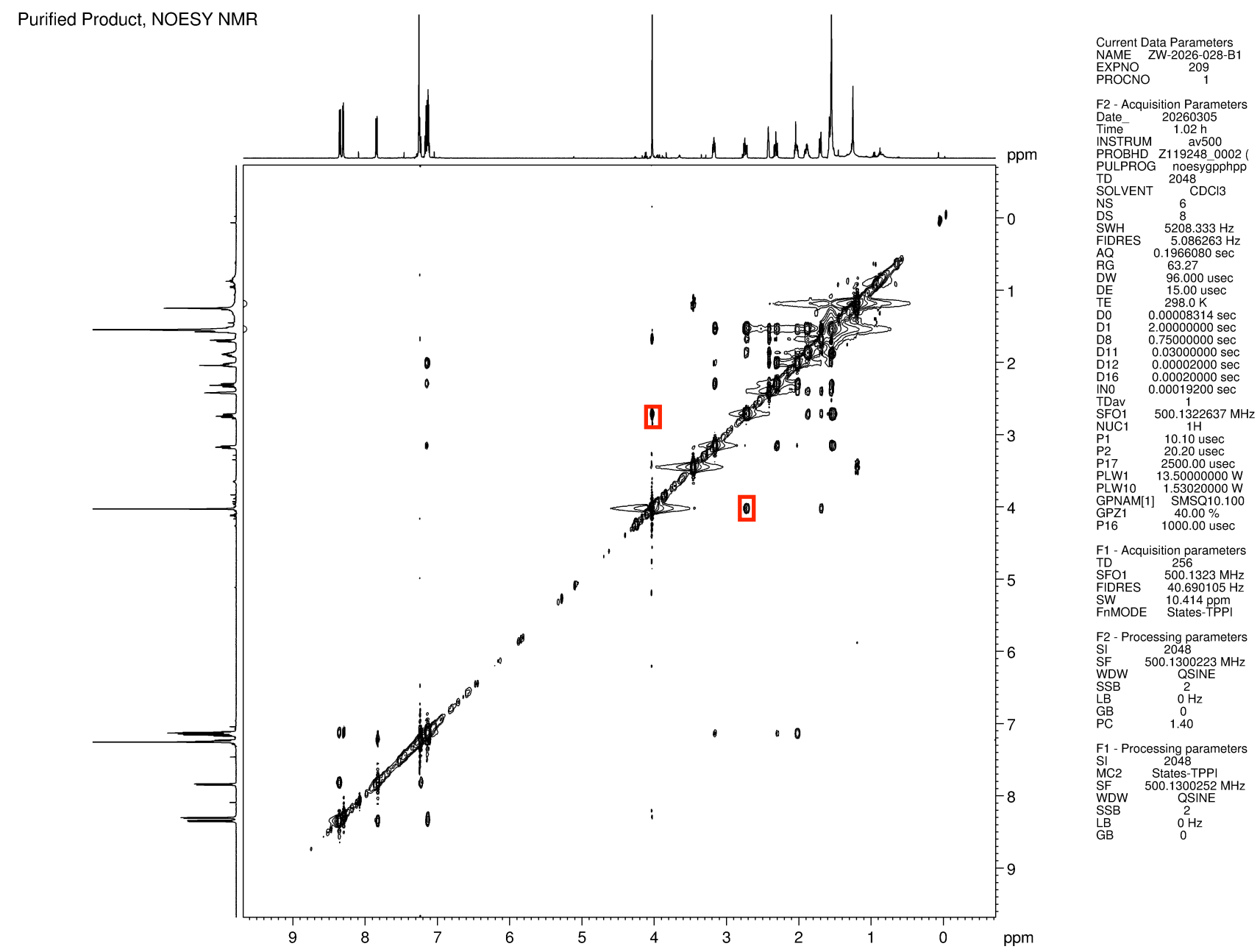

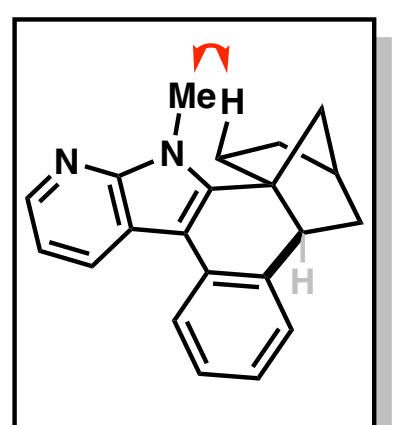

iso-20

Figure S61. 2D NOESY (500 MHz, $CDCl_3$) of annulation product ***iso*-20**.

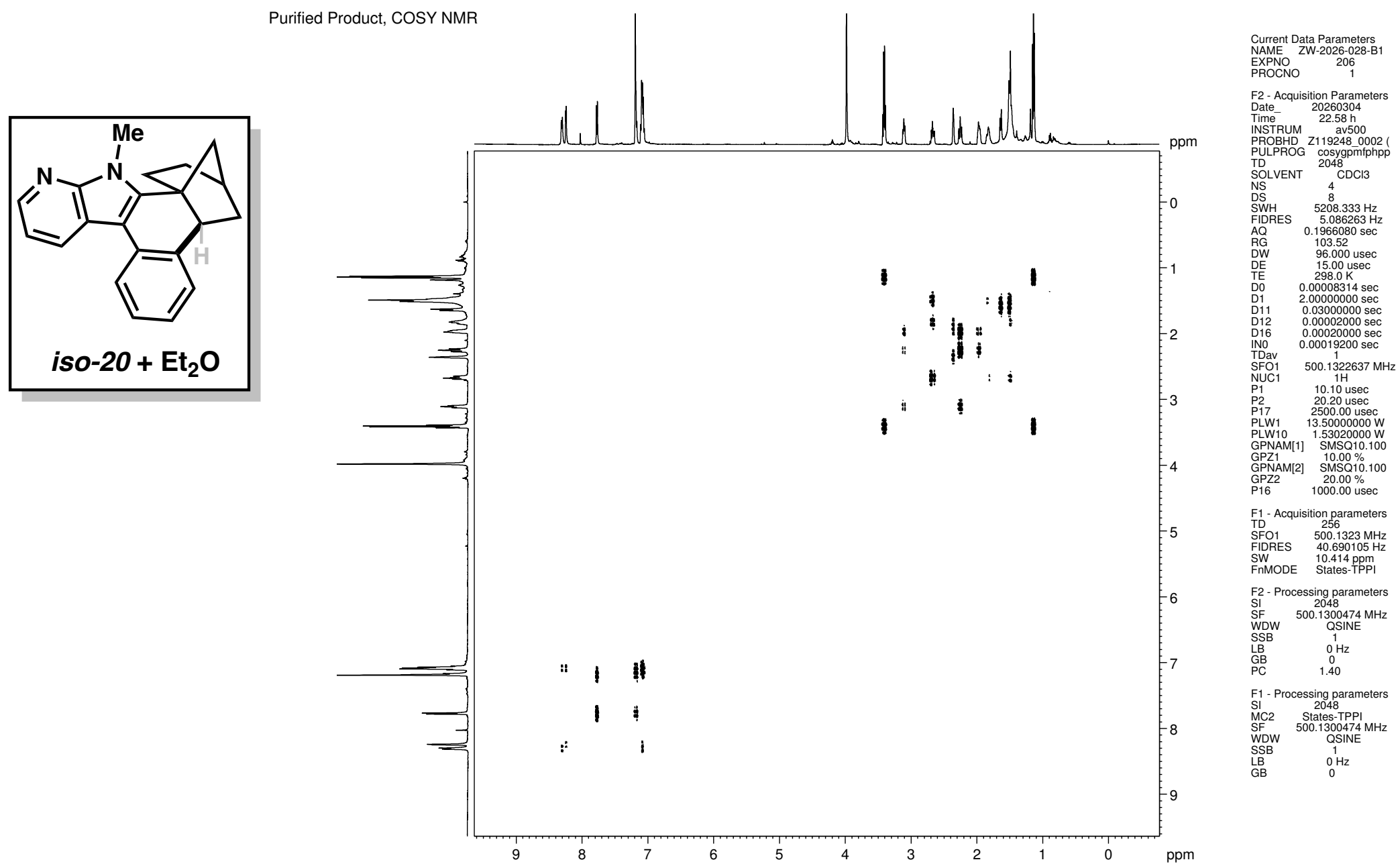

Figure S62. COSY (500 MHz, $CDCl_3$) of annulation product ***iso*-20**.

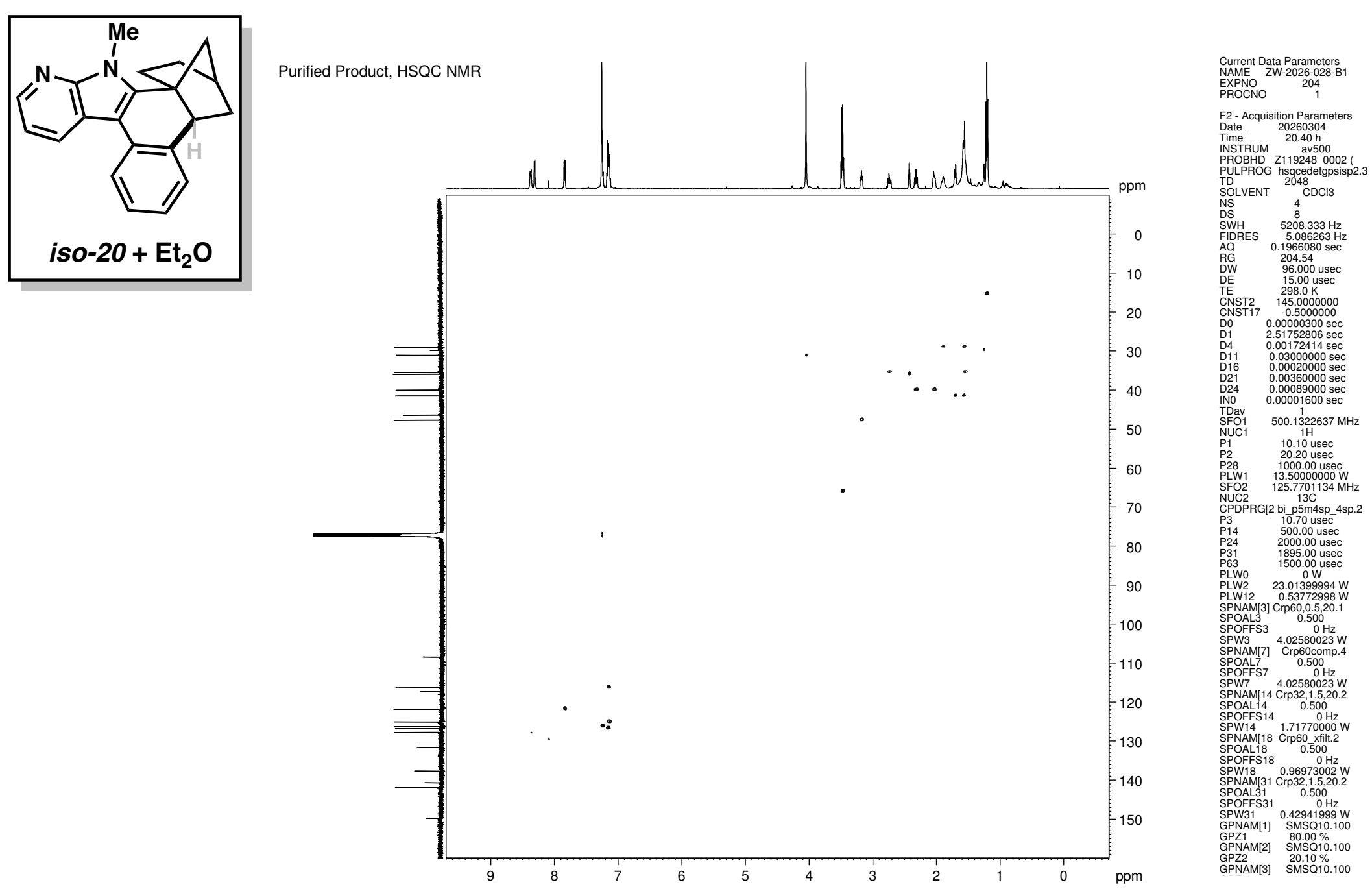

Figure S63. HSQC (500 MHz, $CDCl_3$) of annulation product ***iso*-20**.

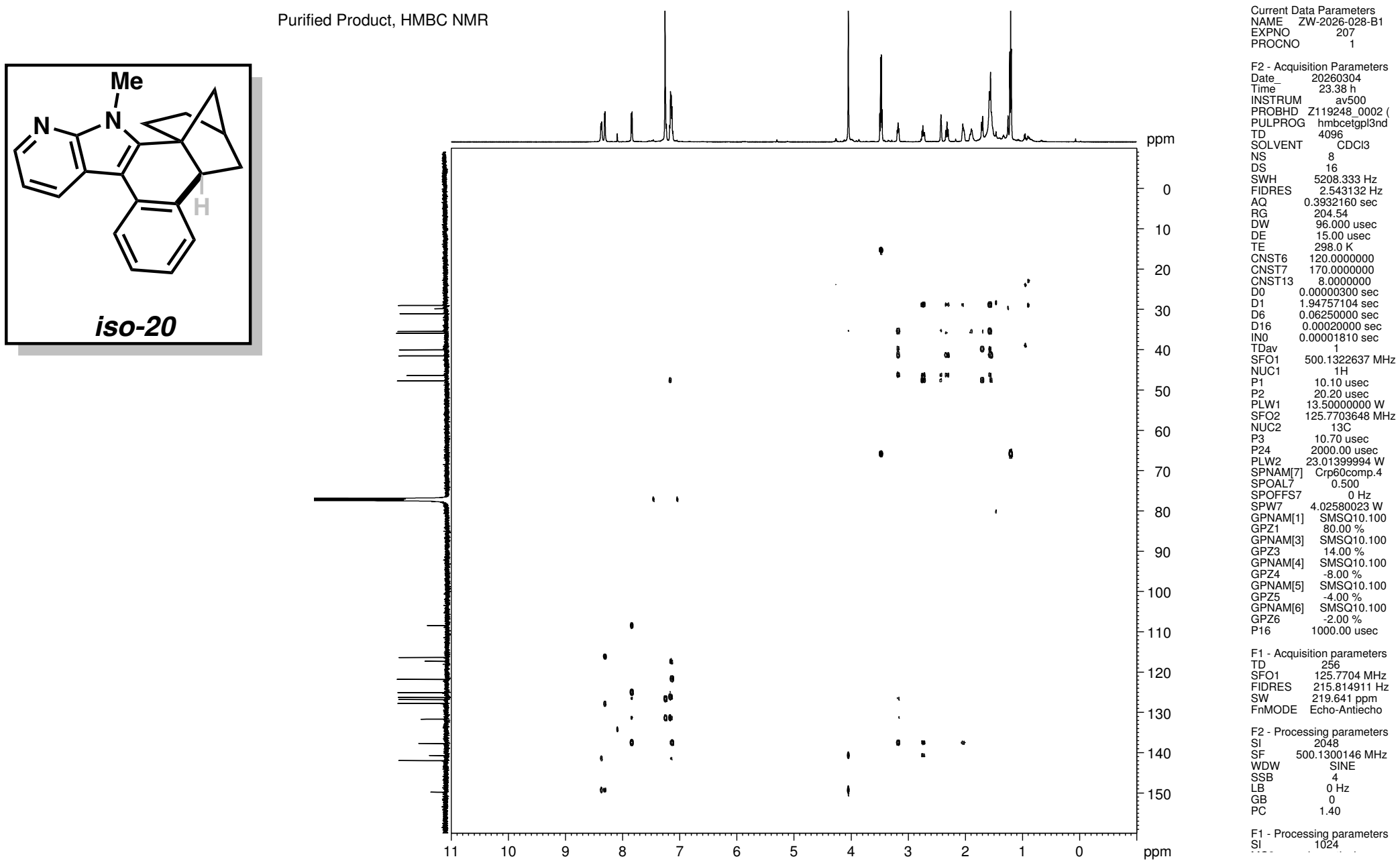

Figure S64. HMBC (500 MHz, $CDCl_3$) of annulation product ***iso*-20**.

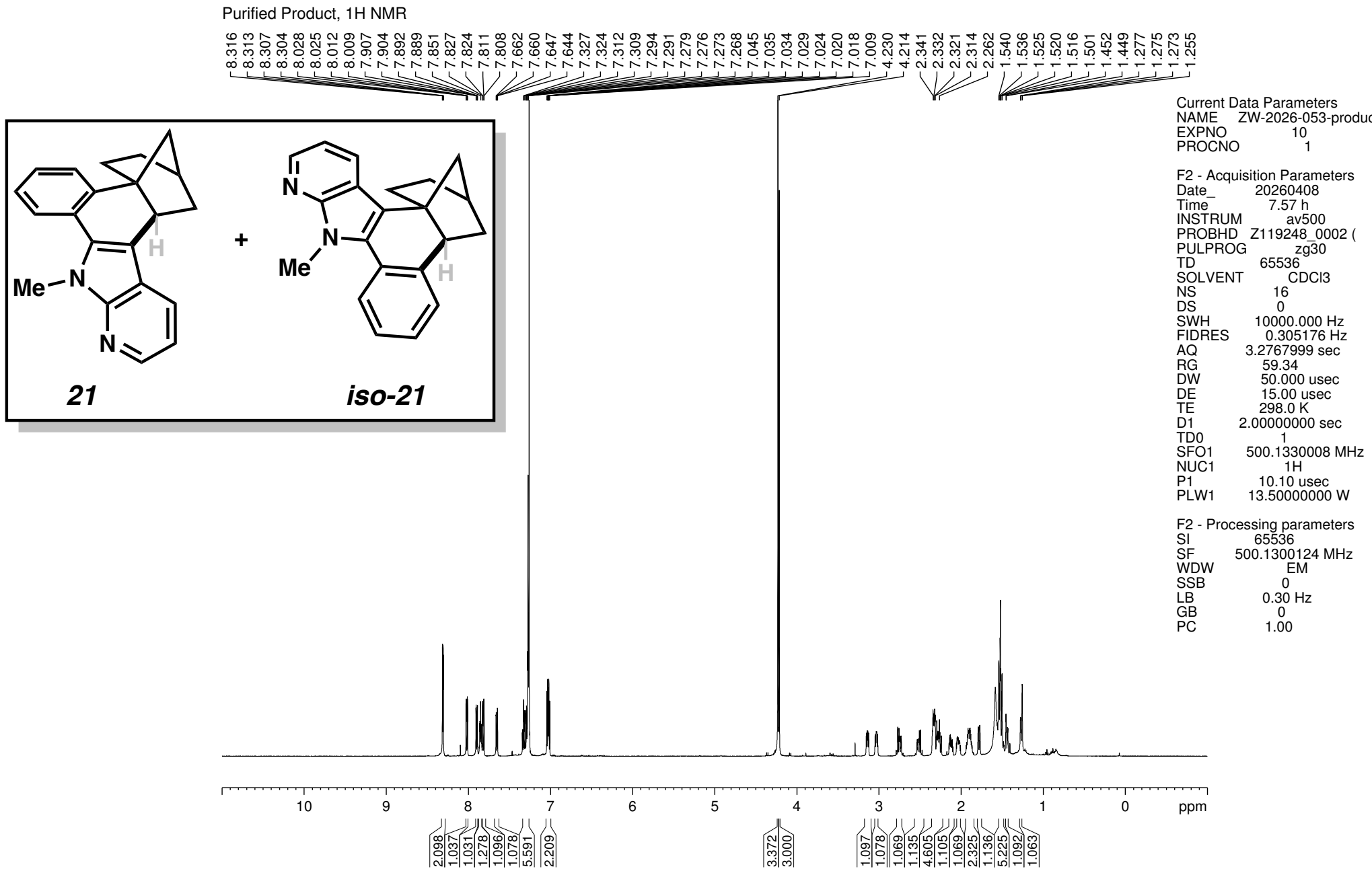

Figure S65. ^{1}H NMR (500 MHz, $CDCl_3$) of annulation products **21** and ***iso*-21**.

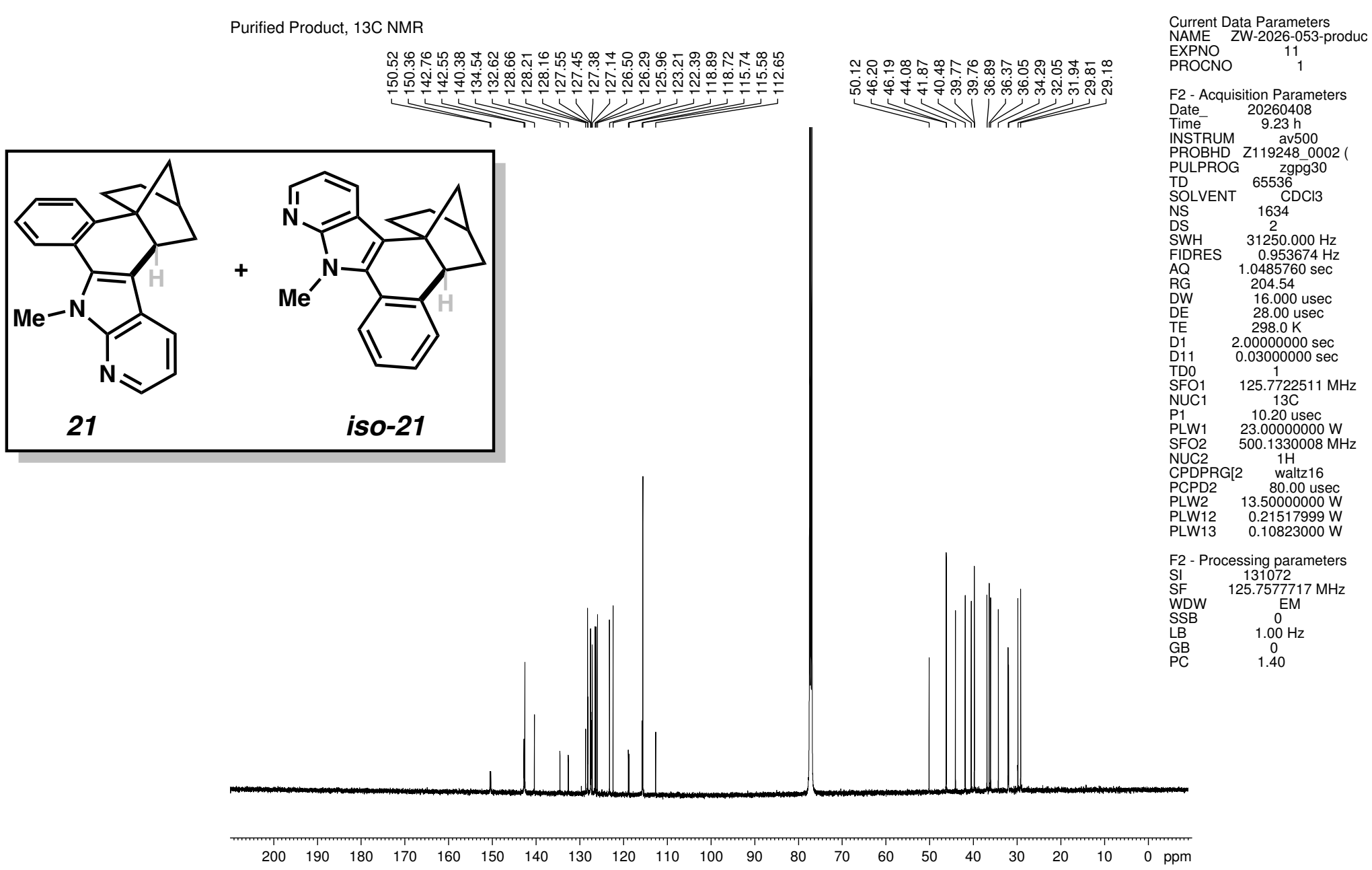

Figure S66. ^{13}C NMR (125 MHz, $CDCl_3$) of annulation products **21** and ***iso*-21.**

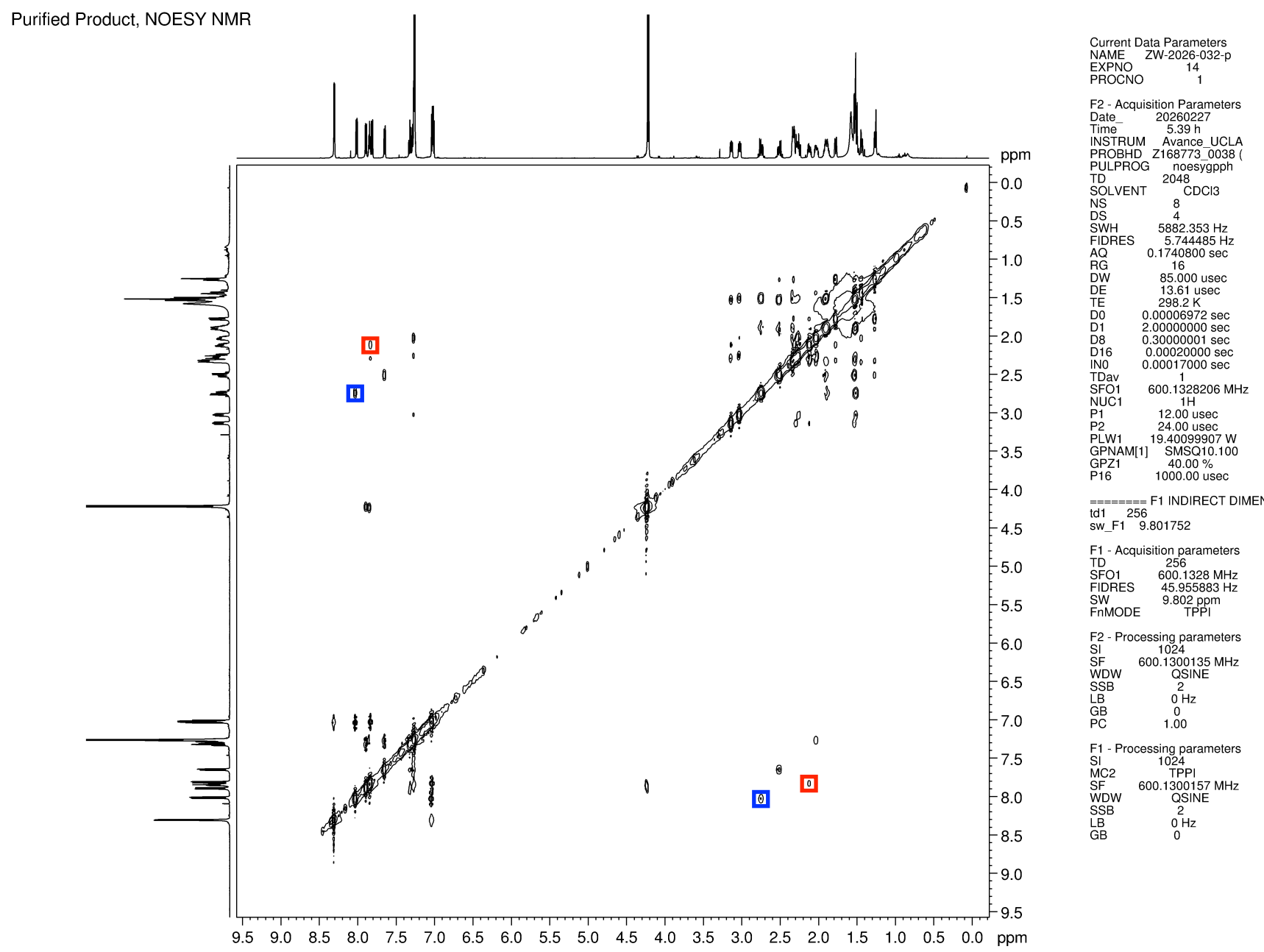

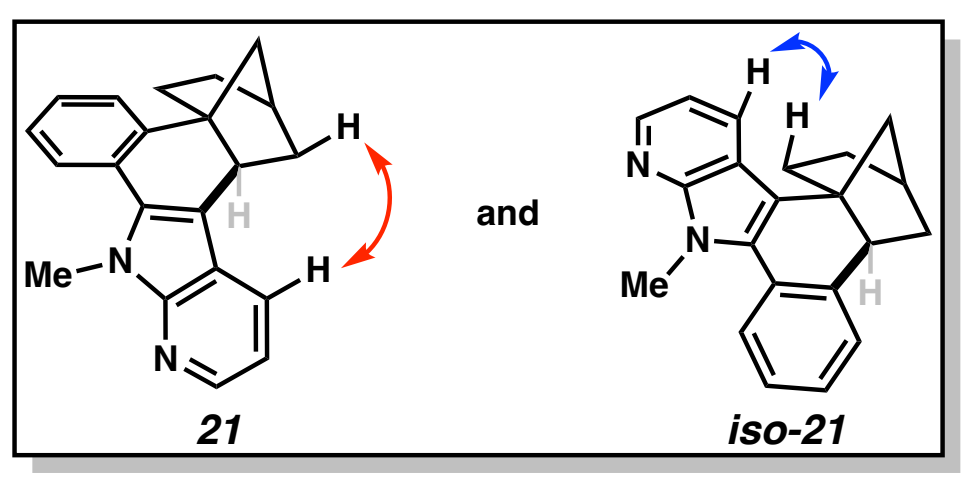

Figure S67. 2D NOESY (600 MHz, $CDCl_3$) of annulation products **21** and ***iso*-21**.

Part II. Computational Section

A. Computational Methods

Initial structures were prepared in Spartan '24 V1.2.0[13] and optimized using Molecular Mechanics. All DFT calculations were performed using Gaussian 16 (see Part II, section B for reference) or Spartan '24 V1.2.0. All geometries were verified as stationary points on the potential energy surface and characterized as minima by frequency calculations. GoodVibes[14] (v3.2) with quasi-harmonic entropy[15] and enthalpy[16] treatment (frequency cut-off value: 100 cm^{-1}) was used to obtain corrected Gibbs free energies and enthalpies at 298.15 K and 1 atm. All Gaussian calculations are performed on UCLA Hoffman2 supercomputers.

B. Complete Reference of Gaussian 16

Frisch, M. J.; Trucks, G. W.; Schlegel, H. B.; Scuseria, G. E.; Robb, M. A.; Cheeseman, J. R.; Scalmani, G.; Barone, V.; Petersson, G. A.; Nakatsuji, H.; Li, X.; Caricato, M.; Marenich, A. V.; Bloino, J.; Janesko, B. G.; Gomperts, R.; Mennucci, B.; Hratchian, H. P.; Ortiz, J. V.; Izmaylov, A. F.; Sonnenberg, J. L.; Williams-Young, D.; Ding, F.; Lipparini, F.; Egidi, F.; Goings, J.; Peng, B.; Petrone, A.; Henderson, T.; Ranasinghe, D.; Zakrzewski, V. G.; Gao, J.; Rega, N.; Zheng, G.; Liang, W.; Hada, M.; Ehara, M.; Toyota, K.; Fukuda, R.; Hasegawa, J.; Ishida, M.; Nakajima, T.; Honda, Y.; Kitao, O.; Nakai, H.; Vreven, T.; Throssell, K.; Montgomery, J. A., Jr.; Peralta, J. E.; Ogliaro, F.; Bearpark, M. J.; Heyd, J. J.; Brothers, E. N.; Kudin, K. N.; Staroverov, V. N.; Keith, T. A.; Kobayashi, R.; Normand, J.; Raghavachari, K.; Rendell, A. P.; Burant, J. C.; Iyengar, S. S.; Tomasi, J.; Cossi, M.; Millam, J. M.; Klene, M.; Adamo, C.; Cammi, R.; Ochterski, J. W.; Martin, R. L.; Morokuma, K.; Farkas, O.; Foresman, J. B.; Fox, D. J. *Gaussian 16, Revision C.01;* Gaussian, Inc., Wallingford, CT, 2016.

C. Details of Olefin Insertion Pathways

Several distinct mechanistic pathways could plausibly lead to the formation of products **8** or **iso-8**. Both involve initial formation of anti-Bredt olefin **2** through fluoride-promoted Kobayashi-type elimination of silyl nonaflate **7**, proceeding through the silicate shown, as well as oxidative addition involving the Pd(0) catalyst and the aryl iodide substrate **6**. These steps are shown in

Figure S68. From oxidative addition adduct **23**, two pathways are envisioned, which either involve formation of palladacycle **9**, which undergoes further reaction upon engaging ABO **2** (**Path A**) or direct engagement of the oxidative addition adduct **23** with ABO **2** (**Path B**).

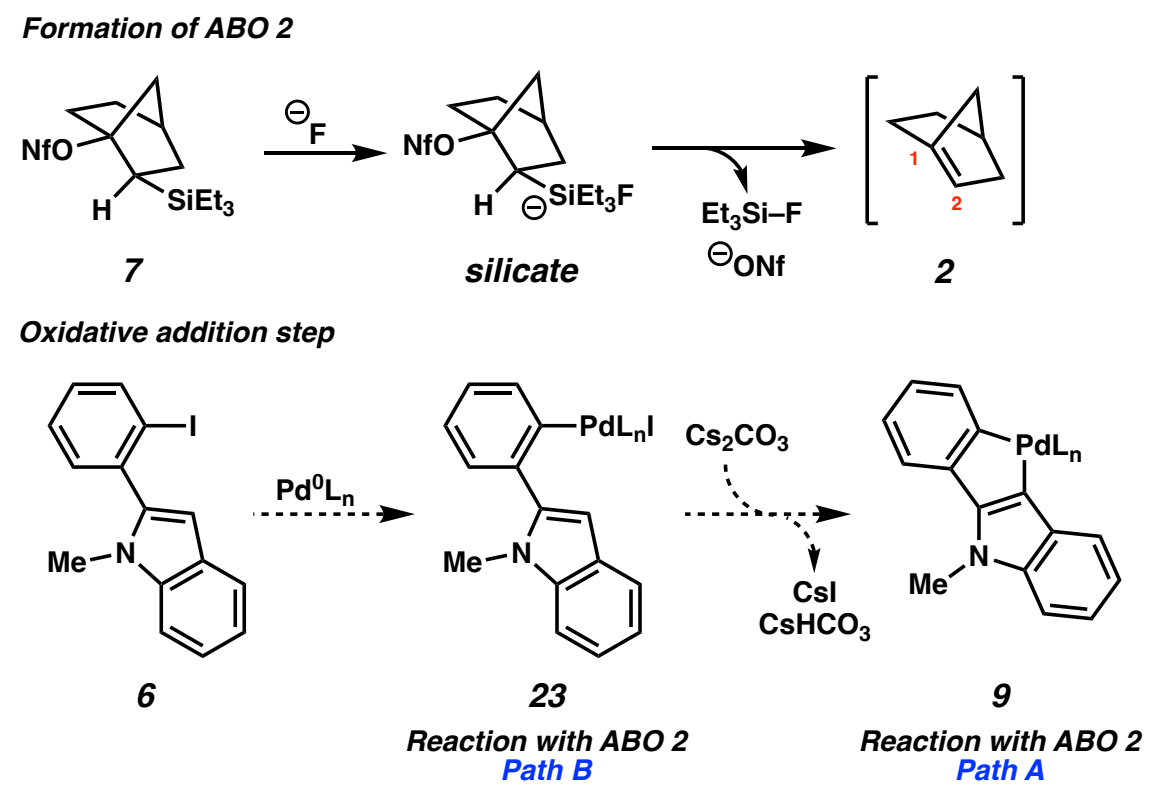

Figure S68. Proposed initial steps of reaction mechanism showing formation of ABO **2** and oxidative addition involving substrate **6**.

Further reaction via **Path A** is shown in Figure S69. This pathway is reasonable, especially given that palladacycle formation is known to readily occur in related systems.[17,18] The reaction of palladacycle **9** with ABO **2**, would proceed to give either complex **24** or **iso-24**, which differ based on the orientation of the ABO fragment relative to the palladacycle. We note that facial selectivity is guided by the bridged bicyclic framework, which permits Pd to approach from the external face of the bicycle, whereas approach from the opposite side, within the bicycle, is sterically prohibited.

Either intermediates **24** or **iso-24** can then undergo olefin insertion, with four possible paths available, which are labeled as Paths A-1, A-2, A-3, and A-4. In Path A-1, complex **24** undergoes olefin insertion, with Pd–C bond formation occurring at C1 of the ABO fragment, giving rise to palladacyclic intermediate **10a**. Alternatively, in Path A-2, Pd–C bond formation occurs at C2 of the ABO fragment, yielding **10b**. Following reductive elimination, both Paths A-1 and A-2 would lead to the major experimentally observed product, **8**.

Paths A-3 and A-4 are analogous, but stem from **iso-24**. Much like Path A-1, in Path A-3, initial Pd–C bond formation occurs at C1, which would furnish intermediate **iso-10a**. Finally, in Path A-4, C2 Pd–C bond formation would give **iso-10b**. Either of these paths would afford the minor product, **iso-8**, after reductive elimination. We intuitively favor the C1–Pd bond formation

pathways, Paths A-1 and A-3, as ABO **2** is more pyramidalized at C1. In cycloadditions, the C1 position of **2** typically reacts with the more electrophilic position of polarized, unsymmetrical traps.[2] However, the C2–Pd bond formation pathways, Paths A-2 and A-4, cannot be ruled out at this time.

DFT calculations were used to calculate the relative energies of the lowest energy conformers of the four possible palladacyclic intermediates (**10a, 10b, iso-10a, and iso-10b**). Although olefin insertion is likely irreversible and therefore expected to be kinetically controlled, we reasoned that a large thermodynamic difference among the resulting palladacycles might provide indirect support for one pathway over the others. However, the computed energies are closely comparable. Paths A-2 and A-4 (C_2–Pd bond formation) which lead to **10b** and **iso-10b**, were slightly more thermodynamically favorable, but the energy differences among the four palladacycles are small. As such, the calculations do not obviously exclude any of the four insertion pathways and therefore remain viable possibilities.

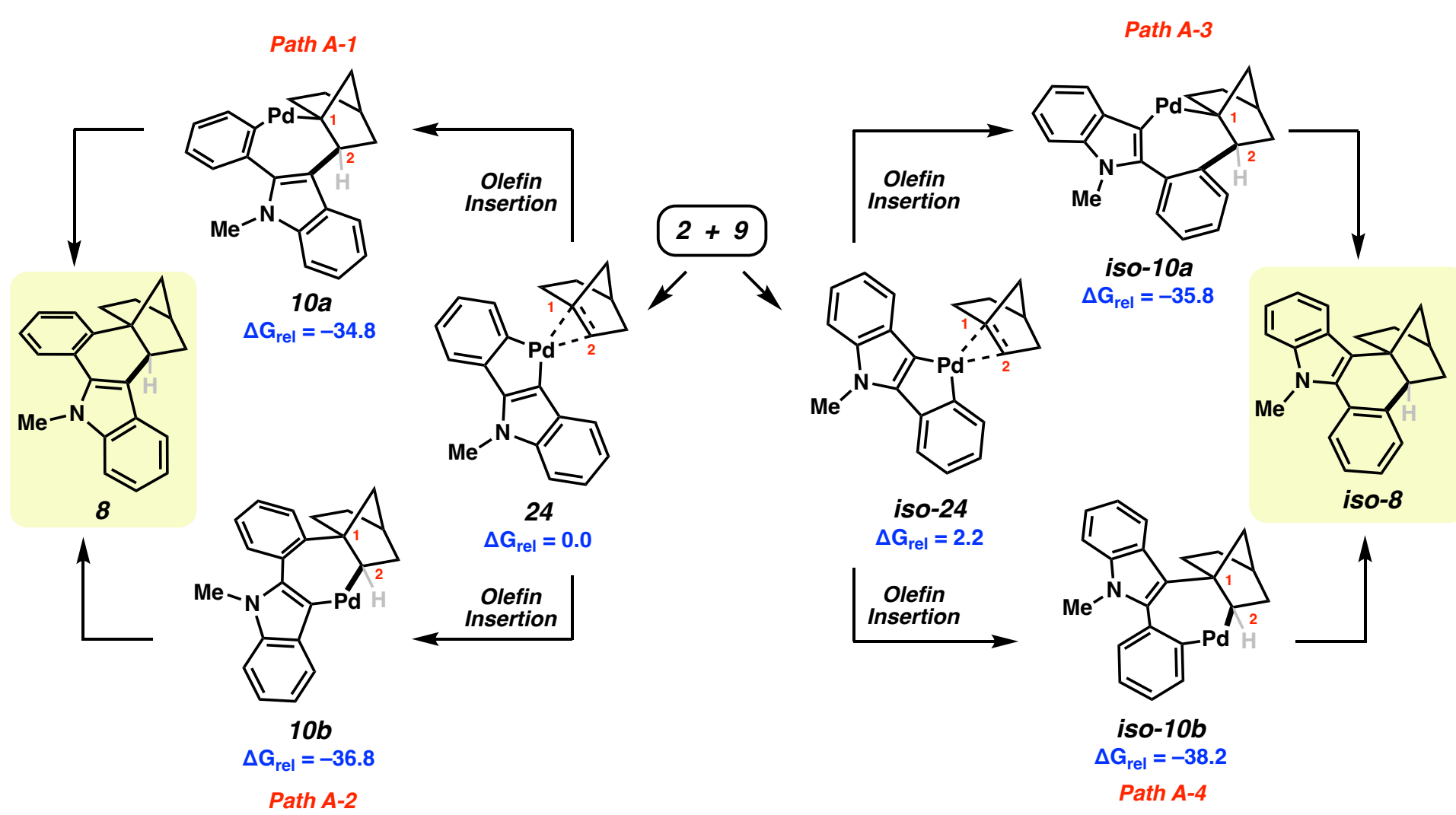

Figure S69. Plausible mechanism for the formation of **8** and **iso-8** via intermediate palladacycles **24** and **iso-24** (Path A). All units are in kcal/mol.

Structure	E_SPC	E	ZPE	H_SPC	T.S	T.qh-S	G(T)_SPC	qh-G(T)_SPC
**24**	-1033.717544	-1032.47847	0.371475	-1033.324565	0.072416	0.068737	-1033.396981	-1033.396503
***iso*-24**	-1033.713337	-1032.47608	0.371275	-1033.320396	0.073439	0.069404	-1033.393835	-1033.393095
**10a**	-1033.779321	-1032.559702	0.375504	-1033.383365	0.068395	0.066044	-1033.451759	-1033.452021
**10b**	-1033.782262	-1032.562146	0.375189	-1033.3867	0.068071	0.065812	-1033.454817	-1033.45516
iso-10a	-1033.78052	-1032.560792	0.375396	-1033.384652	0.068587	0.066184	-1033.453239	-1033.453496
***iso*-10b**	-1033.784425	-1032.563507	0.374982	-1033.3891	0.067829	0.065816	-1033.456959	-1033.457436

Table S7. Computed energies of lowest energy conformers of compounds **24**, ***iso*-24**, **10a**, **10b**, **iso-10a** and **iso-10b** at 25 °C at the M06-L/6-311++G(d,p)/SDD(Pd)//B3LYP-D3(BJ)/6-31G*/LANL2DZ(Pd) level of theory. All units are in Hartrees. E = electronic energy; SPC = single-point correction; ZPE = zero-point energy; H = enthalpy; T = temperature; S = entropy; G(T) = Gibbs free energy; qh-S = quasi-harmonic corrected entropy; qh-G(T) = quasi-harmonic corrected Gibbs free energy.

The aforementioned alternative reaction pathway involving the initial oxidative addition adduct **23** (**Path B**) is shown in Figure S70. In this scenario, ABO **2** would directly complex with **23**, giving rise to **53**. We envision that **53** could then undergo olefin insertion via Paths B-1 or B-2. In Path B-1, olefin insertion gives rise to C2–Pd bond formation in **54**, which following cyclization of the indole and reductive elimination, would furnish **8**. Alternatively, Path B-2 would proceed through C1–Pd bond formation and intermediate **iso-54. Iso-54** could then undergo an analogous indole cyclization and reductive elimination to yield **iso-8**. Lastly, we cannot rule out the possibility of complex **53** undergoing cyclization to intercept **24** or **iso-24**, interconnecting Paths A and B.

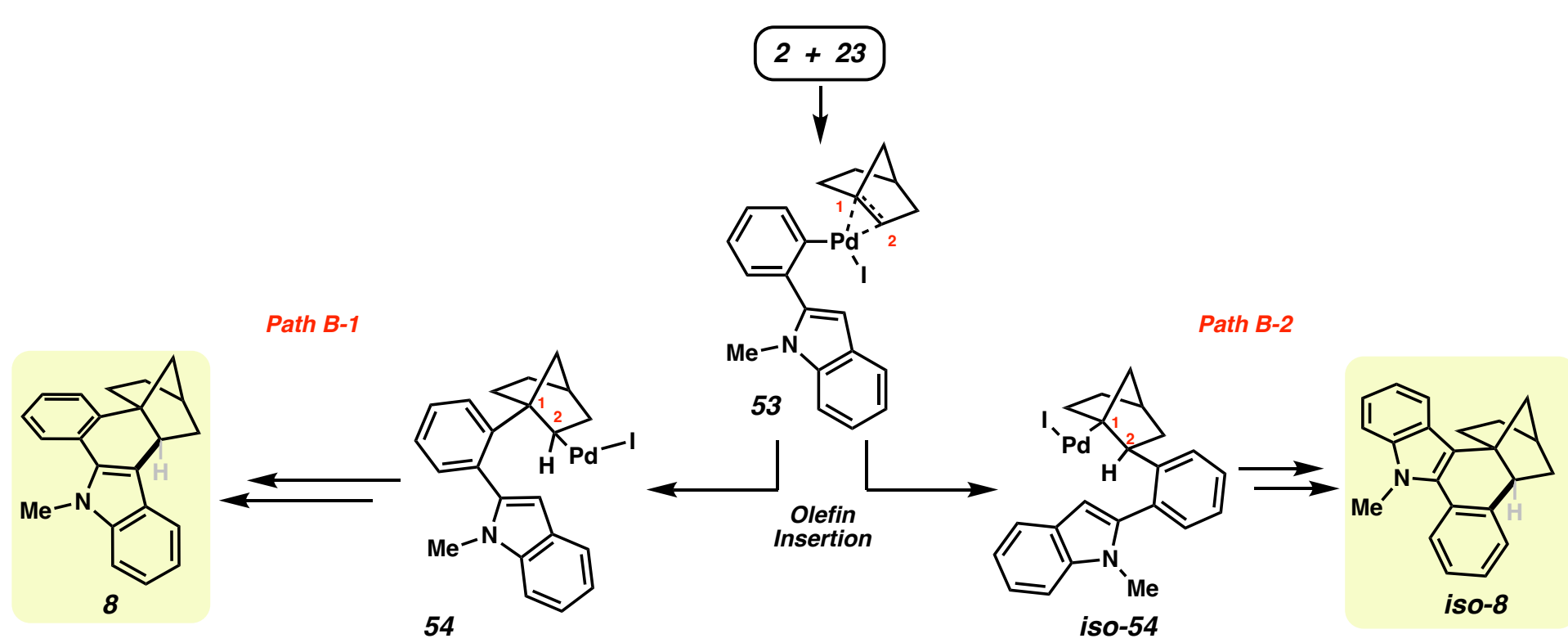

Figure S70. Plausible mechanism for the formation of **8** and **iso-8** via intermediate complexes **54** and **iso-54** (Path B).

Although it is challenging to obtain experimental support for any of these mechanistic scenarios due to the fleeting nature of **2**, we hope this discussion of plausible mechanisms will prompt further reaction design and mechanistic studies.

D. Comparison of Product Isomers 16 and iso-16 to Assess Steric Factors

It was envisioned that steric factors may play a role in the regioselectivity of annulations. However, in studying the reaction of substrate **35** (see Figure 3 in the manuscript), experimentally, poor regioselectivity for the formation of compounds **16** and **iso-16** was observed, despite the presence of the proximal *N*-Me group. More specifically, we presumed that compound **iso-16** would be thermodynamically less stable compared to **16**, due to steric interactions between the *N*-methyl group and the bicyclic scaffold. In order to probe this, the ground state geometries of **16** and **iso-16** were calculated at the M06-L/6-311++G(d,p)//B3LYP-D3(BJ)/6-31G* level of theory. As indicated in Figure S71, **iso-16** was found to be higher in energy by 2.1 kcal/mol. We presume that the impact of this steric interaction must be minor in the relevant transition state that ultimately leads to **iso-16**, leading to the observed lack of selectivity. However, as discussed in Section II.C, several mechanistic scenarios are viable, so it is challenging to unambiguously explain the lack of regioselectivity, including in cases where steric factors may have been expected to play a role.

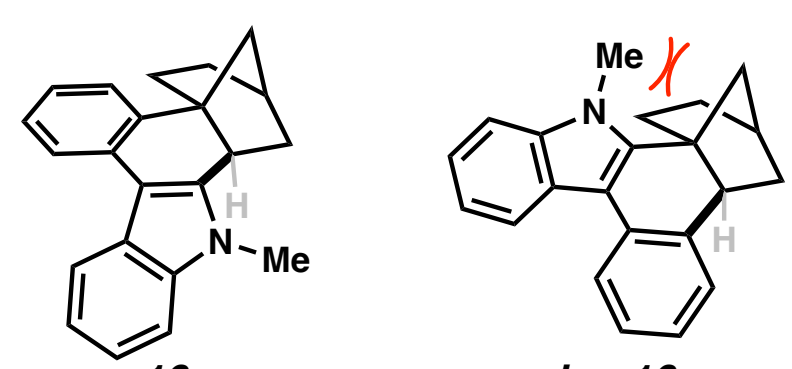

16
ΔG_{rel} = 0.0 kcal/mol

iso-16
ΔG_{rel} = 2.1 kcal/mol

Figure S71. Relative energies and predicted steric clash in products ***16*** and ***iso-16***.

Structure	E_SPC	E	ZPE	H_SPC	T.S	T.qh-S	G(T)_SPC	qh-G(T)_SPC
**16**	-905.86121	-905.86784	0.368443	-905.47438	0.062659	0.061205	-905.53704	-905.53745
**iso-16**	-905.85863	-905.86542	0.368809	-905.4716	0.062081	0.06068	-905.53368	-905.53403

Table S8. Computed energies of **16** and **iso-16** at 25 °C at the M06-L/6-311++G(d,p) //B3LYP-D3(BJ)/6-31G* level of theory. All units are in Hartrees. E = electronic energy; SPC = single-point correction; ZPE = zero-point energy; H = enthalpy; T = temperature; S = entropy; G(T) = Gibbs free energy; qh-S = quasi-harmonic corrected entropy; qh-G(T) = quasi-harmonic corrected Gibbs free energy.

E. Evaluation of Ligand Exchange

All complexes and ligands were rigorously conformer searched utilizing Crest V3.0.2[19] at the GFN2-xTB[20] level of theory. The geometries and energies of low energy conformers (<5 kcal/mol above lowest energy conformers) were refined by optimization at the B3LYP[21,22,23]-D3(BJ)[24]/6-31G*[25,26,27,28,29] level of theory, with the LANL2DZ[30] pseudopotential for Pd and I. Single point energies were calculated using M06-L[31]/6-311++G(d,p)[32,33,34,35] and the SDD[36] pseudopotential for Pd and I. The local exchange-correlation functional M06-L was chosen as it is recommended for thermochemistry calculations of transition metal-containing systems,[37] and has been utilized for calculations of transition metal-catalyzed reactions of strained intermediates.[38] The free energy of ligand exchange was calculated by comparing the lowest energy conformers of the palladium-ABO complex and the free ligand to the energies of the palladium-ligand complex and free ABO. Although other systems, such as four-coordinate Pd complexes could potentially be relevant, we propose that the systems modeled indicate the general trends and favorability of ligand exchanges involving ABOs.

F. Ligand Exchange in Cyclic Complex

The thermodynamics of ligand exchange between palladacyclic complexes **55**, **57**, and **59** with ABO **2** to give **24** were investigated (Figure S72A-C). See Figure 4B in the manuscript and associated discussion for further details.

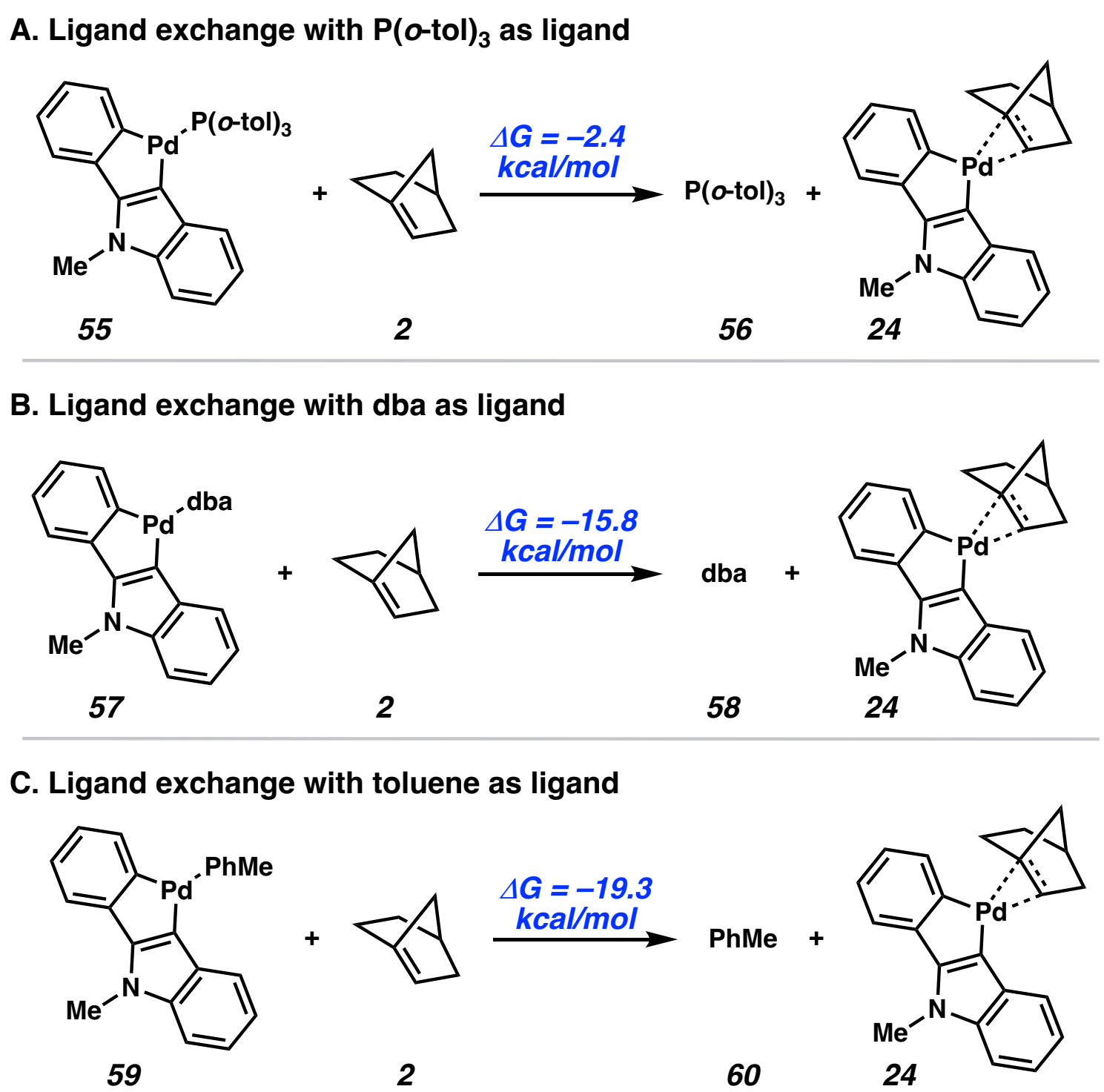

Figure S72. Energies of ligand exchange for the formation of **24**.

Structure	E_SPC	E	ZPE	H_SPC	T.S	T.qh-S	G(T)_SPC	qh-G(T)_SPC
**55**	-1915.36719	-1914.141854	0.5774	-1914.752945	0.106692	0.098578	-1914.859637	-1914.858229
**2**	-272.67946	-272.67735	0.152478	-272.520677	0.034912	0.034918	-272.555589	-272.555621
**56**	-1154.331846	-1154.346665	0.359221	-1153.95148	0.069805	0.066832	-1154.021285	-1154.021229
**24**	-1033.717544	-1032.47847	0.371475	-1033.324565	0.072416	0.068737	-1033.396981	-1033.396503
**57**	-1492.538684	-1491.28343	0.477629	-1492.029437	0.097211	0.089362	-1492.126648	-1492.124773
**58**	-731.523305	-731.507029	0.259267	-731.247753	0.062263	0.05868	-731.310017	-731.309116
**59**	-1032.596901	-1031.365896	0.346332	-1032.227701	0.076748	0.071794	-1032.304449	-1032.303688
**60**	-271.591541	-271.590818	0.128422	-271.455978	0.037729	0.037041	-271.493706	-271.493523

Table S9. Computed energies of lowest energy conformers of compounds Pd–P(*o*-tol)$_3$ complex **55,** ABO **2**, P(*o*-tol)$_3$ **56**, Pd–ABO complex **24**, Pd–dba complex **57**, dba **58**, Pd–PhMe complex **59**, and PhMe **60** at 25 °C at the M06-L/6-311++G(d,p)/SDD(Pd)//B3LYP-D3(BJ)/6-31G*/LANL2DZ(Pd) level of theory. All units are in Hartrees. E = electronic energy; SPC = single-point correction; ZPE = zero-point energy; H = enthalpy; T = temperature; S = entropy; G(T) = Gibbs free energy; qh-S = quasi-harmonic corrected entropy; qh-G(T) = quasi-harmonic corrected Gibbs free energy.

G. Ligand Exchange in Acyclic Oxidative Addition Complex

As we cannot rule out the involvement of an alternative process wherein oxidative addition complex **61** interacts directly with ABO **2**, the thermodynamics of ligand exchange between acyclic complexes **61**, **62**, and **63** with ABO **2** to give **53** were also investigated (Figure S73A-C). Ultimately, although absolute energetics differed, the trends and favorability of ABO ligation were similar to those depicted in Figure 4 and Section II.F. Additionally, analysis of the computed structure of ABO–Pd complex **53** showed that similar bonding interactions were present to those observed in complex **24**.

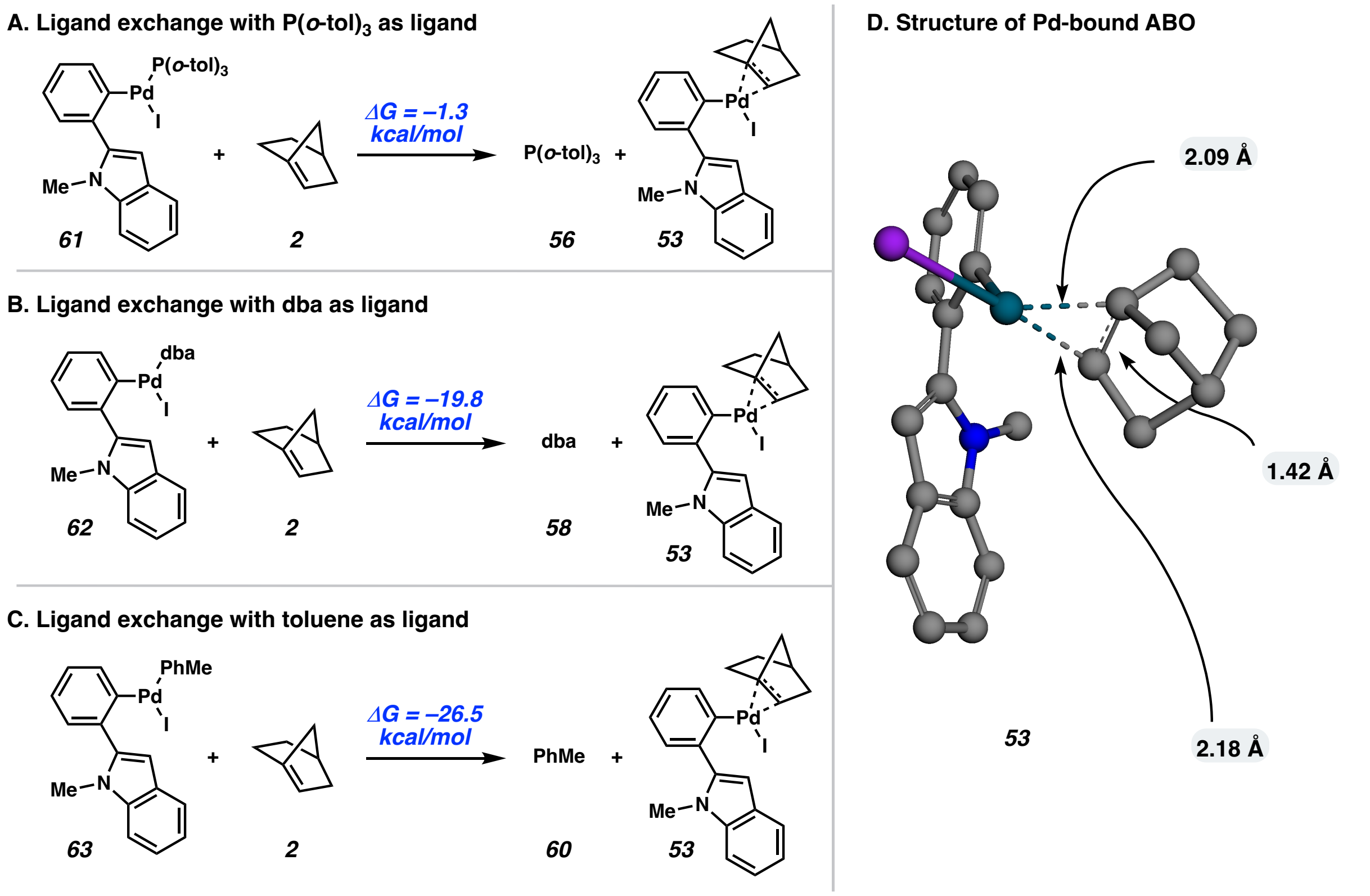

Figure S73. Energies of ligand exchange for the formation of **53**.

Structure	E_SPC	E	ZPE	H_SPC	T.S	T.qh-S	G(T)_SPC	qh-G(T)_SPC
**61**	-1927.465103	-1926.230317	0.591284	-1926.834903	0.110432	0.102661	-1926.945336	-1926.944822
**2**	-272.67946	-272.67735	0.152478	-272.520677	0.034912	0.034918	-272.555589	-272.555621
**56**	-1154.331846	-1154.346665	0.359221	-1153.95148	0.069805	0.066832	-1154.021285	-1154.021229
**53**	-1045.809769	-1044.556441	0.384322	-1045.401291	0.081777	0.075565	-1045.483067	-1045.481309
**62**	-1504.627238	-1503.357094	0.491311	-1504.102229	0.103137	0.094339	-1504.205366	-1504.203194
**58**	-731.523305	-731.507029	0.259267	-731.247753	0.062263	0.05868	-731.310017	-731.309116
**63**	-1044.678341	-1043.434204	0.359276	-1044.293699	0.08469	0.078078	-1044.378389	-1044.376974
**60**	-271.591541	-271.590818	0.128422	-271.455978	0.037729	0.037041	-271.493706	-271.493523

Table S10. Computed energies of lowest energy conformers of compounds Pd–P(*o*-tol)$_3$ complex **61**, ABO **2**, P(*o*-tol)$_3$ **56**, Pd–ABO complex **53**, Pd–dba complex **62**, dba **58**, Pd–PhMe complex **63**, and PhMe **60** at 25 °C at the M06-L/6-311++G(d,p)/SDD(Pd, I)//B3LYP-D3(BJ)/6-31G*/LANL2DZ(Pd, I) level of theory. All units are in Hartrees. E = electronic energy; SPC = single-point correction; ZPE = zero-point energy; H = enthalpy; T = temperature; S = entropy; G(T) = Gibbs free energy; qh-S = quasi-harmonic corrected entropy; qh-G(T) = quasi-harmonic corrected Gibbs free energy.

H. Structural Analysis of Pd–Ethylene Complex 64

The geometry of complex **64** was optimized at the B3LYP-D3(BJ)/6-31G(d)/LANL2DZ(Pd) level of theory, and its geometric properties were analyzed. The significantly longer C–Pd bonds, and short C=C bond, relative to **24**, are indicative of the decreased π-backbonding in this complex, presumably due to the unstrained nature of the alkene.

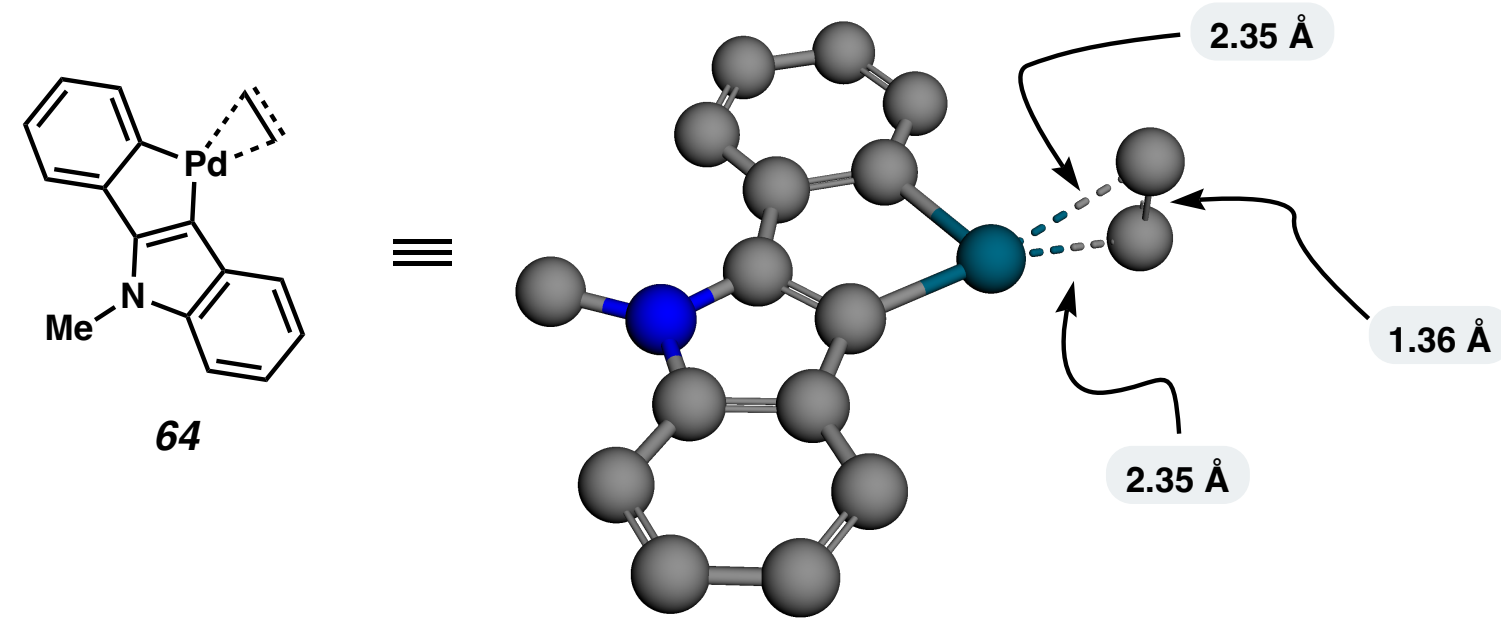

Figure S74. Structural parameters of Pd–ethylene complex **64**.

I. EDA-NOCV Analysis of Pd Complexes 24 and 64

EDA-NOCV analysis[39,40] was conducted at the BP86[22]-D3(BJ)/def2-TZVPP[41] level of theory as implemented in Orca 6.1.1[42,43,44,45,46] on compounds **24** and **64**, using the geometries previously optimized. This functional and a similar basis set have been utilized previously for analysis of transition metal-strained intermediate bonding modes.[47] The def2-TZVPP basis set was used in place of TZ2P, as TZ2P is not available outside of the ADF suite. Results of the EDA analysis are depicted in Table S11. The more stabilizing bonding energy in **24** compared to **64** (–50.07 vs –29.62 kcal/mol, respectively), as well as the higher orbital energy contribution (–82.17 vs –44.96 kcal/mol, respectively) are indicative of the much stronger Pd–ABO bonding.

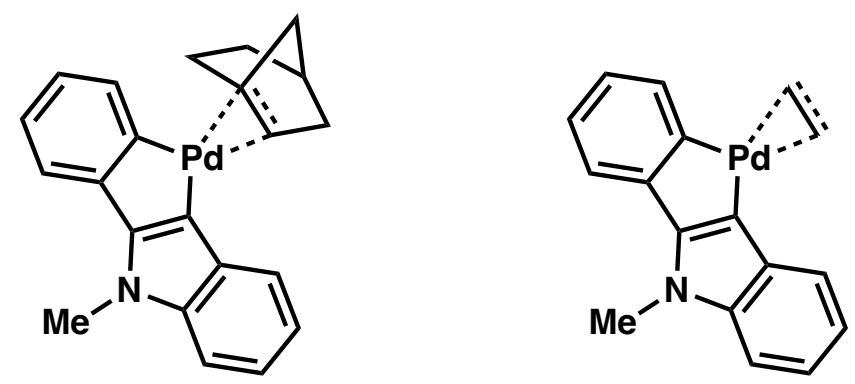

24 **64**

Structure	E_{bond}	E_{orb}	E_{elec}	E_{Pauli}	E_{XC}	E_{disp}
**24**	-50.07	-82.17	-73.07	182.91	-68.52	-9.21
**64**	-29.62	-44.96	-44.97	109.98	-43.82	-5.86

Table S11. Energy decomposition analysis of complexes **24** and **64** at the BP86-D3(BJ)/def2-TZVPP level of theory. All units are in kcal/mol. E_{bond} = Bond Energy; E_{orb} = Orbital Energy; E_{elec} = Electrostatic Energy; E_{Pauli} = Pauli Energy; E_{XC} = Exchange-Correlation Contribution; E_{disp} = Dispersion Contribution.

Further analysis of the EDA-NOCV allows the orbital energy (E_{orb}) to be decomposed into pairs of orbital contributions, representing the flow of charge between fragments. These are visualized as the deformation densities ($\Delta\rho$), depicted below. These can be mapped onto specific orbital interactions between FMOs. In the case of compound **24**, the most contributing interaction is the π backbonding from the HOMO of the Pd fragment to the LUMO of the ABO fragment (–37.6 kcal/mol), although π donation from the HOMO of the ABO fragment to the LUMO of the Pd fragment remains significant (–31.3).

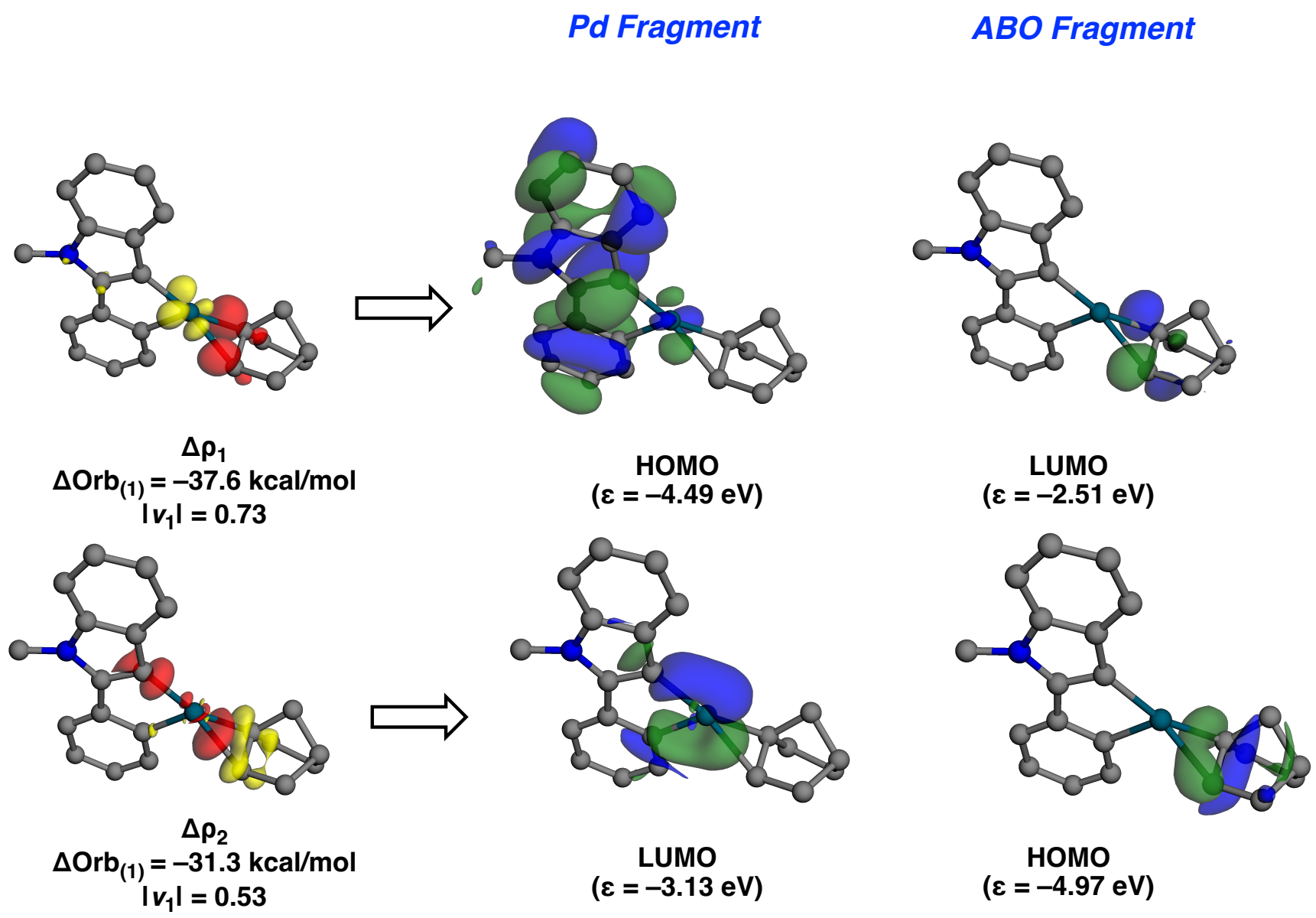

Figure S75. Plots of the two most major EDA-NOCV deformation densities in **24** and the corresponding FMOs of each fragment. Charge flow is from yellow to red, and the energetic contribution (ΔOrb) and eigenvalues for each are included.

When the same analysis is applied to complex **64**, the π backbonding from the HOMO of the Pd fragment to the LUMO of the ethylene fragment is less significant (–17.7 kcal/mol), representing a 52% decrease in backbonding. Additionally, π donation from the HOMO of the ethylene fragment to the LUMO of the Pd fragment is also decreased (–21.2), albeit by only 33% compared to complex **24**. Along with the ligand exchange computations and geometric analysis, these results support that the ABO is more tightly bound to Pd than a typical alkene, and heightened backbonding plays a major role in this interaction, likely due to the strain and geometric distortion of ABO **2**.

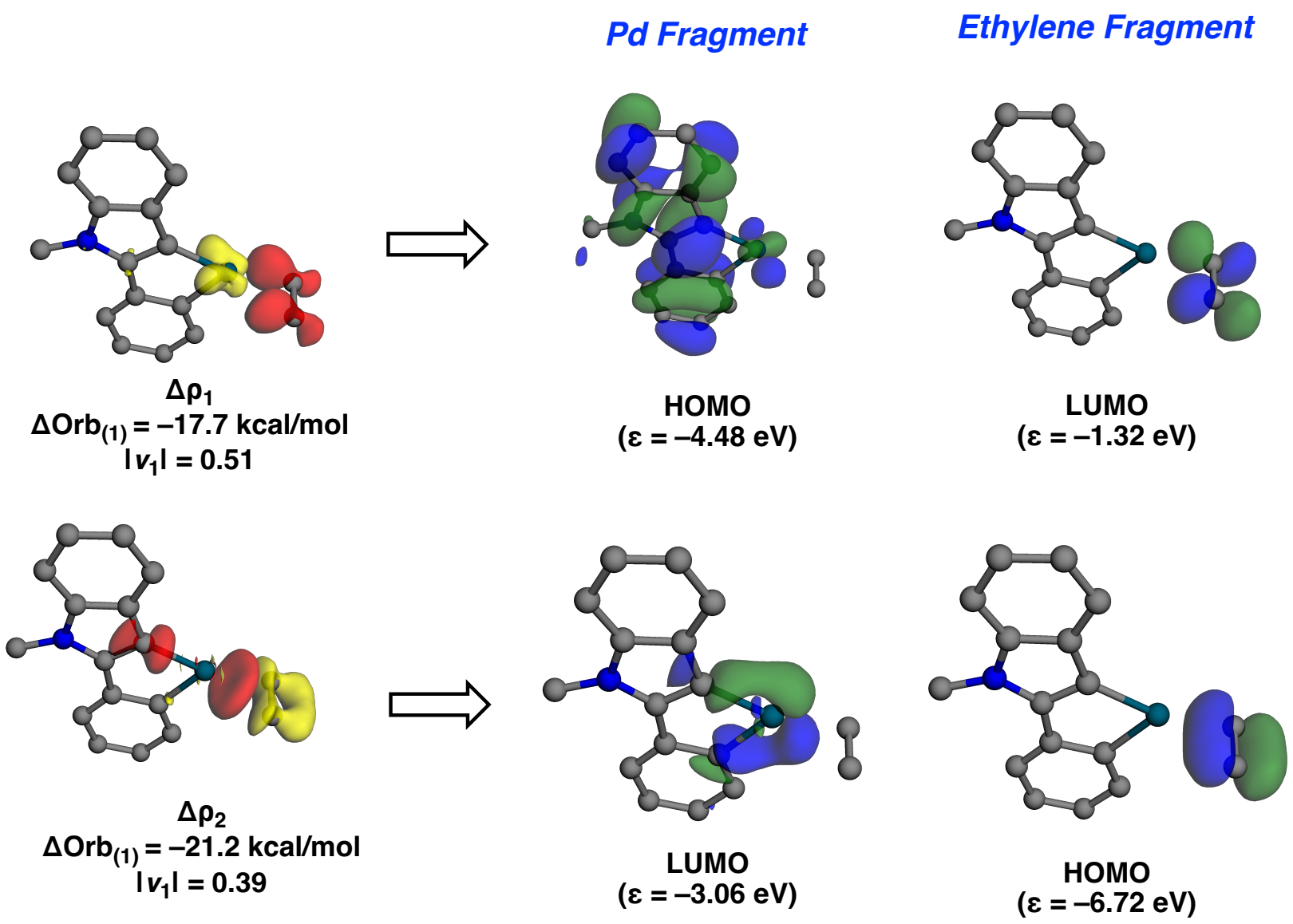

Figure S76. Plots of the two most major EDA-NOCV deformation densities in **64** and the corresponding FMOs of each fragment. Charge flow is from yellow to red, and the energetic contribution (ΔOrb) and eigenvalues for each are included.

J. Bond Order of ABO Fragment and Pd Complex

The Mayer bond orders[48] (MBO) of ABO **2**, the isolated ABO fragment of **24**, as well as in the complex **24** were calculated at the B3LYP-D3(BJ)/6-31G(d)/LANL2DZ(Pd) level of theory by using the *pop=(nboread,always)* keyword and adding *$nbo bndidx mulorb $end* at the end of Gaussian input file. The MBO of the C=C bond of **2** was found to be 1.83, the C=C bond of isolated ABO fragment of **24** was found to be 1.81, while the C=C bond in **24** was 1.28, while the C_1–Pd and C_2–Pd bonds were 0.51. The calculated MBOs, EDA-NOCV, and DFT analysis all support the proposed strong ABO–Pd interactions as well as indicating that they are not captured by looking at the ABO fragment in isolation, indicating that consideration of only the ABO fragment provides an incomplete picture. This suggests that electronic effects between the Pd and ABO fragment are responsible for this change in MBO, rather than simply geometric distortion.

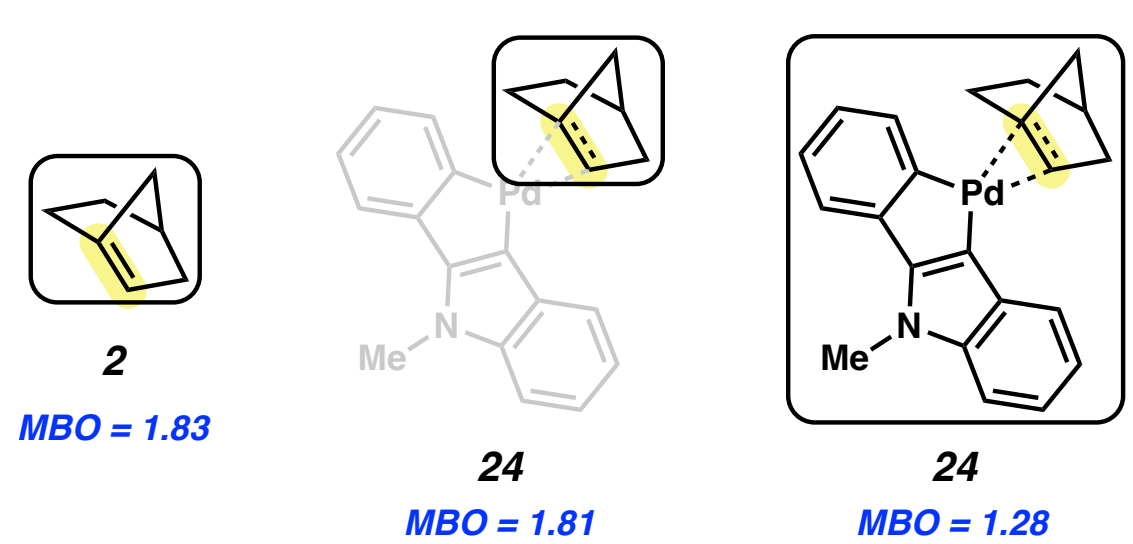

Figure S77. Mayer bond orders of highlighted C=C bonds.

K. Cartesian Coordinates for Optimized Structures

ABO–Pd Complex **24**

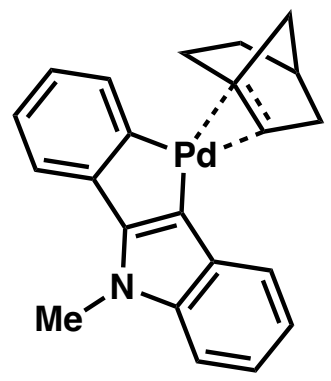

24

H	5.74730	-0.75795	-0.08383
C	4.80583	-1.29975	-0.08046
C	2.36387	-2.75637	-0.08158
C	4.79616	-2.69132	-0.11859
C	3.57545	-0.63817	-0.04274
C	2.33318	-1.35228	-0.04236
C	3.58658	-3.41390	-0.11966
H	1.43296	-3.31851	-0.08332
H	5.74030	-3.22765	-0.14962
H	3.61560	-4.49952	-0.15109
C	1.28721	-0.37149	0.00306
C	1.91182	0.86536	0.02219
N	3.29182	0.72380	0.00022
C	1.06058	2.04262	0.03482
C	-0.94540	4.01014	-0.01608
C	-0.31355	1.68594	0.04609
C	1.39792	3.39873	0.00927
C	0.39404	4.37650	-0.01119
C	-1.30377	2.64735	0.00906
H	-1.72379	4.76784	-0.04067
H	2.43742	3.70667	-0.00386
H	0.67177	5.42660	-0.03043
H	-2.35263	2.37324	-0.00525
C	4.27620	1.78489	0.02661
H	4.12812	2.43118	0.89823
H	4.22750	2.39691	-0.88162
H	5.27301	1.34763	0.09340
C	-2.80031	-0.48691	-0.47537
C	-5.07901	-0.84608	-0.53721
C	-3.60743	-1.42054	1.53726
C	-4.26980	-1.96546	0.22582
C	-2.82484	-0.25375	0.91260
C	-3.98879	0.11170	-1.20373
H	-5.70827	-1.30885	-1.30414
H	-5.73300	-0.28491	0.13863
H	-4.31692	-1.03174	2.27647
H	-3.01900	-2.20600	2.02948
H	-4.83744	-2.89411	0.35088

H	-3.01525	0.74615	1.29311
H	-4.16834	1.16168	-0.95979
H	-3.95289	0.00165	-2.29169
C	-3.00679	-2.00832	-0.64768
H	-2.20876	-2.63262	-0.22255
H	-3.16662	-2.30010	-1.68946
Pd	-0.73932	-0.29299	0.08945

ABO–Pd Complex ***iso*-24**

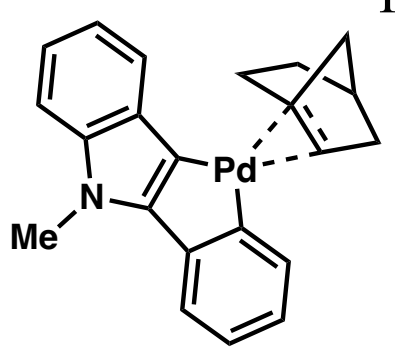

iso-24

H	-4.736135	-2.648228	0.256628
C	-3.664822	-2.719338	0.134210
C	-0.868230	-2.973880	-0.204158
C	-3.063622	-3.953575	0.012014
C	-2.859434	-1.586686	0.089136
C	-1.447405	-1.708164	-0.071716
C	-1.681417	-4.080815	-0.159822
H	0.200198	-3.058747	-0.352317
H	-3.673828	-4.844362	0.043395
H	-1.256528	-5.068866	-0.261887
C	-0.940605	-0.384316	-0.073806
C	-2.021808	0.467744	0.063841
N	-3.184234	-0.257363	0.169747
C	-1.713749	1.885802	0.033794
C	-0.643220	4.441463	-0.117757
C	-0.316062	2.050523	-0.140694
C	-2.546511	2.989525	0.126654
C	-2.001065	4.265590	0.051042
C	0.200036	3.332714	-0.212314
H	-0.228563	5.438244	-0.176764
H	-3.612541	2.878578	0.256447
H	-2.649799	5.127009	0.124759
H	1.268571	3.504484	-0.348640
C	-4.504074	0.282622	0.332077
H	-4.559614	0.893419	1.236753
H	-5.219931	-0.531744	0.419262
H	-4.778561	0.897092	-0.529551
C	2.548920	-0.849128	-0.505263
C	4.691448	-1.371655	0.243080
C	3.097873	0.037457	1.646946
C	4.240427	0.072046	0.605298
C	2.100091	-0.795908	0.863861

C	3.575163	-1.950335	-0.691611
H	5.649462	-1.338928	-0.275473
H	4.811314	-1.980322	1.138972
H	3.367418	-0.452498	2.583829
H	2.758130	1.059933	1.887583
H	5.073315	0.725316	0.873323
H	1.667058	-1.673949	1.344488
H	3.220582	-2.921633	-0.350790
H	3.922471	-2.042810	-1.720869
C	3.428825	0.422004	-0.634215
H	2.860360	1.378376	-0.528046
H	4.014095	0.447621	-1.555041
Pd	0.881035	0.408074	-0.295606

Palladacycle **10a**

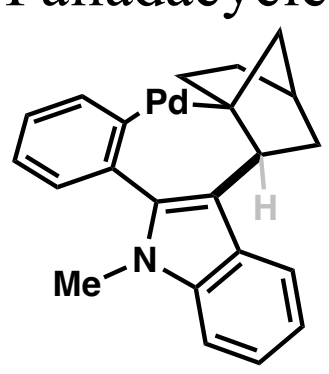

10a

H	0.619842	4.439495	0.746350
C	0.018634	3.525577	0.752381
C	-0.051726	1.107081	1.141730
C	-2.135892	2.418080	0.577565
C	-0.940232	1.641618	-0.011545
C	-1.477911	3.758296	1.050950
C	0.532761	2.437178	1.727082
H	-0.701637	0.625687	1.878657
H	-2.904499	2.604865	-0.178122
H	-2.606461	1.856687	1.390261
H	-1.863421	4.606858	0.474805
H	-1.658554	3.967972	2.110391
H	1.620164	2.411067	1.782855
H	0.168537	2.615670	2.744104
C	-0.050606	2.760804	-0.597295
H	-0.529866	3.350464	-1.388179
H	0.921590	2.401316	-0.945032
Pd	-1.476372	0.321216	-1.427131
C	-0.798247	-1.771551	0.563898
C	-3.428716	-2.729335	0.811467
C	-1.084632	-2.895772	1.357745
C	-1.854096	-1.130528	-0.124777
C	-3.163703	-1.604417	0.023788
C	-2.387166	-3.377436	1.476214
H	-0.276364	-3.377416	1.902072
H	-3.983769	-1.103052	-0.481708

H	-2.589635	-4.244532	2.098558
H	-4.448881	-3.091543	0.909350
C	0.565667	-1.233825	0.466291
N	1.663160	-1.993732	0.067486
C	2.761232	-1.161759	-0.016042
C	2.352777	0.159317	0.348969
C	0.949333	0.078620	0.679862
C	3.321552	1.181625	0.272415
C	4.618110	0.876880	-0.124489
C	4.993274	-0.438510	-0.456630
C	4.065983	-1.473025	-0.408872
H	3.068376	2.211431	0.495215
H	5.355995	1.672048	-0.183919
H	6.015296	-0.646983	-0.759511
H	4.346321	-2.488764	-0.671408
C	1.655216	-3.370160	-0.382868
H	2.146423	-3.446932	-1.359097
H	2.177763	-4.026872	0.323720
H	0.624095	-3.709833	-0.480761

Palladacycle **10b**

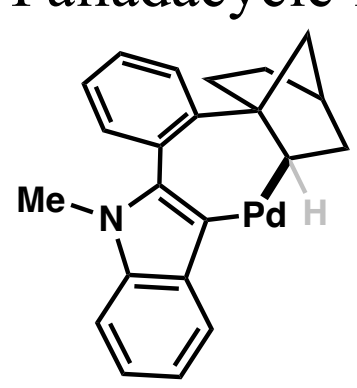

10b

H	0.888785	-2.717315	2.871291
C	1.372169	-2.165404	2.066732
C	1.487738	-1.555436	-0.251471
C	3.253998	-0.865939	1.319945
C	1.894262	-0.431064	0.717065
C	2.894213	-2.073701	2.205537
C	1.046440	-2.706370	0.668933
H	2.331748	-1.832849	-0.927196
H	3.669470	-0.056505	1.920864
H	3.978875	-1.131710	0.551891
H	3.176327	-1.896745	3.243470
H	3.386320	-2.988699	1.877096
H	1.574310	-3.637314	0.456036
H	-0.032170	-2.890934	0.583187
C	0.977829	-0.698514	1.927971
H	1.255138	-0.095514	2.790726
H	-0.077259	-0.580977	1.706539
Pd	0.216265	-1.080727	-1.699163
C	0.705662	1.700129	-0.087520
C	3.159986	2.870813	-0.683772

C	0.786293	3.019099	-0.534514
C	1.889547	0.956903	0.110478
C	3.096433	1.567749	-0.220665
C	2.000515	3.610656	-0.822070
H	-0.128188	3.572765	-0.690658
H	4.019738	1.021119	-0.114276
H	2.038719	4.631956	-1.171070
H	4.117135	3.306049	-0.930579
C	-0.621324	1.097758	0.055697
N	-1.690542	1.656845	0.729976
C	-2.770080	0.830638	0.573560
C	-2.380189	-0.271397	-0.233799
C	-3.301014	-1.273757	-0.543708
C	-4.580796	-1.163680	-0.052559
C	-4.957756	-0.072055	0.737449
C	-4.068631	0.931438	1.059886
H	-2.987049	-2.111773	-1.157591
H	-5.314598	-1.924629	-0.275537
H	-5.973370	-0.015867	1.101559
H	-4.377528	1.769602	1.667289
C	-1.660075	2.817959	1.573409
H	-0.625663	3.107004	1.750964
H	-2.134586	2.594056	2.531370
H	-2.190497	3.657014	1.112723
C	-1.010232	-0.069694	-0.560988

Palladacycle ***iso*-10a**

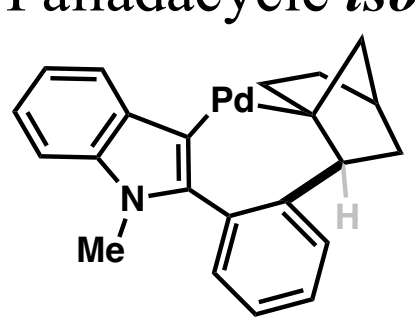

iso-10a

H	-4.360544	-1.950644	1.417630
C	-3.309127	-1.717528	1.249526
C	-1.571085	-0.024828	1.137892
C	-1.037892	-2.475083	0.955899
C	-1.428350	-1.218697	0.168494
C	-2.353224	-2.835632	1.687223
C	-2.854585	-0.409345	1.914142
H	-0.709914	-0.003264	1.811127
H	-0.729852	-3.276627	0.273530
H	-0.214887	-2.278352	1.641817
H	-2.728732	-3.808338	1.368516
H	-2.229922	-2.860645	2.768860
H	-3.626441	0.353415	1.844704
H	-2.640848	-0.559125	2.971888

C	-2.887571	-1.503612	-0.209718
H	-3.013884	-2.406134	-0.816040
H	-3.388419	-0.652822	-0.679176
Pd	-0.429290	-0.906628	-1.496454
C	-0.344298	1.881576	0.035282
C	-2.691767	3.270016	-0.476334
C	-0.308576	3.157784	-0.520484
C	-1.580657	1.300807	0.397730
C	-2.737602	2.010977	0.101904
C	-1.475083	3.858045	-0.766138
H	0.645049	3.587710	-0.791335
H	-3.708545	1.596009	0.326423
H	-1.434356	4.845518	-1.201898
H	-3.612392	3.791907	-0.693606
C	0.887834	1.093510	0.136819
N	2.078986	1.499671	0.703141
C	2.997843	0.502629	0.517399
C	2.376521	-0.556316	-0.198390
C	1.034249	-0.150084	-0.436225
C	3.102385	-1.704581	-0.518778
C	4.418869	-1.779486	-0.127924
C	5.023401	-0.730737	0.573910
C	4.330113	0.414479	0.904697
H	2.613590	-2.505925	-1.061533
H	5.004971	-2.656190	-0.362480
H	6.060799	-0.822612	0.861215
H	4.812556	1.216945	1.443432
C	2.304240	2.705491	1.448285
H	2.940258	3.400793	0.891874
H	1.348358	3.185359	1.651711
H	2.793774	2.473913	2.396947

Palladacycle ***iso*-10b**

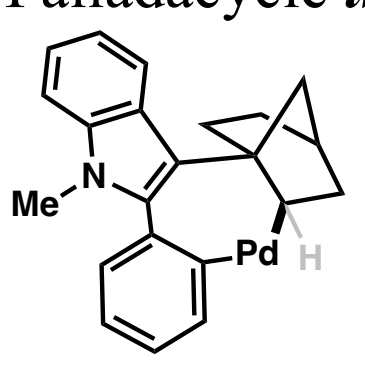

iso-10b

H	-0.378729	4.528764	-0.335209
C	-0.308190	3.567374	0.175127
C	-1.260188	1.412434	0.617865
C	0.763923	2.171402	1.829949
C	0.267331	1.418269	0.576444
C	0.422991	3.641763	1.522004
C	-1.660043	2.881034	0.404406
H	-1.709125	0.927808	1.488799

H	0.251281	1.805586	2.718595
H	1.832075	2.024947	1.977191
H	-0.199570	4.094201	2.292825
H	1.327993	4.242800	1.426769
H	-2.308991	2.997209	-0.486989
H	-2.199648	3.297137	1.256726
C	0.475979	2.474518	-0.542404
H	1.517011	2.730220	-0.721907
H	0.001952	2.151510	-1.502431
Pd	-1.598801	0.632388	-1.178739
C	-0.994709	-1.656014	0.451510
C	-3.581750	-2.639265	0.755247
C	-1.243088	-2.749458	1.279400
C	-2.065152	-1.032598	-0.209119
C	-3.353770	-1.525077	-0.042011
C	-2.530238	-3.236129	1.427922
H	-0.431577	-3.203871	1.829899
H	-4.193471	-1.043248	-0.536079
H	-2.712409	-4.081234	2.076207
H	-4.584465	-3.026758	0.867064
C	0.384111	-1.177369	0.238179
N	1.383571	-2.089592	-0.031936
C	2.574269	-1.432419	-0.128901
C	2.339746	-0.053766	0.093484
C	0.932461	0.095803	0.312536
C	3.428650	0.822088	0.061028
C	4.684380	0.320709	-0.194442
C	4.893494	-1.041461	-0.421247
C	3.844588	-1.933364	-0.390937
H	3.303139	1.879503	0.235360
H	5.531211	0.991353	-0.220462
H	5.893840	-1.397301	-0.618832
H	4.008540	-2.987652	-0.556516
C	1.216312	-3.475607	-0.372733
H	0.167099	-3.681784	-0.575256
H	1.799674	-3.702663	-1.267482
H	1.557388	-4.126430	0.437452

Annulation product **16**

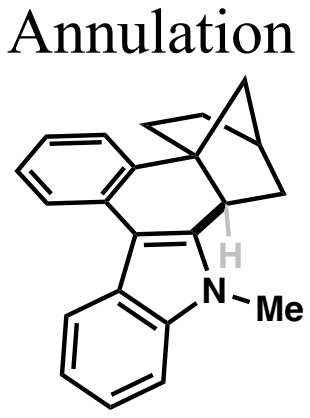

16

H	2.796725	-2.849373	1.889665
C	2.585288	-2.171372	1.059023

C	1.012573	-1.281700	-0.574140
C	3.411601	-0.649440	-0.638935
C	2.049366	-0.241981	-0.027169
C	3.764855	-1.977019	0.090104
C	1.351021	-2.574279	0.244150
H	1.186216	-1.450434	-1.646223
H	3.347469	-0.755786	-1.726429
H	4.179462	0.099700	-0.435435
H	4.707139	-1.881969	0.639099
H	3.872907	-2.822554	-0.597295
H	1.540360	-3.439485	-0.399904
H	0.525634	-2.832118	0.915742
H	-4.268404	-2.184228	-0.232189
C	-3.949369	-1.161201	-0.056267
C	-3.108257	1.494566	0.455080
C	-4.863965	-0.178243	0.285368
C	-2.606499	-0.792169	-0.144089
C	-2.157943	0.529877	0.088450
C	-4.443730	1.135747	0.545350
H	-2.811739	2.509949	0.693279
H	-5.916701	-0.431494	0.365825
H	-5.178199	1.882827	0.830682
C	-0.724831	0.507079	-0.091364
C	-0.382322	-0.790662	-0.373381
N	-1.510790	-1.579232	-0.436724
H	3.656210	1.982160	-0.187163
C	2.600204	2.230964	-0.181470
C	-0.072835	2.922242	-0.171036
C	1.646393	1.215022	-0.140852
C	2.238504	3.574594	-0.213977
C	0.893639	3.919507	-0.216371
C	0.279358	1.570498	-0.112249
H	-1.119490	3.199659	-0.212692
H	3.006231	4.341772	-0.246073
H	0.591928	4.961864	-0.261126
C	-1.597141	-2.965443	-0.833844
H	-1.779645	-3.621918	0.024669
H	-2.411588	-3.091242	-1.553825
H	-0.668350	-3.269281	-1.316929
C	2.233751	-0.728007	1.432487
H	1.318223	-0.634258	2.025929
H	3.043172	-0.190660	1.938243

Annulation product **iso-16**

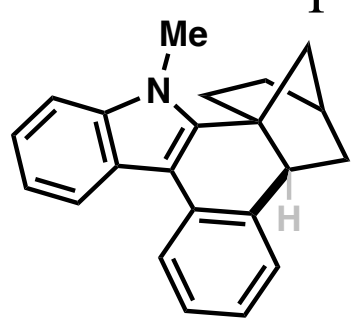

iso-16

H	4.063896	-1.947059	1.350292
C	3.266865	-1.672134	0.655289
C	2.070326	0.043032	-0.590425
C	1.585495	-2.354999	-0.956878
C	1.184926	-1.141676	-0.073325
C	3.007721	-2.717671	-0.443608
C	3.475109	-0.302962	-0.006519
H	2.097710	-0.017417	-1.686475
H	1.566652	-2.088076	-2.017632
H	0.906634	-3.199698	-0.833282
H	3.764623	-2.676273	-1.233033
H	3.025589	-3.730356	-0.027052
H	3.766058	0.432916	0.749858
H	4.257124	-0.320991	-0.772091
H	-4.060010	-2.400113	0.130000
C	-3.817265	-1.344004	0.061049
C	-3.197736	1.406097	-0.188685
C	-4.820360	-0.393062	-0.025691
C	-2.494680	-0.897368	0.030805
C	-2.157012	0.471238	-0.076679
C	-4.511616	0.970326	-0.157752
H	-2.988306	2.460824	-0.326991
H	-5.859111	-0.708914	-0.007227
H	-5.316848	1.693239	-0.246242
C	-0.715872	0.530598	-0.085810
C	-0.258279	-0.764650	-0.031346
N	-1.327686	-1.634821	0.077726
H	3.518362	2.334725	-0.404652
C	2.461860	2.509228	-0.217926
C	-0.222579	2.969342	0.251437
C	1.575788	1.435419	-0.252004
C	2.023414	3.803986	0.044136
C	0.674652	4.029005	0.292591
C	0.202436	1.669511	-0.046633
H	-1.264817	3.151074	0.485397
H	2.733311	4.625445	0.063877
H	0.317555	5.028113	0.524312
C	-1.305356	-3.056647	0.336857
H	-0.397791	-3.328285	0.876090
H	-2.155631	-3.313944	0.973335

H	-1.366435	-3.645830	-0.585254
C	1.871530	-1.495673	1.267950
H	1.809412	-0.677698	1.992944
H	1.479620	-2.409545	1.728751

P(*o*-tol)$_3$ Pd complex **55**

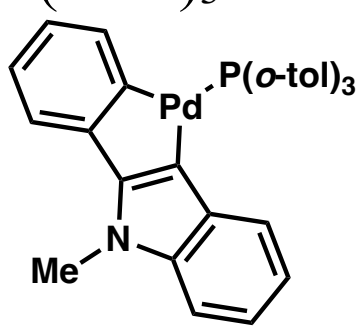

55

C	-3.63092	-1.48149	0.17189
C	-3.58514	-4.27906	0.10816
C	-2.39160	-2.16799	0.06715
C	-4.82554	-2.20195	0.25115
C	-4.79556	-3.60268	0.22012
C	-2.38033	-3.55534	0.03090
H	-3.56763	-5.36588	0.07926
H	-5.78122	-1.69556	0.33683
H	-5.72690	-4.15937	0.28160
H	-1.44122	-4.09951	-0.06129
Pd	-0.69793	-1.03442	0.05493
P	1.53735	-0.05632	0.12604
C	1.97908	1.41104	-0.86895
C	2.44538	3.78147	-2.28283
C	1.47852	1.54249	-2.18619
C	2.69796	2.46264	-0.28385
C	2.92907	3.64587	-0.98271
C	1.73501	2.73515	-2.87097
H	2.61513	4.69918	-2.83859
H	3.07596	2.35426	0.72716
H	3.48334	4.45290	-0.51264
H	1.35378	2.84664	-3.88245
C	2.33625	-1.54992	-0.60081
C	3.10840	-4.02702	-1.67527
C	3.46088	-1.61381	-1.45074
C	1.64457	-2.73576	-0.28254
C	2.01608	-3.96808	-0.81021
C	3.81760	-2.86568	-1.97566
H	3.41636	-4.97551	-2.10544
H	0.82114	-2.70750	0.44166
H	1.46282	-4.86434	-0.54747
H	4.68209	-2.92455	-2.63204
C	2.39110	0.16248	1.73414
C	3.69795	0.42395	4.19722
C	3.63145	-0.45437	1.95742

C	1.78426	0.91954	2.76357
C	2.46287	1.03384	3.98359
C	4.28672	-0.32572	3.17988
H	4.19457	0.53009	5.15764
H	4.08184	-1.05128	1.17180
H	2.00369	1.61165	4.78134
H	5.24617	-0.81056	3.33481
C	0.67916	0.45450	-2.85561
H	-0.21946	0.20841	-2.27434
H	1.25344	-0.47389	-2.95418
H	0.36397	0.76601	-3.85531
C	4.30311	-0.41201	-1.80742
H	4.50199	0.21926	-0.93761
H	5.26250	-0.73528	-2.22235
H	3.81304	0.22545	-2.55068
C	0.45677	1.60779	2.58138
H	0.10492	2.02762	3.52800
H	-0.30988	0.92359	2.20158
H	0.52166	2.43000	1.86056
H	-0.13017	2.59978	-0.32259
C	-1.17945	2.84577	-0.23873
C	-3.93168	3.52552	-0.08196
C	-2.13108	1.82516	-0.06375
C	-1.59403	4.16779	-0.32558
C	-2.95828	4.50735	-0.24168
C	-3.51936	2.19208	-0.00318
H	-0.85360	4.95054	-0.46587
H	-3.25961	5.54887	-0.30978
H	-4.98171	3.79882	-0.02969
C	-2.09088	0.39995	0.05706
C	-3.39981	-0.04671	0.15021
N	-4.27348	1.03419	0.12904
C	-5.71722	0.97134	0.20311
H	-6.13248	0.41754	-0.64649
H	-6.12198	1.98383	0.18446
H	-6.04156	0.48849	1.13144

ABO **2**

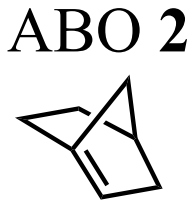

2

H	2.48430	-0.18050	0.03948
C	1.54956	0.01909	-0.50447
C	-0.27330	0.85044	0.67252
C	-0.94163	-0.75765	-0.81936
C	-1.55054	0.53629	-0.08615

C	0.45225	-1.04664	-0.12373
C	0.74181	1.26924	-0.12737
H	1.79730	0.06175	-1.57215
H	-1.59710	-1.62597	-0.68948
H	-0.83502	-0.57288	-1.89392
H	0.76520	-2.09418	-0.22314
H	0.60335	2.06264	-0.86006
C	0.13920	-0.50058	1.27808
H	-0.67678	-1.01621	1.79527
H	1.00979	-0.44331	1.93802
H	-2.41468	0.28444	0.53803
H	-1.84046	1.30308	-0.80918

P(*o*-tol)$_3$ **56**

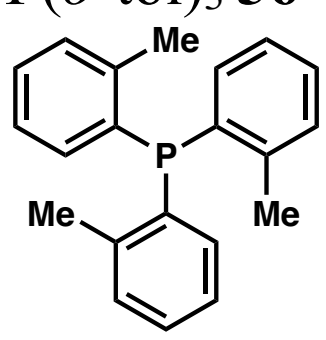

54

P	0.03938	-0.08643	-1.09697
C	-1.28669	-1.08074	-0.30032
C	-3.34810	-2.67330	0.74626
C	-1.01450	-2.07687	0.64688
C	-2.61250	-0.90817	-0.76395
C	-3.62381	-1.70458	-0.21898
C	-2.03580	-2.86704	1.17294
H	-4.15326	-3.27997	1.15125
H	0.00699	-2.23551	0.97589
H	-4.64528	-1.56536	-0.56487
H	-1.80461	-3.62795	1.91327
C	1.60710	-0.81487	-0.43942
C	4.04590	-2.07441	0.17942
C	2.17412	-0.64998	0.84711
C	2.28261	-1.60681	-1.38374
C	3.48601	-2.24204	-1.08440
C	3.39125	-1.28604	1.12434
H	4.98743	-2.55377	0.43324
H	1.84896	-1.71877	-2.37390
H	3.98153	-2.85306	-1.83342
H	3.83018	-1.16116	2.11128
C	0.00338	1.63069	-0.39259
C	0.31551	4.34973	0.28798
C	-0.82633	2.20280	0.60116
C	0.96071	2.44693	-1.02571
C	1.13625	3.78365	-0.68599
C	-0.64906	3.56164	0.90801

H	0.42373	5.39485	0.56418
H	1.58662	2.01184	-1.80152
H	1.89629	4.37748	-1.18570
H	-1.28391	4.00315	1.67239
C	-1.88225	1.45491	1.38193
H	-2.21444	2.06033	2.23059
H	-2.76442	1.22764	0.77570
H	-1.52256	0.49968	1.76742
C	1.53072	0.17304	1.93406
H	0.47301	-0.07432	2.05263
H	1.58621	1.24402	1.71620
H	2.02740	-0.00488	2.89270
C	-2.94761	0.11454	-1.82305
H	-2.71738	1.13651	-1.49867
H	-4.01023	0.07666	-2.08056
H	-2.36441	-0.05945	-2.73519

dba-Pd complex **57**

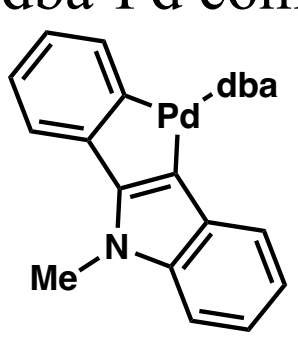

57

H	2.92542	2.52387	-0.60312
C	2.06658	3.17231	-0.45321
C	-0.17354	4.87559	-0.03214
C	2.11765	4.51706	-0.79566
C	0.88980	2.64646	0.10690
C	-0.23016	3.52176	0.31082
C	1.00744	5.36023	-0.58769
H	-1.01677	5.54059	0.13168
H	3.02555	4.93079	-1.22617
H	1.07376	6.40975	-0.86000
N	-1.26811	2.78065	0.86412
C	-0.81933	1.47452	1.01835
C	0.48347	1.35204	0.56336
C	-1.44201	0.27969	1.55538
C	-2.14190	-2.23175	2.58434
C	-2.68875	0.09127	2.15945
C	-0.56121	-0.82522	1.43963
C	-0.88490	-2.05693	1.97694
C	-3.03726	-1.16872	2.65927
H	-2.41142	-3.20606	2.98276
H	-3.38861	0.91460	2.25232
H	-0.20967	-2.90316	1.89568
H	-4.01103	-1.30996	3.11987

C	-2.58630	3.28028	1.19231
H	-3.35524	2.63661	0.75357
H	-2.74088	3.33091	2.27691
H	-2.70310	4.28334	0.77873
Pd	1.14269	-0.53115	0.39866
H	1.08344	-1.84648	-1.93019
C	1.13385	-2.48445	-1.05303
C	-0.03134	-3.39844	-0.81821
O	0.12925	-4.47700	-0.25051
C	-1.37346	-2.93332	-1.20412
H	-2.15186	-3.68177	-1.08671
C	-1.65329	-1.63340	-1.43790
H	-0.82446	-0.93199	-1.46444
C	2.27547	-2.56175	-0.30058
H	2.31643	-3.32079	0.47698
C	-2.95651	-0.98828	-1.53346
C	-5.40032	0.39606	-1.60215
C	-4.17575	-1.68685	-1.48081
C	-2.98619	0.41601	-1.62224
C	-4.19698	1.10250	-1.65640
C	-5.38431	-1.00004	-1.51561
H	-6.34716	0.92792	-1.62778
H	-4.17374	-2.76956	-1.40376
H	-2.04785	0.96350	-1.63847
H	-4.20150	2.18651	-1.72805
H	-6.31905	-1.55157	-1.47182
C	3.43367	-1.66797	-0.43199
C	5.59528	0.14225	-0.48348
C	3.46142	-0.59708	-1.35662
C	4.51415	-1.80320	0.46401
C	5.58197	-0.91448	0.43339
C	4.53384	0.29638	-1.37433
H	6.42707	0.83967	-0.49990
H	2.66432	-0.47520	-2.08305
H	4.49765	-2.61351	1.18748
H	6.40501	-1.03900	1.13076
H	4.53642	1.10988	-2.09346

dba **58**

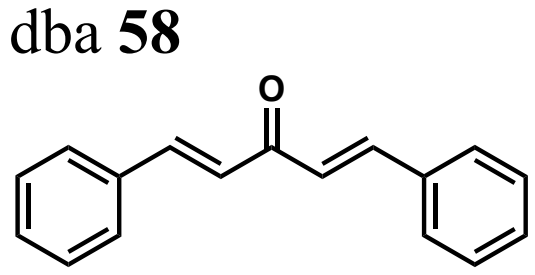

58

H	5.41361	2.85997	-0.00026
C	5.27250	1.78285	-0.00011
C	4.90662	-0.97650	0.00028

C	6.38312	0.93266	0.00017
C	3.98645	1.25641	-0.00019
C	3.77895	-0.13627	0.00000
C	6.19569	-0.44967	0.00036
H	4.75964	-2.05335	0.00043
H	7.38681	1.34791	0.00024
H	3.13394	1.92839	-0.00042
H	7.05270	-1.11699	0.00058
C	2.45301	-0.74560	-0.00008
H	2.42358	-1.83447	0.00002
C	1.25662	-0.12671	-0.00024
H	1.17169	0.95717	-0.00032
C	0.00000	-0.91192	-0.00026
C	-1.25662	-0.12671	-0.00023
H	-1.17169	0.95717	-0.00034
C	-2.45301	-0.74560	-0.00006
H	-2.42358	-1.83447	0.00003
C	-3.77895	-0.13627	0.00003
C	-6.38312	0.93266	0.00017
C	-4.90662	-0.97650	0.00012
C	-3.98645	1.25641	0.00002
C	-5.27250	1.78285	0.00009
C	-6.19569	-0.44967	0.00019
H	-7.38681	1.34791	0.00023
H	-4.75964	-2.05335	0.00013
H	-3.13394	1.92839	-0.00004
H	-5.41361	2.85997	0.00008
H	-7.05270	-1.11699	0.00026
O	0.00000	-2.14398	-0.00027

PhMe-Pd complex **59**

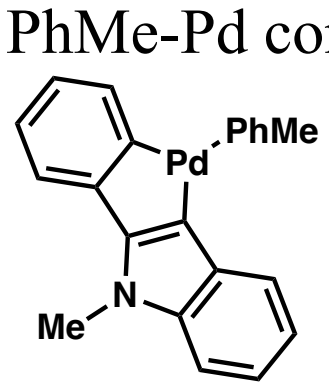

59

C	0.47613	2.03894	-0.09554
C	-0.48015	4.63401	0.30698
C	1.36038	3.10567	-0.09018
C	-0.91479	2.26093	0.09933
C	-1.38264	3.56222	0.29881
C	0.88007	4.41320	0.11499
H	-0.85010	5.64377	0.46301
H	2.42550	2.94349	-0.24251
H	-2.44039	3.75557	0.44578
H	1.57590	5.24834	0.12153

C	-1.62501	0.99716	0.04010
C	-0.86510	-0.13289	-0.19508
C	-1.75536	-1.24935	-0.22593
C	-3.07112	-0.72018	0.01260
N	-2.96729	0.65506	0.16988
C	-4.19474	-1.55024	0.05690
C	-4.01207	-2.91638	-0.13943
C	-2.73251	-3.45378	-0.38314
C	-1.61298	-2.63357	-0.42896
H	-5.18914	-1.15147	0.23641
H	-4.87383	-3.57694	-0.10839
H	-2.62516	-4.52349	-0.54100
H	-0.63088	-3.05120	-0.63049
C	-4.06210	1.56637	0.42213
H	-4.98957	0.99926	0.51104
H	-4.17446	2.28593	-0.39715
H	-3.90524	2.11743	1.35610
Pd	1.09326	0.14402	-0.34344
H	2.94076	-1.20157	-2.44533
C	2.98995	-1.22647	-1.36188
C	3.16170	-1.35703	1.43366
C	2.28114	-2.20879	-0.64991
C	3.75492	-0.27491	-0.66339
C	3.83439	-0.34970	0.73533
C	2.35465	-2.28332	0.75809
H	3.22954	-1.39991	2.51634
H	1.68397	-2.93466	-1.19291
H	4.29351	0.49582	-1.20471
H	4.42505	0.38072	1.27959
C	1.54796	-3.30940	1.51023
H	1.92383	-3.44236	2.52868
H	0.50011	-2.99302	1.57402
H	1.56532	-4.28090	1.00479

PhMe **60**

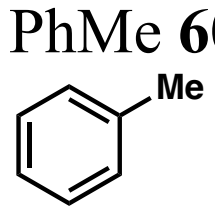

60

C	-1.20032	1.20502	0.00221
C	0.19457	1.20214	-0.00908
C	0.91288	0.00025	-0.01158
C	0.19486	-1.20198	-0.00909
C	-1.19989	-1.20525	0.00222
C	-1.90345	-0.00017	0.00855
H	-1.73795	2.14941	0.00159
H	0.73567	2.14555	-0.01783

H	0.73626	-2.14524	-0.01786
H	-1.73731	-2.14976	0.00161
H	-2.98981	-0.00037	0.01402
C	2.42259	0.00010	0.00946
H	2.80526	-0.01313	1.03877
H	2.83033	-0.87982	-0.49945
H	2.83015	0.89264	-0.47698

$P(o\text{-tol})_3$ PdI complex **61**

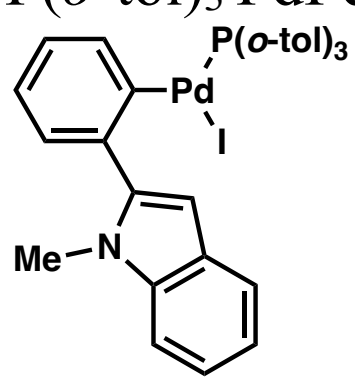

H	2.98674	-1.38810	1.95060
C	2.00868	-1.51885	2.39827
C	-0.49935	-1.88574	3.55161
C	1.89828	-1.78269	3.76555
C	0.85886	-1.41477	1.61291
C	-0.41528	-1.61665	2.17096
C	0.64100	-1.95690	4.34646
H	-1.48474	-2.03012	3.98461
H	2.79827	-1.85337	4.37024
H	0.54996	-2.16141	5.40922
C	-1.67021	-1.44097	1.42356
C	-2.69257	-0.55951	1.70370
C	-3.72039	-0.75972	0.72492
C	-3.25688	-1.79216	-0.14020
N	-2.00447	-2.18975	0.29311
H	-2.67582	0.17731	2.49459
C	-4.02155	-2.26878	-1.20931
C	-5.27269	-1.69284	-1.41076
C	-5.75128	-0.67207	-0.56495
C	-4.98618	-0.19967	0.49445
H	-3.65604	-3.06048	-1.85614
H	-5.89380	-2.04191	-2.23093
H	-6.73447	-0.24779	-0.74899
H	-5.35976	0.59255	1.13697
C	-1.35883	-3.41560	-0.14739
H	-1.08018	-3.35279	-1.20291
H	-0.44694	-3.57032	0.42516
H	-2.03170	-4.26902	-0.00196
Pd	1.10186	-0.85121	-0.27115
I	2.39086	-3.03155	-1.08249
P	0.26255	1.32524	-0.09166
C	1.40248	2.05912	-1.33832

C	3.12690	3.09697	-3.29039
C	0.90420	2.52675	-2.56402
C	2.79513	2.11216	-1.08009
C	3.62921	2.64029	-2.07308
C	1.75589	3.04122	-3.53713
H	3.80455	3.49432	-4.04040
H	-0.16413	2.50882	-2.74813
H	4.69780	2.68766	-1.88193
H	1.34861	3.39936	-4.47793
C	0.38938	2.37673	1.40737
C	0.59053	3.78663	3.82528
C	0.35158	1.70818	2.64042
C	0.51872	3.78865	1.37919
C	0.61977	4.46009	2.60484
C	0.44855	2.40193	3.84419
H	0.67271	4.34601	4.75292
H	0.25044	0.63424	2.65985
H	0.71887	5.54240	2.59446
H	0.41722	1.85484	4.78136
C	-1.42605	1.58214	-0.75062
C	-4.00440	2.05608	-1.73732
C	-1.90305	0.82241	-1.84593
C	-2.26209	2.53389	-0.14861
C	-3.54220	2.77628	-0.63870
C	-3.19046	1.09077	-2.32320
H	-5.00530	2.22307	-2.12291
H	-1.91166	3.08422	0.71658
H	-4.17346	3.51761	-0.15769
H	-3.56982	0.50243	-3.15262
C	3.40534	1.64921	0.21989
H	3.14178	0.60571	0.43084
H	4.49552	1.72087	0.17870
H	3.05350	2.24549	1.06858
C	0.54954	4.61488	0.11466
H	0.37091	5.66745	0.35349
H	1.51794	4.54510	-0.39121
H	-0.20991	4.29906	-0.60425
C	-1.09780	-0.26648	-2.50847
H	-1.66176	-0.71969	-3.32815
H	-0.85993	-1.06149	-1.79188
H	-0.15036	0.09961	-2.92039

ABO–PdI Complex **53**

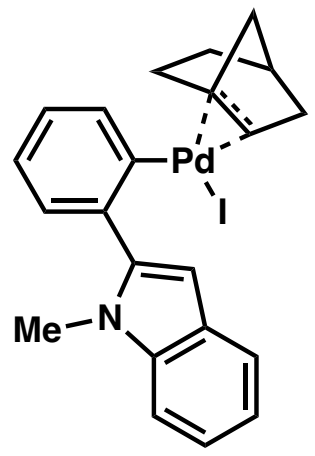

53

H	-2.93028	-0.17773	4.25645
C	-2.06856	0.06921	3.64273
C	0.14849	0.72503	2.04394
C	-0.94380	0.66517	4.21728
C	-2.10171	-0.20319	2.27091
C	-0.99337	0.12101	1.49856
C	0.15325	0.99435	3.42430
H	-0.92695	0.88489	5.28057
H	-2.97850	-0.64875	1.81584
H	1.02060	1.48185	3.86127
C	1.28538	1.03558	1.16355
C	1.27350	1.74699	-0.02193
C	2.58491	1.65294	-0.59450
C	3.36483	0.86466	0.29773
N	2.56289	0.51067	1.36986
C	4.70024	0.54273	0.03814
C	5.25855	1.02997	-1.13994
C	4.50749	1.82135	-2.03270
C	3.18051	2.13961	-1.76968
H	5.28383	-0.06049	0.72714
H	6.29377	0.79871	-1.37365
H	4.97770	2.18746	-2.94077
H	2.60695	2.74907	-2.46232
C	2.97236	-0.42407	2.39751
H	3.34440	-1.35034	1.94309
H	3.76772	0.00139	3.02028
H	2.11918	-0.65993	3.03331
Pd	-0.93578	-0.27303	-0.44704
H	1.05831	-4.20033	-2.37470
C	0.64151	-3.60481	-1.55609
C	0.57148	-1.79720	-0.07638
C	-0.99135	-3.62205	0.39045
C	-0.70414	-2.34880	-0.37299
C	0.13241	-4.48514	-0.34828
C	1.55275	-2.42383	-1.07379
H	0.87794	-1.73567	0.96275
H	-0.80579	-3.53496	1.46322
H	-1.99457	-4.02678	0.22903
H	-0.29229	-5.41877	-0.72909

H	0.94443	-4.74054	0.34028
H	1.85642	-1.79102	-1.91611
H	2.46536	-2.73563	-0.55417
C	-0.63726	-2.79886	-1.84685
H	-1.50557	-3.38997	-2.14935
H	-0.48362	-1.98966	-2.57095
I	-2.70469	1.60057	-1.16602
H	0.43023	2.30765	-0.40002

dba-PdI complex **62**

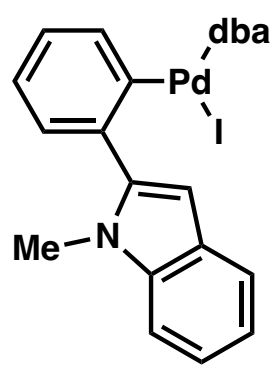

62

H	4.28297	-0.33386	0.01647
C	3.78942	-1.23355	0.36560
C	2.53238	-3.56107	1.24038
C	4.54509	-2.28257	0.89670
C	2.40052	-1.34300	0.27777
C	1.75121	-2.51565	0.71398
C	3.91644	-3.44957	1.33288
H	2.02736	-4.46223	1.57658
H	5.62582	-2.18599	0.95708
H	4.50103	-4.27012	1.73861
C	0.28400	-2.63429	0.76558
C	-0.49554	-2.82558	1.89020
C	-1.86725	-2.67402	1.50019
C	-1.85987	-2.41538	0.09835
N	-0.54565	-2.39615	-0.32844
H	-0.11128	-2.97226	2.89033
C	-3.03628	-2.21514	-0.63107
C	-4.23840	-2.24710	0.06668
C	-4.26836	-2.47651	1.45857
C	-3.09859	-2.68953	2.17932
H	-3.01202	-2.02221	-1.69879
H	-5.16890	-2.07634	-0.46529
H	-5.22585	-2.49299	1.97214
H	-3.13365	-2.87416	3.24971
C	-0.15932	-2.43178	-1.73101
H	-0.45974	-1.51259	-2.23998
H	-0.62682	-3.29242	-2.22332
H	0.92295	-2.51516	-1.80394
Pd	1.33311	0.24383	-0.27688
I	2.43632	0.23927	-2.68788
C	-3.29429	1.62649	-0.27548

H	-2.83170	2.22605	-1.05819
C	-2.45387	1.05038	0.61385
H	-2.80481	0.40451	1.41216
C	-1.01256	1.24049	0.49717
O	-0.47502	1.89997	-0.42025
C	-0.13837	0.50317	1.43926
H	-0.54111	-0.42551	1.82947
C	1.12215	0.87430	1.90624
H	1.58345	0.11134	2.52973
C	-4.74365	1.52283	-0.31807
C	-7.54179	1.35509	-0.51181
C	-5.48859	0.81818	0.64863
C	-5.43218	2.14417	-1.37663
C	-6.81846	2.06019	-1.47514
C	-6.87181	0.73623	0.54980
H	-8.62337	1.28733	-0.58480
H	-4.97949	0.32344	1.46741
H	-4.86536	2.69061	-2.12576
H	-7.33340	2.54288	-2.30033
H	-7.43367	0.18791	1.30036
C	1.86090	2.14468	1.92311
C	3.44372	4.46250	2.05117
C	3.18426	2.09104	2.40267
C	1.33994	3.38668	1.51828
C	2.12667	4.53151	1.58822
C	3.97181	3.23593	2.45853
H	4.05350	5.36019	2.09425
H	3.59291	1.13284	2.71236
H	0.33206	3.45345	1.13267
H	1.71163	5.48384	1.27190
H	4.99350	3.17254	2.82094

PhMe-PdI Complex **63**

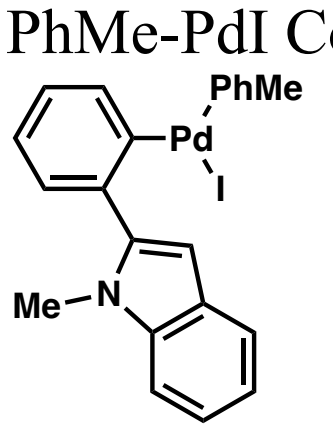

63

H	3.18873	1.71382	0.75176
C	2.30966	2.19626	0.33996
C	0.07080	3.41996	-0.79884
C	2.29968	3.57692	0.11558
C	1.18257	1.44250	0.02153
C	0.04786	2.03268	-0.56460
C	1.18261	4.18685	-0.45692
H	-0.78699	3.88773	-1.27439

H	3.17553	4.16709	0.37063
H	1.18320	5.25489	-0.65360
C	-1.12213	1.20185	-0.89109
C	-1.15235	-0.00742	-1.55692
C	-2.49922	-0.48929	-1.52487
C	-3.26760	0.48561	-0.82692
N	-2.41781	1.51411	-0.45783
H	-0.29876	-0.47872	-2.02106
C	-4.63127	0.31540	-0.56757
C	-5.22777	-0.86003	-1.01561
C	-4.48651	-1.83695	-1.71188
C	-3.13280	-1.65982	-1.97399
H	-5.20835	1.06796	-0.03867
H	-6.28485	-1.02497	-0.82856
H	-4.98604	-2.74021	-2.05015
H	-2.56653	-2.41657	-2.50970
C	-2.83837	2.64249	0.34666
H	-1.96278	3.17560	0.71470
H	-3.42506	2.29519	1.20501
H	-3.45531	3.33870	-0.23434
Pd	1.03792	-0.43934	0.56392
I	2.78415	-1.10329	-1.29646
H	-2.46846	0.08848	1.73781
C	-1.65804	-0.59726	1.95615
C	0.43321	-2.37129	2.47997
C	-1.75639	-1.92098	1.53983
C	-0.51821	-0.12051	2.64375
C	0.52183	-1.03634	2.92076
C	-0.70037	-2.80781	1.78705
H	1.25019	-3.05627	2.68206
H	-2.63166	-2.24586	0.98774
H	1.38396	-0.71125	3.49527
H	-0.75692	-3.83416	1.43842
C	-0.45734	1.30378	3.13866
H	-0.67433	2.00973	2.33223
H	0.53507	1.54450	3.52900
H	-1.18956	1.46959	3.93828

Ethylene–Pd Complex **64**

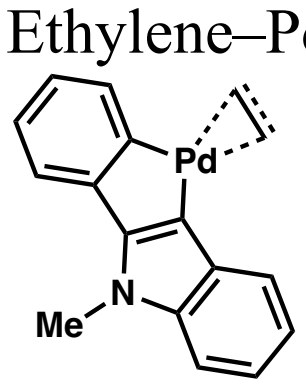

64

H	4.74382	1.54900	-0.00065
C	4.19505	0.61160	-0.00030

C	2.79825	-1.86544	0.00037
C	4.87734	-0.60123	-0.00020
C	2.79744	0.57848	-0.00005
C	2.07391	-0.66068	0.00025
C	4.18592	-1.82944	0.00015
H	2.27008	-2.81597	0.00066
H	5.96360	-0.59861	-0.00041
H	4.75038	-2.75771	0.00023
C	0.68262	-0.32089	0.00043
C	0.60680	1.05977	0.00013
N	1.87751	1.62051	-0.00008
C	-0.72079	1.64419	0.00011
C	-3.44873	2.31109	0.00045
C	-1.71736	0.63126	0.00051
C	-1.12146	2.98289	-0.00026
C	-2.48396	3.31040	-0.00008
C	-3.06122	0.95607	0.00073
H	-4.50511	2.56544	0.00062
H	-0.38371	3.77786	-0.00074
H	-2.78253	4.35493	-0.00037
H	-3.82942	0.18927	0.00110
C	2.19976	3.03143	-0.00091
H	1.79785	3.52786	0.88954
H	1.80033	3.52649	-0.89326
H	3.28359	3.15207	0.00058
C	-2.98461	-2.46422	-0.68421
C	-2.98670	-2.46592	0.68005
H	-3.52695	-1.72168	1.25657
Pd	-1.07402	-1.28759	0.00063
H	-2.57650	-3.30179	1.24238
H	-3.52354	-1.71897	-1.26061
H	-2.57290	-3.29886	-1.24738

References

1. S. Arae, S. Beppu, T. Kawatsu, K. Igawa, K. Tomooka, R. Irie. “Asymmetric synthesis of axially chiral benzocarbazole derivatives based on catalytic enantioselective hydroarylation of alkynes.” *Organic Letters* **20** (2018): 4796–4800.

2. L. McDermott, Z. G. Walters, S. A. French, et al. “A solution to the anti-Bredt olefin synthesis problem.” *Science* **386** (2024): eadq3519.

3. X. Wang, J. Wang, M. Ji, X. Wu, C. Zhu. “Z-selective radical difunctionalization of aromatic alkynes: synthesis of multi-substituted triarylethenes.” *Chemical Communications* **60** (2024): 4894–4897.

4. Q.-B. Zhang, Y.-L. Ban, P.-F. Yuan, et al. “Visible-light-mediated aerobic selenation of (hetero)arenes with diselenides.” *Green Chemistry* **19** (2017): 5559–5563.

5. E. D. Nacsa, T. H. Lambert. “Cross-coupling of sulfonic acid derivatives *via* aryl-radical transfer (ART) using TTMSS or photoredox.” *Organic Chemistry Frontiers* **5** (2018): 64–69.

6. W.-J. He, W.-Z. Qin, S. Yang, et al. “Pd(COD)(DQ): A stable, versatile, and monometallic palladium(0) source for organometallic synthesis and catalysis.” *ChemRxiv* (2024). DOI: 10.26434/chemrxiv-2024-sj081.

7. Z. Liu, X. Zhang, R. C. Larock. “Synthesis of fused polycyclic aromatics by palladium-catalyzed annulation of arynes using 2-halobiaryls.” *Journal of the American Chemical Society* **127** (2005): 15716–15717.

8. Y.-M. Tian, X.-N. Guo, Z. Wu, et al. “Ni-catalyzed traceless, directed C3-selective C–H borylation of indoles.” *Journal of the American Chemical Society* **142** (2020): 13136–13144.

9. H. Miura, M. Chida, T. Shishido. "C3-selective C–H borylation of indoles by supported gold catalysts." *Tetrahedron Letters* **171–172** (2025): 155758.

10. J. R. Butler, D. Erlanson, R. Graceffa, et al. "N-Cyano-7-azanorbornane derivatives and uses thereof." *WO2020036940A1* (2020).

11. G. Luo, L. Chen, A. Easton, et al. "Discovery of indole- and indazole-acylsulfonamides as potent and selective NaV1.7 inhibitors for the treatment of pain." *Journal of Medicinal Chemistry* **62** (2019): 831–856.

12. R. Maruyama, A. Takeda, D. Maruyama, T. Hamura. "Noncross coupling route to biaryls and heterobiaryls via iodoarylation of arynes." *Asian Journal of Organic Chemistry* **14** (2025): e202500263.

13. *Spartan'20*, Version 1.1.5. Wavefunction, 2022.

14. G. Luchini, J. V. Alegre-Requena, I. Funes-Ardoiz, R. S. Paton. "GoodVibes: automated thermochemistry for heterogeneous computational chemistry data." *F1000Research* **9** (2020): 291.

15. S. Grimme. "Supramolecular binding thermodynamics by dispersion-corrected density functional theory." *Chemistry – A European Journal* **18** (2012): 9955–9964.

16. Y.-P. Li, J. Gomes, S. M. Sharada, A. T. Bell, M. Head-Gordon. "Improved force-field parameters for QM/MM simulations of the energies of adsorption for molecules in zeolites and a free rotor correction to the rigid rotor harmonic oscillator model for adsorption enthalpies." *The Journal of Physical Chemistry C* **119** (2015): 1840–1850.

17. D. Masselot, J. P. H. Charmant, T. Gallagher. "Intercepting Palladacycles Derived by C−H Insertion. A Mechanism-Driven Entry to Heterocyclic Tetraphenylenes." *Journal of the American Chemical Society* **128** (2006): 694–695.

18. X. Chen, S. Lei, Y. Chen, et al. "Decarboxylative Cyclization of o-Chlorobenzoic Acids with C,C-Palladacycles Formed by an Aminopalladation/Dealkylation Strategy to Access Dibenzo[a,c]carbazoles." *Organic Chemistry Frontiers* **9** (2022): 3414–3419.

19. P. Pracht, S. Grimme, C. Bannwarth, et al. "CREST–A program for the exploration of low-energy molecular chemical space." *The Journal of Chemical Physics* **160** (2024): 114110.

20. C. Bannwarth, S. Ehlert, S. Grimme. "GFN2-xTB–An accurate and broadly parametrized self-consistent tight-binding quantum chemical method with multipole electrostatics and density-dependent dispersion contributions." *Journal of Chemical Theory and Computation* **15** (2019): 1652–1671.

21. C. Lee, W. Yang, R. G. Parr. "Development of the Colle-Salvetti correlation-energy formula into a functional of the electron density." *Physical Review B* **37** (1988): 785–789.

22. A. D. Becke. "Density-functional exchange-energy approximation with correct asymptotic behavior." *Physical Review A* **38** (1988): 3098–3100.

23. B. Miehlich, A. Savin, H. Stoll, H. Preuss. "Results obtained with the correlation energy density functionals of Becke and Lee, Yang and Parr." *Chemical Physics Letters* **157** (1989): 200–206.

24. S. Grimme, S. Ehrlich, L. Goerigk. "Effect of the damping function in dispersion corrected density functional theory." *Journal of Computational Chemistry* **32** (2011): 1456–1465.

25. R. Ditchfield, W. J. Hehre, J. A. Pople. "Self-consistent molecular-orbital methods. IX. An extended Gaussian-type basis for molecular-orbital studies of organic molecules." *The Journal of Chemical Physics* **54** (1971): 724–728.

26. W. J. Hehre, R. Ditchfield, J. A. Pople. "Self-consistent molecular orbital methods. XII. Further extensions of Gaussian-type basis sets for use in molecular orbital studies of organic molecules." *The Journal of Chemical Physics* **56** (1972): 2257–2261.

27. P. C. Hariharan, J. A. Pople. “The influence of polarization functions on molecular orbital hydrogenation energies.” *Theoretica Chimica Acta* **28** (1973): 213–222.

28. M. S. Gordon, J. S. Binkley, J. A. Pople, W. J. Pietro, W. J. Hehre. “Self-consistent molecular-orbital methods. 22. Small split-valence basis sets for second-row elements.” *Journal of the American Chemical Society* **104** (1982): 2797–2803.

29. M. M. Francl, W. J. Pietro, W. J. Hehre, et al. “Self-consistent molecular orbital methods. XXIII. A polarization-type basis set for second-row elements.” *The Journal of Chemical Physics* **77** (1982): 3654–3665.

30. P. J. Hay, W. R. Wadt. “*Ab initio* effective core potentials for molecular calculations. Potentials for K to Au including the outermost core orbitals.” *The Journal of Chemical Physics* **82** (1985): 299–310.

31. Y. Zhao, D. G. Truhlar. “A new local density functional for main-group thermochemistry, transition metal bonding, thermochemical kinetics, and noncovalent interactions.” *The Journal of Chemical Physics* **125** (2006): 194101.

32. T. Clark, J. Chandrasekhar, G. W. Spitznagel, P. v. R. Schleyer. “Efficient diffuse function-augmented basis sets for anion calculations. III. The 3-21+G basis set for first-row elements, Li–F.” *Journal of Computational Chemistry* **4** (1983): 294–301.

33. R. Krishnan, J. S. Binkley, R. Seeger, J. A. Pople. “Self-consistent molecular orbital methods. XX. A basis set for correlated wave functions.” *The Journal of Chemical Physics* **72** (1980): 650–654.

34. A. D. McLean, G. S. Chandler. “Contracted Gaussian basis sets for molecular calculations. I. Second row atoms, $Z = 11–18$.” *The Journal of Chemical Physics* **72** (1980): 5639–5648.

35. G. W. Spitznagel, T. Clark, P. v. R. Schleyer, W. J. Hehre. "An evaluation of the performance of diffuse function-augmented basis sets for second row elements, Na–Cl." *Journal of Computational Chemistry* **8** (1987): 1109–1116.

36. M. Dolg, U. Wedig, H. Stoll, H. Preuss. "Energy-adjusted *ab initio* pseudopotentials for the first row transition elements." *The Journal of Chemical Physics* **86** (1987): 866–872.

37. Y. Zhao, D. G. Truhlar. "Density functionals with broad applicability in chemistry." *Accounts of Chemical Research* **41** (2008): 157–167.

38. M. M. Yamano, A. V. Kelleghan, Q. Shao, et al. "Intercepting fleeting cyclic allenes with asymmetric nickel catalysis." *Nature* **586** (2020): 242–247.

39. R. Cárdenas Sabando, C. Riplinger, F. Wennmohs, F. Neese, G. Bistoni. "Broadening the scope of the ETS-NOCV scheme: A versatile implementation in ORCA." *Journal of Chemical Theory and Computation* **21** (2025): 7920–7934.

40. F. M. Bickelhaupt, C. Fonseca Guerra, M. Mitoraj, et al. "Clarifying notes on the bonding analysis adopted by the energy decomposition analysis." *Physical Chemistry Chemical Physics* **24** (2022): 15726–15735.

41. F. Weigend, R. Ahlrichs. "Balanced basis sets of split valence, triple zeta valence and quadruple zeta valence quality for H to Rn: Design and assessment of accuracy." *Physical Chemistry Chemical Physics* **7** (2005): 3297–3305.

42. F. Neese. "Software update: The ORCA program system—Version 6.0." *WIREs Computational Molecular Science* **15** (2025): e70019.

43. F. Neese. "Software update: The ORCA program system—Version 5.0." *WIREs Computational Molecular Science* **12** (2022): e1606.

44. F. Neese. “Software update: the ORCA program system, version 4.0.” *WIREs Computational Molecular Science* **8** (2018): e1327.

45. F. Neese. “The ORCA program system.” *WIREs Computational Molecular Science* **2** (2012): 73–78.

46. F. Neese, F. Wennmohs, U. Becker, C. Riplinger. “The ORCA quantum chemistry program package.” *The Journal of Chemical Physics* **152** (2020): 224108.

47. S. Suthar, K. C. Mondal. “Unveiling the bonding scenario in metal–aryne complexes with EDA-NOCV analyses.” *Organometallics* **43** (2024): 261–272.

48. I. Mayer. “Charge, bond order and valence in the *ab initio* SCF theory.” *Chemical Physics Letters* **97** (1983): 270–274.
